# Anatomy of *Rhinochelys pulchriceps* (Protostegidae) and marine adaptation during the early evolution of chelonioids

**DOI:** 10.7717/peerj.6811

**Published:** 2019-05-01

**Authors:** Serjoscha W. Evers, Paul M. Barrett, Roger B. J. Benson

**Affiliations:** 1 Department of Earth Sciences, University of Oxford, Oxford, UK; 2 Department of Earth Sciences, Natural History Museum, London, UK

**Keywords:** Phylogeny, Chelonioidea, Protostegidae, Marine adaptation, Flipper evolution, Intraspecific variation, Taxonomy, Neuroanatomy

## Abstract

Knowledge of the early evolution of sea turtles (Chelonioidea) has been limited by conflicting phylogenetic hypotheses resulting from sparse taxon sampling and a superficial understanding of the morphology of key taxa. This limits our understanding of evolutionary adaptation to marine life in turtles, and in amniotes more broadly. One problematic group are the protostegids, Early–Late Cretaceous marine turtles that have been hypothesised to be either stem-cryptodires, stem-chelonioids, or crown-chelonioids. Different phylogenetic hypotheses for protostegids suggest different answers to key questions, including (1) the number of transitions to marine life in turtles, (2) the age of the chelonioid crown-group, and (3) patterns of skeletal evolution during marine adaptation. We present a detailed anatomical study of one of the earliest protostegids, *Rhinochelys pulchriceps* from the early Late Cretaceous of Europe, using high-resolution μCT. We synonymise all previously named European species and document the variation seen among them. A phylogeny of turtles with increased chelonioid taxon sampling and revised postcranial characters is provided, recovering protostegids as stem-chelonioids. Our results imply a mid Early Cretaceous origin of total-group chelonioids and an early Late Cretaceous age for crown-chelonioids, which may inform molecular clock analyses in future. Specialisations of the chelonioid flipper evolved in a stepwise-fashion, with innovations clustered into pulses at the origin of total-group chelonioids, and subsequently among dermochelyids, crown-cheloniids, and gigantic protostegids from the Late Cretaceous.

## Introduction

Turtles (Testudinata) are a major group of reptiles comprising 335 living species (Turtle Taxonomy Working Group, 2017), with a high ecological diversity, inhabiting marine, freshwater, and terrestrial environments. Early fossil representatives of the stem-group provide evidence for adaptation to both terrestrial and aquatic habitats (Joyce & Gauthier, 2004; Scheyer & Sander, 2007; Li et al., 2008; Lyson et al., 2010, Joyce, 2017), and the ancestral ecology for the crown-group is thought to be freshwater aquatic (Joyce & Gauthier, 2004). Marine ecologies evolved secondarily in several groups (i.e. Angolachelonia (*sensu* Evers & Benson, 2019): Meylan et al., 2000; Anquetin, Püntener & Billon-Bruyat, 2015; Anquetin, Püntener & Joyce, 2017; Evers & Benson, 2019; Bothremydidae: Gaffney, Tong & Meylan, 2006; Rabi, Tong & Botfalvai, 2012; Stereogyina: Sánchez-Villagra et al., 2000; Winkler & Sánchez-Villagra 2006; Gaffney et al., 2011; Ferreira et al., 2015; Chelonioidea: Hirayama, 1994, 1998; Evers & Benson, 2019). However, only one such group is extant: Chelonioidea. Extant chelonioids are highly marine animals with adaptations to a pelagic lifestyle that include modifications in the shell, limbs, and skull (Zangerl, 1980; Hirayama, 1994).

Chelonioids are divided into two main clades, the cheloniids (hard-shelled sea turtles) with six living species, and the dermochelyids with only one living species, the leatherback turtle *Dermochelys coriacea* (Turtle Taxonomy Working Group, 2017). Numerous fossil taxa have been placed variably on the stem of *Dermochelys coriacea*, cheloniids, and chelonioids, but there is little consensus regarding the placement of most taxa. The oldest undisputed stem-group chelonioid is *Toxochelys* spp. from the Coniacian–Campanian of North America (Nicholls, 1988; Brinkman et al., 2006; Kear & Lee, 2006; Joyce et al., 2013; Weems & Brown, 2017; Evers & Benson, 2019), and several Late Cretaceous taxa (e.g. *Allopleuron hofmanni*; Evers & Benson, 2019) have been proposed to be stem-group cheloniids. However, undisputed stem-group taxa for both cheloniids and *Dermochelys coriacea* are generally much younger and date to the Palaeocene–Eocene (e.g. Nielsen, 1959; Joyce et al., 2013; Weems, 2014).

A diverse assemblage of Early–Late Cretaceous marine turtles, the protostegids, has frequently been hypothesised to be on the stem-group of *Dermochelys coriacea* (e.g. Hirayama, 1994, 1998; Lehman & Tomlinson, 2004; Brinkman et al., 2006; Kear & Lee, 2006; Bardet et al., 2013; Cadena, 2015; Cadena & Parham, 2015; Evers & Benson, 2019). This hypothesis has been contested by results from global phylogenetic analyses of testudine interrelationships, which were not focused specifically on marine turtles and that included representatives of most major living and extinct fossil lineages (e.g. Joyce, 2007; Sterli, 2010; Anquetin, 2012). These studies found protostegids in more stemward positions outside of Chelonioidea, on the stem-group of either cryptodires or turtles (see Evers & Benson, 2019 for a recent summary). However, these analyses included only a single Early Cretaceous species of protostegid, *Santanachelys gaffneyi*, in their taxon samples. Only recently have such global analyses included a wider array of protostegids, as well as other fossil sea turtles (Cadena, 2015; Cadena & Parham, 2015; Evers & Benson, 2019), and found protostegids nested within modern chelonioids on the stem-group of *Dermochelys coriacea*, consistent with historical views.

Protostegids are taxonomically and ecologically diverse (e.g. Cope, 1871; Wieland, 1896; Zangerl, 1953a; Collins, 1970; Hooks, 1998; Hirayama, 1998; Tong et al., 2006; Bardet et al., 2013; Cadena & Parham, 2015) and achieved a global distribution early in their history (Collins, 1970; Hirayama, 1998; Kear & Lee, 2006; Cadena & Parham, 2015). Although some protostegid species are known from numerous specimens, their anatomy is quite poorly known, especially with respect to the skull (Seeley, 1869; Lydekker, 1889; Moret, 1935; Collins, 1970; Tong et al., 2006; Cadena & Parham, 2015; but see Raselli, 2018). This is because many specimens are either preserved on slabs with crushed skulls (e.g. Hirayama, 1998; Tong et al., 2006) or in nodules that include completely preserved skulls, but in which most of the internal anatomy is concealed by matrix that is hard to prepare either mechanically or chemically (Collins, 1970; Cadena & Parham, 2015). A thorough understanding of the cranial anatomy of protostegids, especially in early representatives of the group, is important for several reasons. For example, it has been hypothesised that anatomical adaptations in the postcranial skeleton related to the marine habitat of protostegids could represent convergent acquisitions of these features with chelonioids, and falsely support relationships with those taxa (e.g. Cadena & Parham, 2015). Furthermore, cranial features such as the carotid circulation have been important in establishing the phylogenetic relationships of turtles (Jamniczky, 2008; Müller, Sterli & Anquetin, 2011; Rabi et al., 2013).

Here, we used X-ray computed-tomography (CT) to illustrate the cranial and mandibular anatomy of the early Late Cretaceous protostegid *Rhinochelys pulchriceps*. We present a detailed osteological description of this taxon based on digital segmentation of CT scans of six skulls from the Cenomanian aged Cambridge Greensand Member of the West Melbury Marly Chalk Formation in the United Kingdom. This sample includes the holotype specimens of all three species considered valid by the latest revision of the material by Collins (1970). Our work represents the most detailed account of the cranial and mandibular anatomy of any protostegid. This data was used by some of us (SWE & RBJB) in a recent phylogenetic paper (Evers & Benson, 2018, 2019) to inform cranial and mandibular phylogenetic character scores. We extend that phylogenetic work by analysis of an expanded dataset, including the addition and revision of several postcranial characters and the addition of 16 taxa. Our new phylogenetic analysis recovers protostegids as stem-group chelonioids. We provide a taxonomic revision of *Rhinochelys*, and provide evidence for the hypothesis that only one taxon from Europe, *R. pulchriceps*, should be considered valid. Nevertheless, other turtle and sea turtle specimens from the Cambridge Greensand Member of the West Melbury Marly Chalk Formation indicate a higher taxonomic richness of turtles, specifically sea turtles, in the early Late Cretaceous of England. This previously unrecognised diversity prohibits the assignment of isolated postcranial material to *R. pulchriceps* until skull-postcranial associations are found.

## Materials and Methods

### Computed-tomography data and 3D models used in this study

We used high-resolution X-ray CT to generate slice data for six specimens of *Rhinochelys*, including the holotype specimens of *R. pulchriceps*, *R. elegans*, and *R. cantabrigiensis*. Voxel size information is summarised in Table S1.1 in the supplements and full details of the scanning parameters are reported with the deposited scans. 3D models were generated through manual segmentation in the software Mimics 16.0–18.0 (Materialise NV, Leuven, Belgium). 3D models were exported as .ply-files, and the software Blender 2.71 (blender.org) was used to compile figures of digital renderings. CT-slice data as well as 3D models are deposited at MorphoSource (Evers, Barrett & Benson, 2018).

## Systematic Palaeontology

TESTUDINES Linnaeus, 1758
CRYPTODIRA Cope, 1868
CHELONIOIDEA Baur, 1893
PROTOSTEGIDAE Cope, 1873
*RHINOCHELYS* Seeley, 1869

Type species: *Rhinochelys pulchriceps* (Owen, 1851)

**Diagnosis:** *Rhinochelys* can be referred to the Protostegidae based on the presence of a combination of features otherwise only known in protostegids. These include the presence of nasals; the absence of a medial contact between the prefrontals; the absence of a medial process of the jugal; the presence of a long interpalatine contact; the presence of a laterally open foramen palatinum posterius; the presence of processus pterygoideus externus that projects as a free process into the subtemporal fenestra; the presence of a contact of the pterygoid with the mandibular articular surface of the quadrate. *Rhinochelys* differs from all other protostegids by having a preorbital bulge formed by the maxilla and prefrontal.

**Remarks:** The genus *Rhinochelys* is known from a series of specimens from Europe (*R. pulchriceps*) and Lebanon (*R. nammourensis*). All *Rhinochelys* material is from the latest Lower Cretaceous and the earliest Upper Cretaceous, whereby *R. pulchriceps* specimens occur in rocks that date from the late Albian (e.g. Scavezzoni & Fischer, 2018) to the early Cenomanian (e.g. Collins, 1970), and *R. nammourensis* specimens are found in rocks that were dated to be middle Cenomanian in age (Tong et al., 2006). The type species *R. pulchriceps* is known from cranial specimens, some of which include articulated mandibles, but no postcranial material can be assigned to the genus at present (see Discussion). *R. nammourensis* is known from complete specimens (Tong et al., 2006). Because the skulls of *R. nammourensis* are not well known (partially due to the preservation of specimens on slabs of rock), we could not include a detailed revision of *R. nammourensis*, but accept it as a valid species of *Rhinochelys* pending a more detailed cranial comparison with *R. pulchriceps* than given here. *R. nammourensis* can be referred to *Rhinochelys* due to the presence of a prominent preorbital bulge that is otherwise only known in *R. pulchriceps* (see Tong et al., 2006). Features that distinguish *R. nammourensis* from *R. pulchriceps* are listed in the diagnosis for the latter (see below).

**Figure 1:**
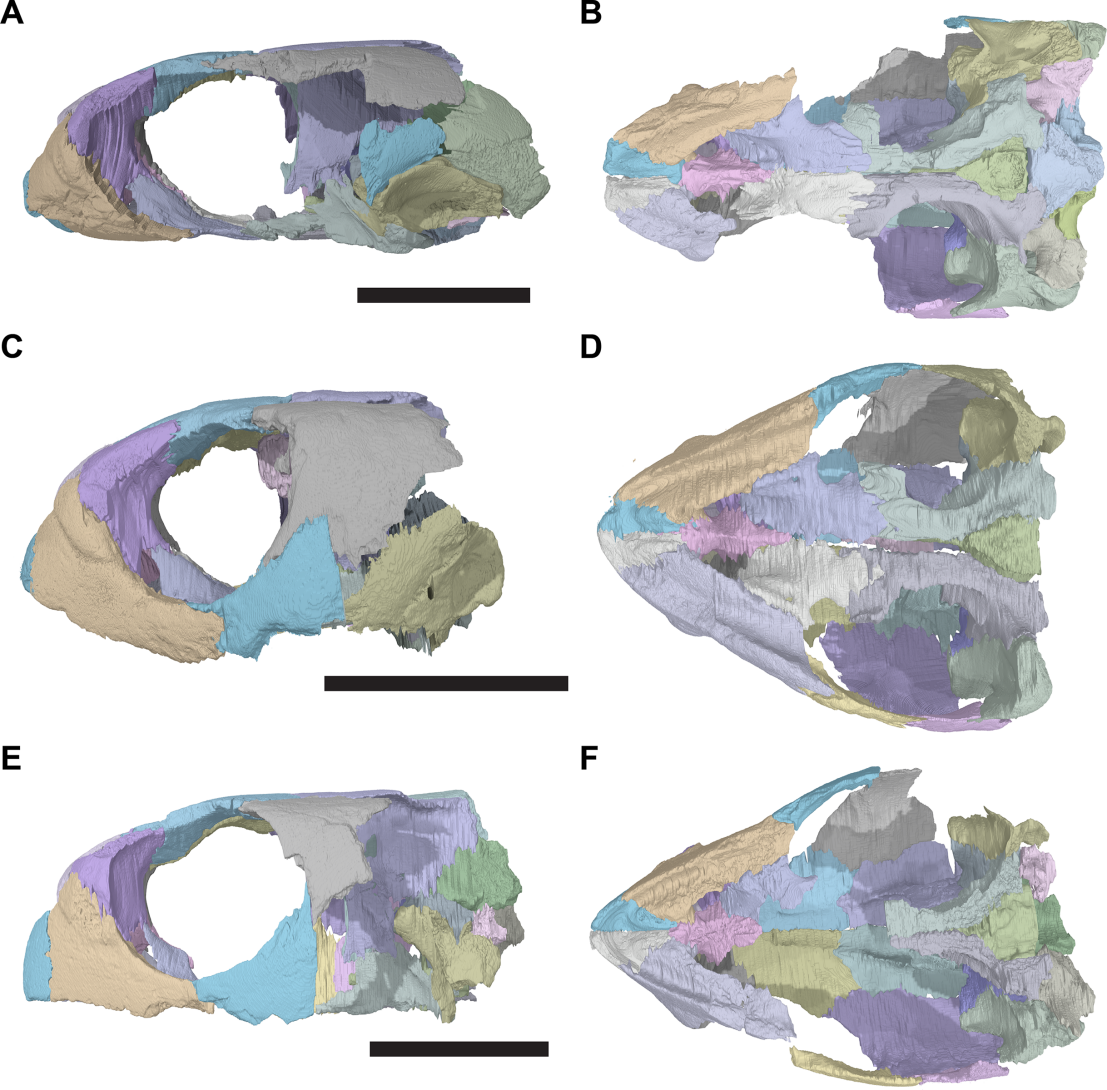
3D renderings of the holotypes of UK species of *Rhinochelys* considered valid by Collins (1970). (A) CAMSM B55775, the holotype of *Rhinochelys pulchriceps*, left lateral view; (B) as (A), but ventral view; (C) NHMUK PV OR43980, the holotype of *R. cantabrigiensis*, left lateral view; (D) as (B), but ventral view; (E) NHMUK PV R2226, the holotype of *R. elegans*, left lateral view; (F) as (E), but ventral view. Scale bars equal 20 mm.

**Figure 2:**
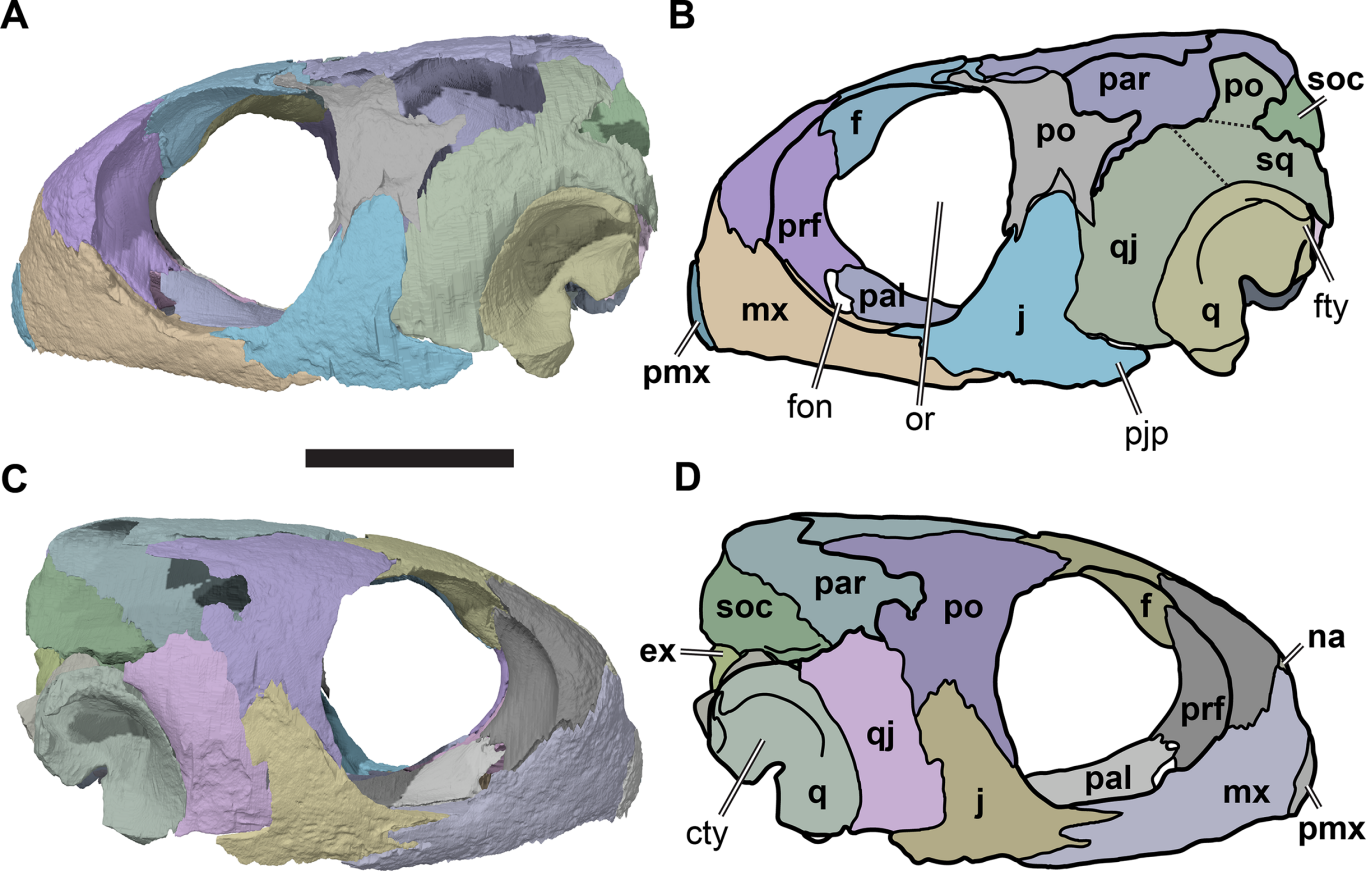
Lateral views of cranium of CAMSM B55783. (A) 3D rendering of left lateral view; (B) interpretative line drawing of (A); (C) 3D rendering of right lateral view; (D) interpretative line drawing of (C). Scale bar equals 10 mm. Note that bones are labelled in bold, and that the left squamosal, quadratojugal, and part of the postorbital in (A–B) are shown as a single model because sutures between these bones were unclear in the CT scan. Abbreviations: *cty*, cavum tympani; *ex*, exoccipital; *f*, frontal; *fon*, foramen orbito-nasale; *j*, jugal; *mx*, maxilla; *na*, nasal; *op*, opisthotic; *or*, orbit; *pal*, palatine; *par*, parietal; *pjp*, posterior jugal process; *pmx*, premaxilla; *po*, postorbital; *prf*, prefrontal; *q*, quadrate; *qj*, quadratojugal; *soc*, supraoccipital; *sq*, squamosal.

**Figure 3:**
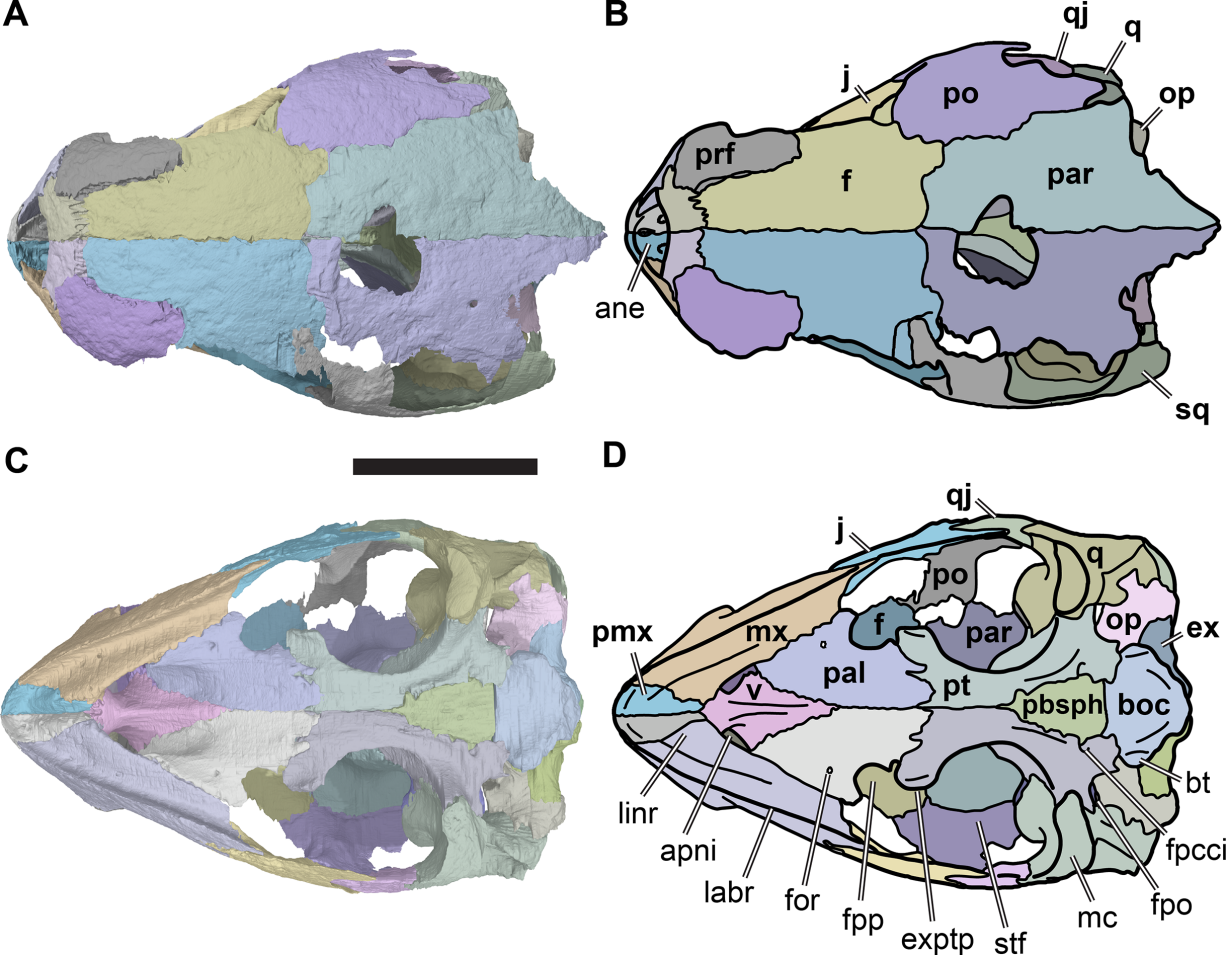
Dorsal and ventral views of cranium of CAMSM B55783. (A) 3D rendering of dorsal view; (B) interpretative line drawing of (A); (C) 3D rendering of ventral view; (D) interpretative line drawing of (C). Scale bar equals 10 mm. Note that bones are labelled in bold. Abbreviations: *ane*, apertura narium externa; *apni*, aperture narium intera; *boc*, basioccipital; *bt*, basal tuber; *ex*, exoccipital; *exptp*, external pterygoid process, *f*, frontal; *for*, foramen; *fpcci*, foramen posterius canalis carotici interni; *fpo*, fenestra postotica; *fpp*, foramen posterius palatinum; *j*, jugal; *labr*, labial ridge; *linr*, lingual ridge; *mc*, mandibular condyle; *mx*, maxilla; *na*, nasal; *op*, opisthotic; *or*, orbit; *pal*, palatine; *par*, parietal; *pbsph*, parabasisphenoid; *pmx*, premaxilla; *po*, postorbital; *prf*, prefrontal, *pt*, pterygoid; *qj*, quadratojugal; *q*, quadrate; *sq*, squamosal; *stf*, subtemporal fossa; *v*, vomer.

**Figure 4:**
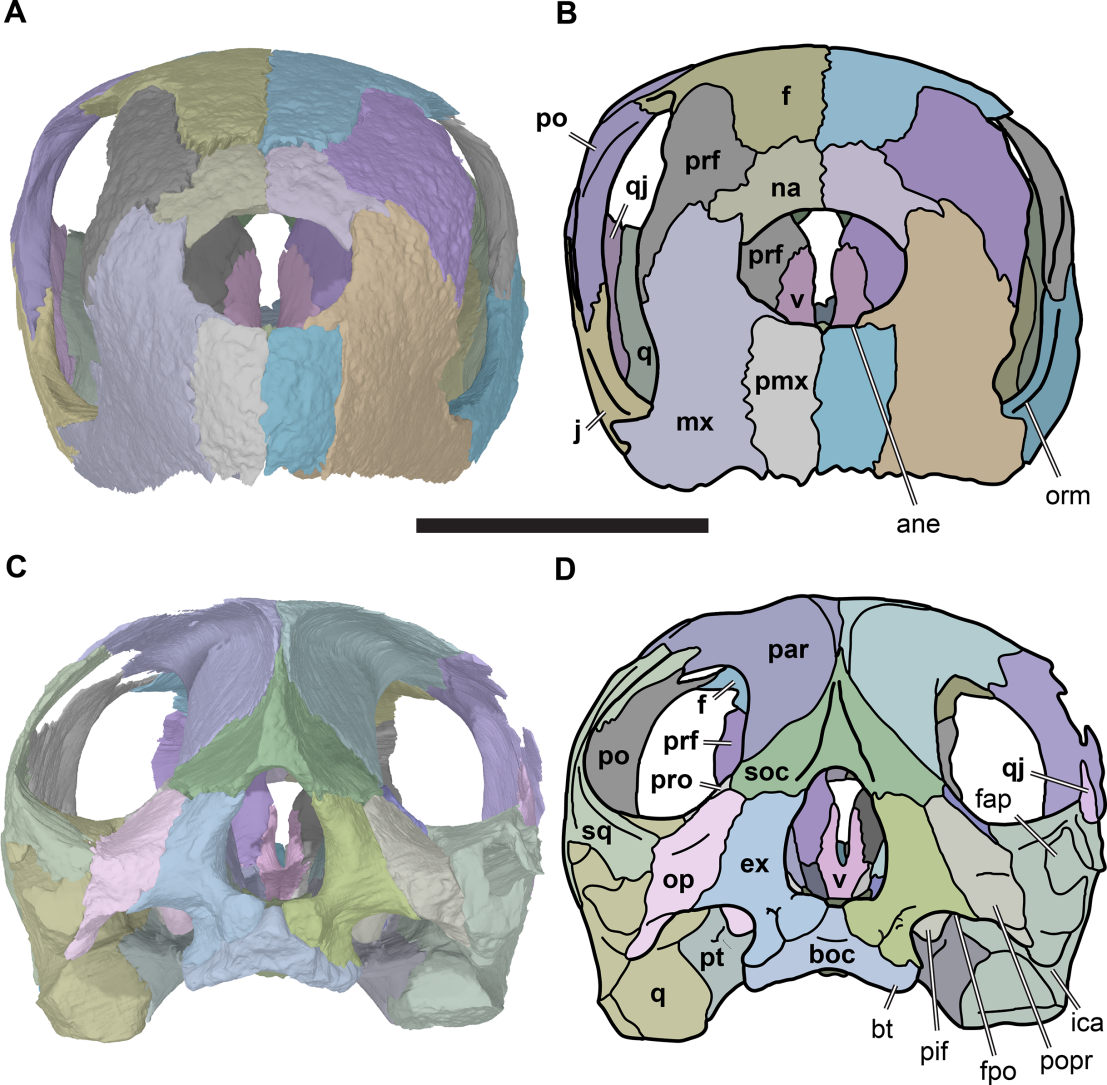
Anterior and posterior views of cranium of CAMSM B55783. (A) 3D rendering fo anterior view; (B) interpretative line drawing of (A); (C) 3D rendering of posterior view; (D) interpretative line drawing of (C). Scale bar equals 10 mm. Note that bones are labelled in bold. Abbreviations: *ane*, apertura narium externa; *boc*, basioccipital; *bt*, basal tuber; *ex*, exoccipital; *f*, frontal; *fap*, foramen antrum postoticum; *fpo*, fenestra postotica; *ica*, incisura columella auris; *j*, jugal; *mx*, maxilla; *na*, nasal; *occ*, occipital condyle; *op*, opisthotic; *orm*, orbital margin; *par*, parietal; *pif*, processus interfenestralis; *po*, postorbital; *popr*, paroccipital process; *prf*, prefrontal; *pro*, prootic; *pt*, pterygoid; *q*, quadrate; *qj*, quadratojugal; *sq*, squamosal; *v*, vomer.

**Figure 5:**
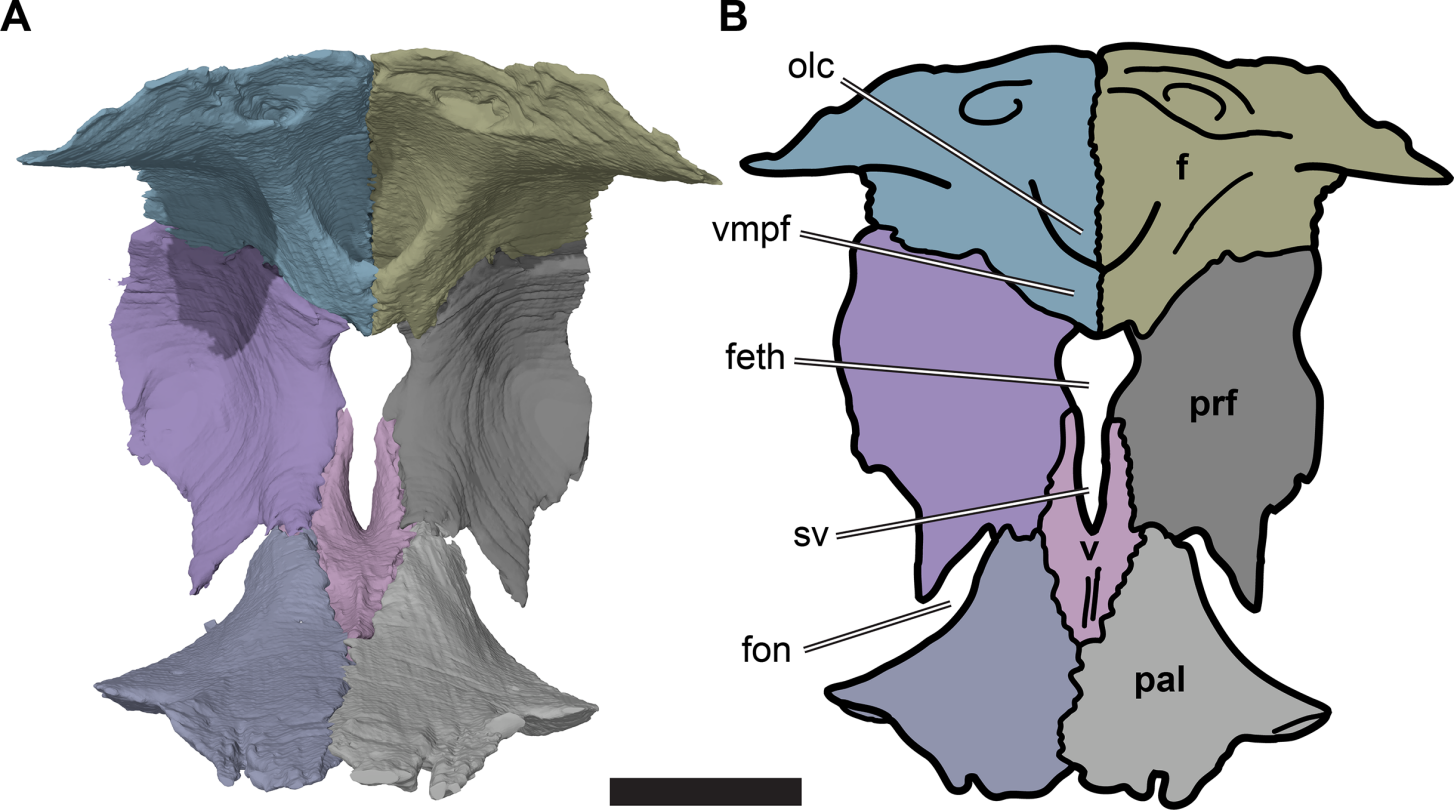
Posterior view of partial anterior part of the cranium of CAMSM B55783 showing the region of the fissura ethmoidalis. (A) 3D rendering; (B) interpretative line drawing. Scale bar equals five mm. Note that bones are labelled in bold. Abbreviations: *f*, frontal; *feth*, fissura ethmoidalis; *fon*, foramen orbito-nasale; *olc*, olfactory canal; *prf*, prefrontal; *pal*, palatine; *sv*, sulcus vomeri; *v*, vomer; *vmpf*, ventromedial process of frontal.

**Figure 6:**
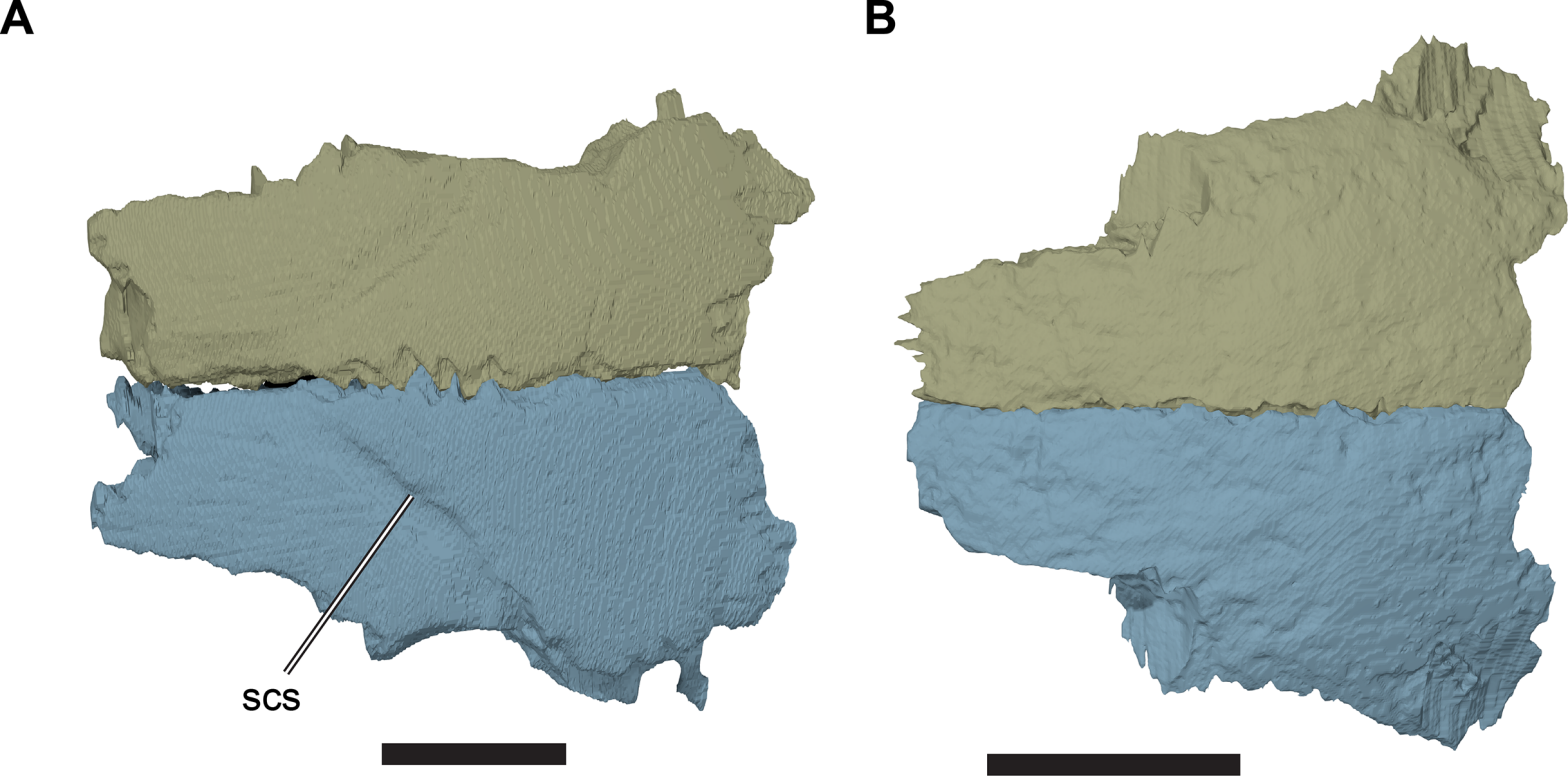
Comparison of frontals in dorsal view. (A) 3D rendering of CAMSM B55775; (B) 3D rendering of CAMSM B55783. Scale bars equal five mm. Abbreviations: *scs*, scute sulcus.

**Figure 7:**
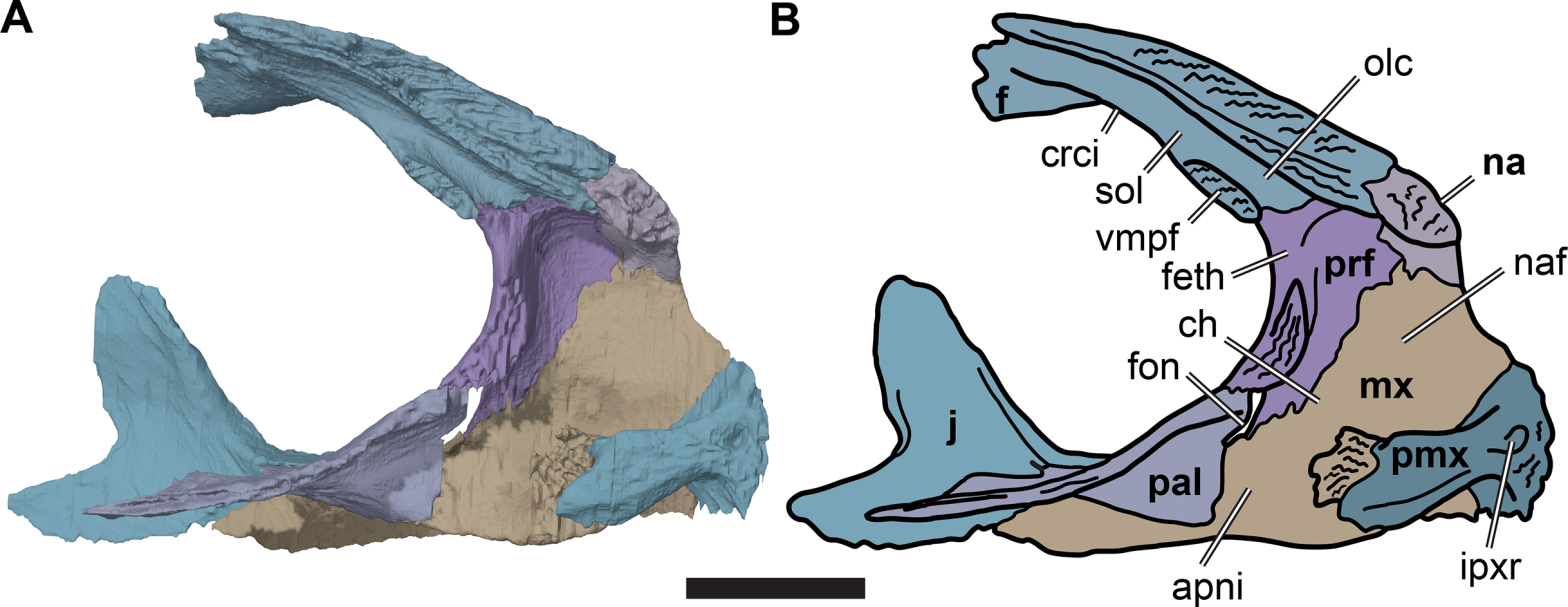
Medial view of the left side of the partial anterior cranium of CAMSM B55783. (A) 3D rendering; (B) interpretative line drawing. Scale bar equals five mm. Note that bones are labelled in bold. Abbreviations: *apni*, apertura narium interna; ch, *choane*; *crci*, crista cranii; *f*, frontal; *feth*, fissura ethmoidalis; *fon*, foramen orbito-nasale; *ipxr*, interpremaxillary recess; *mx*, maxilla; *na*, nasal; *naf*, nasal fossa; *olc*, olfactory canal; *pal*, palatine; *prf*, prefrontal; *pmx*, premaxilla; *sol*, sulcus olfactorius; *vmpf*, ventromedial process of frontal.

**Figure 8:**
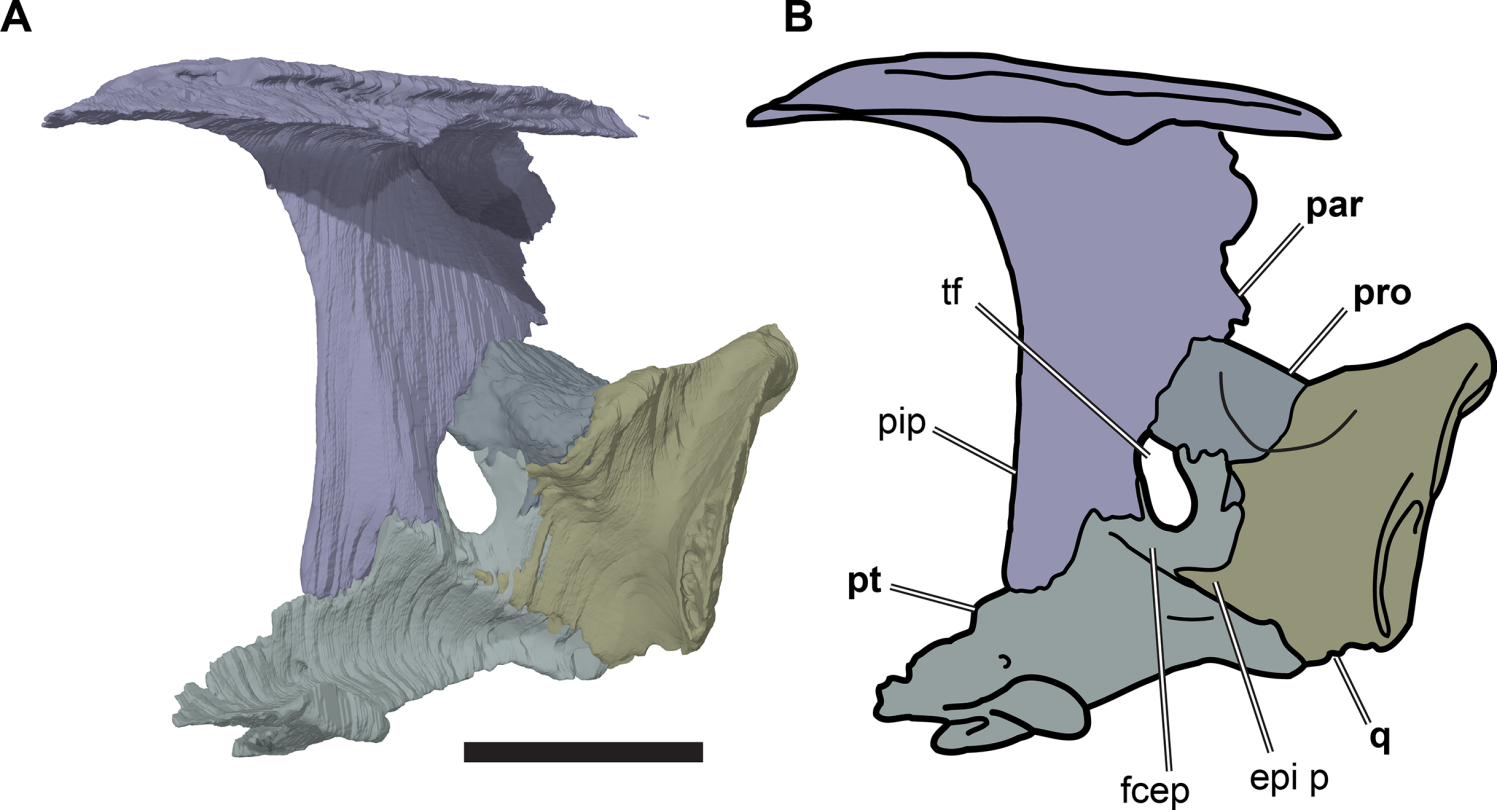
Partial left side of the braincase of NHMUK PV OR35197. (A) 3D rendering; (B) interpretative line drawing. Note that bones are labelled in bold. Scale bar equals five mm. Abbreviations: *epi p*, epipterygoid process of quadrate; *fcep*, fossa cartilaginis epipterygoidei; *par*, parietal; *pip*, processus inferior parietalis; *pro*, prootic; *pt*, pterygoid; *q*, quadrate; *tf*, trigeminal foramen.

**Figure 9:**
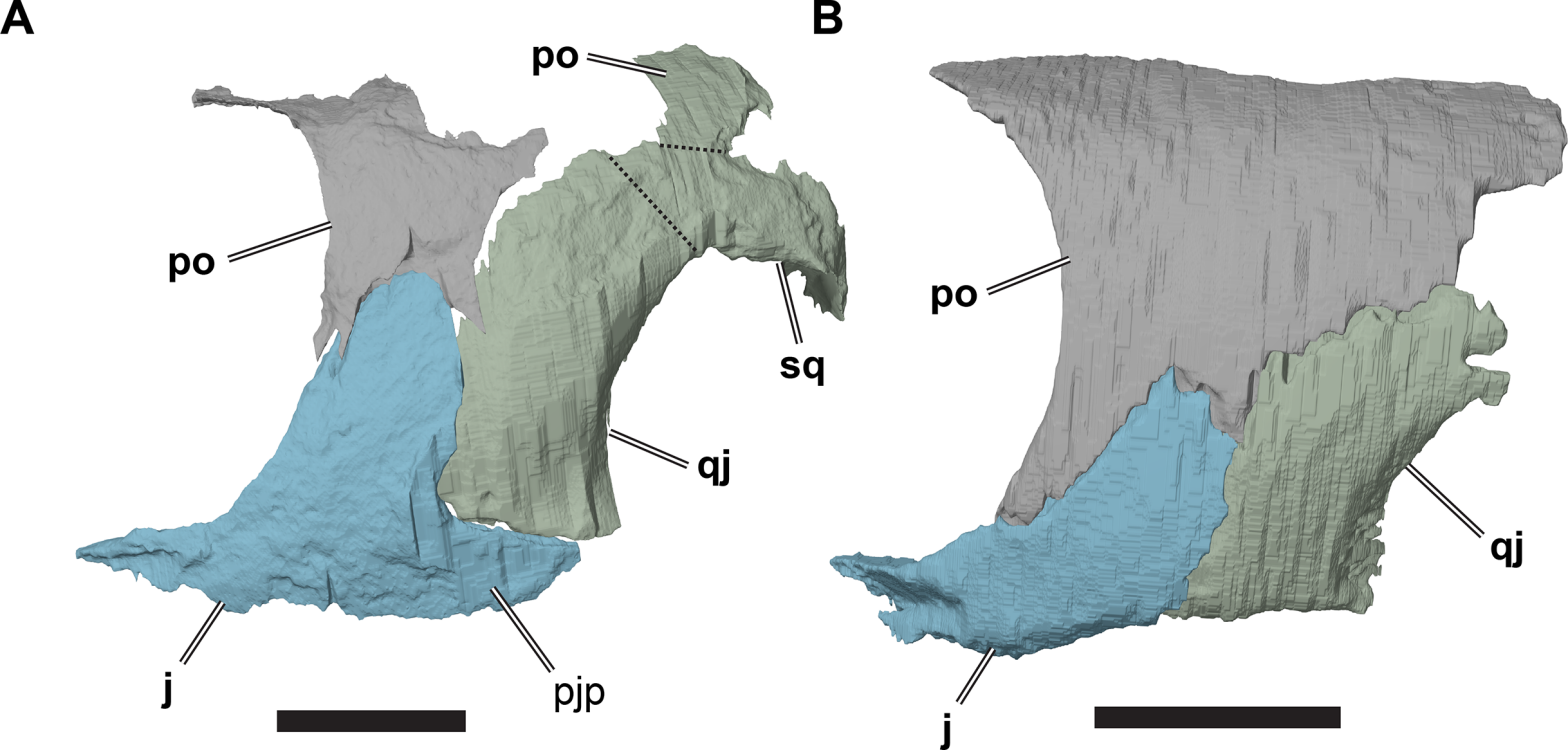
Comparison of left cheek regions showing differences in jugal morphology. (A) 3D rendering of CAMSM B 55783; (B) 3D rendering of NHMUK PV OR35197. Scale bars equal five mm. Note that bones are labelled in bold, and that the left squamosal, quadratojugal, and part of the postorbital in (A) are shown as a single model because sutures between these bones were unclear in the CT scan. Abbreviations: *j*, jugal; *pjp*, posterior jugal process; *po*, popstorbital; *qj*, quadratojugal; *sq*, squamosal.

**Figure 10:**
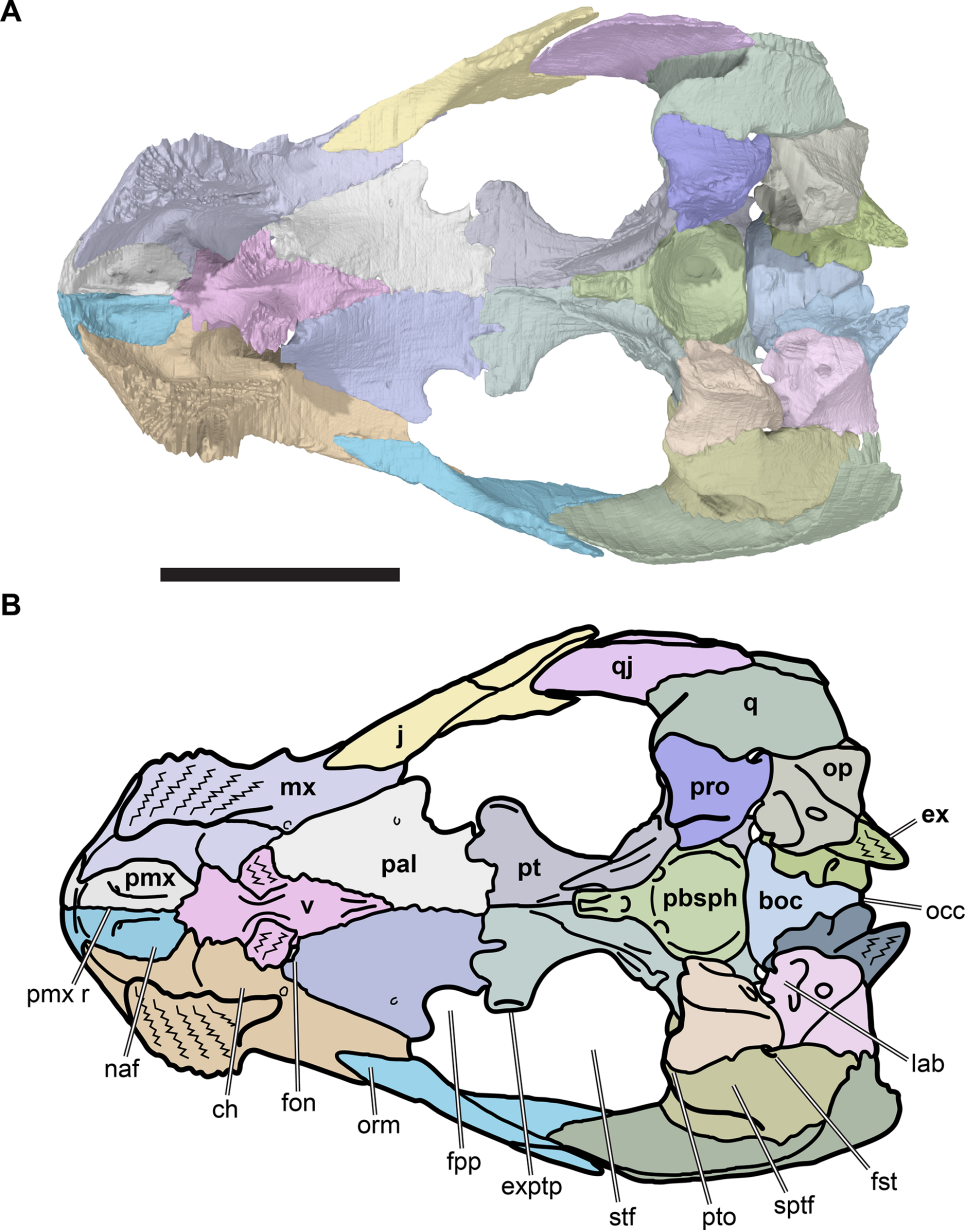
Dorsal view of palate and basicranium of CAMSM B55783. (A) 3D rendering; (B) interpretative line drawing. Scale bar equals 10 mm. Note that bones are labelled in bold. Abbreviations: *boc*, basioccipital; *ch*, meatus choane; *ex*, exoccipital; *exptp*, external pterygoid process; *fon*, foramen orbito-nasale; *fst*, foramen stapedio-temporale; *fpp*, foramen palatinum posterius; *j*, jugal; *lab*, cavum labyrinthicum; *mx*, maxilla; *occ*, occipital condyle; *naf*, nasal fossa; *op*, opisthotic; *orm*, orbital margin; *pal*, palatine; *pbsph*, parabasisphenoid; *pmx*, premaxilla; *pmx r*, premaxilla ridge; *pro*, prootic; *pt*, pterygoid; *pto*, processus trochlearis oticum; *q*, quadrate; *qj*, quadratojugal; *sptf*, supratemporal fossa; *stf*, subtemporal fossa; *v*, vomer.

**Figure 11:**
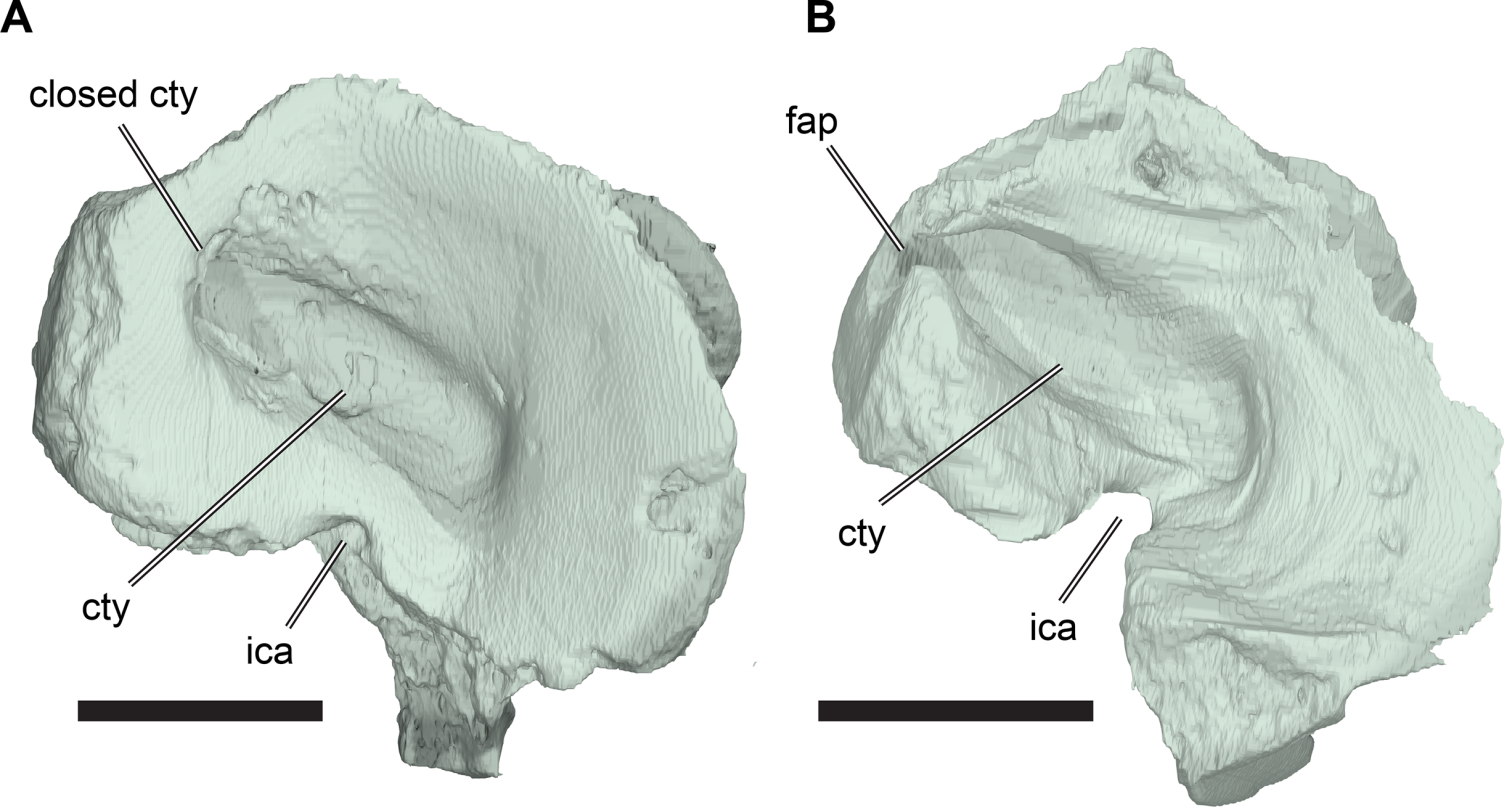
Comparison of quadrates in right lateral view. (A) 3D rendering of right quadrate of CAMSM B55775; (B) 3D rendering of right quadrate of CAMSM B55776. Scale bars equal five mm. Abbreviations: *cty*, cavum tymani; *fap*, foramen antrum postoticum; *ica*, incisura columella auris.

**Figure 12:**
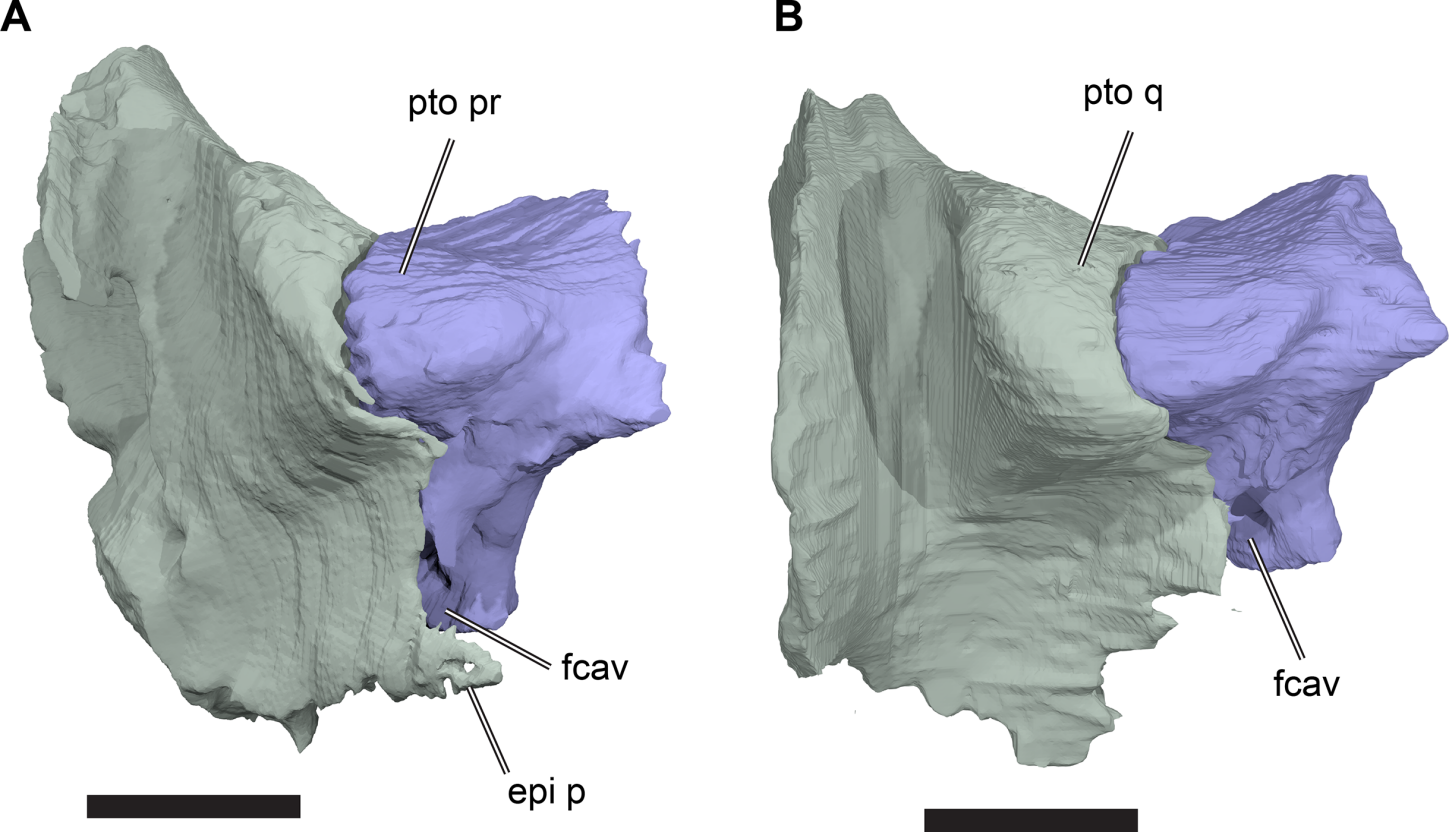
Comparison of trochlear processes in anterior view. (A) 3D rendering of right quadrate and prootic of NHMUK PV OR35197; (B) 3D rendering of right quadrate and prootic of CAMSM B55775. Scale bars equal two mm in (A) and five mm in (B). Abbreviations: *epi p*, epipterygoid process of quadrate; *fcav*, foramen cavernosum; *pto pr*, prootic portion of processus trochlearis oticum; *pto q*, quadrate portion of processus trochlearis oticum.

**Figure 13:**
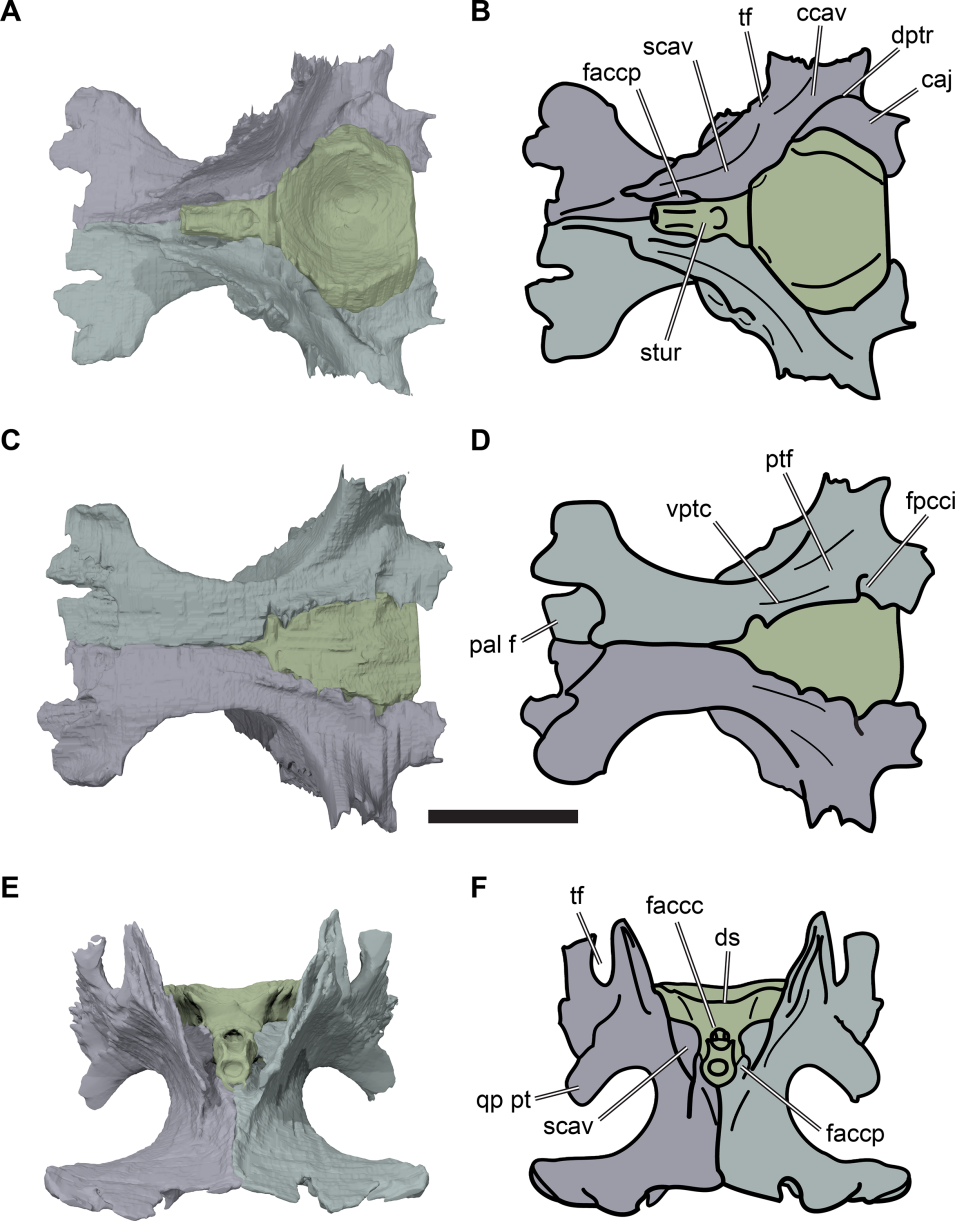
Pterygoids and parabasisphenoid of CAMSM B55783 in articulation. (A) 3D rendering in dorsal view; (B) interpretative line drawing of (A); (C) 3D rendering in ventral view; (D) interpretative line drawing of (C); (E) 3D rendering in anterior view; (F) interpretative line drawing of (E). Scale bar equals five mm. Abbreviations: *caj*, cavum acustico-jugulare; *ccav*, canalis cavernosus; *dptr*, dorsal pterygoid ridge; *ds*, dorsum sellae; *faccc*, foramen anterius canalis carotici cerebralis; *faccp*, foramen anterius canalis carotici palatinum; *fpcci*, foramen posterius canalis carotici interni; *pal f*, palatine facet; *ptf*, pterygoid fossa; *qp pt*, quadrate process of pterygoid; *scav*, sulcus cavernosus; *stur*, sella turcica; *tf*, trigeminal foramen; *vptc*, ventral pterygoid ridge.

**Figure 14:**
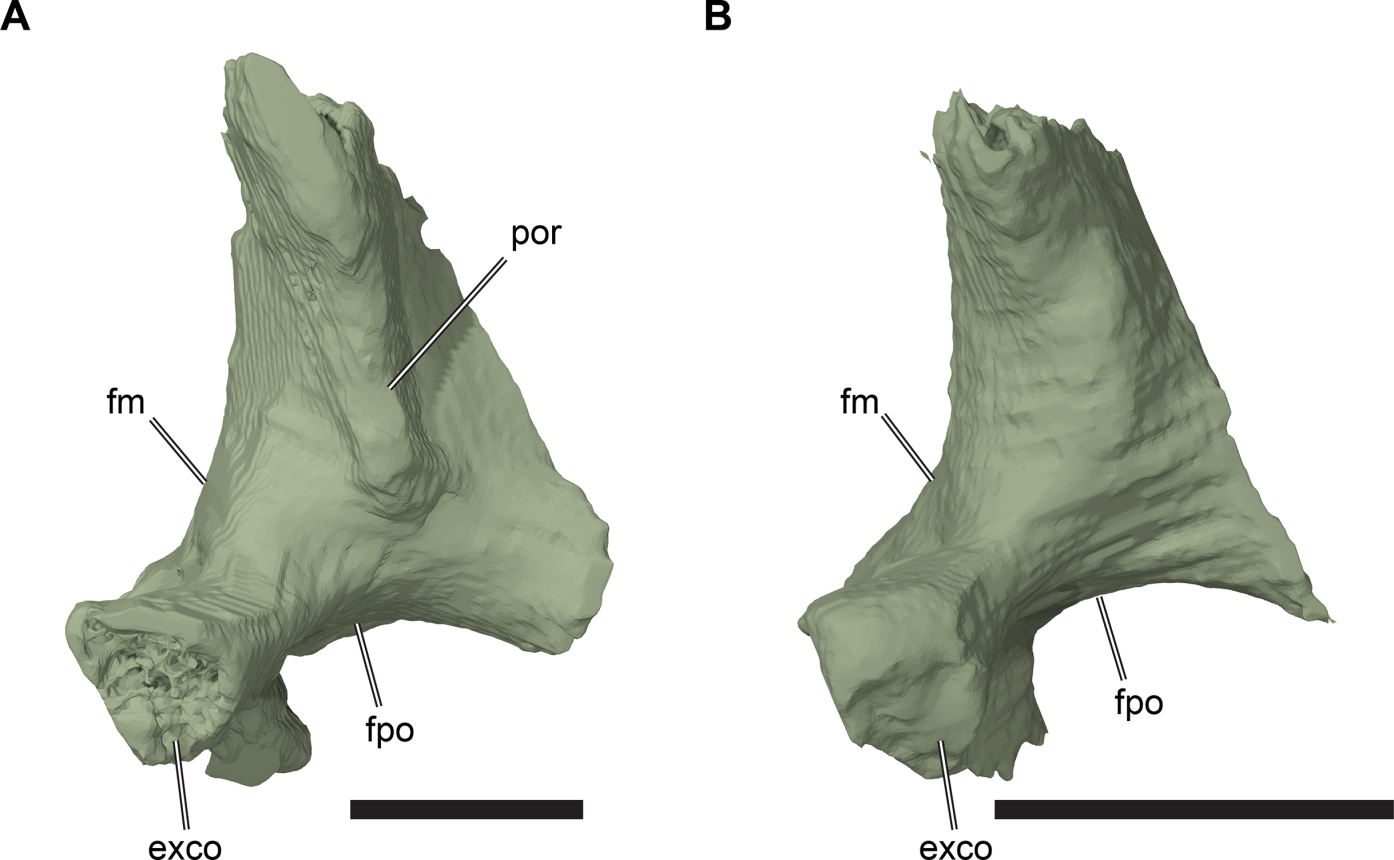
Comparison of right exoccipitals. (A) 3D rendering of CAMSM B55775; (B) 3D rendering of CAMSM B55783. Scale bars equal three mm. Abbreviations: *exco*, exoccipital part of the condylus occipitalis; *fm*, foramen magnum; *fpo*, fenestra postotica; *por*, posterior ridge of exoccipital.

**Figure 15:**
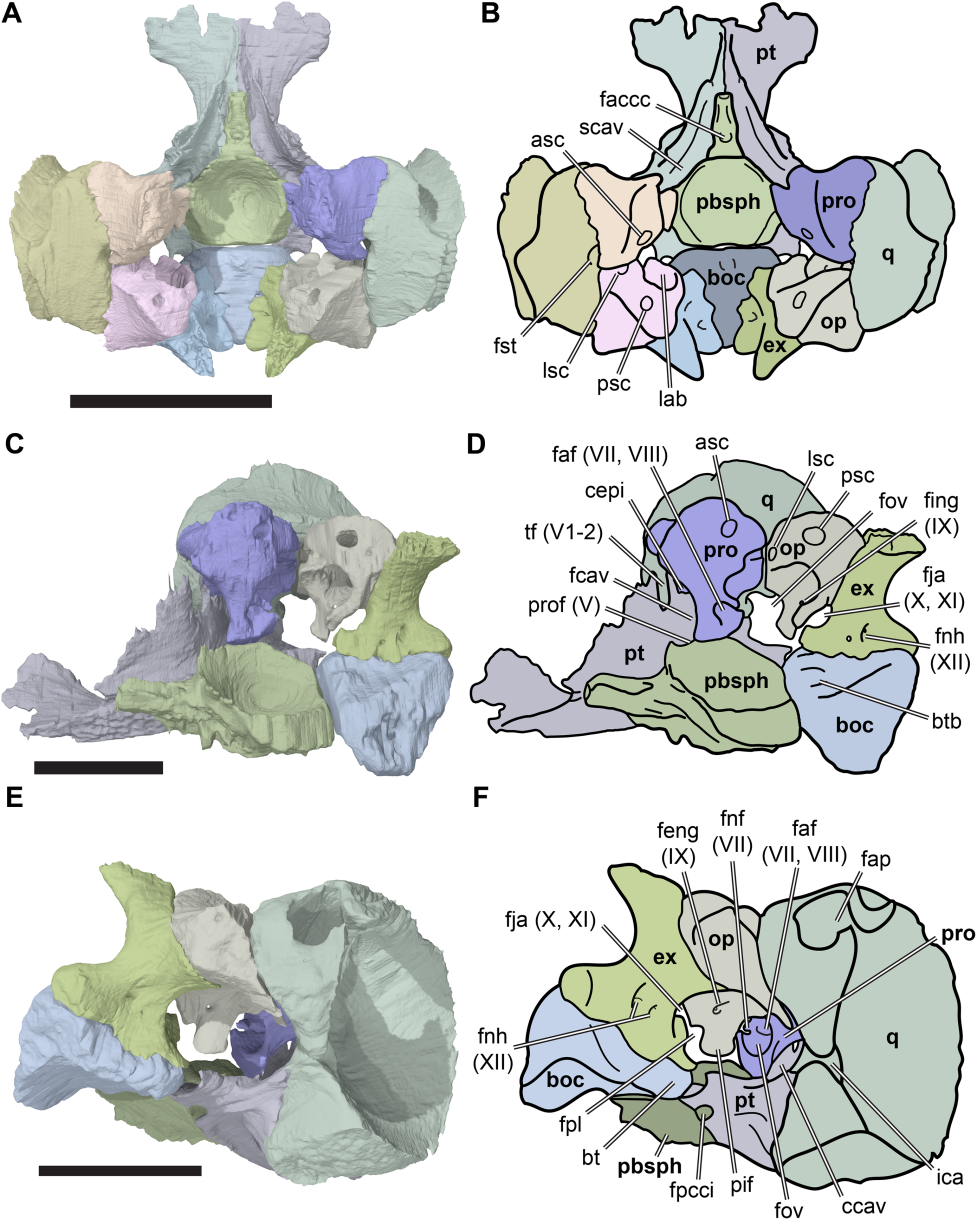
Basicranial details of CAMSM B55783. (A) 3D rendering of basicranium in dorsal view; (B) interpretative line drawing of (A); (C) 3D rendering of the right otic capsule and partial basicranium in medial view; (D) interpretative line drawing of (C); (E) 3D rendering of cavum acustico-jugulare in posteroventrolateral view; (F), interpretative line drawing of (E). Scale bar in top row equals 10 mm, scale bar in middle and bottom row equal five mm. Note that bones are labelled in bold. Abbreviations: *asc*, anterior semicircular canal; *boc*, basicoccipital; *bt*, basal tuber; *btb*, basis tuberculi basalis; *ccav*, canalis cavernosus; *cepi*; cavum epitericum; *ex*, exoccipital; *faccc*, foramen anterius canalis carotici cerebralis; *faf (VII; VIII)*, fossa acustico-facialis; *fap*, foramen antrum postoticum; *fcav*, foramen cavernosum; *feng* (IX), foramen externum nervi glossopharyngei; *fing (IX)*, foramen internum nervi glossopharyngei; *fja (X, XI)*, foramen jugulare anterius; *fnf* (VII), foramen nervi facialis; *fnh (XII)*, foramina nervi hypoglossi; *fpcci*, foramen posterius canalis carotici interni; *fpl*, fenestra perilymphatica; *fov*, fenestra ovalis; *fst*, foramen stepadio-temporale; *ica*, incisura columalla auris; *lab*, cavum labyrinthicum; *lsc*, lateral semicircular canal; *op*, opisthotic; *pbsph*, parabasisphenoid; *pif*, processus interfenestralis; *pro*, prootic; *prof (V)*, prootic foramen; *psc*, posterior semicircular canal; *pt*, pterygoid; *q*, quadrate; *scav*, sulcus cavernosus; *tf (V_1–2_)*, trigeminal foramen.

**Figure 16:**
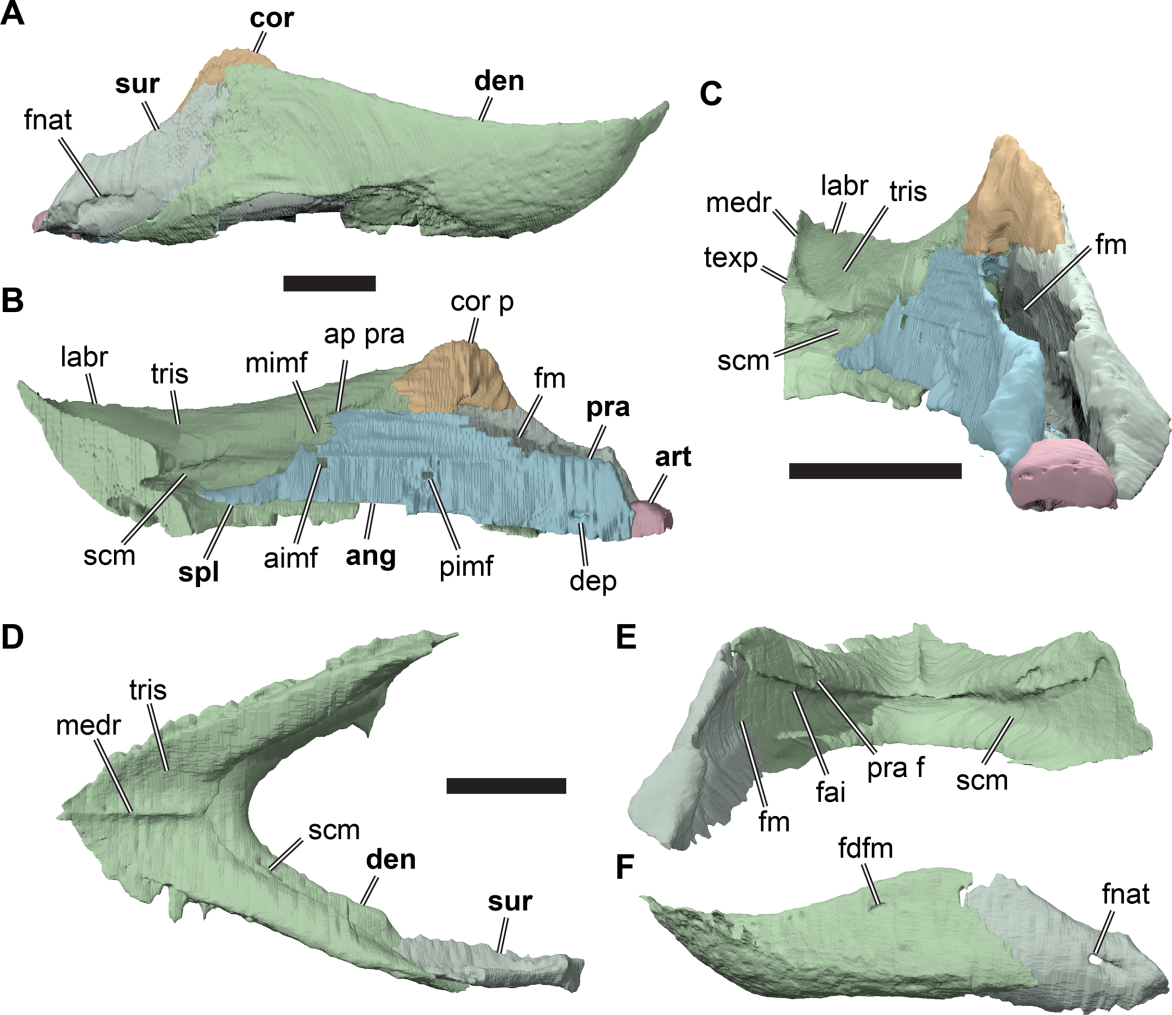
3D renderings of the mandibles of *Rhinochelys*. (A) 3D renderings of right mandibular ramus of CAMSM B55776 in lateral view; (B) medial view; (C) posterior view; (D) 3D renderings of the mandible of CAMSM B55783 in dorsal view; (E) posterior view; (F) left lateral view. All scale bars equal five mm. Note that bones are labelled in bold. Abbreviations: *aimf*, anterior intermandibular foramen; *ang*, angular; *ap pra*, anterior process of prearticular; *art*, articular; *cor*, coronoid; *cor p*, coronoid process; *den*, dentary; *dep*, depression; *fai*, foramen alveolare inferius; *fm*, Meckellian fossa; *fnat*, foramen nervi auricolotemporalis; *for*, foramen; *labr*, labial ridge; *medr*, median dentary ridge; *mimf*, medial intermanibular foramen; *pimf*, posterior intermandibular foramen; *pra*, prearticular; *pra f*, prearticular facet; *scm*, sulcus for the Meckelian cartilage; *spl*, splenial; *sur*, surangular; *texp*, triangular expansion; *tris*, triturating surface.

*Rhinochelys pulchriceps* (Owen, 1851)
*Chelone pulchriceps* Owen, 1851, p. 8, plate 7, figs 1–3
*Rhinochelys pulchriceps* (Owen, 1851)–Seeley (1869): p. xviii; Lydekker (1889): p. 230, plate VIII, Fig. 1; Collins (1970) *partim*: p. 358f, figs 5, 7, plate 67: figs 1–8, plate 68: figs 1–2; Hirayama (1994): figs 1f, 2g, 3g; Hirayama (1997): p. 228, Fig. 7G; Hooks (1998): p. 86f
*Rhinochelys macrorhina* Lydekker, 1889–Lydekker (1889): p. 230, plate VII fig. 7; Collins (1970), fig. 5; plate 68: Fig. 3
*Rhinochelys elegans* Lydekker, 1889–Lydekker (1889): p. 230, plate VIII fig. 5; Collins (1970) *partim*: p. 359, figs 1–2, 5, 8, plate 68: figs 3–7
*Rhinochelys cantabrigiensis* Lydekker, 1889–Lydekker (1889): p. 230, plate VIII fig. 2; Collins (1970) *partim*: p. 359, Fig. 5, plate 68: figs 8–16
*Rhinochelys jessoni* Lydekker, 1889–Lydekker (1889): p. 231, plate VIII, fig. 3; Collins (1970): fig. 3, plate 68: 11–13
*Rhinochelys brachyrhina* Lydekker, 1889–Lydekker (1889): p. 231, plate VIII, fig. 6; Collins (1970), fig. 4, plate 68: fig. 4
*Rhinochelys amaberti* Moret, 1935: p. 606, figs 1–2; plates XXVII–XXVIII; Collins (1970), fig. 6; Scavezzoni & Fischer (2018) *partim*: p. 7, figs 3–6

**Holotype:** CAMSM B55775, a partially preserved skull.

**Type locality and horizon:** Cambridge Greensand Member of the West Melbury Marly Chalk Formation (early Cenomanian: Upper Cretaceous), near Barnwell, Cambridgeshire (Owen, 1851).

**Referred material and range:** Cranial specimens: CAMSM B55771–55774, B55776, B55779–55788, B55791–55796, B55798–55800, B55811, B56274, B56397, B56570–56576, B56578, B56580, B56583; IRSNB GS63–65, IRSNB GS67–68, IRSNB GS70; NHMUK PV R27, R1558, R1806, R2224–2237, R8339, R11521, OR35193–35197, OR41796, OR 43980, OR46371, OR46371a, OR47206; UJF-ID.11167. Mandibles: CAMSM B55809–55810, B55819, B56590, B56593, B59560; NHMUK PV R2238–2239, R2916, OR35183, OR35185a, OR46373–46374; OR49919–49920. All CAMSM, IRSNB, and NHMUK specimens listed are from the early Cenomanian Cambridge Greensand Member of the West Melbury Marly Chalk Formation (UK); the specimen UJF-ID.11167 is from the Aptian–Cenomanian Marnes Bleues Formation (France), and the locality for the specimen is late Albian.

**Differential diagnosis:** *R. pulchriceps* can be distinguished from other known protostegids by the absence of a median ridge or projection on the dorsum sellae of the parabasisphenoid and by the presence of a splenial in the mandible; a median ridge on the dorsum sellae is present, and a splenial is absent in closely related taxa in which the bones exhibiting these features are preserved, such as *Bouliachelys suteri*. However, these features could not be checked for *R. nammourensis* and some other Early Cretaceous protostegids, such as *Santanachelys gaffneyi*. *R. pulchriceps* differs from *R. nammourensis* in having a relatively larger frontal bone which laterally has an anteroposteriorly longer contribution to the orbit; a mediolaterally broader dorsal surface of the parietal which forms more than 50% of the width of the skull roof in dorsal view; posteriorly rounded squamosals that lack the elongate processes seen in *R. nammourensis*. Additionally, *R. nammourensis* has a much deeper posterior skull emargination than *R. pulchriceps*.

## Description

The following description is based largely on the specimens that were CT scanned, i.e. CAMSM B55775 (holotype of *R. pulchriceps*; Figs. 1A and 1B; Data S1: Figs. S1.1 and S1.2), NHMUK PV OR43980 (holotype of *R. cantabrigiensis*; Figs. 1B and 1C; Data S1: Figs. S1.3 and S1.4), NHMUK PV R2226 (holotype of *R. elegans*; Figs. 1D and 1E; Data S1: Figs. S1.5 and S1.6), CAMSM B55776 (referred to *R. elegans* by Collins, 1970; Data S1: Figs. S1.7 and S1.8), NHMUK PV OR35197 (referred to *R. elegans* by Collins, 1970; Data S1: Figs. S1.9 and S1.10), and CAMSM B55783 (referred to *R. cantabrigiensis* by Collins, 1970; Figs. 2–4; Data S1: Fig. S1.11).

### Nasal

The nasals are the anterior-most bones of the skull roof (Figs. 3C and 3D, 4A and 4B; Data S1: Figs. S1.2, S1.4, S1.6, S1.8 and S1.10). They are small elements that contact each other on the midline. Their anterior margins border the external naris dorsally, and the nasals form the roof of the nasal capsule. Each nasal contacts the maxilla anterolaterally, the prefrontal posterolaterally, and the frontal posteriorly.

The nasal shows considerable variation among specimens referred to *R. pulchriceps*. Two gross morphologies can be distinguished, and are described in the following paragraphs, although some features of the nasal are shared by all specimens. In all specimens of *R. pulchriceps*, the nasal is a thin plate with a mediolaterally concave anterior margin that borders the external naris. At its contact with the maxilla, the nasal develops a thin ventrolaterally directed spur that continues for a short distance in the rim of the external naris. In all specimens, the posterior surface of the nasal forms a low transverse crest that slots into a groove in the anterior surface of the frontal. The ventral surface of the nasal is gently excavated and contributes to the fossa forming the dorsal roof of the nasal valve.

One nasal morphotype is shown by CAMSM B55776, CAMSM B55775, and NHMUK PV OR43980, in which the nasal is anteroposteriorly long (approx. 35% longer than wide) and has a relatively constant transverse width across its entire length (Fig. S1.2E). The lateral margin of the nasal is roughly convex in these specimens, and the nasal contacts the frontal posteriorly, the prefrontal posterolaterally, and the ascending process of the maxilla anterolaterally along this margin.

In contrast, the nasals are slightly shorter in CAMSM B55783, NHMUK PV R2226 and NHMUK PV OR35197 (as wide as long at the narial margin; Figs. 4A and 4B; Data S1: Fig. S1.6E). In these specimens, the lateral margin is concave posterolaterally adjacent to the prefrontal. Additionally, the nasal extends laterally between the prefrontal and the ascending process of the maxilla via a short but laterally prominent process. At the level of this process, the nasal becomes mediolaterally as wide as its anteroposterior length. The length–width ratios of the nasals were measured for a large number of specimens, and these data are discussed below (see Discussion).

### Prefrontal

The prefrontals are large bones situated in the anterior part of the skull (Figs. 2, 3C 3D, 4A and 4B; Data S1: Figs. S1.2, S1.4, S1.6, S1.8, S1.10). Each contributes to the mediolaterally oriented vertical wall that separates the orbital fossa from the nasal cavity. Structurally, the prefrontal is a dorsoventrally tall element that connects the bony palate with the dorsal skull roof, contacting the vomer, palatine and maxilla ventrally, and the frontal and nasal dorsally. It contributes to the margins of the nasal cavity, orbit, fissura ethmoidalis, and foramen orbito-nasale.

The prefrontal comprises a long, mediolaterally broad descending process and a shorter posterodorsal process. The ventral process has a large posterior surface that forms the anterior wall of the orbit, and is excavated deeply by the orbital fossa. The anterior surface of the ventral process forms parts of the posterior wall of the nasal cavity, and is therefore posteriorly deeply concave (Fig. S1.12). The external orbital margin of the prefrontal is sharp-edged and is concave when seen in lateral view, contributing to the circular outline of the orbit. This is similar to the condition in the Early Cretaceous protostegid *Bouliachelys suteri*, in which the prefrontal also forms a large section of the orbit. In the Late Cretaceous taxon *Desmatochelys lowii* the prefrontal is generally much smaller and contributes less to the orbit (KUVP 1200; Raselli, 2018). The anterior orbital wall is very gently inclined in *R. pulchriceps*, so that it is oriented posterolaterally, rather than strictly posteriorly, and as a result various internal structures, such as the prefrontal/palate contact and the foramen orbito-nasale, can be seen in lateral view (Fig. 2).

The foramen orbito-nasale is enclosed dorsally and anteriorly by the ventral process of the prefrontal, which therefore has a concave posteroventral margin, dividing it into two terminal rami. The lateral ramus tapers towards its end, and contacts the maxilla laterally. The medial ramus is mediolaterally broader, and contacts the vomer medially and palatine posteroventrally (Fig. 5).

The medial margins of the prefrontals are separated from the midline by the fissura ethmoidalis dorsally and by the ascending processes of the vomer ventrally. Each of these structures occupies around half of the dorsoventral height of the prefrontal medial margin. The fissura ethmoidalis is continuous with the more ventrally located sulcus vomeri, and dorsally enclosed by the olfactory tract formed by the frontals (see Frontal). Together, the fissura ethmoidalis and sulcus vomeri form an opening between the orbital fossa posteriorly and the nasal capsule anteriorly. The fissura ethmoidalis varies slightly in its outline among the *R. pulchriceps* specimens that were CT scanned. In some specimens (CAMSM B55775: *R. pulchriceps* holotype; CAMSM B55776 and NHMUK PV OR35197: both *R. elegans sensu* Collins (1970); NHMUK OR43980: *R. cantabrigiensis* holotype), the combined sulcus vomeri and fissura ethmoidalis form a transversely narrow slit that expands slightly in width as it extends dorsally. By contrast, in CAMSM B55783 (*R. cantabrigiensis sensu* Collins, 1970) and NHMUK PV R2226 (*R. elegans* holotype) the sulcus vomeri has parallel lateral sides, whereas dorsally the fissura ethmoidalis broadens abruptly due to the concave medial margins of the prefrontals, giving the combined opening a keyhole-like outline (Fig. 5).

The posterodorsal process of the prefrontal forms the convex dorsolateral surface of the skull anterodorsal to the orbit. It contacts the maxilla anterolaterally, the nasal anteromedially, and the frontal posteromedially. It has an approximately triangular outline in dorsolateral view and is widest mediolaterally at its anterior contacts with the nasal and maxilla. The contact between the maxilla and nasal anteriorly excludes the prefrontal from the margin of the external naris.

The lateral suture of the prefrontal is weakly interdigitating. The suture between the prefrontal and the anterior process of the frontal is parallel to the skull midline. At the posterior end of the posterodorsal process of the prefrontal, the suture becomes mediolaterally oriented, and slightly convex posteriorly. The prefrontal overlaps the frontal here, whereas the medial contact with the anterior process of the frontal is more complex. Medially, the prefrontal is expanded underneath the anterior process of the frontal and forms a broad, dorsomedially facing contact surface for the frontal.

The sutures of the prefrontal with the nasal and maxilla are highly interdigitated. The suture with the nasal extends anteroventrolaterally from the anterior contact with the frontal to the contact with the maxilla. The maxillary-prefrontal contact expands over the entire height of the prefrontal. The prefrontal articular surface for the maxilla faces anteriorly in its dorsal part, and anteroventrally in its ventral part. The surface narrows ventrally.

### Frontal

The frontals are dorsoventrally thin, anteroposteriorly long bones that form large parts of the skull roof dorsal to the orbits, including the central portions of the dorsal orbital margins (Figs. 3C and 3D; Data S1: Figs. S1.2, S1.4, S1.6, S1.8 and S1.10). The lateral frontal process that contributes to the orbit is relatively larger in *R. pulchriceps* than in *R. nammourensis* (Tong et al., 2006). The frontals of *R. pulchriceps* contact the parietals and postorbitals posteriorly and the nasals and prefrontals anteriorly, and articulate with each other via a weakly interdigitating median suture along their entire anteroposterior length. The length of each frontal exceeds twice its width. The frontals are posteriorly broad but they become mediolaterally narrower anteriorly, terminating in an anterior process that lies anterior to the orbital region. This anterior process has a rectangular outline in dorsal view and is about half the mediolateral width of the posterior portion of the frontal.

The dorsal surface of the frontal is gently curved anteroposteriorly. It is generally smooth, except for a transverse incision that forms the sulcus for a cranial scute. The incision marking the sulcus extends posterolaterally from the skull midline to the orbital margin of the frontal. The part of the frontal posterior to the sulcus is slightly dorsally raised with respect to the surface anterior to it, so that the sulcus appears as a step on the dorsal surface of the frontal. The sulcus is variably developed in the specimens studied. For example, the sulcus is very clearly defined in CAMSM B55775 (*R. pulchriceps* holotype; Fig. 6; Data S1: Fig. S1.2C), but is discernible only as a faint structure on the well-preserved frontals of CAMSM B55783 (*R. cantabrigiensis sensu* Collins (1970); Figs. 3C and 3D, 6).

A small posterolateral spur of the frontal extends between the parietal and postorbital on the external surface of the skull roof. CT scans show that this spur is part of a posterolateral expansion of the frontal that underlays the postorbital. This underlying part is thinner than the externally visible part of the frontal, as it bears a dorsally facing, planar facet for the postorbital that is recessed from the thicker body of the frontal. The externally visible suture of the frontal with the postorbital on the dorsal surface of the skull is gently concave laterally, and extends anterolaterally from the contact with the parietal to the orbital margin. The suture between the frontal and parietal extends medially from the tip of the spur to the skull midline. The suture is weakly convex anteriorly and the posterior margin of the frontal overlaps the dorsal surface of the parietal. This simple contact is reinforced by a deep, anteriorly recessed socket on the ventral surface of the frontal just anterior to its posterior margin, which receives an anterior peg-like process of the parietal.

The frontal contribution to the orbit margin has a concave lateral margin that contributes to the overall circular outline of the orbit. This margin is constricted medially along its length, so that the ventral floor of the orbital fossa can be seen in dorsal view.

The externally visible suture with the prefrontal extends from the orbital margin medially for about half of the width of the frontal, resulting in the constriction of the anterior process of the frontal relative to its posterior portion. From there, the suture curves anteriorly and continues anteriorly up to its contact with the nasal. The internal contact surfaces for the prefrontal are more complex and generally highly interdigitated (see also Prefrontal, above). Laterally, a thin sheet of the frontal extends anteriorly and underlaps the prefrontal ventrally. Medially, the part of the frontal forming the anterior process laps onto the prefrontal. Additionally, a small prong of the frontal inserts into the posteromedial margin of the prefrontal, immediately dorsolateral to the opening for the fissura ethmoidalis (Fig. 7).

The anterior margin of the frontal contacts the nasal. The suture with the nasal is slightly oblique and is oriented anterolaterally/posteromedially rather than strictly mediolaterally. CT images show that the anterior contact surface with the nasal has a transverse groove that receives a transverse ridge from the posterior surface of the nasal. The frontal underlaps the nasal ventrally and thus forms part of the posterodorsal roof of the nasal cavity.

In turtles, and many other tetrapods, the ventral surfaces of the frontals bear paired parasagittal ridges, the cristae cranii, between which the olfactory nerves (CN I) extend anteriorly toward the nasal capsule (Gaffney, 1979; Evans, 2008; Ali et al., 2008). The resulting medially situated, ventrally open trough is called the sulcus olfactorius (Gaffney, 1972, 1979). The sulcus olfactorius of *R. pulchriceps* is located entirely on the frontals and its posterior portion is bounded by low cristae cranii. Each crista cranii separates the orbital fossa laterally from the sulcus olfactorius medially. Anteriorly, the cristae cranii expand into prominent, sheet-like ventromedial processes that contact on the midline in an interdigitating suture (Fig. 5). These processes therefore enclose the sulcus olfactorius ventrally, forming an anteroposteriorly oriented olfactory canal with a transversely wide, oval transverse cross-section. As the cavum cranii is often filled with matrix in many of the specimens studied, the olfactory canal could only be detected in those specimens that were CT scanned, but it is present in all of those specimens (*R. elegans* NHMUK PV R2226 (holotype), NHMUK PV OR35197, CAMSM B55776; *R. pulchriceps* CAMSM B55775 (holotype), *R. cantabrigiensis* NHMUK PV OR43980 (holotype), CAMSM B55783).

To our knowledge, the presence of a ventrally enclosed olfactory canal has not been reported in any other marine turtle. Nevertheless, we also observed this structure in our CT scans of another protostegid, *Notochelone costata* (NHMUK PV R9590). However, it is absent in the protostegids *Bouliachelys suteri* (QMF 31669) and *Desmatochelys lowii* (KUVP 1200; Raselli, 2018). Furthermore, it is also absent in other modern and fossil chelonioids (e.g. *Argillochelys cuneiceps* NHMUK PV OR49465; *Allopleuron hofmanni* NHMUK PV R4213; *Lepidochelys olivacea* SMNS 11070; Evers & Benson, 2018, 2019). However, a similar condition is present in some taxa outside of the marine cryptodires: the lateral margins of the sulcus olfactorius are hypertrophied in some turtles, including *Macrochelys temminckii* (FMNH 22111) and many testudinoids (e.g. Joyce & Bell, 2004) such as *Chelonoidis denticulata* (Gaffney, 1979), and closely approach the midline, without forming an interdigitated contact (Evers & Benson, 2019).

### Parietal

The paired parietals are the posterior-most and largest bones of the skull roof (Figs. 3C and 3D; Data S1: Figs. S1.2, S1.4, S1.6, S1.8 and S1.10). They meet along the midline via a straight suture, which may be slightly interdigitated in its anterior third. The parietal consists of two plates of bone, a horizontal plate and a ventrally directed parasagittal plate that descends ventrally from the horizontal plate as the processus inferior parietalis. The horizontal plate forms the posterior part of the skull roof and contacts the frontal anteriorly and the postorbital laterally. In *R. pulchriceps*, the horizontal plate is mediolaterally relatively wider compared to *R. nammourensis*, and forms most of the width of the skull roof. The processus inferior parietalis connects the skull roof with the palate and braincase. The processus inferior parietalis of *R. pulchriceps* reaches its greatest height anteriorly, where it extends ventrally to contact the pterygoid. The height of the processus inferior parietalis decreases posteriorly and it forms parts of the lateral wall of the braincase posterior to the trigeminal (CN V_2–3_) foramen, contacting the prootic and, more posteriorly, the supraoccipital.

The parietal portion of the skull roof is anteroposteriorly elongate. The anterior suture with the frontal extends from the midline of the skull laterally and slightly posteriorly, until it meets the postorbital. From here, the parietal-postorbital suture is posterolaterally directed for approximately half of its length, so that the parietal becomes progressively broader posteriorly before turning posteriorly. The parietal underlaps the frontal anteriorly and the anterior part of the postorbital anterolaterally. The posterior half of the parietal-postorbital contact does not underlap the postorbital but bears a deep, longitudinal groove for the reception of the postorbital.

The posterior margin of the parietal forms the medial part of the posterior skull emargination. This margin is concave posterolaterally, but the posterior skull emargination is not as deeply developed as in *Desmatochelys lowii* (KUVP 1200; Raselli, 2018). The parietal of *R. pulchriceps* tapers to a thin posterior process along the skull midline (Figs. 3C and 3D). The skull of *R. pulchriceps* is only weakly emarginated compared to many non-marine turtles, so most of the subtemporal fossa is covered by the parietals dorsally. The combined posterior processes of both parietals cover most of the dorsal surface of the supraoccipital (Figs. 3C and 3D).

The processus inferior parietalis forms the lateral surface of the endocranial fossa dorsally. It spans over most of the anteroposterior length of the parietal, extending from the level of the externally visible suture with the frontal to the posterior end of the parietal. The anterior portion of the processus inferior parietalis is oriented ventromedially so that the endocranial cavity is broadest dorsally. The anterior margin of the processus inferior parietalis is weakly concave, with a straight, vertical ventral half and an anterodorsally oriented dorsal part (Fig. 8; Data S1: Fig. S1.13). This is different to the morphology of *Bouliachelys suteri* (QM F31669), in which the anterior margin of the processus inferior parietalis bears two short anterior projections that divide the margin into a series of three concave sections. However, the morphology seen in *Rhinochelys* matches that of *Desmatochelys lowii* (KUVP 1200; Raselli, 2018). As a result of the anteroposteriorly relatively shallow processes inferior parietalis the interorbital fenestrae of *R. pulchriceps* are large, as in other protostegids for which this feature can be observed (e.g. *Bouliachelys suteri*, *Notochelone costata*, *Desmatochelys lowii*). This morphology has been interpreted to be indicative of salt glands in modern sea turtles (Hirayama, 1994) and is thus compatible with the presence of a well-developed salt gland in *R. pulchriceps*. However, large interorbital fenestrae are also present in some other, non-marine turtles (e.g. meiolaniformes) and the presence of salt glands should therefore not be interpreted as the only possible explanation for the presence of a large interorbital fenestra. The anterior portion of the processus inferior parietalis is braced against the horizontal plate of the parietal by a low but robust, dorsoventrally oriented ridge on its lateral surface. This ridge is situated at the anterior end of the processus inferior parietalis, and marks the separation of the anteriorly positioned orbital fossa anteriorly from the temporal fossa posteriorly (Data S1: Fig. S1.13). The anterior part of the processus inferior parietalis articulates ventrally with the crista pterygoidea, a dorsally ascending process of the pterygoid. Several thin bony prongs extend ventrally from the processus inferior parietalis here to interlock with the crista pterygoidea. A single, large, deep socket is also present on the ventral surface of the processus inferior parietalis for the reception of a posterodorsal projection from the crista pterygoidea, which borders the anterior margin of the trigeminal (CN V_2–3_) foramen (Fig. 8).

Posterior to its contact with the pterygoid, the ventral margin of the processus inferior parietalis curves posterodorsally and slightly laterally. As a consequence, the endocranial cavity becomes posteriorly broader and the processus inferior parietalis becomes dorsoventrally shallower. A short portion of the ventral margin of the processus inferior parietalis forms the dorsal margin of the trigeminal (CN V_2–3_) foramen (Fig. 8; see Pterygoid, below, for more detailed description of the trigeminal foramen). However, a posteroventral process extending along the posterior margin of the trigeminal foramen is absent in *R. pulchriceps*. Posterior to the position of the trigeminal foramen the processus inferior parietalis contacts the dorsal surface of the prootic medially, articulating with a shallow groove on the dorsomedial surface of the prootic.

Posterior to the prootic the processus inferior parietalis contacts the supraoccipital ventrally, overlapping the anterodorsolateral surface of the supraoccipital. In this region, the endocranial cavity is mediolaterally constricted by the convergence of the processus inferior parietalis posteromedially. This transition is gradual and at the posterior end of the parietal the bases of the right and left processus inferior parietalis contact each other along the midline. The ventral margins of the right and left processes stay widely separated from the midline, however, so that the lateral surface of each processus inferior parietalis faces dorsolaterally. The transversely thin midline dorsal crest of the supraoccipital is wedged between the left and right parietals. Nevertheless, the parietals retain a midline contact dorsal to the supraoccipital so that the supraoccipital is largely covered by the parietals in dorsal view and is dorsally exposed only at the posterior tip of the crista supraoccipitalis.

### Postorbital

The postorbital is a large element that forms the posterolateral part of the skull roof, including the posterior and posterodorsal margins of the orbit, and covers large parts of the subtemporal fossa dorsally (Figs. 2B, 2D, 3C and 3D; Data S1: Figs. S1.2, S1.4, S1.6, S1.8, S1.10). The postorbital contacts the frontal anteromedially, the parietal medially, the quadratojugal posteroventrolaterally and the jugal anteroventrolaterally. An additional posterolateral contact with the squamosal is not evident from any CT-scanned specimens but is visible in CAMSM B55791, which has complete squamosals and postorbitals preserved (Data S1: Fig. S1.14).

Few specimens of *R. pulchriceps* possess a well-preserved postorbital. Nevertheless, two of the specimens that were CT scanned do have well-preserved postorbitals. In NHMUK PV OR35197 (*R. elegans sensu* Collins (1970) the left postorbital is virtually complete (Data S1: Fig. S1.10A, S1.10C) and CAMSM B55783 (*R. cantabrigiensis sensu* Collins, 1970) includes a right postorbital missing only small parts of its posterior margin (Figs. 2B, 2D, 3C and 3D).

The postorbital is thin and plate-like. As it connects the skull roof with the lateral skull elements it is strongly flexed with a dorsoventrally convex external surface. The medial skull roof portion of the postorbital faces dorsally whereas the ventrolateral parts contacting the jugal and quadratojugal face laterally.

The suture between the postorbital, frontal and the anterior half of the parietal is medially convex, and the postorbital has its greatest medial extent at the frontal-parietal-postorbital contact. Both the frontal and the parietal extend underneath the postorbital, forming a thin shelf of bone with dorsally recessed articular facets for the postorbital. However, the thin lateral margin of the frontal and parietal at the edge of this shelf, just below the externally visible suture line, is anteroposteriorly grooved, and the medial margin of the postorbital inserts into this groove. The posterior half of the suture between postorbital and parietal is posteriorly directed. In this part of the contact the postorbital does not overlap the parietal, but forms an anteroposteriorly oriented crest along its medial surface, which articulates with a groove on the lateral surface of the parietal.

The jugal process of the postorbital curves along the posteroventral margin of the orbit, so that the anterior margin of the postorbital is concave. The jugal process is slightly expanded into the orbit medially, forming an anteromedially facing surface that delimits the orbital fossa posteriorly. The jugal process becomes ventrally thinner and tapers distally to a tip that inserts into a facet on the dorsal surface of the jugal. Both the jugal and the postorbital bear articular facets for each other: the jugal process of the postorbital fits into an anterolaterally recessed facet of the jugal, but the posterior margin of the jugal process is deeply recessed to form a wide groove that receives the anterior margin of the jugal. As a result, the jugal process wraps around the anterior margin of the jugal. Additionally, the postorbital has a ventrally directed sheet-like process posterior to the jugal process that braces against the medial side of the dorsal tip of the jugal. Externally, this part of the postorbital is visible as a ventrally narrowing triangular process that inserts between the jugal and quadratojugal.

The suture between the postorbital and quadratojugal is anterodorsally concave and posterodorsally directed so that the postorbital becomes transversely narrower as the quadratojugal becomes higher dorsoventrally. These bones appear to have a simple overlapping contact, whereby the posteroventrolateral margin of the postorbital laterally overlaps the quadratojugal.

The posterior margin of the postorbital is rarely completely preserved. In NHMUK PV OR35197, the specimen with the best preserved postorbital, the posterior margin of the bone forms a short contribution to the posterior skull emargination adjacent to the parietal. Laterally, this margin develops a posterior notch. It is possible that this notch represents a contact surface for the squamosal, but the latter is not preserved in NHMUK PV OR35197. Alternatively, the notch could be the result of damage.

### Jugal

The morphology of the jugal varies between *R. pulchriceps* specimens. Specifically, a posterior jugal process is not present in all specimens. In the following, the jugal is described on the basis of the condition seen in CAMSM B55783 (*R. cantabrigiensis sensu* Collins (1970); Fig. 2), as most other specimens for which we have CT scans also possess this morphology (NHMUK PV OR43980: *R. cantabrigiensis* holotype; NHMUK PV R2226: *R. elegans* holotype; CAMSM B55776: *R. elegans sensu* Collins, 1970). The deviating pattern is described based on NHMUK PV OR35197 (*R. elegans sensu* Collins, 1970). In CAMSM B55775 the jugals are not preserved.

The jugal is a transversely thin, triradiate bone. It comprises an anterior process that articulates with the maxilla, a posterodorsally oriented ascending process wedged between the postorbital and quadratojugal and a short posterior process that extends ventral to the quadratojugal. As seen in all protostegids from the Early Cretaceous (Evers & Benson, 2019), the jugal lacks a medial process and, as a consequence, it has no contact with either the parietal or pterygoid.

The anterior process of the jugal contacts the maxilla via a deeply interdigitated, bifurcated suture and also forms the posterior part of the suborbital bar. The dorsolateral edge of the anterior process forms a low, sharp crest that bounds the posteroventral portion of the orbital margin. The dorsal surface of the jugal adjacent to this crest extends medially to dorsally overlap the jugal process of the maxilla. This dorsal surface forms the ventral floor of the orbital fossa. It becomes transversely narrower posteriorly and curves posterodorsally, forming the anterior surface of the jugal ascending process.

The lateral surface of the anterior jugal process bears a deep notch for the reception of the laterodorsal ramus of the jugal process of the maxilla. Otherwise, the lateral surface of the jugal is smooth. The lateral surface of the jugal faces slightly ventrolaterally, so that the ascending process of the jugal is positioned slightly more laterally than the ventral portions of the jugal.

The ascending process extends posterodorsally to a point approximately level with orbital midheight. Furthermore, the base of the process is anteroposteriorly broad, occupying about half of the total anteroposterior extent of the jugal. The anterior margin of the ascending process, which forms the posteroventral rim of the orbit, has a concave outline in lateral view, and the anteroposterior width of the ascending process tapers dorsally. The ascending process is inclined posterodorsally at an angle of approximately 70° relative to horizontal. Nevertheless, its posterior edge forms an approximate right angle with the posterior process of the jugal in CAMSM B55783. In NHMUK PV OR35197, which lacks a posterior process, the suture line between the ascending process of the jugal and the anterior surface of the quadratojugal shows a similar inclination. In all *R. pulchriceps* specimens, an anteroventral process of the postorbital overlaps the dorsal half of the anterior margin of the ascending process. The lateral surface of the ascending process is recessed to a shallow facet in the area of this contact (see also Postorbital, above). Posterior to this facet, the dorsal end of the ascending process overlaps the postorbital laterally.

The posterior process of the jugal is short and tapers posteriorly to a pointed tip, as its ventral edge curves posterodorsally (e.g. CAMSM B55783). This process ventrally underlaps the plate-like quadratojugal. Because the quadrate extends ventrally beneath the level of the quadratojugal, and the posterior process of the jugal does not extend posteriorly to contact the quadrate, there is a dorsal notch between the jugal and quadrate that forms a very weakly developed lower temporal emargination. The morphology of the articulation between the quadratojugal and jugal is not clear in any of the specimens with a posterior process or in our CT scans. The phosphatic nodules in which *R. pulchriceps* skulls are preserved are almost always damaged by erosion in this area, making detailed examination impossible. The CT scans of CAMSM B55783 show that the dorsal surface of the posterior process, which faces the quadratojugal, seems to possess a very shallow, longitudinal groove. Thus, it seems likely that the ventral margin of the quadratojugal would have fitted into this groove. In any case, it appears that these bones did not have a tight sutural contact.

NHMUK PV OR35197, a specimen referred to *R. elegans* by Collins (1970), lacks a posterior jugal process (Fig. 9; Data S1: Fig. S1.10). It seems unlikely that the process is broken, as the ventral margin of the jugal is smooth and seems to be completely preserved. Underneath the ventral margin of the jugal a small amount of matrix preserved, which also indicates that the bone did not extend further ventrally and posteriorly. Additionally, the ventral margin of the jugal aligns with the margin of the quadratojugal. Taken together, both bones form a gently concave ventral margin of the temporal region of the skull, but this area is not markedly emarginated.

In all *R. pulchriceps* specimens, the ventral margin of the quadratojugal is situated above the ventral margin of maxilla. In specimens possessing a posterior jugal process, it assumes a position lying approximately between the level of the ventral margins of those bones. In NHMUK PV OR35197, in which the process is absent, the ventral margin of the jugal simply forms a continuously sloping margin between the maxilla and quadratojugal, whereas this transition is more stepped in specimens with a posterior process (Fig. 9). Unfortunately, the presence or absence of the posterior jugal process does not occur consistently or unambiguously among the species holotypes of *Rhinochelys* (*sensu* Collins, 1970), so it is not a taxonomically informative character.

### Quadratojugal

The quadratojugal is a vertically oriented plate in the lateral temporal region of the skull (Figs. 2 and 9; Data S1: Figs. S1.2, S1.4, S1.6, S1.8 and S1.10). It contacts the quadrate posteriorly, the squamosal posterodorsally, the postorbital anterodorsally, and the jugal anteriorly. The quadratojugal covers the subtemporal fossa posterolaterally.

The quadratojugal is damaged in many specimens. Furthermore, the sutures of the quadratojugal with the jugal, postorbital, and squamosal are hard to distinguish in our CT scans. The sutures are clearest in NHMUK PV OR35197, which preserves a complete left quadratojugal that forms the basis of this description.

The quadratojugal is dorsoventrally much taller than it is anteroposteriorly wide. It curves posterodorsally from its origin at the ventral surface of the temporal region so that its anterior margin is convex and its posterior margin is concave. The posterior margin of the quadratojugal is laterally overlapped by the anterior margin of the cavum tympanum of the quadrate over the entire height of the quadratojugal. The quadratojugal has only a short contact with the squamosal (although the squamosal is not preserved in NHMUK PV OR35197). A squamosal is present on the left side of CAMSM B55783, but the squamosal-quadratojugal suture cannot be located in the CT scan of that specimen so the exact shape of the contact between these bones cannot be determined. In CAMSM B55791, the suture between the quadratojugal and squamosal is externally visible and show widely spaced interdigitations.

The anterodorsal margin of the quadratojugal bears a dorsoventrally oriented groove on its lateral surface for contact with the postorbital. The jugal overlaps the anterior margin of the quadratojugal laterally in a simple, planar contact. In specimens possessing a posterior jugal process, such as CAMSM B55783 (see Jugal, above), the ventral margin of the quadratojugal articulates with a shallow groove on the ventral margin of the former. In specimens lacking a posterior jugal process (e.g. NHMUK PV OR35197), the quadratojugal forms part of the ventral skull margin. Although its ventral margin is gently concave, it does not form a significant lower temporal skull emargination. In specimens with a posterior jugal process, the quadratojugal contribution to the ventral skull margin is much smaller, but forms a distinct notch between the quadrate and jugal.

### Squamosal

Of the specimens for which we have CT scans, the squamosal is preserved only in CAMSM B55783 (Figs. 2A and 2C). It is positioned in the posterodorsolateral part of the skull. However, it is incomplete and indistinguishable from the quadratojugal on the basis of CT data, thus limiting our description. Complete squamosals are present in CAMSM B55791 (Data S1: Fig. S1.14), but we did not CT scan this specimen and the surrounding matrix prohibits medial and posterior views of the squamosal.

The squamosal of *R. pulchriceps* contacts the quadratojugal anteroventrally, the postorbital anterodorsally, the quadrate posteroventrally and the opisthotic posteromedially, but a contact with the parietal is absent (CAMSM B55791). The incompletely preserved squamosal of CAMSM B55783 suggests the presence of a dorsomedial process that curves onto the skull roof and covers parts of the subtemporal fossa. The squamosal has a posterior process that covers parts of the quadrate dorsally and posteriorly (CAMSM B55791). The posterior surface of the squamosal is formed as a relatively thin ridge in CAMSM B55791, which curves posteroventrally to form a convex posterior margin in lateral view. Long posterior extensions of the squamosal are absent in *R. pulchriceps*, but present in *R. nammourensis* (Tong et al., 2006). The posterior process of the squamosal of *R. pulchriceps* articulates with a deep groove on the dorsal surface of the quadrate (CAMSM B55783). The tip of the posterior process extends posteroventrally over the posterodorsal surface of the quadrate, thereby covering the open antrum postoticum in CAMSM B55783 and CAMSM B55791. The anterior surface of this part of the squamosal is shallowly excavated to form an anteriorly open fossa that bounds the antrum postoticum posteriorly, which can be seen in the CT scans of CAMSM B55783. However, the squamosal is excluded from the posterodorsal margin of the cavum tympanum, whereas it forms the margin of the cavum tympanum in *Desmatochelys lowii* (KUVP 1200; Raselli, 2018). It is not clear how the squamosal-quadrate contact is formed in specimens with a posteriorly closed quadrate, which implies the absence of a squamosal portion of the antrum postoticum (see Quadrate, below). The posterior process of the squamosal in medially expanded in CAMSM B55783 to form a short, interdigitating contact with the opisthotic at the posterior margin of the subtemporal fossa.

The anterolateroventral extent of the squamosal in *R. pulchriceps* is similar to that of most chelonioids (e.g. *Lepidochelys olivacea*: SMNS 11070) in that it is relatively short. However, the squamosal extends much further anteroventrally in *Desmatochelys lowii* (KUVP 1200; Raselli, 2018), which is one of the few protostegids for which this region has been described.

### Premaxilla

The premaxilla is a paired bone at the anterior end of the skull (Figs. 3A, 3B, 4A and 4B; Data S1: Figs. S1.2, S1.4, S1.6, S1.8 and S1.10). It comprises an anterior portion that contacts the maxilla posterolaterally and the external naris dorsally, and a posterior process that forms the median portion of the triturating surface and parts of the floor of the nasal cavity. This posterior process also contacts the vomer posteriorly.

The left and right premaxillae meet at a midline suture. The suture can be seen in most specimens (e.g. CAMSM B55783, B55791) and is weakly interdigitating, as confirmed by CT scans. The anterior surface of the premaxilla is approximately as high as the external naris dorsoventrally and is weakly convex both transversely and dorsoventrally. The anterior surfaces and labial ridge of the premaxillae curve posterolaterally from the midline until they meet the maxillae, which then extend further posterolaterally at a constant angle (the jaw angle, see below).

The external nares are conjoined to form a single opening that is bordered ventrally by the articulated premaxillae. The narial margin of the joined premaxillae is developed as a transversely oriented, thin crest. The ventral margin of the external naris is mediolaterally concave, contributing to the approximately circular outline of the external naris in anterior view. The dorsal surface of the posterior process of the premaxilla, which forms the floor of the nasal cavity, is concealed by matrix in most specimens. However, it is visible in CT scans and in specimens in which sufficient matrix has been removed (NHMUK PV R1806, NHMUK PV R27).

The ventral floor of the nasal cavity, as formed by the premaxilla, faces posterodorsally. The relevant surfaces of each premaxilla are both anteroposteriorly and transversely concave, so that their mid-parts form shallow depressions either side of a median ridge. This ridge likely anchored an internarial septum that separated the right and left nasal valves. The ridge continues posteriorly on to the dorsal surface of the vomer, terminating just anterior to the sulcus vomeri. The depression on the dorsal surface of each premaxilla extends onto the dorsomedial surface of the maxilla, forming a large fossa that forms the ventral boundary of each nasal valve. The dorsal surface of the premaxilla broadens posteriorly within the nasal cavity. A foramen located anteriorly in the premaxillary depression extends anteroventrally through the premaxilla, exiting as a small foramen on the palatal side of the premaxilla within the triturating surface. It is possible that these foramina are associated with the vomeronasal system. However, this sensory organ is poorly understood even in modern turtles (Schwenk, 2008). The topology, symmetry, and size of these foramina varies among specimens of Rhinochelys pulchriceps, although their position is conserved. This foramen does not seem to represent a foramen praepalatinum, as this opening is usually positioned more posteriorly within the premaxilla or in the suture between the premaxillae and vomer, and it is also usually larger (Gaffney, 1979). The foramen praepalatinum is also absent in modern cheloniids (Gaffney, 1979) and *Dermochelys coriacea* (Nick, 1912, Wegner, 1959; Albrecht, 1976; Evers & Benson, 2018).

The median contact of the premaxillae within the nasal cavity is interrupted shortly posteroventral to the external naris, where a slit-like median opening extends anterolaterally into the inter-premaxillary suture. This opening extends anteroventrally into a recess between the premaxillae. The function of this recess is unknown, but no further canals exiting it are visible in our CT data.

The premaxilla articulates laterally with the maxilla over its entire dorsoventral height in a vertical, highly interdigitating suture that is visible externally in all specimens. The ventral surface of the premaxilla forms the anteromedial portion of the triturating surface. The suture between the premaxilla and maxilla extends posteromedially across the palate, crossing the triturating surface. As a consequence, the palatal portions of the premaxillae taper posteriorly. The triturating surface is anterolaterally bounded by the labial margin of the premaxilla, which is elevated to a sharp ridge (the labial ridge). The labial ridge curves posterolaterally from the midline, and continues onto the maxilla, where it retains an almost straight posterolateral course. The angle between the right and left maxillary labial ridges is the jaw angle of Collins (1970: see Maxilla, below). Immediately lingual to the labial ridge, paralleling its course, and also continuous from the premaxilla to the maxilla, is a deep groove (the labial groove). This groove accommodates the sharp-edged margin of the mandible when the jaws are in occlusion. The groove is deepest mesially on the premaxilla and becomes shallower distally on the maxilla. A second more shallowly developed ridge, the lingual ridge, defines the lingual margin of the labial groove. The lingual ridge is located primarily on the maxilla, with only a small portion extending onto the premaxilla.

An anteroposteriorly oriented median groove is present on the palatal surface of the premaxillae and was termed the premaxillary groove by Collins (1970). This groove is also present in some other sea turtles and, because it intersects with the anteriorly convex labial groove, the combined form of the palatal grooves has sometimes been described as being ‘anchor-shaped’ (Lynch & Parham, 2003). The premaxillary groove of *R. pulchriceps* terminates abruptly where the premaxillae meet the vomer posteriorly, jointly forming a transversely convex bar between the internal nares.

### Maxilla

The maxilla forms large parts of the snout (Figs. 2, 3A and 3B; Data S1: Figs. S1.2, S1.4, S1.6, S1.8 and S1.10). Anteromedially, it contacts the premaxilla, with which it forms the triturating surface and palatal ridges on the ventral side of the snout. Posteromedially, the left and right maxillae contact a columnar ventral process of the vomer just anterior to the internal nares, which prevents midline contact of the maxillae on the triturating surface, although they do approach each other closely. The maxilla has a short, anteriorly positioned ascending process, which is posterodorsally directed and contacts the nasal and prefrontal. The ascending process contributes to the margins of the external naris anteriorly and to the orbit posteriorly. Posteriorly, the maxilla possesses a relatively slender jugal process that extends ventral to the orbit. The jugal process interlocks posteriorly with the jugal and contacts the palatine along its medial margin. Within the orbital fossa, the maxilla contributes to the foramen orbito-nasale. The medial surface of maxilla also forms part of the nasal cavity, the choanae, and the internal naris.

The maxillae are directed posterolaterally, diverging from one another at an angle of 33–57°, so that the snout becomes broader posteriorly. The divergence angle between the maxillae was referred to as the jaw angle by Collins (1970: p. 357), who interpreted differences in this angle as one of the features separating the three species of *Rhinochelys* recognised in that paper (see Discussion, below). The maxilla forms most of the triturating surface, which is composed of a sharp labial ridge, a shallower lingual (tomial) ridge and a labial groove separating these ridges (Figs. 3A and 3B). The labial ridge of the maxilla is continuous with the labial ridge of the premaxilla and forms a high but slender cutting edge. The labial ridge becomes more prominent posteriorly and continues to the posterior end of the maxillary jugal process. *R. pulchriceps* specimens vary in the curvature of the maxillary labial ridge; while the ridge is completely straight in many specimens (e.g. *R. elegans* holotype, NHMUK PV R2226), others show marked curvature in which the labial ridge is ventrally convex (e.g. *R. cantabrigiensis* holotype, NHMUK PV OR43980). Immediately lingual to the labial ridge is the labial groove, which parallels the course of the labial ridge and is also continuous between the premaxilla and maxilla. The labial groove gets shallower posteriorly, as it is bordered on the lingual side by the lingual ridge, which also gets lower posteriorly. The lingual ridge is a low plateau that forms most of the grinding or triturating surface and has its deepest and broadest extent at the level of the vomer. This is similar in *Bouliachelys suteri* (QM F31669; Kear & Lee, 2006), but different in *Desmatochelys lowii* (KUVP 1200; Raselli, 2018) in which the lingual ridge is narrow, robust, and deep. The labial and lingual ridges of *R. pulchriceps* are not parallel; the medial margin of the lingual ridge diverges from the midline at a higher angle than the labial ridge. Thus, the triturating surface gets progressively narrower posteriorly as the ridges converge posteriorly. The triturating surface is penetrated by many small neurovascular foramina. The anterior half of the medial margin of the lingual ridge forms the lateral margin of the internal naris, which is otherwise bounded by the vomer medially and the palatine posteriorly. The posterior half of the medial margin of the lingual ridge is dorsoventrally slightly expanded and forms the articular surface for the maxillary process of the palatine.

The ascending process of the maxilla extends posterodorsally from the maxillary body. Its anterior margin forms part of the lateral margin of the external naris. This margin is medially concave, contributing to the near circular outline of the external naris. At its anterior end, the ascending process contacts the nasal via a short suture. The latter slots into the ascending process via two bony prongs, the medial of which extends ventrally for several millimetres along the margin of the external naris, while the lateral prong inserts more posteriorly between the maxilla-prefrontal contact. The maxilla achieves its greatest dorsoventral height at the nasal-maxilla-prefrontal suture. Posterior to this contact, the maxilla forms a highly interdigitating, posteroventrally sloping suture with the prefrontal. The ascending process provides only a minor contribution to the orbital margin. CT scans (e.g. CAMSM B55783, NHMUK PV OR35197) show that the maxilla has an anteroposteriorly oriented incision on its contact surface with the prefrontal. This incision runs parallel to the posterodorsomedial margin of the maxilla, into which the prefrontal slots in addition to the many finger-like extensions on both bones that can be seen externally by tracing the undulating path of the suture.

The lateral surface of the ascending process is separated from the lateral surface of the maxillary body by a moderately deep sulcus. The sulcus runs from the maxillary-nasal suture to the orbital margin a few millimetres below the maxilla-prefrontal suture and is ventrally convex. The ascending process, defined as the maxillary bone above this sulcus, is convexly curved in all directions, so that it appears swollen with a surface that is laterally expanded with respect to the maxillary body. This preorbital bulge is an autapomorphy of *Rhinochelys* (Collins, 1970; Tong et al., 2006). The surface of the ascending process is confluent with those of the nasal and prefrontal. Therefore, the sulcus distinguishes a slightly lower beak region, formed by most of the lateral surface of the maxilla, the jugal process of the maxilla, and the premaxilla, from a slightly protruding forehead region formed by the ascending process of the maxilla, the nasals, and the prefrontals. The maxillary sulcus is present in all specimens, although its depth, as well as the degree to which the ascending process is bulged, differs between them. This variation does not seem to be ontogenetic, as deep sulci and high degrees of swelling appear in both small and large specimens (see Discussion).

The medial surface of the maxilla is shallowly excavated by a fossa at the base of the ascending process (Fig. 7). This fossa is continuous with the fossa on the dorsal surface of the premaxilla: together they form the ventral half of the nasal valve. At its posterior contact with the premaxilla, the maxilla forms a short but stout medial process. This process joins the ventral columnar process of the vomer laterally and articulates with it in a highly interdigitated fashion, as evident from the CT scans.

The jugal process of the maxilla extends from the maxilla-prefrontal suture in the orbital margin posteriorly to the contact with the jugal. Hence, the jugal process extends ventral to the orbit, forming the anterior portion of the suborbital bar. Its dorsal edge forms a slightly raised crest, so that the orbit is well defined anteroventrally and ventrally. This dorsal margin is concavely rounded, contributing to the circular outline of the orbit. The jugal process becomes shallower posteriorly. Medial to the orbital margin, the jugal process is transversely expanded to form a narrow shelf that frames part of the orbital fossa. Anteromedially, this shelf participates in the formation of the foramen orbito-nasale. The maxillary shelf framing this foramen is also pierced by a tiny circular opening, the foramen alveolare superius (Gaffney, 1972, 1979; Albrecht, 1976), which leads into a channel within the maxilla, the canalis alveolaris superior. As shown by CT scans, the canalis alveolaris superior extends anteriorly, and branches to form a dense network within the maxilla, and that exits it via the numerous small foramina present on the triturating surface. This canal carries the superior alveolar artery, which supplies the maxilla with blood (Albrecht, 1976). As in *Chelonia mydas* (Albrecht, 1976), there is no posterior branch (canalis infraorbitalis) for the supramaxillary artery off of the canalis alveolaris superior in *R. pulchriceps*. The absence of the supramaxillary artery in *R. pulchriceps* is also supported by the absence of a foramen supramaxillare, which usually exits the canalis infraorbitalis posteriorly in the floor of the orbital fossa (Gaffney, 1979). The condition in *Dermochelys coriacea* has not been described (see Albrecht, 1976), but CT scans of a *Dermochelys coriacea* skull (FMNH 171756) show that a canalis infraorbitalis exists in this taxon and that the posterior exit, the foramen supramaxillare, is located between the jugal and maxilla. The foramen alveolare superius of *Dermochelys coriacea* is in the same position as in *R. pulchriceps*, but the former has an additional foramen posteriorly adjacent to the foramen alveolare superius, which also connects to the canalis alveolaris superior.

In *R. pulchriceps*, at a point halfway along the orbit, the dorsal surface of the maxillary jugal process bears a deep ventral incision for the jugal near its lateral margin, which can be seen in CT scans. The jugal enters this incision via a short anterior process, forming a strongly interlocking contact between the maxilla and jugal. In addition to this interlocking articulation, the posterior end of the maxillary jugal process bifurcates into laterodorsal and medioventral rami. The laterodorsal ramus articulates with a small groove on the lateral surface of the jugal anterior process. The medioventral ramus of the maxillary jugal process, which bears the posterior end of the labial ridge, extends ventral to the jugal and articulates with a small ventral groove.

### Vomer

The vomer is an unpaired midline bone situated in the anterior part of the palate (Figs. 3A, 3B, 5 and 10; Data S1: Figs. S1.2, S1.4, S1.6, S1.8, S1.10, S1.12 and S1.15). It contacts the premaxilla anteriorly and the maxilla anterolaterally in the floor of the nasal cavity via an anteroventral process that forms the medial margin of the internal naris. The main portion of the vomer is anteroposteriorly long, transversely narrow, and has a concave posterodorsal surface that forms the floor of the orbital fossa. It contacts the palatine posterolaterally, and produces paired dorsal processes that contact the prefrontals laterally. Together with the prefrontals, the vomer forms a vertical bony wall that separates the orbital fossa from the nasal cavity.

The anteroventral process is short and columnar and its anterior end is transversely broadened toward its contacts with the maxilla and premaxilla (Data S1: Fig. S1.15). The base of the anteroventral process is constricted between the anterior end of the process and the dorsal processes, forming the medial wall of each choanae. CT scans show that the contact surfaces for the premaxillae and maxillae are highly interdigitated. A low dorsomedian ridge on the anteroventral process of the vomer is continuous with a ridge on the premaxilla and likely served as an anchor for the narial septum. This ridge becomes shallower posteriorly and disappears just anterior to the dorsal processes.

The dorsal processes are gently inclined dorsolaterally and are situated in the anterior one-third of the element (Fig. 5; Data S1: Fig. S1.12). The right and left dorsal processes are separated from the midline by a deep fissure, the sulcus vomeri. Dorsally, the sulcus vomeri is continuous with the fissure ethmoidalis (see Prefrontal, above). The prefrontal articulates with the posterolateral side of the dorsal process. The contact surface of the vomer with the prefrontal is characterised by many finger-like extensions and deep pockets, indicating rigid interlocking between these elements.

The posterior process of the vomer forms a narrow triangular contribution to the palate that is wedged between the palatines. This part of the vomer thins dorsoventrally as it extends posteriorly. It also slopes gently posteroventrally toward its posterior margin, contributing to the dorsally concave form of the vomer that is best seen in lateral view. The lateral margins of the vomer that contact the palatines are also slightly curved dorsally, so that the dorsal surface of the vomer forms a shallow dorsally open trough that leads to the sulcus vomeri anteriorly. This trough disappears in the posterior half of the posterior process of the vomer and is replaced by a low, but sharp, median crest that likely served as the anchor for the interorbital septum.

The ventral surface of the vomer is transversely convex. Anteriorly, just before merging with the posterior surface of the anteroventral process, a median, well-rounded keel arises from the ventral surface of the posterior process. The keel separates the left and right internal nares and becomes very shallow posteriorly where the vomer tapers between the palatines.

### Palatine

The paired palatines are situated in the anterior part of the palate (Figs. 3A, 3B and 10; Data S1: Figs. S1.2, S1.4, S1.6, S1.8 and S1.10). Each palatine contacts the vomer anteromedially, the prefrontal anterolaterally, the pterygoid posteriorly, and the maxilla laterally via an anteroposteriorly broad and dorsoventrally deep lateral or maxillary process. The palatine forms the floor of the orbital fossa, the roof of most of the choanae, the posterior and lateral margins of the internal naris, and the posteromedial margin of the foramen orbito-nasale. The palatine also forms a clearly developed, albeit posterolaterally open, foramen palatinum posterius.

The palatine is horizontally oriented and plate-like. It is longer anteroposteriorly than it is wide transversely, narrows somewhat anterior to the lateral process and is transversely constricted at the level of the foramen palatinum posterius. Anteromedially, the palatine curves dorsally to contact the vomer and form a domed central part of the palate that roofs the choanae and separates the left and right orbital fossae. The palatines are sutured to each on the midline posterior to their contact with the vomer and form a horizontally flat surface with the posteriorly adjacent pterygoids.

Anteriorly, the palatine develops a narrow, spur-like process that contacts the vomer medially and articulates with the prefrontal anteriorly. The lateral margin of this spur forms the posteromedial border of the foramen orbito-nasale. The palatine contacts the maxilla in the posterolateral margin of this foramen. From here, the suture between the palatine and maxilla extends posterolaterally in a straight line that is visible on the ventral palatal surface and on the floor of the orbital fossa. The lateral margin of the palatine contacting the maxilla is expanded ventrally to form a dorsoventrally thick articular surface. This surface is deeply excavated and wraps around the posterior parts of the maxillary labial ridge. The anteromedial surface of the ventrally expanded articulation surface for the maxilla forms the lateral wall of the choanae. The palatine is pierced by a small foramen near the base of the maxillary process. This foramen is dorsoventrally directed, forming a small canal that connects the floor of the orbital fossa with the ventral surface of the palatine.

The posterior end of the palatine maxillary process forms a small tip that extends beyond the distal end of the maxillary labial ridge into the fenestra subtemporalis (Figs. 3A, 3B and 10). Posterior to the tip of the palatine maxillary process a well-rounded, medial notch is present in the lateral margin of the posterior part of the palatine. The notch is open posterolaterally and represents an open foramen palatinum posterius. This foramen is absent in modern cheloniids, *Dermochelys coriacea* (Gaffney, 1979) and probably the protostegid *Desmatochelys lowii* (KUVP 1200; see Raselli, 2018), but is present in other protostegids from the Early Cretaceous (e.g. Kear & Lee, 2006; Cadena & Parham, 2015). Posterior to the foramen palatinum posterius the palatine becomes slightly wider transversely, but it does not reach the width of the palatine anterior to the foramen. The palatine articulates with the pterygoid posteriorly. Thin bony sheets arising from the posterior margin of the palatine extend deeply into the anterior surface of the pterygoid, resulting in tight interlocking of these elements.

### Quadrate

The quadrate is a large bone in the posterolateroventral corner of the skull that forms the mandibular condyle and cavum tympani (Figs. 2, 3A and 3B; Data S1: Figs. S1.2, S1.4, S1.6, S1.8 and S1.10). It contacts the squamosal dorsolaterally and posteriorly, the quadratojugal anterolaterally, the opisthothic posteromedially and posteriorly, the prootic anterodorsolaterally and the pterygoid anteroventromedially. This description is based on several specimens with well-preserved quadrates, namely the left quadrate of CAMSM B55783 (Figs. 2A and 2B), the right quadrate of CAMSM B55775 (Data S1: Fig. S1.2B) and the right quadrate of CAMSM B55776 (Data S1: Fig. S1.8B). Variation between these specimens is noted throughout the text, but they agree in morphology unless stated otherwise.

The quadrate forms a vertical wall of bone that separates the middle ear into a lateral section that includes the cavum tympani and the incisura columella auris, and a medial part that constitutes the cavum acustico-jugulare. The dorsal portion of the quadrate borders the foramen stapedio-temporale laterally and contributes to the floor of the supratemporal fossa. The anterodorsal portion of the quadrate forms the lateral part of the processus trochlearis oticum (the medial part is formed by the prootic), which forms the posterodorsal border of the subtemporal fossa. Ventrally, the quadrate forms the articular process (=processus articularis of Gaffney, 1972), which bears the mandibular condyle for the articulation of the lower jaw. The medial surface of the quadrate borders the canalis stapedio-temporalis at the level of the prootic contact. Its dorsal foramen exits into the supratemporal fossa, while the medial foramen leads into the cavum acustico-jugulare. The medial surface of the quadrate further borders the posterior part of the canalis cavernosus, which extends anteriorly.

The cavum tympani is a large funnel-like cavity on the lateral surface of the quadrate. It is shallowest along the contact with the quadratojugal and squamosal, and deepens centrally. From this deep central portion, a posterodorsally oriented trough extends into a small recess of the squamosal, the antrum postoticum. This trough is bordered dorsally by a prominent, posteroventrally sloping shelf of the quadrate, and ventrally by a small process posterodorsal to the incisura columella auris. These structures do not contact each other in most specimens, and so the trough is ‘open’ laterally. The opening toward the antrum postoticum in the quadrate is referred to herein as the foramen antrum postoticum (Fig. 11B). In CAMSM B55775, however, the cavum tympani is closed posterodorsally so that the foramen antrum postoticum is absent. Nevertheless, the cavum tympani extends into a posterodorsally situated pocket-like recess, which excavates the body of the quadrate in a similar way to that seen in other specimens (Fig. 11A). Although the squamosal is missing in CAMSM B55775, the quadrate is well preserved and includes its dorsal suture for the squamosal. This indicates that the posterodorsal portion of the cavum tympanum is genuinely formed by the quadrate in this specimen rather than by a partially preserved fragment of the squamosal.

The posteroventral margin of the cavum tympani is embayed by a posteroventrally open notch, the incisura columella auris, through which the stapes passes (see Stapes, below). The incisura columella auris forms a short, open channel that connects the cavum tympani laterally to the cavum acustico-jugulare medially, just dorsal to the contact with the quadrate process of the pterygoid, and ventral to the level of the fenestra ovale, which is formed by the opisthotic and prootic.

The quadrate overlaps the lateral surface of the posterolateral surface of the quadratojugal via a simple, planar contact. The contact between these bones has an anteriorly convex outline in lateral view. The quadrate has a dorsally facing groove along the posterodorsal margin of the cavum tympanum, which is positioned posterior to the quadrate-quadratojugal contact. This groove receives the ventral margin of the squamosal. The groove separates the dorsal surface of the quadrate, which forms the floor of the supratemporal fossa, from the posterolateral margin of the quadrate, which borders the cavum tympanum. The dorsal surface of the quadrate slopes toward the prootic and opisthotic medially, so that it faces dorsomedially. The dorsal surface is anteroposteriorly elongate compared to its transverse width. It is widest centrally, rather than anteriorly or posteriorly, and its combined suture with the prootic and opisthotic is slightly convex medially in dorsal view.

A process bearing the quadrate portion of the processus trochlearis oticum extends anteriorly from the anterodorsal surface of the quadrate. The quadrate part of the processus trochlearis oticum curves medioventrally with respect to its prootic part, so that the process is braced ventrally by the quadrate. The anterior margin of the processus trochlearis oticum is convex and its dorsal surface is transversely concave. The relative contributions of the quadrate and prootic to the processus trochlearis oticum vary among specimens of *R. pulchriceps* (Fig. 12). In CAMSM B55783 (*R. cantabrigiensis sensu* Collins, 1970), CAMSM B55776 (*R. elegans sensu* Collins, 1970) and NHMUK PV OR35197 (*R. elegans sensu* Collins, 1970), most of the process is formed by the prootic, whereas the quadrate contributes just over half of the width of the process in CAMSM B55775 (*R. pulchriceps*, holotype).

The anterior surface of the quadrate is strongly concave transversely between the quadrate portion of the processus trochlearis oticum and the lateral sheet that articulates with the quadratojugal. Ventral to the processus trochlearis oticum, a thin epipterygoid process (=processus epipterygoidei of Gaffney, 1972) of the quadrate extends anteromedially to contact the lateral surface of the pterygoid (Fig. 8). The ventral margin of the epipterygoid process of the quadrate slots into a dorsally open groove on the anterolateral surface of the quadrate process of the pterygoid. The preserved extent of the epipterygoid process toward the trigeminal (CN V_2–3_) foramen is variable. Nevertheless, a small area on the lateral surface of the pterygoid ventral to the trigeminal foramen usually remains uncovered, exposing the fossa cartilaginis epipterygoidei, as is common in cryptodires (Gaffney, 1979). Posteromedial to the epipterygoid process, the quadrate and pterygoid are tightly sutured to each other.

The articular process of the quadrate extends ventrolaterally, projecting ventral to the level of the pterygoid. The ventral surface of this process forms the mandibular condyle (=condylus mandibularis of Gaffney, 1972). The mandibular condyle is broad transversely and has a centrally placed, anteroposteriorly oriented sulcus. The mandibular condyle is constricted anterior and posterior to this sulcus, dividing it into medial and lateral convex facets.

The dorsal part of the medial surface of the quadrate contacts the opisthotic posteriorly and the prootic anteriorly. The opisthotic appears to be loosely connected to the quadrate, as there are no interdigitations between these bones. However, the paroccipital process (=processus paroccipitalis of Gaffney, 1972) of the opisthotic wraps ventrally around the posteromedial surface of the quadrate. The canalis stapedio-temporalis extends vertically between the quadrate and prootic near the posterior margin of their contact, connecting the supratemporal fossa dorsally with the cavum acustico-jugulare ventrally through the aditus canalis stapedio-temporalis. The cavum acustico-jugulare is a broad cavity between the quadrate, prootic, opisthotic, and pterygoid (see Cavum acustico-jugulare, below).

### Epipterygoid

Among turtles, the epipterygoid is ossified only in cryptodires and various stem-group turtles. With the exception of *Proganochelys quenstedtii* (Gaffney, 1990), the epipterygoid is usually discernible in adults as a small plate of bone situated on the lateral side of the crista pterygoidea of the pterygoid, anterior to the trigeminal (CN V_2–3_) foramen. None of the CT-scanned specimens of *R. pulchriceps* possesses an ossified epipterygoid. Nevertheless, a fossa cartilaginis epipterygoidei, which receives the epipterygoid, is present on the lateral surface of the pterygoid ventral to the trigeminal foramen, indicating the potential presence of a cartilaginous element. Based on CT scans, we can also confirm the absence of an ossified epipterygoid in *Bouliachelys suteri* (QM F31669) and *Notochelone costata* (NHMUK PV R9590). Raselli (2018) recently described an epipterygoid for *Desmatochelys lowii*. However, the morphology and position of this bone are unusual, in that it is very small, limited to the anterior margin of the secondary lateral wall of the braincase and does not extend further posteriorly. Examination of the same CT scan of KUVP 1200 used by Raselli (2018) could not unambiguously confirm the presence of an epipterygoid for *Desmatochelys lowii* and we think that the reported ‘epipterygoid’ is more likely to represent part of the processus inferior parietalis.

### Pterygoid

The pterygoids form the posterior part of the palate and connect with the braincase and skull roof (Figs. 3A, 3B, 10 and 13; Data S1: Figs. S1.2, S1.4, S1.6, S1.8, S1.10 and S1.16). They are paired, contacting each other anteromedially, and each also contacts the parabasisphenoid posteromedially, the quadrate posterolaterally, the basioccipital posteriorly, the prootic posterodorsally, the parietal dorsally, and the palatine anteriorly. As in other Early Cretaceous protostegids (e.g. Hirayama, 1998; Kear & Lee, 2006; Cadena & Parham, 2015) and *Dermochelys coriacea*, but unlike in cheloniids (e.g. *Eretmochelys imbricata* FMNH 22242; *Lepidochelys olivacea* SMNS 11070), there is no contact between the pterygoid and the jugal in *R. pulchriceps*. The pterygoid floors the canalis cavernosus, sulcus cavernosus, and cavum acustico-jugulare, encloses large parts of the trigeminal (CN V_2–3_) foramen and parts of the internal carotid arterial system, and forms the ventral border of the fenestra postotica.

The palatal portion of the pterygoid consists of a transversely narrow and anteroposteriorly elongate horizontal plate that is constricted around its midlength so that it has concave lateral margins when seen in ventral view (Figs. 13A and 13B). The anterior halves of the left and right pterygoids contact each other on the midline (Figs. 13A–13D). The anterior part of the pterygoid forms a dorsoventrally narrow sheet that expands laterally at its anterior end to form the external pterygoid process (=processus pterygoideus externus of Gaffney, 1972). The external pterygoid process is slightly thickened anterodorsally at its lateral tip and projects laterally into the subtemporal fenestra. As the foramen palatinum posterius of the palatine is open, and there is no pterygoid contact with the maxilla and/or jugal, the foramen palatinum posterius and the subtemporal fenestra form a large, continuous opening between the palate medially and zygatomal arch laterally, which is roughly kidney-shaped in outline (Figs. 3A and 3B). The anterior part of the pterygoid overlaps the palatine for a short distance.

In its posterior third, the palatal portion of the pterygoid forms the posteroventrolaterally directed quadrate process. This process is sutured tightly to the medial surface of the articular process of the quadrate. The posterior halves of the pterygoids diverge to form a broad triangular opening for the parabasisphenoid (Figs. 13C and 13D). The ventral surface of the pterygoid lateral to the parabasisphenoid bears the pterygoid fossa, which forms a shallow, anteromedially oriented groove. The medial side of this groove is bounded by a low, anteromedially oriented ridge, immediately adjacent to the parabasisphenoid (see Evers & Benson, 2019). This ridge turns posterolaterally at the level of the foramen posterius canalis carotici interni (fpcci).

The foramen posterius canalis carotici interni (*sensu* Rabi et al., 2013) is the entrance for the internal carotid artery and enters the skull between the parabasisphenoid and pterygoid in most specimens. In one specimen (CAMSM B55776: *R*. *elegans sensu* Collins, 1970), the foramen is enclosed completely within the pterygoid, albeit immediately adjacent to its contact with the parabasisphenoid. In all other CT-scanned specimens in which this feature can be observed (CAMSM B55783, CAMSM B55775, NHMUK PV OR35197, NHMUK PV OR43980), the parabasisphenoid forms the medial border of the foramen (Figs. 13C and 13D). The canalis caroticus internus extends anteriorly from this foramen and is contained within the internal contact surfaces of pterygoid and parabasisphenoid in all specimens, including CAMSM B55776 (Data S1: Fig. S1.16). The course of the internal carotid artery, as well as its bifurcation into the cerebral and palatine arteries, is entirely enclosed by bone. This bifurcation occurs at the level of the dorsum sellae of the parabasisphenoid. The canalis caroticus cerebralis for the cerebral artery diverges anteromedially, and is completely contained within the parabasisphenoid (for additional details of the cerebral artery see Parabasisphenoid). The canalis caroticus palatinum for the palatine artery is enclosed primarily by the pterygoid, with the parabasisphenoid enclosing only its medial surface. The canalis caroticus palatinum extends anteriorly and slightly dorsally from the bifurcation point of the internal carotid artery. It is positioned ventral to the sulcus cavernosus and is separated from it by a medial, sheet-like extension of the pterygoid. This horizontal sheet of bone contacts the lateral side of the rod-like anterior process of the parabasisphenoid, the rostrum basisphenoidale. The anterior exit of the canalis caroticus palatinum, the foramen anterius canalis carotici palatinum (faccp), is located approximately halfway along the rostrum basisphenoidale, ventrally below the level of the anterior end of the sulcus cavernosus (Figs. 13A, 13B, 13E and 13F). One specimen, NHMUK PV OR35197 (*R*. *elegans sensu* Collins, 1970), has an additional foramen for a short ventromedial branch of the palatine artery. This additional foramen exits on the lateral surface of the pterygoid into the subtemporal fossa.

The posterior process of the pterygoid extends beyond the posterior margin of the parabasisphenoid to contact the basioccipital. The dorsal surface of this posterior process is mediolaterally concave and wraps around the anterior part of the ventrolateral surface of the basal tuber of the basioccipital.

On the dorsal surface of the anterior end of the pterygoids the interpterygoid suture is raised to a low medial ridge. The ridge becomes broader posteriorly and serves as a buttress for the rod-like rostrum basisphenoidale of the parabasisphenoid (Figs. 13E and 13F). Lateral to this median ridge, the dorsal surface of the pterygoid is incised by a narrow, deep, anteroposteriorly oriented trough. This trough marks the course of the palatine artery anterior to the foramen anterius canalis carotici palatinum. The posterior part of the course of the palatine artery is covered dorsally by a medial extension of the crista pterygoidea, which is a vertical sheet of bone that ascends along the lateral margin of the pterygoid.

The medial surface of the crista pterygoidea bears a thin horizontal shelf of bone that projects medially and contacts the rostrum basisphenoidale of the parabasisphenoid. This shelf covers the trough for the palatine artery posterior to the foramen canalis carotici palatinum. The medial shelf of the crista pterygoidea also forms the floor of the sulcus cavernosus. The sulcus cavernosus is a dorsally open trough that carries the vena capitis lateralis (Gaffney, 1972). The sulcus cavernosus extends parallel to the rostrum basisphenoidale of the parabasisphenoid anteriorly and curves posterolaterally along the anteromedial margin of the parabasisphenoid more posteriorly. The sulcus cavernous is covered by the prootic dorsally in its posterior third and is termed the canalis cavernous in this region. The opening between the canalis cavernous and sulcus cavernosus is the foramen cavernosus. In *R. pulchriceps*, the foramen cavernosus is positioned at the level of the posterior margin of the trigeminal foramen. Just anterior to the foramen cavernosus, a short canal extends from the floor of the sulcus cavernosus ventromedially between the pterygoid and parabasisphenoid and connects to the canalis caroticus internus. This canal is the passage for the vidian (or palatine) ramus of the facial (CN VII) nerve (foramen pro ramo nervi vidiani of Gaffney, 1972) and leads from the sulcus cavernous into the canalis caroticus internus, where it continues anteriorly (Gaffney, 1979). This small canal is best preserved in NHMUK PV OR35197. In the posterior part of the pterygoid, the course of the canalis cavernosus is marked by a low ridge on the dorsal surface of the pterygoid (Figs. 13A and 13B). This ridge contacts the lateral margin of the ventromedially directed process of the prootic. The canalis cavernosus opens posteriorly into the cavum acustico-jugulare, but the ridge on the dorsal surface of the pterygoid delimits the course of the vena capitis lateralis posteriorly up to the fenestra postotica.

The crista pterygoidea forms an anteroposteriorly long contact with the processus inferior parietalis of the parietal (see also Parietal, above). Together, these structures form the secondary lateral wall of the braincase (Fig. 8). Right and left cristae pterygoideae diverge posterolaterally from the midline. The crista pterygoidea becomes dorsally higher posteriorly. Just anterior to the contact with the quadrate, the crista pterygoidea bifurcates dorsally into two prongs, which form the anterior and posterior margins of the trigeminal (CN V_2–3_) foramen. The trigeminal foramen is a dorsoventrally high, oval opening, with a concave ventral margin that is also formed by the crista pterygoidea. The dorsal margin of the trigeminal foramen is shared between the prootic posteriorly and the parietal anteriorly.

The lateral surface of the crista pterygoidea of the pterygoid is marked by a small groove, which is posterolateroventrally directed from the ventral margin of the trigeminal foramen and continues onto the anterior surface of the quadrate process of the pterygoid. This incision receives the epipterygoid process of the quadrate, which is ossified variably. The anterior end of the groove, directly posteroventral to the trigeminal foramen, is not usually occupied by the processus epipterygoidei and is termed the fossa cartilaginis epipterygoidei. It possibly indicates the presence of an unossified epipterygoid in *R. pulchriceps* (see also Epipterygoid, above).

### Supraoccipital

The supraoccipital is an unpaired median bone in the posterodorsal corner of the skull (Figs. 4C and 4D; Data S1: Figs. S1.2, S1.4, S1.6, S1.8 and S1.10). It forms the roof of the posterior part of the endocranial cavity and contacts the parietal anterodorsolaterally, the prootic anteroventrolaterally, the opisthotic lateroventrally and the exoccipital posteroventrolaterally. The supraoccipital forms the dorsal margin of the foramen magnum and houses parts of the endosseous labyrinth.

In transverse cross-section, the posterior part of the supraoccipital has an inverted ‘Y’-shaped outline, as it consists of a dorsally placed, vertically oriented plate from which ventrolateral processes diverge to either side. The vertical plate is wedged between the posterior processes of the parietals, but extends posteriorly beyond them and dorsal to the foramen magnum as a short crista supraoccipitalis. The exact posterior extent of the crista supraoccipitalis is unknown, as it is damaged in all *R. pulchriceps* skulls. The ventrolateral processes of the supraoccipital are dorsoventrally short at the level of the foramen magnum, but become progressively taller dorsoventrally anteriorly. The vertical sheet of the supraoccipital disappears anteriorly as the ventrolateral processes become dorsoventrally taller. As a consequence, the anterior part of the supraoccipital has a simple arch-shaped transverse cross-section and the endocranial cavity is dorsally expanded anteriorly along the length of the supraoccipital. Anteriorly, the parietal overlaps the dorsolaterally facing surface of the ventrolateral process of the supraoccipital, forming a weakly interdigitating contact.

The prootic and opisthotic facets of the supraoccipital are mediolaterally expanded with respect to the dorsal part of the ventrolateral processes. These facets face almost ventrally, but are inclined slightly anteroventrally (prootic facet) and posteroventrally (opisthotic facet) so that the ventral portion of the supraoccipital appears gently kinked in lateral view. The contacts between the three bones forming the otic capsule (prootic, opisthotic, and supraoccipital) are not tightly closed in any of the specimens that were CT scanned. Instead, the sutures are widely open, suggesting that these specimens might represent ontogenetically immature individuals. However, this region of the skull is the least ossified among turtles more widely (SWE, personal observation), so the implications of this observation for ontogeny are unclear.

The prootic and opisthotic facets of the supraoccipital are penetrated by foramina for the anterior semicircular canal (prootic facet) and the posterior semicircular canal (opisthotic facet). These canals meet within the supraoccipital at the level of the prootic-opisthotic contact, forming the common crus. The common crus extends ventrally through the supraoccipital into the space lateral to the hiatus acusticus. In some specimens (CAMSM B55783, NHMUK PV OR35197), the ossification of the ventral surface of the ventrolateral process of the supraoccipital is incomplete, so that the portions of the anterior and posterior semicircular canal that lie within the supraoccipital join via a single, ventrally open and medially convex trough. In one specimen (CAMSM B55775) the foramen aquaducti vestibuli is fully ossified, while it is not ossified or only partially ossified in others (e.g. NHMUK PV OR35197). This foramen is located in the medioventral wall of the supraoccipital, ventral to the common crus, and transmits the endolymphatic duct (Gaffney, 1979).

### Exoccipital

The exoccipitals are paired bones situated in the posterior portion of the braincase (Figs. 4C and 4D; Data S1: Figs. S1.2, S1.4, S1.6, S1.8 and S1.10). They form the lateral margins of the foramen magnum and contact the supraoccipital dorsally and the basioccipital ventrally. Anterolaterally, each exoccipital is sutured to the opisthotic. A marginal point contact to the pterygoid also seems to be present. The exoccipitals contribute to the formation of the occipital condyle (=condylus occipitalis of Gaffney, 1972) just lateral to the basioccipital. The exoccipital also forms the medial margin of the fenestra postotica, borders the recessus scalae tympani posteriorly, closes the anterior jugular foramen (=foramen jugulare anterius of Gaffney, 1972) posteriorly, and houses the foramina and associated canals for the rami of the hypoglossal (CV XII) nerve.

The exoccipital consists of an anterolaterally broad base from which a centrally placed, rod-like dorsal process emerges that forms the lateral wall of the foramen magnum. The base of the exoccipital contacts the basioccipital over its entire anteroposterior length via a ventromedially directed articular surface. Posteriorly, the base of the exoccipital merges into a short, rounded process that forms the dorsolateral portion of the occipital condyle. The processes of the right and left exoccipital approach each other dorsomedially, but they are separated on the midline by the posterior portion of the basioccipital. Anteriorly, the base of the exoccipital broadens dorsoventrally and extends anteroventrolaterally into the floor of the cavum acustico-jugulare and onto the dorsal surface of the basal tuber of the basioccipital. The posterior process of the pterygoid articulates with the basal tuber of the basioccipital. However, this contact is slightly disarticulated in those specimens for which we have CT scans. This makes it difficult to assess if a pterygoid-exoccipital contact was present in *R. pulchriceps*, which is the case in *Plesiochelys etalloni* (Anquetin, Püntener & Billon-Bruyat, 2015) and cheloniids (Gaffney, 1979). It also unclear if the exoccipitals and pterygoids are in contact in *Bouliachelys suteri*, but a contact is evident in *Notochelone costata* (NHMUK PV OR37213). In *Dermochelys coriacea* (UMZC R3031) a pterygoid-exoccipital contact is absent.

The dorsal process of the exoccipital curves dorsomedially around the lateral margin of the foramen magnum, which has a dorsoventrally high, oval outline. The posterior surface of the dorsal process bears a low ridge that extends ventrolaterally from the supraoccipital contact. This ridge merges with the surface of the exoccipital just dorsal to the occipital condyle. The prominence of this ridge varies among specimens: it is strongly developed in CAMSM B55775 (*R. pulchriceps* holotype), but absent in CAMSM B55783 (*R. cantabrigiensis sensu* Collins, 1970) (Fig. 14). It is likely that this ridge is an attachment site for axial musculature, and differences in its development might represent intraspecific variation (see Discussion, below).

The lateral portion of the exoccipital has complicated geometry with several surfaces of different orientation. The exoccipital underlaps the opisthotic forming a transversely broad, concave contact surface that buttresses the posteroventromedial surface of the opisthotic. A concave ridge on the ventrolateral surface of the process contacting the opisthotic forms the dorsomedial margin of the fenestra postotica. This ridge separates the ventrolateral surface of the exoccipital into a posterior portion and an anterior portion. The fenestra postotica is transversely wide in *Rhinochelys* and incorporates the passage of the vena cerebralis posterior, contrasting with the condition present in many cryptodires, including some sea turtles such as *Allopleuron hofmanni* (NHMUK PV R4213), in which this vein is housed in a separate posterior jugular foramen (=foramen jugulare posterius of Gaffney, 1972, 1979). The absence of an ossified boundary between the fenestra postotica and posterior jugular foramen is, however, common in turtles and occurs in most chelonioids (Gaffney, 1979; Evers & Benson, 2019, 2018).

The portion of the ventrolateral surface of the exoccipital posterior to the margin of the fenestra postotica extends from the medial margin of the fenestra postotica to the exoccipital part of the occipital condyle posteriorly, and the posterior surface of the process that contacts the basal tuber of the basioccipital. This surface is roughly triangular, faces posteroventrolaterally, and is excavated to form a shallow fossa. The fossa is penetrated by two circular foramina for the hypoglossal (XII) nerve (the foramina nervi hypoglossi). The posterior foramen is oval in outline and approximately three times the diameter of the anterior foramen, which has a circular outline. In turtles, the hypoglossal nerve frequently exits the skull through three openings (Gaffney, 1979), so that it seems likely that the posterior foramen nervi hypoglossi transmits two rami of the hypoglossal nerve. The nerve canals are anterodorsomedially directed and connected to the endocranial cavity by two internal foramina near the base of the exoccipital.

The portion of the ventrolateral surface anterior to the margin of the fenestra postotica lies within the cavum acustico-jugulare. The recessus scalae tympani is a medial extension of the cavum acustico-jugulare (see Cavum acustico-jugulare, below). The anterior surface of the dorsal process of the exoccipital forms the posterior wall of the recessus scalae tympani, which is anteriorly bordered by the opisthotic. The recessus scalae tympani connects the cavum acustico-jugulare with the endocranial cavity via the anterior jugular foramen (Figs. 15C and 15D). This foramen is formed by the exoccipital posteriorly and forms the passage for the vagus (CN X) and accessory (CN XI) nerves and the vena cerebralis posterior (Gaffney, 1972). The anterior surface of the exoccipital does not quite contact the processus interfenestralis of the opisthotic, which forms the anterior margin of the anterior jugular foramen and the anterior wall of the recessus scalae tympani (see Opisthotic, below). The resulting gap between these bones was presumably covered by cartilage. The fenestra perilymphatica, an opening between the cavum labyrinthicum and the recessus scalae tympani, is bordered ventrally by the exoccipital but lies mostly within the processus interfenestralis of the opisthotic (see Opisthotic, below).

### Basioccipital

The basioccipital is an unpaired, median bone in the posteroventral region of the braincase (Figs. 3A, 3B, 15C and 15D; Data S1: Figs. S1.2, S1.4, S1.6, S1.8 and S1.10). It contacts the parabasisphenoid anteriorly, the pterygoid anterolaterally, and the exoccipital dorsolaterally. The basioccipital forms the posterior floor of the endocranial cavity, the ventral margin of the foramen magnum, the posteroventral portion of the occipital condyle and the basal tubera.

The basioccipital is horizontally aligned with the parabasisphenoid and broadens anteriorly so that is has a triangular outline in ventral view (Figs. 3A and 3B). The anterior surface of the basioccipital is flat and faces the parabasisphenoid. In all CT-scanned specimens of *R. pulchriceps* with preserved basioccipitals, there is a small vertical space between these bones. The posterior part of the basioccipital is mediolaterally broad ventrally and mediolaterally constricted dorsally between the posterior portions of the exoccipitals. The basioccipital and exoccipitals jointly form the occipital condyle.

The dorsal surface of the basioccipital forms the floor of the endocranial cavity and slopes gently anteroventrally from the foramen magnum towards the parabasisphenoid. This surface has a narrow triangular outline that tapers posteriorly towards the basioccipital portion of the occipital condyle. The lateral margins of this surface are elevated to form shallow, sharp-edged ridges, so that the dorsal surface is gently transversely concave. In NHMUK PV OR35197, a low parasagittal, median crest is present on the dorsal surface of the basioccipital. This is the crista dorsalis basioccipitalis, which separates the dorsal surface of the basioccipital into two transversely concave regions either side of the midline. The crista dorsalis basioccipitalis is not present in CAMSM B55783 or CAMSM B55775. Another median structure on the dorsal surface of the basioccipital is the basis tuberculi basalis. This is a low, mound-like tubercle at the anterior end of the crista dorsalis basioccipitalis (Figs. 15C and 15D). In all specimens preserving a basioccipital, the basis tuberculi basalis is positioned at the anterodorsal margin of the basioccipital and projects slightly anteriorly into the otherwise vertically flat anterior surface of the basioccipital. The posterodorsal edge of the parabasisphenoid has a small notch for this tubercle, which is most clearly developed in NHMUK PV OR35197, but is present in all specimens examined.

The posterolateral surfaces of the basioccipital are sutured to the exoccipitals, which become slightly broader anteriorly than posteriorly. The basal tubera emerge ventrolaterally from the basioccipital and are positioned slightly anterior to its midlength. The basal tubera are anteroposteriorly longer than they are dorsoventrally deep and have rounded posteroventral surfaces. The anterior surfaces of the basal tubera contact the posterior processes of the pterygoids. The anteroventrolateral bases of the tubera are also covered ventrally by short extensions from the posterior processes of the pterygoids. The ventral surface of the basioccipital between the basal tubera is gently concave transversely and smooth.

### Prootic

The prootic forms the anterior part of the otic capsule (Fig. 15A–15D; Data S1: Fig. S1.17). It contacts the parietal anteromediodorsally, the pterygoid anteroventrally, the quadrate laterally, the opisthotic posteriorly, the supraoccipital posterodorsomedially, and the parabasisphenoid ventromedially. The prootic forms the anterior part of the cavum labyrinthicum and fenestra ovale and contributes to the cavum acustico-jugulare, the canalis stapedio-temporale, the canalis cavernosus, and foramen cavernosum, the trigeminal (CN V_2–3_) foramen, and the processus trochlearis oticum. It further forms the foramina nervi facialis (CN VII) and the foramina nervi acustici (CN VIII).

The prootic has its most extensive contact with the quadrate, which it abuts along its entire lateral surface. The prootic and quadrate are sutured to each other and the externally visible suture line in the floor of the supratemporal fossa is laterally concave. The dorsomedial margin of the prootic, which contacts the parietal anteriorly and the opisthotic posteriorly, is roughly convex in dorsal view. As a result, the prootic has a roughly semilunate shape in dorsal view.

Together, the prootic and quadrate form the vertically directed canalis stapedio-temporale, which exits dorsally into the supratemporal fossa via the foramen stapedio-temporale and exits ventrally into the cavum acustico-jugulare via the aditus canalis stapedio-temporalis. The foramen stapedio-temporale is large in *R. pulchriceps* and exceeds the diameter of the foramina associated with the carotid circulation. Anteromedially, the quadrate and prootic form the processus trochlearis oticum (see Quadrate, above, with respect to the variation seen in this feature). The prootic part of the processus trochlearis oticum protrudes anterolaterally and is separated from the anteromedial corner of the prootic by a concave notch. The anteromedial corner of the prootic forms a short, anteriorly projecting, pointed process. This process forms the posterodorsal border of the trigeminal (CN V_2–3_) foramen. The anterior surface of the prootic is covered largely by the epipterygoid process of the quadrate and, more medially, the posterior ramus of the crista pterygoidea of the pterygoid. The latter excludes the prootic from the posterior margin of the trigeminal foramen. The anteromediodorsal margin of the prootic contacts the ventral surface of the processus inferior parietalis and has a weakly developed, parasagittally oriented incision for its articulation.

The prootic has a prominent ventromedial process (Figs. 15C and 15D). The process is broad and has a subtriangular cross-section. It terminates dorsally above the floor of the pterygoid, and nearly contacts the laterodorsal surface of the parabasisphenoid. In all CT scans, the ventral surface of the ventromedial process of the prootic approaches the parabasisphenoid very closely, but they are never sutured to one another. The small gap between the prootic and parabasisphenoid was likely closed by cartilage during life. The ventromedial process of the prootic forms a bony bridge over the trough in the pterygoid for the course of the lateral head vein, thereby forming the roof of the canalis cavernosus. The resulting foramen between the ventromedial process of the prootic medially, and the pterygoid laterally, is the foramen cavernosum. The foramen cavernosum is located directly posterior to the level of the trigeminal (CN V_2–3_) foramen (Figs. 15C and 15D).

The medial surface of the ventromedial process of the prootic is penetrated by a deep fossa acustico-facialis (Figs. 15C and 15D). Several canals extend laterally from the fossa acustico-facialis. The facial nerve (CV VII) has a short, anterolaterally directed canal that connects the endocranial cavity with the canalis cavernosus. The medial foramen nervi facialis is a small oval opening located in the anterior corner of the fossa acustico-facialis (Data S1: Figs. S1.17H and S1.17K). The lateral foramen that exits into the canalis cavernosus is of similar size and shape. This is indicative of a facial nerve innervation like that of cryptodires, in which the facial nerve forms the geniculate ganglion in the canalis cavernosus, which gives off an anteriorly directed vidian (or palatine) branch, and a posteriorly directed hyomandibular branch (Soliman, 1964; Gaffney, 1979). The fossa acustico-facialis is penetrated by two further foramina for the acoustic nerve (CN VIII). These foramina are located in the posterodorsal corner of the fossa acustico-facialis and connect the endocranial cavity with the prootic recess for the cavum labyrinthicum. Two distinct foramina nervi acustici can be distinguished in CAMSM B55775 (Data S1: Figs. S1.17C and S1.17F) and CAMSM B55776. In those specimens, the more anteriorly positioned foramen is about three times larger than the other foramen. In the other specimens that were CT scanned, the thin bony bar separating these foramina is not preserved, so that both foramina nervi acustici share a single, large opening toward the cavum labyrinthicum.

The posteromedial margin of the prootic has a deep, posteriorly concave notch positioned just dorsal to the ventromedial process. This notch forms the anterior margin of the hiatus acusticus, which is a large opening between the endocranial cavity medially and the cavum labyrinthicum laterally, and between the prootic anteriorly and the opisthotic posteriorly.

The prootic is invaded by a large cavity that makes up the prootic part of the cavum labyrinthicum (recessus labyrinthicus prooticus of Gaffney, 1979). The cavum labyrinthicum is anteroventrally bordered by the ventromedial process of the prootic and extends anteriorly deep into the body of the prootic. Laterally, the cavum labyrinthicum is closed by an extensive wall of bone, which laterally abuts the quadrate. The anterior semicircular canal projects through the dorsal part of the body of the prootic and connects to the large chamber of the cavum labyrinthicum in its anterodorsal corner. The canal then curves posterodorsomedially and opens in the posterodorsomedial surface of the prootic. This surface faces the supraoccipital. In all *Rhinochelys* specimens there is a gap between this surface and the supraoccipital, suggesting that the contact of these bones was covered by cartilage. An imprint of the lateral semicircular canal can be seen on the medial surface of the lateral wall of the cavum labyrinthicum. The lateral canal is bordered medially by bone only on the opisthotic side, whereas the prootic part is medially confluent with the large cavity for the cavum labyrinthicum (Figs. 15C and 15D). The prootic and opisthotic contact each other on via the posterolateral surface of the prootic, and both surround the lateral semicircular canal dorsally, ventrally, and laterally. The contact surfaces between the prootic and opisthotic are planar, with only a minor gap between them in comparison with to the widely open contact to the supraoccipital.

The ventral side of the lateral wall of the cavum labyrinthicum connects with the posteromedioventral side of the medioventral process of the prootic, forming a concave margin between both structures. This concave margin forms the anterodorsal border of the fenestra ovale, which is the lateral opening of the cavum labyrinthicum toward the cavum acustico-jugulare. The fenestra ovale serves as the attachment for the stapedial footplate of the stapes. The fenestra ovale is completed posteriorly by the opisthotic, but ventrally it has no bony border and opens above the level of the floor of the cavum acustico-jugulare, which is formed by the pterygoid.

### Opisthotic

The opisthotic forms the posterior half of the otic capsule (Figs. 4C, 4D and 15A–15D; Data S1: Figs. S1.2, S1.4, S1.6, S1.8, S1.10 and S1.18). It contacts the prootic anteriorly, the supraoccipital dorsomedially, the exoccipital posterodorsomedially, the quadrate laterally, and the squamosal posterodorsolaterally. The opisthotic forms the posterior floor of the supratemporal fossa, the posterior part of the roof of the cavum acustico-jugulare, the posterior margin of the fenestra ovale, the posterior part of the cavum labyrinthicum, the anterior wall of the recessus scalae tympani, contributes to the anterior jugular foramen (=foramen jugulare anterius of Gaffney, 1972) and forms the fenestra perilymphatica, as well as the canal for the glossopharyngeal nerve (CN IX). Two important processes emerge from the body of the opisthotic, namely the processus interfenestralis anteroventrally, and the paroccipital process (=processus paroccipitalis of Gaffney, 1972) lateroventrally.

The lateral surface of the opisthotic is buttressed against the quadrate and is also confluent with the dorsolateral surface of the paroccipital process. The latter forms a relatively thin posteroventrolaterally directed extension of the opisthotic. The paroccipital process wraps around the posteroventromedial surface of the quadrate ventrally and terminates just before reaching the incisura columella auris of the quadrate. The large contact surface between the opisthotic and quadrate is smooth: these bones are not sutured but merely abut each other.

The dorsal surface of the body of the opisthotic forms the floor of the supratemporal fossa. This surface is roughly triangular and narrows anteriorly toward the prootic contact. The squamosal has a very small contact with the posterodorsolateral corner of the opisthotic, which is preserved only in CAMSM B55783. The facet for this contact is preserved in other specimens also, such as CAMSM B55775 (Data S1: Figs. S1.18C and S1.18F). On the posteromedial side, the opisthotic has an extensive contact surface for the exoccipital and these bones are sutured to each other.

The anterior margin of the opisthotic toward the prootic and the dorsomedial margin that faces the supraoccipital are angled at approximately 120° to each other, resulting in a roughly ‘Y’-shaped suture between the prootic, opisthotic, and supraoccipital. As mentioned previously (see Supraoccipital and Prootic, above), all of the bones in the otic capsule are widely spaced, with cartilage presumably filling in the voids between them.

The anterior surface of the opisthotic that contacts the prootic is penetrated by the canal for the lateral semicircular canal. By contrast with the prootic, the opisthotic completely encloses the lateral semicircular canal on all sides, so that it is a true canal rather than a medially open trough. The lateral semicircular canal extends posteromedially into the body of the opisthotic and opens internally into a large cavity that forms the opisthotic portion of the cavum labyrinthicum (recessus labyrinthicus opisthoticus of Gaffney, 1979). The cavum labyrinthicum extends deep into the body of the opisthotic posterolaterally and is connected to another canal in its dorsolateral corner. The latter is the posterior semicircular canal, which extends dorsomedially in a slightly arched trajectory above the opisthotic portion of the cavum labyrinthicum and exits the opisthotic via its articular surface for the supraoccipital. The posterior semicircular canal continues into the supraoccipital, where its meets the anterior semicircular canal in the common crus (see Supraoccipital, above).

The main body of the opisthotic bears a ventrally and slightly anteromedially directed process termed the processus interfenestralis (Figs. 15C and 15D; Data S1: Fig. S1.18). The processus interfenestralis projects into the otherwise continuous, unossified space between the ventral part of the cavum labyrinthicum and the cavum acustico-jugulare, and thus forms a landmark separating these two cavities. The processus does not contact the ventral floor of the skull, so a gap remains between the ventral margin of the processus interfenestralis and the area around the pterygoid-basioccipital contact. The processus interfenestralis also separates the fenestra ovale and fenestra perilymphatica. The former is situated anterolaterally with respect to the latter and the margins of the processus interfenestralis are oriented accordingly; the margin toward the fenestra ovale faces anterolaterally, and is markedly concave, and the margin toward the fenestra perilymphatica faces posterolaterally and slightly ventrally. The fenestra perilymphatica is not covered by bone posteroventromedially, so that the fenestra forms a deep, but open, semicircular notch in the posteromedial margin of the processus interfenestralis. The fenestra perilymphatica opens posteroventrally from the cavum labyrinthicum into the recessus scalae tympani. The recessus scalae tympani is a transversely oriented cavity between the opisthotic and exoccipital and is the medial portion of the cavum acustico-jugulare (see Cavum acustico-jugulare, below). It is laterally open toward, and thus continuous with, the more anteroposteriorly oriented cranioquadrate space portion of the cavum acustico-jugulare (see Gaffney, 1979).

A short, posteroventrally directed canal for the glossopharyngeal nerve (CN IX) pierces the floor of the cavum labyrinthicum, near the base of the processus interfenestralis, and exits into the recessus scalae tympani. The respective foramen on the internal surface of the opisthotic facing the cavum labyrinthicum is called the foramen internum nervi glossopharyngei (Figs. 15C and 15D), whereas the foramen in the surface facing the recessus scalae tympani is the foramen externum nervi glossopharyngei. An additional foramen (foramen medialis nervi glossopharyngei of Gaffney, 1979) for the exit of the glossopharyngeal nerve from the endocranial cavity remains usually unossified in the hiatus acusticus, although it is evident in CT scans of CAMSM B55775 (Data S1: Figs. S1.18A, S1.18D, S1.18H and S1.18K). In this specimen, the foramen medialis nervi glossopharyngei is located at the posteromedial margin of the processus interfenestralis and opens anterolaterally into the cavum labyrinthicum.

The posteroventral side of the opisthotic is a relatively thin wall of bone between the cavum labyrinthicum and recessus scalae tympani. The posterior wall of the recessus scalae tympani is formed by the exoccipital, ventral to its sutural contact with the opisthotic. The medial margin of the opisthotic and the anteromediodorsal margin of the exoccipital form an elongate anterior jugular foramen, which forms the medial opening of the recessus scalae tympani toward the endocranial cavity. The medial connexion between the recessus scalae tympani and the cranioquadrate space portion of the cavum acustico-jugulare is positioned in the posterior-most third of the latter, posterior to the position of the processus interfenestralis and just anterior to the fenestra postotica.

### Parabasisphenoid

The parabasisphenoid is an unpaired median bone in the centre of the basicranium (Figs. 3A, 3B, 13, 15A and 15B; Data S1: Figs. S1.2, S1.4, S1.6, S1.8, S1.10, S1.19 and S1.20). It forms the anterior floor of the endocranial cavity and contacts the pterygoid laterally, the prootic posterolaterally, and the basioccipital posteriorly. Several important nervous and vascular structures traverse the parabasisphenoid, including the canals for the abducens (CN VI) nerve, the canalis caroticus cerebralis, as well as parts of the canalis caroticus internus and canalis caroticus palatinum. Other important structures formed by the parabasisphenoid include the clinoid process (=processus clinoideus of Gaffney, 1972), the dorsum sellae, and the rostrum basisphenoidale.

The parabasisphenoid consists of a cup-shaped portion posteriorly and extends anteriorly into a thin, rod-like process, the rostrum basisphenoidale (Figs. 13, 15A and 15B). The posterior cup-shaped portion of the parabasisphenoid is anteroposteriorly approximately as long as it is transversely wide. Its dorsal surface is transversely and anteroposteriorly concave, producing a deeply excavated area that forms the floor of the central part of the endocranial cavity.

The dorsal surface of the parabasisphenoid cup is pierced by paired foramina for the abducens nerve (CN VI). The foramina are located at a level just anterior to the prootic contact of the parabasisphenoid. Each respective canalis nervi abducentis projects anteriorly and slightly medially through the dorsolateral wall of the parabasisphenoid, exiting into the sulcus cavernosus. Unfortunately, the thin canals and small foramina cannot be unambiguously identified in all specimens, but the position of the anteriorly exiting foramen seems to vary slightly. In CAMSM B55783 (*R. cantabrigiensis sensu* Collins, 1970), the anterior foramen nervi abducentis is located directly ventral to the clinoid process, but it is more posterolaterally placed in NHMUK PV OR35197 (*R. elegans sensu* Collins (1970); Data S1: Figs. S1.19 and S1.20).

The dorsal margin circumscribing the parabasisphenoid cup forms various structures. Posteriorly, the margin is very low and often bears a small posterior notch for the basis tuberculi basalis of the basioccipital, which is especially well developed in CAMSM B55776 and NHMUK PV OR35197. Posterolaterally and laterally, the margin becomes taller, forming a broad surface that faces posterodorsolaterally, which changes orientation slightly in its anterior part to face dorsolaterally. The posterodorsolaterally oriented portion of this margin faces the cavum acustico-jugulare and forms the ventral border of the hiatus acusticus ventral to the inner ear. The dorsolaterally oriented portion of the dorsal parabasisphenoid margin faces the ventromedial process of the prootic. A gap remains between both structures, but the space between them was likely bridged by cartilage. The parabasisphenoid margin anterior to the contact with the prootic is thin and sharp-edged. The margin also becomes taller in this portion of the parabasisphenoid. The anterolateral part of the margin forms the medial wall of the sulcus cavernosus. The clinoid processes arise on either side from the anterolateral margin at the level of the anterior margin of the trigeminal foramen, and project dorsally beyond the rest of the parabasisphenoid. The clinoid process is thin, and variably preserved in the specimens that were CT scanned, but seems to be complete in NHMUK PV OR35197. Medially, the clinoid processes are connected through the anterior margin of the parabasisphenoid cup, the dorsum sellae.

The raised dorsum sellae of turtles usually forms the posterior wall of the sella turcica, a distinct, relatively broad and often ‘U’-shaped depression or pit for the pituitary on the dorsal surface of the rostrum basisphenoidale (Gaffney, 1972). In turtles, the cerebral artery usually exits through paired foramina anterius canalis carotici cerebralis (unless stated otherwise, all carotid foramina and canals follow the nomenclature of Rabi et al., 2013), which are located in the posterolateral corners of the sella turcica, over which the dorsum sellae may anteriorly project to hide the foramina in dorsal view (Gaffney, 1979). In members of the total group of Chelonioidea, the foramina anterius canalis carotici cerebralis are usually relatively closely spaced within the sella turcica, which forms a dorsally tall wall of bone (e.g. Hirayama, 1998; Kear & Lee, 2006; Cadena & Parham, 2015; Evers & Benson, 2019). *R. pulchriceps* varies somewhat from this generalised condition. Instead of paired foramina for the cerebral artery, there is a single orifice at the contact of dorsum sellae and rostrum basisphenoidale (Figs. 15A and 15B; Data S1: Fig. S1.20). This foramen anterius canalis carotici cerebralis (faccc) is usually large and circular (although it is square in CAMSM B55775) and ramifies posteroventrally into a canal that bifurcates into right and left branches deep within the parabasisphenoid. We refer to these internal foramina as internal faccc (ifaccc). The same morphology is also present in some extant cheloniids (e.g. *Lepidochelys olivacea*), and is furthermore present in some other protostegids, such as *Notochelone costata* (Evers & Benson, 2019). The canals for the right and left cerebral canals diverge posteroventrolaterally from the single canal for the cerebral artery in *R. pulchriceps*, and merge with the canalis caroticus palatinum to continue posteriorly as the canalis caroticus internus (see Pterygoid, above, for descriptions of the latter two canals). If the parabasisphenoid is seen in isolation (Data S1: Figs. S1.19C–S1.19F), the point of bifurcation between the cerebral and palatine arteries can be located from the position of a medially directed foramen in the lateral surface of the parabasisphenoid (for the cerebral artery). According to the nomenclature of Rabi et al. (2013), this is not a true foramen posterius canalis carotici cerebralis (fpccc), as this foramen is said to only be developed in turtles in which the split between both arteries remains uncovered by bone. Here, we refer to the foramen as the internal fpccc to avoid confusion with respect to Rabi et al.’s (2013) nomenclature.

The sella turcica of *R. pulchriceps* is an elongate trough with dorsally raised lateral margins that excavates the dorsal surface of the rostrum basisphenoidale anterior to the faccc. The morphology of the sella turcica and the presence of a single canal for the anterior-most course of the cerebral arteries are also present in *Notochelone costata* (NHMUK PV R9590) and *Bouliachelys suteri* (QM F31669) (visible in CT scans). However, the parabasisphenoids of *Notochelone costata* and *Bouliachelys suteri* vary slightly from that of *R. pulchriceps* in having a median vertical ridge on the anterior surface of the dorsum sellae, situated dorsal to the faccc. The condition in *R. pulchriceps*, *Notochelone costata* and *Bouliachelys suteri* is similar to that present in modern cheloniids: *Chelonia mydas* (NHMUK 1969.776) has a narrow, trough-like sella turcica, in which the paired foramina anterius canalis carotici cerebralis lie close together and that extends anteriorly onto the dorsal surface of the rostrum basisphenoidale, and is laterally bordered by dorsally raised crests. *Eretmochelys imbricata* (FMNH 22242) has the same morphology, except that the lateral margins of the sella turcica terminate in anterodorsolateral processes that project from the rostrum basisphenoidale, the trabeculae. These trabeculae are very long in *Caretta caretta* (NHMUK 1940.3.15.1). In *Lepidochelys kempii* (M009/08; see Jones et al., 2012) the sella turcica is limited to a deep pit at the dorsal base of the rostrum basisphenoidale. However, the foramina anterius canalis carotici cerebralis seems to remain separate openings in all modern cheloniids, as well as in fossil chelonioids for which either the condition is described or noted during this study (*Ocepechelon bouyai*: Bardet et al., 2013; *Nichollsemys baieri*: Brinkman et al., 2006; *Ctenochelys stenoporus*: Matzke, 2007, FMNH PR 444; *Toxochelys* sp.: FMNH PR 219; *Allopleuron hofmanni*: NHMUK PV R4213; *Puppigerus camperi*: NHMUK PV R14375). Although *Toxochelys* sp. has closely spaced, separate foramina anterius canalis carotici cerebralis as in modern cheloniids, the sella turcica differs from that in *R. pulchriceps* and the aforementioned chelonioids, and is more like that in most other cryptodires in having a broad, ‘U’-shaped fossa on the dorsal surface of a flat rostrum basisphenoidale that is well sutured to the pterygoids (FMNH PR 219).

In *R. pulchriceps*, the rostrum basisphenoidale is rod-like and emerges ventral to the dorsum sellae, projecting anteriorly and slightly dorsally. The rostrum basisphenoidale forms the medial wall of the anterior course of the canalis caroticus palatinum, which is otherwise largely contained within the pterygoid (see Pterygoid, above). At its anterolateral margin, the rostrum basisphenoidale of *R. pulchriceps* has two weakly dorsally projecting flanges, which are interpreted as trabeculae.

The pterygoid contacts the parabasisphenoid along its entire lateral side, covering its ventral half. In the posterior part, where the margin of the parabasisphenoid cup is thickened, the contact surface of the parabasisphenoid for the pterygoid is broad and faces strongly ventrolaterally, so that the parabasisphenoid effectively overlaps the pterygoid. Anteriorly, parallel to the course of the sulcus cavernosus, the contact surface faces anterolaterally, but not ventrally. Further anteriorly, from the base of the rostrum basisphenoidale, the pterygoids form a midline contact and extend ventrally to the rostrum. The rostrum basisphenoidale is partially embedded in the dorsal part of the interpterygoid suture. Only the anterior end of the rostrum loses contact with the pterygoids, as also occurs in modern cheloniids and some fossil taxa (e.g. *Nichollsemys baieri*: Brinkman et al., 2006, TMP 97.99.1).

The ventral surface of the parabasisphenoid of *R. pulchriceps* is exposed as a triangular wedge between the pterygoids. Its surface texture is roughened by short, irregular longitudinal ridges, which are also present in modern cheloniids where they serve as muscle attachments (Gaffney, 1979).

### Cavum acustico-jugulare

The cavum acustico-jugulare is a broad cavity in the posteroventrolateral region of the skull (Figs. 15E and 15F). It is bordered by the quadrate laterally, the opisthotic and prootic dorsally, the prootic anterolaterally, the pterygoid ventrally, the exoccipital posteromedially, and the basioccipital posteroventromedially. The cavum acustico-jugulare represents a ventrally closed cranioquadrate space, which is unique to turtles amongst reptiles due to their fused basipterygoid articulation, which sutures the palate and quadrate to the neurocranium (Gaffney, 1979). The cavum acustico-jugulare can be separated into two sub-chambers, which Gaffney (1979) refers to as the cranioquadrate space portion and the recessus scalae tympani.

The cranioquadrate space portion is larger than the recessus scalae tympani and is positioned centrally between the canalis cavernosus anteriorly and the fenestra postotica posteriorly, and centrally between the cavum labyrinthicum medially and the cavum tympanum laterally. The cranioquadrate space portion of the cavum acustico-jugulare includes the medial part of the middle ear and is connected to its lateral part, the cavum tympanum, by the incisura columella auris of the quadrate. The stapes passes from the cavum tympanum through the incisura columella auris and then traverses the cranioquadrate space portion of the cavum acustico-jugulare (Data S1: Fig. S1.21). The stapedial footplate enters the fenestra ovale medially. The latter is formed by the prootic anteriorly and the processus interfenestralis of the opisthotic posteriorly. Ventrally, the margin of the fenestra ovale remains unossified in *R. pulchriceps*. Therefore, the cranioquadrate space portion of the cavum acustico-jugulare and the cavum labyrinthicum are confluent with the fenestra ovale ventrally. However, a parasagittal ridge on the dorsal surface of the pterygoid, which borders the course of the vena capitis lateralis medially, is aligned approximately with the border between the cavum acustico-jugulare and the cavum labyrinthicum (Fig. 13; Data S1: Fig. S1.16).

The recessus scalae tympani is a medial extension of the cavum acustico-jugulare in its posterior portion. It is formed by the opisthotic anteriorly and the exoccipital posteriorly. Medially, the recessus scalae tympani opens into the endocranial cavity via the anterior jugular foramen, which is bound by the opisthotic and exoccipital. The vena cerebralis posterior, as well as the vagus (CN X) and accessory (CN XI) nerves, enter the recessus scalae tympani through this opening (Gaffney, 1979). The fenestra perilymphatica is a ventrally open notch in the posterior surface of the opisthotic. It is a relatively large opening. The fenestra perilymphatica defines the posteromedial margin of the processus interfenestralis and connects the cavum labyrinthicum anteriorly with the recessus scalae tympani posteriorly. The fenestra perilymphatica is important for the dissipation of sound energy in the turtle ear. In turtles, a round window is absent (Henson, 1974; Wever, 1978; Hetherington, 2008). Instead, turtles have a re-entrant fluid flow, in which endolymph passes through the fenestra perilymphatica into a perilymphatic recess. This perilymphatic recess passes through the recessus scalae tympani and into the cranioquadrate space portion of the cavum acustico-jugulare, where it connects to the fenestra ovale to close the flow circuit.

The floor of the cavum acustico-jugulare is formed by the pterygoid. However, the pterygoid only extends posteriorly to the level of the incisura columella auris of the quadrate laterally and to the lateral process of the basioccipital medially. Hence, large parts of the posterior portion of the cavum acustico-jugulare are not closed ventrally, so that the ventral surface of the opisthotic is visible in ventral view. The posterior margin of the cavum acustico-jugulare is also unossified and the large, posteroventrally directed opening that results is termed the fenestra postotica. Several blood vessels enter the skull through the fenestra postotica, most notably the vena capitis lateralis, which continues anteriorly on the floor of the cavum acustico-jugulare into the canalis cavernosus, and the stapedial artery (Albrecht, 1976; Gaffney, 1979). In *R. pulchriceps*, a separate posterior jugular foramen is not present, but is incorporated in the medial margin of the fenestra postotica instead (Figs. 15E and 15F). Consequentially, the path of the vena cerebralis posterior is contained within the cavum acustico-jugulare but limited to the recessus scalae-tympani portion.

The roof of the cavum acustico-jugulare is pierced by a foramen shared between the quadrate and prootic, the aditus canalis stapedio-temporale. This foramen leads into a vertical tunnel dorsally, the canalis stapedio-temporale, which opens into the supratemporal fossa via the foramen stapedio-temporale. The stapedial artery exits the cavum acustico-jugulare through the canalis stapedio-temporale (Albrecht, 1976; Gaffney, 1979). Anteriorly, the cavum acustico-jugulare becomes constricted transversely and dorsoventrally and leads into the canalis cavernous, which extends anteroventrally from the cavum acustico-jugulare and between the quadrate, prootic, and pterygoid. The anterior part of the cavum acustico-jugulare serves as a passage for the hyomandibular branch of the facial nerve (CN VII), which extends posteriorly from the prootic contribution to the medial wall of the canalis cavernosus. The mandibular artery, an anterior branch of the stapedial artery, usually passes through the anterior portion of the cavum acustico-jugulare in cryptodires also and continues through the canalis cavernosus before it exits into the subtemporal fossa via the trigeminal foramen (Gaffney, 1979). However, this cannot be confirmed for *R. pulchriceps*, as the mandibular artery leaves no osteological correlate.

### Stapes

One specimen (CAMSM B55776) preserves a stapes in its original position, with the stapedial footplate articulated with the prootic part of the fenestra ovale (the opisthotic is not preserved in this specimen) (Data S1: Figs. S1.21 and S1.22). It consists of a disk-shaped stapedial footplate, which is continuous with an elongate, lateral rod-like shaft.

The stapedial shaft is gently curved, with the convex part facing ventrally. This contrasts with the straight stapes of *Plesiochelys etalloni* (Carabajal et al., 2013). The stapedial shaft of *R. pulchriceps* has a circular cross-section. The stapedial shaft traverses the incisura columella auris of the quadrate. The part of the stapedial shaft that projects laterally into the cavum tympanum is slightly expanded in relation to the mid-part of the shaft. It extends only a few millimetres into the cavum tympanum and does not reach the lateral extent of the cavum, where the tympanic membrane would presumably close the cavum tympanum laterally. The space between the tympanic membrane and lateral tip of the stapes was most likely bridged by a cartilaginous extrastapes.

Medially, the stapedial shaft expands to form a circular stapedial footplate. The medial surface of the stapedial footplate is gently depressed to form a shallow fossa. The anterior margin of the stapedial footplate articulates closely with the prootic margin of the fenestra ovale, leaving no space between them.

### Lower Jaw

#### Dentary

The dentary is preserved in articulation with the cranium in two of the *R. pulchriceps* specimens that were CT scanned: CAMSM B55776 (*R. elegans sensu* Collins (1970); Figs. 16A–16C) and CAMSM B55783 (*R. cantabrigiensis sensu* (Collins, 1970); Figs. 16D–16F). Both specimens have consistent dentary morphology, but CAMSM B55776 also includes the rest of the lower jaw elements on the right side, whereas CAMSM B55783 preserves only the left surangular. The dentary contacts the coronoid posteromediodorsally, the surangular posteriorly, and the prearticular and angular medially.

The dentary of *R. pulchriceps* consists of fused right and left rami, which meet in an anteroposteriorly extended symphysis that forms a triangular triturating surface dorsally. The symphyseal region is longer than in *Dermochelys coriacea* (UMZC R3031), but shorter than in cheloniids with extensive secondary palates, such as *Lepidochelys olivacea* (SMNS 11070). The rami of the dentary in *R. pulchriceps* are mediolaterally narrow and straight processes that diverge at an angle of 55° from the symphysis in dorsal view. In lateral view, the depth of the rami increases slightly posteriorly toward the coronoid process (=processus coronoideus of Gaffney, 1979). Anteriorly, the dentary is gently curved dorsally to form a shallow, pointed beak. The labial ridge of the dentary is sharp-edged around the triturating surface and continues as a blunt ridge on the diverging dentary rami. The triturating surface also bears a median ridge, which extends over its entire anteroposterior length (Figs. 16D and 16E). The edge of the ridge is sharp in both specimens, but the posterior end of the ridge is slightly expanded transversely in CAMSM B55776 (Fig. 16C), but it is not in CAMSM B55783 (Figs. 16D and 16E). The triturating surface extends posteriorly onto the rami of the dentary, where it forms a narrow surface that slopes lingually.

A lingual ridge is absent in *R. pulchriceps*. The lingual margin of the triturating surface forms the dorsal margin of the sulcus for the Meckelian cartilage. This sulcus forms a shallow, longitudinal groove that extends from the medial surface of the dentary rami to the posterior surface of the symphyseal region. Here, it forms a deep anterior pocket that extends into the dentary ventrally to the level of the triturating surface. The sulcus for the Meckelian cartilage becomes dorsoventrally deeper posteriorly on the dentary rami, and merges into the Meckelian fossa. The subalveolar foramen (=foramen alveolare inferius of Gaffney, 1979) opens from the posterodorsal margin of the sulcus for the Meckelian cartilage into the dentary ramus. It extends anteromedially as the subalveolar canal (=canalis alveolaris inferior of Gaffney, 1979). CT scans of CAMSM B55783 show that this canal extends to the anterior tip of the dentary and emits numerous smaller canals. Most of these canals branch from the dorsal side of the subalveolar canal, are posterodorsally directed, and exit the dentary on its lateral surface just ventral to the lingual ridge. The subalveolar foramen is covered medially by the prearticular and angular and is not level with the medial intermandibular foramen (=foramen intermandibularis medius of Gaffney, 1979; see Angular, Prearticular, and Splenial, below), but positioned ventral to it.

The medial surface of the dentary has a small, anteriorly directed pocket on the posterior end of the triturating surface, dorsal to the level of the subalveolar foramen and the Meckelian fossa. This pocket receives an anterior process of the prearticular. The ventral surface of the dentary is lingually expanded and ventrally convex, so that is covers the surangular ventrally. The lateral surfaces of the dentary rami are flat, and no distinct depression for the external mandibular adductor musculature is evident. Anterior to the coronoid process, the lateral surface bears a small foramen dentofaciale majus, which is anteromedially directed. The associated canal merges internally with the subalveolar canal.

#### Angular, prearticular, and splenial

The bones on the medial surface of the mandible are preserved in CAMSM B55776, but the sutures between them cannot be completely traced in CT scans of the specimen. Consequently, these bones were segmented as a single structure and are described together here (Figs. 16B and 16C). The topological positions of the preserved bones are consistent with their identification as a prearticular, which seems to be completely preserved, and a partially preserved angular. The angular usually covers most of the ventral margin of the mandible, but also extends onto its medial surface where it has its deepest extent at the level of the coronoid process. In CAMSM B55776, the ventral margin of the mandible is incompletely preserved, so large parts of the angular seem to be missing. As the sutural contacts between the elements of the medial mandibular surface are not clear in the available CT scans, and because our model of these elements extends far anteriorly, it is likely that a splenial is incorporated into the segmented model, which would be located in the anterior portion of the prearticular (Gaffney, 1979).

The angular and prearticular form a thin vertical sheet that provides the medial wall of the Meckelian fossa. The angular is anteroventrally positioned with respect to the prearticular (Gaffney, 1979). Anteriorly, a thin, rod-like process extends into the floor of the sulcus for the Meckelian cartilage, which we interpret to be the splenial. This anterior rod gets dorsoventrally deeper posteriorly, at which point it clearly represents part of the angular. The position of the angular-splenial contact is unclear. The medial mandibular elements cover the sulcus for the Meckelian cartilage completely posterior to the position of the medial intermandibular foramen (foramen intermandibulare medius of Gaffney, 1979). Posterior to this foramen, the sulcus for the Meckelian cartilage is confluent with the Meckelian fossa. In most turtles, the medial intermandibular foramen is located slightly posterior to the level of the subalveolar foramen (Gaffney, 1979), but in *R. pulchriceps* the angular and prearticular extend anteriorly to cover the subalveolar foramen medially.

Some indication of the angular-prearticular contact can be seen anteriorly, between the anterior and posterior intermandibular foramina (foramen intermandibulare oralis and foramen intermandibulare caudalis of Gaffney, 1979, respectively). The former is located just ventral to the medial intermandibular foramen and seems to be contained mainly in the angular. The posterior intermandibular foramen is positioned posteroventral to the former, at the level of the anterior end of the coronoid process and seems to be formed largely by the prearticular. Posterior to this foramen the suture cannot be traced.

The prearticular articulates with a pocket of the dentary posterodorsal to the medial intermandibular foramen. The combined angular and prearticular have their deepest extent posterior to this articulation. The prearticular contacts the coronoid medially to the dorsal margin of the Meckelian fossa.

Posterior to the contact with the coronoid, the prearticular gets dorsoventrally shallower, so that the surangular is exposed in medial view above the dorsal margin of the prearticular. At its posterior end, the prearticular becomes transversely slightly broader and contributes to the medial portion of the mandibular articulation facet (= area articularis mandibularis of Gaffney, 1979). The posteroventral margin of the prearticular (and possibly of the angular) contacts the articular. Just anterior to this expanded posterior end, there is a small but deep circular depression on the medial surface of the prearticular. The CT scans do not reveal if this depression leads to an interior canal and its identity is unclear.

The ventral margin of the angular forms a sharp edge that lies medially against the dentary with this contact continuing along the surangular more posteriorly. Along the surangular contact, the angular, and possibly more posteriorly the prearticular, are laterally expanded to form a short shelf that forms parts of the medial floor of the Meckelian fossa.

#### Surangular

Surangulars are preserved on the right side of CAMSM B55776 (Figs. 16A–16C) and the left side of CAMSM B55783 (Figs. 16D and 16E). The surangular of *R. pulchriceps* is plate-like and situated in the posterolateral portion of the mandible. It contacts the dentary anterolaterally, the coronoid anterodorsally, the prearticular/angular ventrally, the articular posteromedially, and covers the Meckelian fossa laterally. The ventral surface of the surangular is slightly expanded medially and meets the prearticular in the floor of the Meckelian fossa.

The surangular reaches its greatest depth at the level of the coronoid, which it contacts along its dorsomedial surface. Both specimens have a semicircular, recessed facet on the medial surface of the dorsal tip of the surangular for articulation with the coronoid. The dorsal margin of the surangular is convex anteriorly around the coronoid contact. Posteriorly, the dorsal surangular margin is concave and slopes posteroventrally to the mandibular glenoid fossa. The surangular has an anteroventral process, which is preserved only in CAMSM B55776. The anteroventral process has a narrow, splint-like shape, and extends along the angular in the floor of the Meckelian fossa. More than half of the length of the surangular is covered laterally by the dentary.

The Meckelian fossa is expressed as a broad depression on the medial surface of the surangular. The dorsal opening of the Meckelian fossa is formed by the concave posterodorsal margin of the surangular laterally, the prearticular medially, and the coronoid anterodorsally.

**Figure 17:**
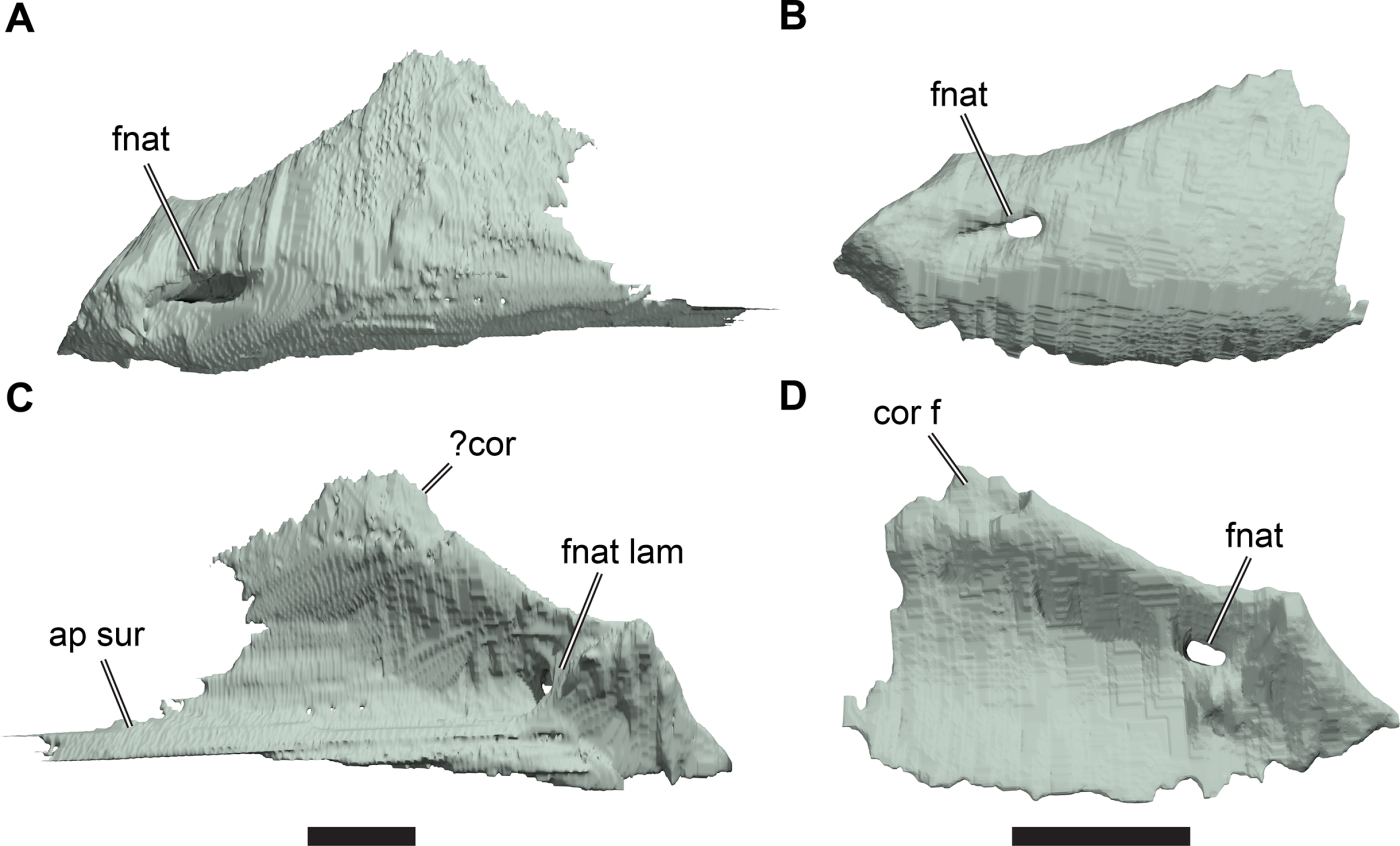
Comparison of surangulars. (A) 3D rendering of right surangular of CAMSM B55776 in lateral view; (B) 3D rendering of left surangular of CAMSM B55783 in lateral view, reflected for comparison; (C) 3D rendering of right surangular of CAMSM B55776 in medial view; (D) 3D rendering of left surangular of CAMSM B55783 in medial view, reflected for comparison. Scale bars equal three mm. Abbreviations: *ap sur*, anterior process of surangular; *cor f*, coronoid facet; *fnat*, foramen nervi auricolotemporalis, *fnat lam*, foramen nervi auricolotemporalis lamina.

Posteriorly, the surangular expands mediolaterally to form a relatively broad articular process. The ventral margin of the posterior process is horizontally aligned with the anteroventral margin of the element in CAMSM B55776, so that the ventral margin of the surangular appears relatively straight. In contrast, the ventral margin is strongly curved convexly in CAMSM B55783 (Fig. 17). The posterior surface of the articular process is roughly triangular in outline in both specimens and forms a posterodorsally exposed facet for the mandibular glenoid. A short canal pierces the surangular just anterior to its articular process. The lateral opening of this canal is the foramen nervi auricolotemporalis, which is oval in shape. The short canal leads anteromedially through the surangular and exits into the posterior corner of the Meckelian fossa. In CAMSM B55776, this medial opening is posteroventrally overlapped by a small lamina that is absent in CAMSM B55783 (Fig. 17).

#### Coronoid

CAMSM B55776 is the only CT-scanned specimen that includes a (right) coronoid. It is a small bone on the dorsal margin of the mandibular ramus (Figs. 16A–16C) and is positioned in its posterior third, so that the mandibular rami have much longer pre-coronoid than post-coronoid portions. The coronoid contacts the dentary anterolaterally, the surangular posterolaterally, and the prearticular anteromedially.

The coronoid has a semilunate outline with a convex dorsal margin and an approximately straight ventral margin. Ventrally, it is slightly broader transversely than at its dorsal margin. The convex dorsal margin forms the coronoid process, which is relatively low in *R. pulchriceps*. With its ventral surface, the coronoid forms a bridge over the Meckelian fossa, forming parts of its roof and its anterodorsal border. The coronoid is overlapped laterally by the dentary in its anterior part and by the surangular in its posterior part, so that most of its lateral surface is obscured in lateral view.

#### Articular

The articular is preserved on the right side of CAMSM B55776. It is a small, block-like element situated at the posterior end of the jaw (Figs. 16B and 16C). In *R. pulchriceps*, it is triangular, anteriorly narrow, and posteriorly broad. It is wedged between the posterolateral end of the prearticular and the posteromedial corner of the articular process of the surangular. In many turtles, the articular forms the posterior wall of the Meckelian fossa, but in *R. pulchriceps* the element is dorsoventrally low and posteriorly displaced, so that the Meckelian fossa seems to be completely closed posteriorly by the surangular and prearticular. The dorsal surface of the articular is a convex hemisphere as in pleurodires and lacks the longitudinal ridge that commonly separates it into medial and lateral articular facets as commonly observed in cryptodires (Gaffney, 1979). The mandibular glenoid fossa is shared between the prearticular, surangular, and articular, with the latter two bones contributing most of its surface. A foramen posterius chorda tympani is absent, which is also the case in modern cheloniids (Gaffney, 1979). A convexity for the insertion of the m. depressor mandibulae is also absent in *R. pulchriceps*.

## Phylogenetic Analysis

### Character-taxon matrix

We used a modified version of the data matrix from Evers & Benson (2019) that included 16 additional taxa (*Adocus lineolatus*, *Angolachelys mbaxi*, *Cabindachelys landanensis*, *Calcarichelys gemma*, *Chelosphargis advena*, *Corsochelys halinches*, *Erquelinnesia gosseleti*, *Nichollsemys baieri*, *Ctenochelys* sp., *Galianemys whitei*, *Oligochelone rupelensis*, *Peritresius martini*, *Petrochelys kyrgyzensis*, *Plesiochelys bigleri*, *Procolpochelys charlestonensis*, and *R. nammourensis*). *Adocus lineolatus* and *Petrochelys kyrgyzensis* were added because they are presumed stem-group trionychians (*Adocus lineolatus*) or early members of the trionychian crown-group (*Petrochelys kyrgyzensis*) and can therefore influence character optimisation and polarisation at the base of Cryptodira. For both these taxa, we used CT scans to complement published sources to inform our character scorings (data for *Petrochelys kyrgyzensis* were downloaded from MorphoSource project M9547-13212; Vitek et al., 2018). *Galianemys whitei* was added as a bothremydid pleurodire using information from CT scans, personal observations (by SWE) and the literature, because bothremydids were not represented in the original matrix of Evers & Benson (2019). *Plesiochelys bigleri* was included as an additional plesiochelyid, as a CT scan of the cranium was available, and because it represents a thalassochelydian for which cranial as well as postcranial material is known (Püntener, Anquetin & Billot-Bruyat, 2017). *Angolachelys mbaxi* was added so that all currently known sandownids are included in our matrix. *Cabindachelys landanensis*, *Oligochelone rupelensis*, and *Procolpochelys charlestonensis* were incorporated as presumed stem-group cheloniids from the Cenozoic. *Ctenochelys* sp. and *Peritresius martini* were added because these have been recently interpreted as Cretaceous stem-group cheloniids (Gentry, 2016; Gentry et al., 2018). *Corsochelys halinches* and *Nichollsemys baieri* were included as they represent Cretaceous taxa with contentious phylogenetic positions within the total group of chelonioids (Zangerl, 1960; Brinkman et al., 2006). *Erquelinnesia gosseleti* was added to represent ‘osteopygid’ chelonioids, which were not included in the original matrix of Evers & Benson (2019). Finally, *Calcarichelys gemma*, *Chelosphargis advena*, and *R. nammourensis* were added to the matrix as they all represent protostegid taxa for which cranial and postcranial material is available. The information sources for each taxon are detailed in supplementary Table S1.2, and the matrix is deposited in the supplements in nexus-format (Data S2).

Evers & Benson (2019) focused largely on a revision of cranial characters and modified postcranial characters only by vetting their implied homology statements according to the hierarchical coding strategy (Hawkins, Hughes & Scotland, 1997), retaining postcranial character scores directly from Cadena & Parham (2015). Here, we provide a more thorough revision of postcranial characters in an attempt to code variation among chelonioids for parts of the skeleton that have been interpreted to be important for their marine lifestyle, such as carapacial and plastral ossification and limb morphology. Specifically, we modified characters regarding the costals and costal fontanelles, the ento- and epiplastron, the xiphiplastra, the plastral serrations, the humerus, and the femur. Our revisions resulted in the modification (i.e. splitting, recombination, or change of character state definitions) of several characters, as well as the addition of nine characters that have not been used in previous analyses, although some of the observations underlying these characters had been mentioned throughout the literature before. Character modifications, previous character definitions, and new characters are detailed, justified, and illustrated in the supplements (Data S1: Figs. S1.23–S1.31). We further added and/or revised postcranial scorings for several species based on first-hand observations of holotype and referred specimens (e.g. *Puppigerus camperi*, *Eochelone brabantica*, *Eosphargis breineri*; see Data S1: Table S1.2 for details). Two new cranial characters were added, based on observations made during our investigations of *R. pulchriceps*. A full character list is deposited in the supplements (Data S3). Our modifications resulted in a data matrix containing 96 taxa and 355 characters.

### Parsimony analysis

**Figure 18:**
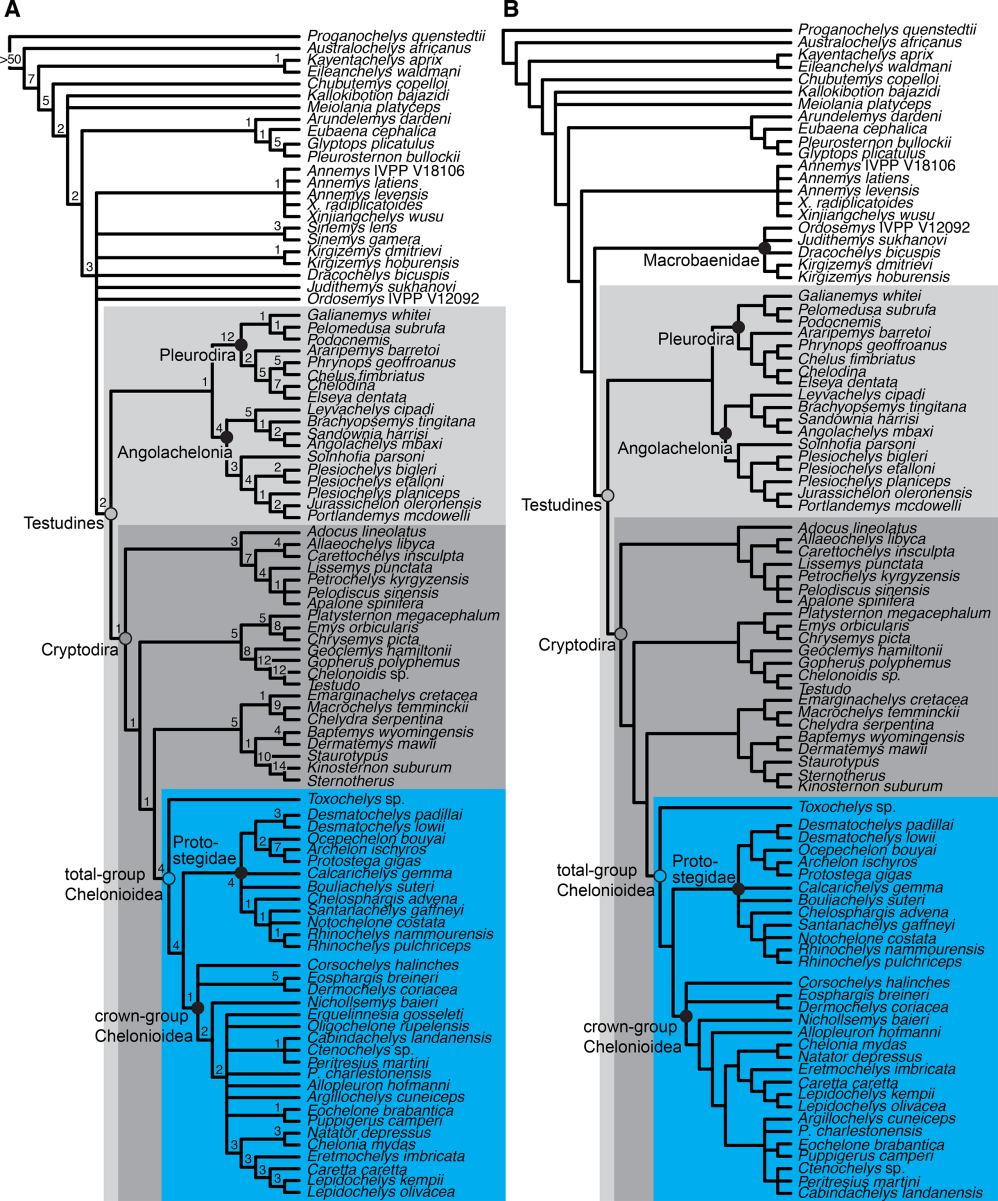
Consensus trees of our phylogenetic analysis. (A) strict consensus tree of >10,000 MPTs. (B) reduced strict consensus tree obtained by pruning the *Sinemys* clade (*Sinemys lens* + *Sinemys gamera*), *Erquelinnesia gosseleti* and *Oligochelone rupelensis*.

The dataset was analysed using TNT 1.5 for Windows (Goloboff, Farris & Nixon, 2008; Goloboff & Catalano, 2016). We used a molecular backbone constraint following the topology of Pereira et al. (2017) for extant taxa, whereas all fossil taxa were left unconstrained. *Proganochelys quenstedtii* was used as the outgroup. All characters were treated as equally weighted and unordered. We employed the new technology search algorithm in TNT with default settings and enabled tree drifting (Goloboff, 1999) and parsimony ratchet (Nixon, 1999). The initial level of driven search was set to 30, and the number of times the minimum tree length should be obtained was set to 30. We subjected the most parsimonious trees (MPTs) of this initial analysis to further tree bisection and reconnection (TBR). The resulting MPTs were used to construct a strict consensus tree, shown in Fig. 18A. TNT was used to calculate absolute Bremer decay indices as a measure of branch support. Because the strict consensus tree topology was unresolved in part (see Results of phylogenetic analysis below), we used the following approaches to identify ‘wildcard’ taxa that occupied multiple distinct phylogenetic positions among the set of MPTs: (1) inspection of an Adams consensus tree in PAUP* for Macintosh (Swofford, 2002) and (2) executing an iterative PCR (Pol & Escapa, 2009) in TNT. Both methods identified the *Sinemys* clade (*Sinemys lens* + *Sinemys gamera*), *Erquelinnesia gosseleti*, and *Oligochelone rupelensis* as the most unstable taxa which, if pruned from the consensus tree, result in an increase in resolution. Among the MPTs, the *Sinemys* clade was variously grouped with other sinemydids/macrobaenids or was found as the sister group to xinjiangchelyids. *Erquelinnesia gosseleti* was found in various positions along the stem-group of cheloniids with *Allopleuron hofmanni*, *Procolpochelys charlestonensis*, the group that included *Eochelone brabantica* or the group that included *Ctenochelys* sp. *Oligochelone rupelensis* was found in the same positions, except it was never found to form a clade with *Allopleuron hofmanni*. These ‘wildcard’ taxa were pruned accordingly to produce a reduced strict consensus tree (Fig. 18B). For visualisation, this tree was scaled to geological time using an a posteriori scaling method that uses first and last appearance dates for fossil taxa and an arbitrary minimum branchlength parameter (Laurin, 2004), by using commands from the *strap* (Bell & Lloyd, 2014) and *palaeotree* packages (Bapst, 2012). Ages and calibration settings, including minimum constraints for the ages of Cryptodira and Pleurodira, were used as in Evers & Benson (2019), and these data and the additional age ranges for taxa added to our study are provided in the supplements (Data S4). One additional constraint for the minimum age of the crown-group of Kinosternoidea was added based on the early Maastrichtian stem-group kinosternid *Yelmochelys rosarioae* (Brinkman et al., 2016). We chose this simple time calibration method to illustrate gross effects of our topological results on the timing of origination for the chelonioid subgroups investigated. A more thorough method that takes stratigraphic uncertainty into account and models divergence times simultaneously with topology is beyond the scope of this study and will be provided elsewhere. The resulting calibrated tree was further summarised to focus on chelonioids (see Calibrated tree and stratigraphic congruence below).

Character optimisation was performed in PAUP*, because TNT only returns unambiguous synapomorphies, whereas the optimality criterion (i.e. ACCTRAN or DELTRAN) can be manually set in PAUP*. For the optimisation, a single MPT was selected from all MPTs. To select an MPT for optimisation, we computed a 50% majority rule consensus tree from the MPTs gained from the original analysis. The topology of this majority rule consensus tree was used as a constraint in PAUP* to find all MPTs compatible with the majority consensus rule topology. One of these MPTs was chosen at random for the optimisation. The topologies for the majority rule consensus tree (Data S1: Fig. S1.32), the MPT selected for optimisation (Data S1: Fig. S1.33), and the optimisation for all nodes, as well as for all characters are given in the supplements (Data S1 and S3, respectively).

### Topological results of phylogenetic analysis

Our parsimony analyses resulted in 138 MPTs that are 1724 steps in length. Additional TBR branch-swapping on these MPTs resulted in >10,000 MPTs. The most important gross topological findings of our analysis are (1) the recovery of xinjiangchelyids as stem-group turtles (as in Evers & Benson, 2019); (2) low resolution for sinemydids and macrobaenids, which form a polytomy with xinjiangchelyids and crown-group turtles (sinemydids-macrobaenids formed a clade in Evers & Benson, 2019); (3) the recovery of a monophyletic Angolachelonia as the sister group to pleurodires (Angolachelonia was the sister taxon to crown-group turtles in Evers & Benson, 2019); (4) protostegids form a monophyletic group on the stem lineage of chelonioids, in a position one node more crownward than *Toxochelys* sp. (protostegids were recovered on the stem of *Dermochelys coriacea* in Evers & Benson, 2019) (Fig. 18).

Our finding of angolachelonians as the sister group to pleurodires is unexpected. Evers & Benson (2019) found Angolachelonia (i.e. thalassochelydians + sandownids) as the most crownwardly positioned group of stem turtles. The topological change from this inferred position to being stem-group pleurodires is relatively little in terms of parsimony cost, and the sister-group relationship of pleurodires and angolachelonians found in this study is poorly supported (Bremer support value = 1). Because we are primarily concerned with the ingroup relationships of the total group of chelonioids in this study, we do not further explore the angolachelonian-pleurodire relationship herein. However, we note that the position of pleurodires has been unstable in many phylogenetic analyses of global turtle datasets (e.g. Sterli, 2010; Zhou, Rabi & Joyce, 2014), and uncertainty regarding the phylogenetic position of pleurodires might explain not only the unexpected sister-group relationship with angolachelonians here, but might also cause the controversy surrounding the relative position of xinjiangchelyids, sinemydids, and macrobaenids as stem-group turtles or stem-group cryptodires (e.g. Zhou, Rabi & Joyce, 2014; Zhou & Rabi, 2015). Examination of a more complete sample of early stem- and crown-group pleurodires will be important for character polarisations at the base of Pleurodira and resolving the membership of their total group.

Evers & Benson (2019) found protostegids to be on the stem lineage of *Dermochelys coriacea*, and specifically as the sister group to *Dermochelys coriacea* + *Eosphargis breineri*. The position of protostegids within Chelonioidea was relatively strongly supported. However, in a series of Templeton’s tests, Evers & Benson (2019) could not reject the hypothesis that protostegids are stem-group chelonioids, and this relationship was found by Evers & Benson (2019) in an analysis in which they only included a subset of Early–early Late Cretaceous protostegids (*R. pulchriceps*, *Santanachelys gaffneyi*). Recently, Raselli (2018) also found protostegids as stem-group chelonioids by addition of five characters to the dataset of Cadena & Parham (2015), which had previously recovered protostegids as dermochelyids. Our results are mainly due to the modifications of postcranial characters and scores relative to Evers & Benson (2019), and provide independent evidence for the finding of Raselli (2018), as her characters were not implemented in our study. More crownward positioned chelonioids (with regard to protostegids), as well as crown-group chelonioids, share several postcranial features that are absent in both protostegids and *Toxochelys* sp. These include, for instance, the presence of rib-free peripherals (character 216.1), the absence of a posterior contact of the xiphiplastra (character 262.0; independently absent in some cheloniids such as *Puppigerus camperi*), the absence of a 3rd phalanx on the 5th manual digit (character 347.0), or the 3rd manual digit being the longest within the hand (character 348.1).

Most phylogenetic studies that included protostegids found the Late Cretaceous taxa *Archelon ischyros* and *Protostega gigas* to be nested within a paraphyletic grade of protostegids from the Early Cretaceous, and *Santanachelys gaffneyi* was often found as the earliest branching protostegid (Hirayama, 1994; Kear & Lee, 2006). The analysis of Evers & Benson (2019) instead found *Archelon ischyros* + *Protostega gigas* in a clade that was the sister group to all other protostegids. Here, we find two principal clades of protostegids: one includes the Late Cretaceous *Protostega gigas*, *Archelon ischyros*, *Ocepechelon bouyai*, *Desmatochelys lowii*, and the Early Cretaceous *Desmatochelys padillai* (Fig. 18). The species of *Desmatochelys* are found as sister taxa, as in Cadena & Parham (2015) and Evers & Benson (2019). The second clade of protostegids is composed mainly of Early Cretaceous and early Late Cretaceous taxa and includes *R. pulchriceps*, *R. nammourensis*, *Santanachelys gaffneyi*, *Notochelone costata*, and *Chelosphargis advena*. The species of *Rhinochelys* are found as sister taxa, supporting the generic assignment of *R. nammourensis* by Tong et al. (2006). *Bouliachelys suteri* and *Calcarichelys gemma* are found in a polytomy with both protostegid subclades.

*Corsochelys halinches* is recovered in a polytomy with dermochelyids and cheloniids: therefore, it is currently unclear if *Corsochelys halinches* is a stem-group chelonioid or a crown-group chelonioid on the stem of either *Dermochelys coriacea* or extant cheloniids. Consistent with previous suggestions (Nielsen, 1959; Wood et al., 1996; Joyce et al., 2013) and the findings of Evers & Benson (2019), *Eosphargis breineri* is found on the stem-group of *Dermochelys coriacea*.

The ingroup relationships of cheloniids show poorer resolution in this study than in Evers & Benson (2019), but this is because we included more taxa and taxa with relatively fragmentary remains, such as *Peritresius martini*. We find *Nichollsemys baieri* from the late Campanian of North America as the earliest stem-group cheloniid, rather than as a toxochelyid stem-group chelonioid as in study first describing *Nichollsemys baieri* (Brinkman et al., 2006). One node more crownward, we find a large polytomy that includes extant cheloniids (as a clade), (*Eochelone brabantica* + *Puppigerus camperi*), (*Cabindachelys landanensis* + *Ctenochelys* sp. + *Peritresius martini*), *Erquelinnesia gosseleti*, *Oligochelone rupelensis*, *Procolpochelys charlestonensis*, *Allopleuron hofmanni*, and *Argillochelys cuneiceps*. Our results support the hypothesis of several recent studies that proposed cheloniid affinities for *Ctenochelys* sp. (Gentry, 2016; Gentry et al., 2018). Alternatively, *Ctenochelys* sp. had been hypothesised to be a stem-group chelonioid, often in a clade with *Toxochelys* sp. (Brinkman et al., 2006). Gentry et al. (2018) found a clade that includes *Ctenochelys* and *Peritresius*, which is supported by our study, but we additionally find *Cabindachelys landanensis* within this clade. *Cabindachelys landanensis* was instead found in a clade with *Erquelinnesia gosseleti* in Myers et al. (2018).

Our finding of a *Puppigerus camperi* + *Eochelone brabantica* clade is in accordance with previous studies of these taxa (e.g. Moody, 1974). *Eochelone brabantica* was found as the earliest branching stem-group cheloniid in Evers & Benson (2019), but that was most likely the result of poor character sampling: in Evers & Benson (2019), *Eochelone brabantica* was scored primarily on the basis of an incomplete, CT-scanned skull and no postcranial material. Since this earlier paper, one of us (SWE) studied the holotype and referred specimens of *Eochelone brabantica*, so that we could now expand the cranial scorings and add postcranial scorings for this taxon.

*Puppigerus camperi* (and by extension the clade of *Puppigerus camperi* + *Eochelone brabantica*) is most often found to be the immediate sister taxon of crown-group cheloniids (Hirayama, 1994; Cadena & Parham, 2015; Gentry, 2016; Gentry et al., 2018), although this could sometimes be an artefact of small sample sizes of fossil cheloniids (Weems & Brown, 2017 find some rarely included Oligocene and Miocene taxa more closely related to crown-group cheloniids than is *Puppigerus camperi*). Our cheloniid in-group relationships are too poorly resolved to thoroughly examine whether *Puppigerus camperi* + *Eochelone brabantica* represent a more recently or less recently diverging clade of stem-group cheloniids with respect to the youngest (Oligocene and Miocene) taxa we included, because *Oligochelone rupelensis* and *Procolpochelys charlestonensis* are found in a polytomy that includes crown-group cheloniids as well as *Puppigerus camperi* + *Eochelone brabantica*.

Many studies also found *Argillochelys cuneiceps* to be closely related to *Eochelone brabantica* and *Puppigerus camperi* (e.g. Hirayama, 1994) and Moody (1968, 1974) referred these three taxa to his subfamily Eochelyinae. The phylogenetic position of *Argillochelys cuneiceps* is unresolved with regard to other stem-group cheloniids in our analysis. This likely results from the fact that we did not include postcranial scorings for *Argillochelys cuneiceps* as most of the material that we studied (in the NHMUK) have incorrect or contradictory specimen labels, and many specimens are labelled as various combinations of ‘*Argillochelys*’, ‘*Lytoloma*’, ‘*Eochelys*’ and ‘*Puppigerus*’. Therefore, in the absence of cranial-postcranial associations of *Argillochelys cuneiceps*, it is not entirely clear to us, which postcranial specimens pertain to *Argillochelys cuneiceps*. Specimens of *Puppigerus camperi* are easier to identify, because several near-complete specimens have been found in association (NHMUK PV R14375) or articulation (IRSNB R 0072, 0073) with *Puppigerus camperi* skulls. In our reduced strict consensus tree, *Argillochelys cuneiceps* is found in a polytomy with (*Eochelone brabantica* + *Puppigerus camperi*) and (*Ctenochelys* sp. + *Cabindachelys landanensis* + *Peritresius martini*), indicating that there is some support for a relatively close relationship with *Puppigerus camperi*.

Pruning *Erquelinnesia gosseleti* and *Oligochelone rupelensis* from the strict consensus tree results in an additional node, placing *Allopleuron hofmanni* as a stem-group cheloniid that is positioned one node more crownward than *Nichollsemys baieri*. Excluding *Sinemys lens* and *Sinemys gamera* also improves the resolution, and results in a clade that contains macrobaenids and sinemydids, which are found as the sister group to crown-group turtles and thus one node crownward of xinjiangchelyids. This result is compatible with the topology found in Evers & Benson (2019).

## Principal Component Analysis

### Skull measurements and PCA procedure

We recorded a set of measurements for all specimens of *R. pulchriceps* that we investigated first-hand. Proportional differences based on these measurements have been used as autapomorphies for various species of *R. pulchriceps* (Collins, 1970; Scavezzoni & Fischer, 2018; see Discussion). Because some authors have raised doubts regarding the taxonomic utility of these measurements and ratios (Smith, 1989; Hooks, 1998), we reproduced the measurements of Collins (1970) and added some new measurements: (1) pre-parietal skull length (measurement ‘z’ of Collins, 1970); (2) skull width (measurement ‘x’ of Collins, 1970); (3) skull height (measurement ‘y’ of Collins, 1970). We modified measurement ‘y’ of Collins (1970), which was illustrated as the skull height from the labial ridge of the skull to the frontal sulcus, and instead measured the skull height from the lateroventral skull margin to the skull roof formed by the parietals, because this was easier to measure; (4) interorbital skull width (new measurement); (5) nasal length–width ratio (new measurement). Because we found the nasal bones to show extreme shape variation (see Description and Discussion), we measured the anteroposterior length of the left and right nasals along their median contact on the skull roof, the straight-line mediolateral width of the nasals at the narial margin, and calculated the length–width ratio for nasals from these measurements; (6) the jaw angle of Collins (1970), which was described as the ‘angle between the two maxillae’ (Collins, 1970: p. 357), was measured by drawing straight lines through the labial ridges of the maxillae in ventral view of photographs and taking the angle between the resulting lines. All length measurements were taken with digital callipers and the jaw angle was measured in the software ImageJ. We did not take individual measurements when a specimen was extremely distorted, which was specifically the case for several skull height and width measurements. Data for *R. amaberti* were measured from the figures provided in Scavezzoni & Fischer (2018). All measurements are recorded in supplementary Table S1.3.

To determine whether these measurements support the taxonomy of Collins (1970) and Scavezzoni & Fischer (2018), we used principal component analysis (PCA) to investigate if specimens assigned to different species form separate clusters in morphospace. We only included the skull width, the skull height, the pre-parietal skull length and the jaw angle in a first analysis, as these were the measurements considered originally by Collins (1970). All our measurements were included in a second analysis. The PCA was conducted on those specimens, for which all respective measurements could be taken (*n* = 31 for first analysis; *n* = 27 for second analysis; see Data S1: Table S1.3) and was computed in R (R Core Team, 2016) using the *prcomp* command and the ‘scale = TRUE’ argument. This argument transforms the data to have unit variance, which is important for our data because it includes measurements of different units and is equivalent to using a correlation matrix (Jolliffe & Cadima, 2016). Because the first principal component (PC1) described size and allometric shape changes (see PCA results), we excluded it for constructing bivariate morphospace plots and re-scaled the absolute variance explained by the remaining PCs to represent the proportion of non-size (i.e. shape) variation. We assigned colours to individual specimens according to the taxonomic identification of Collins (1970) and assigned a different point symbol to the holotype specimens of all four species.

### PCA results

Our principal component analyses show similar patterns across our data partitions. For brevity, only results from our second PCA (including all measurements) are presented in detail but results for the first PCA are presented in the supplements (Data S1: Table S1.4; Fig. S1.34).

**Figure 19:**
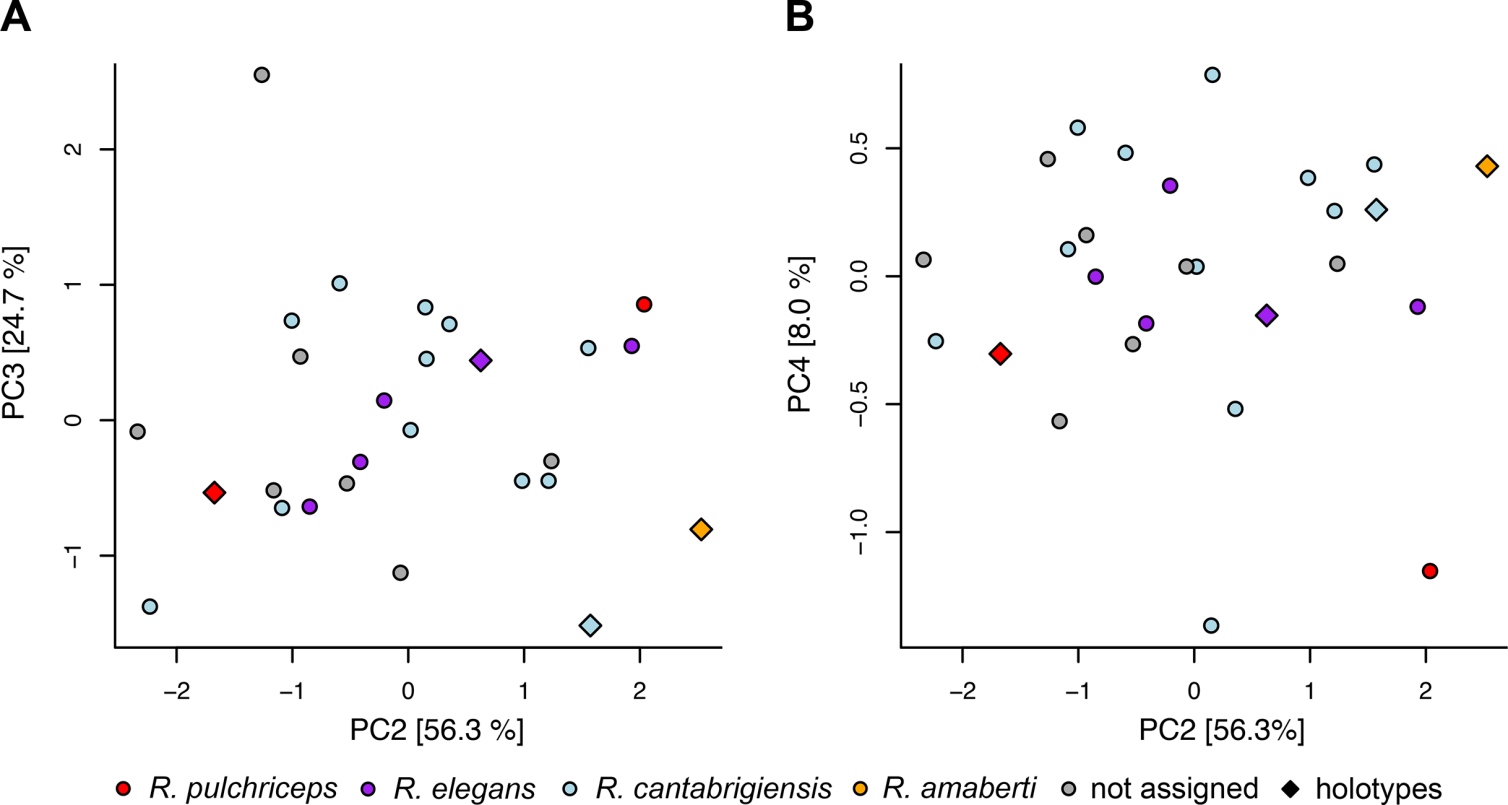
Distribution of specimens of *Rhinochelys* (*n* = 27) in cranial geometry morphospaces recovered by Principal Component Analysis of seven cranial measurements. (A) PC1 vs. PC2; (B) PC1 vs. PC3.

More than 90% of the variance can be explained by the first three principal components (PC1–3) (see Table 1; Fig. 19). PC1 explains 56.6% of the variance in the data and describes relative increases in skull size, jaw angle, and nasal length–width ratio, as the eigenvector coefficients all have the same sign (Table 1). PC1 therefore represents size and allometric shape changes. For the bivariate morphospace plots in Fig. 19, PC1 was excluded to only visualise non-size variation among the data. PC2 explains 56.3% of the shape variance (24.5% of total variance including PC1) and largely describes relative increases in the nasal length–width ratio with decreases in skull size. PC3 explains 24.7% of the shape variance (10.7% of the total variance) and is predominantly determined by relative increases in nasal length–width ratio and jaw angle with decreases in length measurements. PC4 explains 8% of the shape variance (5.4% of the total variance), and describes asymmetry between the right and left nasals, as well as a trend of decreasing skull height and length with increasing width.

**Table 1:** PCA results for our analysis using all measurements (*n* = 27).

	PC1	PC2	PC3	PC4	PC5	PC6	PC7
Eigenvalues	3.962	1.712	0.751	0.243	0.164	0.128	0.041
Proportion of total variance explained	0.566	0.245	0.107	0.035	0.023	0.018	0.006
Proportion of shape variance explained	–	0.563	0.247	0.08	0.054	0.042	0.014
Eigenvector coefficients							
Right nasal length–width ratio	−0.143	−0.686	−0.235	0.261	−0.422	0.453	0.050
Left nasal length–width ratio	−0.255	−0.606	−0.129	−0.446	0.438	−0.400	−0.011
Jaw angle	−0.245	0.318	−0.881	0.043	0.164	0.093	−0.160
Width	−0.460	−0.034	0.190	0.664	0.055	−0.386	−0.398
Orbital width	−0.477	0.117	0.145	0.180	0.355	0.250	0.719
Pre-parietal length	−0.455	0.118	0.307	−0.392	0.059	0.528	−0.498
Height	−0.456	0.178	−0.032	−0.322	−0.686	−0.368	0.222

Figures 19A and 19B shows that specimens do not form clusters but are seemingly randomly distributed in morphospace. The colouration of specimens according to the identification published by Collins (1970) indicates that specimens that were said to belong to different species are not separated from specimens of other species. Instead, the distribution of points on PC2–4 indicates a continuum of variation among Collins’ (1970) diagnostic characters and our new measurements. Furthermore, the holotype specimens are relatively close to one another in morphospace, and do not fall in extreme opposites of the morphospace as would be expected if strong and systematic morphological variation was present between them.

## Discussion

### Taxonomy of *Rhinochelys*

The type species of *Rhinochelys* was described by Owen (1851) as *Chelone pulchriceps* based on a skull in a phosphatic nodule from the Cambridge Greensand Member of the West Melbury Marly Chalk Formation (early Cenomanian, 100.5–93.9 Ma; Hopson, 2005), near Barnwell, Cambridgeshire (Owen, 1851). Seeley (1869) made *Chelone pulchriceps* the type species of a new genus, *Rhinochelys*, without giving specific reasons. However, he provided a diagnosis for the genus (Seeley, 1869: p. 25). Seeley (1869) also named 15 other species of *Rhinochelys* but did not provide descriptions for any of these species, so that they were soon considered to be invalid by Lydekker (1889: p. 229) and were later considered to be *nomina nuda* (e.g. Collins, 1970). Lydekker (1889) named five new species of *Rhinochelys* (*R. cantabrigiensis*, *R. macrorhina*, *R. elegans*, *R. brachyrhina*, *R. jessoni*), and provided specimen numbers, figures, and differential diagnoses for all of them. Half a century later, Moret (1935) coined another species, *R. amaberti*, based on a skull from the late Aptian–Cenomanian Marnes Bleues Formation near Villard-de-Lans in southeastern France, which is roughly contemporaneous with the Cambridge Greensand Member (the locality for the specimen is dated to be late Albian (113–100.5 Ma); Moret, 1935; Scavezzoni & Fischer, 2018).

Collins (1970) revised the British material of *Rhinochelys* based on specimens in the CAMSM and NHMUK. She recognised three morphotypes and accordingly only considered three previously named British species of *Rhinochelys* valid: *R. pulchriceps*, *R. elegans*, and *R. cantabrigiensis* (Collins, 1970). These species were distinguished from one another based on seven features, four of which were angles or ratios of skull measurements, and the rest were based on osteological features related to the snout and cranial scale sulci. Collins (1970) considered *R. amaberti* distinct from the British material based on slightly different skull ratios and a wider angle with which the maxillae diverge posterolaterally from the midline of the skull (jaw angle of Collins, 1970). Collins’ (1970) work represents a thorough description of the *Rhinochelys* material, but the preservation of most specimens in phosphatic nodules prevented detailed description of the internal anatomy. Collins’ (1970) braincase description was based on a specimen (CAMSM B94606) that is larger than all of the other specimens of *Rhinochelys* (Hooks, 1998). Other authors (Smith, 1989; Hooks, 1998) noticed this size discrepancy and Hooks (1998) provided some anatomical arguments (such as the large contributions of the vomer and palatine to the palate) to exclude this specimen from *Rhinochelys*. Collins (1970) referred *Rhinochelys* to Protostegidae based on comparative anatomical arguments such as the presence of nasal bones, the absence of a secondary palate and the contribution of frontals to the orbit. Since then most authors have agreed with this referral and later phylogenetic work provided further evidence for the protostegid affinities of *Rhinochelys* (Hirayama, 1994; Hooks, 1998; Cadena & Parham, 2015; Evers & Benson, 2019).

Collins (1970) tentatively assigned postcranial material of the species *Cimochelys benstedti*, which was also found in the Cambridge Greensand Member of the West Melbury Marly Chalk Formation, to *Rhinochelys* because of the protostegid affinities of the former, despite a lack of overlapping skeletal elements. Later authors largely followed this referral (Hirayama, 1997; Hooks, 1998; Tong et al., 2006). Hooks (1998) provided a systematic review of the Protostegidae. For his phylogenetic work, he used all *Rhinochelys* material (i.e. all four species considered valid by Collins, 1970) to score a monotypic *Rhinochelys* because the different species did not vary from one another in the characters he used. However, Hooks (1998) did not provide a formal synonymisation and stated that ‘it is clear that more work needs to be done on the systematics of *Rhinochelys*’ (Hooks, 1998: p. 87). Similarly, Hooks (1998) listed *Cimochelys benstedti* as a valid species in the systematic palaeontology section of his work, but none the less combined the hypodigms of *Cimochelys* and *Rhinochelys* in the production of his phylogeny.

Tong et al. (2006) described another species, *R. nammourensis*, from the middle Cenomanian of Lebanon. The new material presented by Tong et al. (2006) includes completely preserved specimens with skull–postcranium associations on slabs, and even soft tissue is preserved around the flippers. Several individuals were described, including hatchling, juvenile, and adult specimens. The material was referred to *Rhinochelys* based on the presence of large nasal bones and an anteriorly bulged maxilla-prefrontal region (Tong et al., 2006). The shell of *R. nammourensis* shows clear protostegid synapomorphies, such as ‘star-shaped’ plastral elements with strongly serrated margins. The shell of *R. nammourensis* differs from the *Cimochelys benstedti* material from England in the number of suprapygals and the successive posterior increase in width of the vertebral scutes (Tong et al., 2006). Tong et al. (2006) accepted the synonymy of *Cimochelys benstedti* with *Rhinochelys (pulchriceps)*, but presented only gross morphological observations, such as the carapacial outline and the size of fontanelles as evidence for this hypothesis.

Recently, Scavezzoni & Fischer (2018) reassessed the holotype material of *R. amaberti* and argued for the validity of this species based on a number of proposed autapomorphies related to the narial region (e.g. shape of the nasal bone and the external naris outline), the preorbital bulge and skull measurement ratios. However, many of their comparisons are based on accepting the taxonomy of Collins (1970) for British species of *Rhinochelys*. Scavezzoni & Fischer (2018) referred several previously unreported skulls from the Cambridge Greensand Member to ‘morphotypes’ that follow the taxonomy of Collins (1970). These specimens were then used, together with published information, to code the OTUs for phylogenetic analysis including the different *Rhinochelys* species. Characters relevant to the diagnoses of the proposed *Rhinochelys* species were included to achieve resolution within the dataset (Scavezzoni & Fischer, 2018). Scavezzoni & Fischer (2018) found *Rhinochelys* to be polyphyletic, whereby *R. amaberti*, *R. nammourensis*, and *R. pulchriceps* formed a clade that was the sister group to a clade including Late Cretaceous protostegids such as *Archelon ischyros* and *Protostega gigas*, whereas the *R. cantabrigiensis* and *R. elegans* morphotypes were found in a clade with Early Cretaceous taxa such as *Santanachelys gaffneyi*, *Bouliachelys suteri*, and *Desmatochelys padillai*. Scavezzoni & Fischer (2018) suggested that some specimens in their sample showed differences to all other morphotypes, and may thus represent an additional, unnamed species of *Rhinochelys*.

Our CT scans, and the resulting 3D models of individual skull bones, allow us to systematically assess variation among specimens that have been assigned to the species *R. pulchriceps*, *R. cantabrigiensis*, and *R. elegans*, including the holotypes. Although variation is present among the sample of specimens used for our work, we demonstrate that the apparent variation does not reflect the species delimitation proposed by Collins (1970). Furthermore, we do not find evidence for distinct morphotypes (i.e. clusters) in either morphometric or osteological features. The continuum of variation observed here suggests instead the presence of intraspecific variation of a single species. The holotype specimens of *R. elegans* and *R. cantabrigiensis* do not show unique autapomorphies or unique character combinations and are therefore considered subjective junior synonyms of *R. pulchriceps*. *R. amaberti* is also synonymised with *R. pulchriceps*, as the holotype of the former also lacks distinctive features (see *Status of proposed diagnostic characters of* Rhinochelys *species* for details). Therefore, we only consider the type species, *R. pulchriceps*, to be valid from among the proposed European species.

Nevertheless, the presence of morphologically distinguishable cranial, mandibular, and postcranial material in the collections of the CAMSM indicates a formerly cryptic diversity of sea turtles, and possibly protostegids, in the Cambridge Greensand Member of the West Melbury Marly Chalk Formation. Although we agree with previous authors that the holotype postcranium of *Cimochelys benstedti* has protostegid affinities, the lack of anatomical overlap with the holotype skull of *R. pulchriceps* prevents synonymisation of *Cimochelys benstedti* with *R. pulchriceps*, which we therefore reject provisionally. Future discoveries could resolve whether the holotype of *Cimochelys benstedti* is conspecific with *R. pulchriceps*, or with one of the other (unnamed) turtles of the Cambridge Greensand Member.

We accept *R. nammourensis* as a valid species because the preserved cranial material shows some differences to all specimens of *R. pulchriceps*. Namely, *R. nammourensis* has a relatively smaller frontal with an anteroposteriorly short lateral process that contributes to the orbit, and also a mediolaterally much narrower parietal (Tong et al., 2006). Whereas in *R. pulchriceps*, the parietal forms more than 50% of the width of the skull roof, most of the skull roof in *R. nammourensis* is formed by the postorbital. Additionally, *R. nammourensis* has elongate posterior processes of the squamosal (Tong et al., 2006), whereas the posterior surface of the squamosal in *R. pulchriceps* is rounded and lacks processes altogether (e.g. CAMSM B55791; see Data S1: Fig. S1.14). Furthermore, *R. nammourensis* has been found in deposits from the middle Cenomanian of Lebanon, whereas the youngest occurrence of *R. pulchriceps* is from the early Cenomanian of the UK. Therefore, *R. pulchriceps* is slightly older than *R. nammourensis*.

### Status of proposed diagnostic characters of *Rhinochelys* species

*Rhinochelys* is known from an exceptional number of skulls, many of which are largely complete and relatively well-preserved (at least 30 essentially complete skulls). Regardless of their taxonomic conclusions, all papers on the anatomy *Rhinochelys* have found a large amount of variation among specimens (Collins, 1970; Scavezzoni & Fischer, 2018; this study). This variation has been used to support the validity of four species (*R. pulchriceps*, *R. cantabrigiensis*, *R. elegans*, *R. amaberti*) from Europe alone (Collins, 1970; Scavezzoni & Fischer, 2018). Scavezzoni & Fischer (2018) even tentatively suggested the existence of a new, albeit unnamed, species. However, several authors questioned this high species diversity (e.g. Smith, 1989; Hirayama, 1997; Hooks, 1998) and have argued that the variation documented by Collins (1970) is insufficient to support her proposed taxonomy. Both Collins (1970) and Scavezzoni & Fischer (2018) used cranial measurements and proportions, as well as osteologically variable features, to support their taxonomic opinions. Here, we discuss and re-interpret previously proposed autapomorphies for the various European species of *Rhinochelys*.

#### Measurements of the holotype specimens

Our CT scans and derived 3D models allowed us to more accurately measure aspects of the morphology of *Rhinochelys*, because we could use digitally enlarged versions of the skulls and make our measurements explicitly in three-dimensions. The digital removal of matrix also allowed us to take some taphonomic artefacts into account. For the holotype specimens that we CT scanned, we find less variance than reported by Collins (1970). For example, the jaw angle of Collins (1970) was a central part of her proposed taxonomy. However, our measurements for the jaw angle differ considerably from those of Collins (1970), with values ranging from 49 to 57° for the holotype specimens of the European *Rhinochelys* species (Table 2), whereas the values measured by Collins (1970) range from 50 to 105°.

**Table 2:** Comparison of measurements taken by Collins (1970) and this study for holotype specimens of different *Rhinochelys* specimens.

Specimen number	Holotype	Jaw angle		X/Z*100		Y/Z*100	
		Collins (1970)	This study	Collins (1970)	This study	Collins (1970)	This study
CAMSM B55775	*R. pulchriceps*	72	49	107	150	51	88
NHMUK PV OR43980	*R. cantabrigiensis*	50	57	107	136	62	96
NHMUK PV R2226	*R. elegans*	57	48	96	133	57	118
UJF-ID.11167	*R. amaberti*	105	57	135	154	65	122

**Note:**

‘X’ denotes Collins’ (1970) measurement of the skull width; ‘Y’ denotes Collins’ (1970) measurements of the skull height; ‘Z’ denotes Collins’ (1970) measurement of pre-parietal skull length. Note that measurements of ‘Y’ used for this table were measured as originally done by Collins (1970), whereas this measurement was modified for the data used as input for the PCA.

We found similar discrepancies regarding the dorsoventral height of the skull relative to the anteroposterior length, and the mediolateral width relative to the anteroposterior length, ratios that were used by Collins (1970) to group specimens into categories of narrow or wide and deep or high skulls. Moreover, some measurements of Collins (1970) do not reflect the actual dimensions of the skulls when preservation is taken into account. For example, *R. cantabrigiensis* was said to have the relatively broadest skull of the three British species (Collins, 1970). However, our digital model of the *R. cantabrigiensis* holotype (NHMUK PV OR43980) shows that the specimen is partially disarticulated across the skull midline, artificially increasing the apparent width of the specimen (Fig. 1D; Data S1: Fig. S1.4C). Additionally, the postorbitals on both sides are not tightly articulated with the parietals and frontals and seem to have slipped laterally, further increasing the width. These gaps between the bones account for approx. 12% of the uncorrected postorbital width of the specimen. If this artificial widening is removed (as in the measurement reported in our Table 2), the holotype specimen of *R. cantabrigiensis* has similar proportions to other specimens of *Rhinochelys*.

Similar arguments can be made for the holotype and only specimen of *R. amaberti* (UJF-ID.11167): Scavezzoni & Fischer (2018) suggested that a ‘dorsoventrally compressed’ skull was an autapomorphic feature of *R. amaberti*. However, this specimen lacks most of the braincase, palate, and the descending processes of the parietal. Therefore, the central architecture for structural support of the skull is absent, resulting in little resistance to taphonomic dorsoventral compression. Indeed, the posterior view of the specimen (Scavezzoni & Fischer, 2018: fig. 3C) shows clearly that the skull is distorted, with the right quadrate and parietal/postorbital being quite strongly dorsoventrally compressed. Therefore, the low depth of *R. amaberti* can be explained by taphonomic compression and should not be regarded as supporting evidence for the validity of this taxon. This taphonomic compression also undermines two of the other autapomorphies proposed by Scavezzoni & Fischer (2018), namely the parallel outlines of the skull roof and the ventral margin of the maxilla and the mediolaterally broad external naris, which are both unlikely to reflect the original morphology. Furthermore, our PCA results (using only the measurements of Collins, 1970, as well as an extended measurement dataset) show no clusters that would support proportional differences between the specimens assigned to previously recognised species (see Results).

#### Osteological variation

Although proportional differences among proposed species of *Rhinochelys* are not supported by our study, Collins (1970), Scavezzoni & Fischer (2018) and ourselves noted variation in some osteological features that might support species-level distinctions. Collins (1970) only found unique osteological traits for *R. cantabrigiensis*, while *R. elegans* and *R. pulchriceps* were distinguished based solely on proportional differences. Proposed autapomorphies of *R. cantabrigiensis* were the presence of a ridge of the maxillary sulcus, a convex profile to the premaxillae and a hooked tip to the upper jaw (Collins, 1970). Our CT scans of the *R. cantabrigiensis* holotype (NHMUK PV OR43980) show that the anterior parts of the labial margins of both skull sides are broken in this specimen (see Figs. 1C and 1D; Data S1: Fig. 1.4D). This results in a strongly convex labial margin of the maxillae when seen in lateral view (Fig. 1C; Data S1: Figs. 1.4A and 1.4B), and also in a somewhat acute tip to the premaxillary labial margin. The strongly curved labial margins and the hooked tip of the upper jaw are the result of this breakage. We also investigated if referred specimens of *R. cantabrigiensis* preserve a hooked beak but found them to be absent in all specimens in which this part of the skull is well-preserved (e.g. CAMSM B55783; Figs. 4A and 4B).

When considering only adequately preserved specimens, we agree with Collins (1970) that the outline shape of the ventral margin of the maxilla is somewhat variable. This margin is relatively straight in lateral view in several *Rhinochelys* specimens (e.g. NHMUK PV OR35197), but moderately ventrally convex in others (e.g. CAMSM B55783). Despite this variation, breakage in the holotype specimens of *R. pulchriceps* and *R. cantabrigiensis* make a consistent association between the shape of the ventral margin of the maxilla and the species proposed by Collins (1970) impossible. Additionally, several authors have reported large amounts of intraspecific variation in the shape of the snout and triturating surface margin for several turtle species, including sea turtles (e.g. Dalrymple, 1977; Smith, 1989), which indicates that slight variation in the shape of the labial ridges is not well suited for species distinction by itself. We agree with Collins (1970) that the maxillary sulcus of NHMUK PV OR43980 is particularly deep, which gives the impression of a ‘ridge’ dorsal to the sulcus (Fig. 1C; Data S1: Figs. 1.4A and 1.4B). However, the sulci are very variably developed among all specimens of *Rhinochelys* (see below), and virtually absent in some of the specimens that Collins (1970) considered to belong to *R. cantabrigiensis* (CAMSM B55783; see Data S1: Fig. 1.4), so that we do not think that this feature is diagnostic for *R. cantabrigiensis*.

Most of the autapomorphies listed by Scavezzoni & Fischer (2018) for *R. amaberti* are dubious, because they can be explained by dorsoventral compression of the holotype specimen, or because they are not unique to *R. amaberti*. For example, the antorbital bulge of the maxilla and prefrontal is said to be more prominent in *R. amaberti* than in other species of *Rhinochelys*, and its visibility in ventral view is listed as a proxy for this (Scavezzoni & Fischer, 2018). However, the antorbital bulge is visible in ventral view in almost all specimens of *Rhinochelys* (e.g. Figs. 1D, 1F, 3A and 3B; Data S1: Figs. S1.2D, S1.4D, S1.6D, S1.8D and S1.9D), and its presence is generally considered apomorphic for the genus (e.g. Collins, 1970; Tong et al., 2006). Another proposed autapomorphy of *R. amaberti*, a mediolaterally broad external naris (with a horizontal long axis), is also present in other specimens, notably in the holotype of *R. pulchriceps* (CAMSM B55775; see Data S1: Fig. S1.2E), and thus cannot be an autapomorphy for *R. amaberti*. We agree with Scavezzoni & Fischer (2018) that the nasal of *R. amaberti* is extremely short anteroposteriorly and broad mediolaterally. However, our nasal length–width ratio measurements show that there is great variation in the nasal shape of *Rhinochelys*, and our PCA including nasal shape does not distinguish *R. amaberti* significantly from other *Rhinochelys* specimens. The mediolaterally short contact between the nasals and the frontals of *R. amaberti* is indeed shorter than in most other specimens of *Rhinochelys*. However, UK specimens of *Rhinochelys* also show high levels of variation in this aspect of nasal morphology, so this feature does not provide a reliable diagnosis of *R. amaberti*. In summary, we do not consider the osteological evidence presented by either Collins (1970) or Scavezzoni & Fischer (2018) to unambiguously support the existence of multiple European species of *Rhinochelys*.

### Osteological variation found in this study

Osteological features that varied across the *Rhinochelys* specimens that we CT scanned are mentioned above (see Description), but to facilitate taxonomic discussion they are listed here and shown in Table 3. The most distinct variation that we observed in our sample of *Rhinochelys* occurs in the dermatocranium. Besides the nasal shape, which has already been discussed, variation in the dermatocranium is found in the configuration of the jugal, the shape of the fissura ethmoidalis, in the presence vs. absence of a foramen antrum postoticum (indicating the internal presence of an antrum postoticum) and in the otic trochlear process. Variation is also evident in the braincase elements, namely the exoccipitals (presence vs. absence of a posterior ridge), the basioccipital (presence vs. absence of a crista dorsalis basioccipitalis) and the position of the foramen posterius canalis carotici interni (fpcci) either between the pterygoid and parabasisphenoid, or only within the pterygoid. The epidermal sulci of *Rhinochelys* are also variably developed. In most specimens, a distinct maxillary sulcus is present between the body of the maxilla and the ascending process. This sulcus pronounces the antorbital bulge of the maxilla and prefrontal that is characteristic of all *Rhinochelys* specimens, but is only faintly developed or absent in some specimens. Similarly, the sulcus present on the frontals is variably developed.

**Table 3:** Osteological variation found across specimens of *Rhinochelys* that were examined using CT data.

Specimen number	Holotype	Taxonomy (*sensu* Collins (1970))	Osteological feature								
			Foramen antrum postoticum	Bulk contribution to otic trochlear process	Fissura ethmoidalis	Posterior jugal process	Exoccipital ridge	Fpcci formation	Crista dorsalis basioccipitalis	Maxillary sulcus	Frontal sulcus
CAMSM B55775	*R. pulchriceps*	*R. pulchriceps*	Absent	Quadrate	V-shaped	NA	Present	Pterygoid+parabasispehnoid	Absent	Deep	Deep
NHMUK PV R2226	*R. elegans*	*R. elegans*	Absent	Prootic	Constricted	Present	NA	NA	NA	Moderate	Faint
NHMUK PV OR43980	*R. cantabrigiensis*	*R. cantabrigiensis*	Absent	Prootic	V-shaped	Present	NA	Pterygoid+parabasispehnoid	Absent	Deep	Absent
CAMSM B55783	-	*R. cantabrigiensis*	Present	Prootic	Constricted	Present	Absent	Pterygoid+parabasispehnoid	NA	Absent	Absent
CAMSM B55776	*R. stenicephalus*	*R. elegans*	Present	Prootic	V-shaped	Present	NA	Pterygoid	NA	Moderate	Moderate
NHMUK PV OR35197	-	*R. elegans*	Present	Prootic	V-shaped	Absent	NA	Pterygoid+parabasispehnoid	Present	Faint	Absent

**Note:**

Note that ‘fpcci’ means foramen posterius canalis carotici interni.

#### Taxonomic interpretation

In most cases, the osteological variation documented herein does not correspond with the possible species proposed for *Rhinochelys* (Table 3). Most of these features are either present in several of the holotype specimens for *R. pulchriceps*, *R. cantabrigiensis*, and *R. elegans*, or present in a holotype specimen but not in any of the other specimens that were referred to that species by Collins (1970). Also, there is no obvious pattern of covariation between the variable traits, so that this variation does not support an alternative taxonomic division of *Rhinochelys*. The only possible exceptions are two features that are present exclusively in the *R. pulchriceps* holotype (CAMSM B55775), namely the presence of an exoccipital ridge (see Fig. 14) and the enlarged quadrate contribution to the processus trochlearis oticum (see Fig. 11). Both of these features are best interpreted as related to soft tissue attachments. The position of the exoccipital ridge along the occipital surface of the skull makes it likely that it served as an attachment site for soft tissue structures, such as axial musculature (Werneburg, 2011). Similarly, the otic trochlear process of turtles is related to musculature, as it guides the jaw adductor muscles from their origins on the supratemporal surfaces to their insertions on the lower jaw. Ossification occurs at places with increased loading of respective bone surfaces (e.g. Goodship, Lanyon & Mcfie, 1979; Lanyon et al., 1982) and hypertrophied musculature-related osteological features have been reported for turtles before (Dalrymple, 1977). Therefore, rather than interpreting these features as autapomorphic for *R. pulchriceps*, we hypothesise that they represent individual variation. Due to the absence of any systematic distribution for this osteological variation, we argue for the presence of a single European species with relatively high intraspecific variation, *R. pulchriceps*. This is supported by an assessment of intraspecific variation reported for other turtles, as explained below.

### Intraspecific variation in *R. pulchriceps*

We argue that the observed variation among *R. pulchriceps* specimens does not support the hypothesis of multiple European species, but that this trait variability can be explained by intraspecific variation. Although it is possible that the assemblage is time-averaged, the lack of more detailed stratigraphic provenance of specimens does not permit testing for possible anagenetic changes or the distinction of morphotypes into chronospecies. Intraspecific variation can be the result of geographic variation across different populations, sexual dimorphism, ontogenetic changes or ‘true’ intraspecific variation (i.e. polymorphic traits that cannot be explained by the former categories). Since all UK *Rhinochelys* specimens come from the same region, the existing variation patterns cannot be explained by biogeography. Sexually dimorphic traits are known among modern turtles, including chelonioids specifically, and usually include size variation between females and males (male sea turtles being smaller; Godley et al., 2002), and the presence of longer tails and larger claws in the front flippers of males (Wibbels, Owens & Rostal, 1991; Wibbels, 1999; Wyneken, 2001; Casale et al., 2005). Cranial osteological features are not known to be sexually dimorphic in sea turtles, however, and as a result we do not interpret any of the observed osteological variation of *R. pulchriceps* as sexual dimorphism (although we note that our sample of CT-scanned specimens is too small to test for sexual dimorphism statistically).

Most specimens of *R. pulchriceps* are small (<35 mm total skull length) and of similar size, but the largest skulls are approximately twice as large as the smallest, so we can investigate if some of the externally visible variable features (i.e. those that can be found without CT scanning specimens) could be explained by ontogeny. Our initial hypothesis for the variability of skull sulci was that the depth of the sulci increases ontogenetically. However, the depth of the skull sulci is variable on both large and small skulls, so that we do not consider this feature to be under ontogenetic control. Consequentially, all variable features observed are interpreted to be ‘true’ intraspecific variation. We surveyed the literature to see if the variation of *R. pulchriceps* is comparable with the amount of intraspecific variation documented for other turtles. Only a few studies have focussed on ‘true’ intraspecific osteological variation while considering or controlling for geographic variation, ontogenetic changes or sexual dimorphism in turtles (e.g. Dalrymple, 1977; Bever, 2009). These studies provide important proxies for the extent of intraspecific variation that cannot be explained by sexual dimorphism or ontogenetic changes. The most notable results of Dalrymple’s (1977) study of variation of cranial features in the soft-shelled turtle *Apalone ferox* were that the majority of skull shape variation was related to the feeding mechanism, including variation in the size and shape of the triturating surface, the size and shape of the supraoccipital crest, the size of the supratemporal passages for the jaw adductor musculature, and the size of the otic trochlear process that redirects this musculature. Furthermore, the variable traits identified by Dalrymple (1977) did not covary, so that the presence of one feature did not necessitate the presence of another. These results are consistent with the hypothesis that much of the variation in *R. pulchriceps* (e.g. variation in the curvature of labial margins) can be the result of individual variation. Bever (2009) found that of the 200 discrete characters used in his study on the variation of a single population of the emydid turtle *Pseudemys texana*, which were collected from a lake over the duration of four years, over 50% exhibited some level of variation. Intraspecific variation that could not be explained by ontogeny or sex included changes in the shape of bones, changes in ratios of skull measurements, the participation of certain bones to structures in the cranium such as skull openings, and the shape of sutures between bones. The studies of Dalrymple (1977) and Bever (2009) demonstrate that, given a large enough sample and control for ontogeny, sexual dimorphism and geography, turtles exhibit a range of cranial variations that are comparable to the variation observed for *R. pulchriceps*, which itself represents a fossil taxon for which an unusual number of well-preserved specimens is available. This supports our hypothesis that only one European species, *R. pulchriceps*, can be considered valid.

### Diversity of turtles from the Cambridge Greensand Member of the West Melbury Marly Chalk Formation

**Figure 20:**
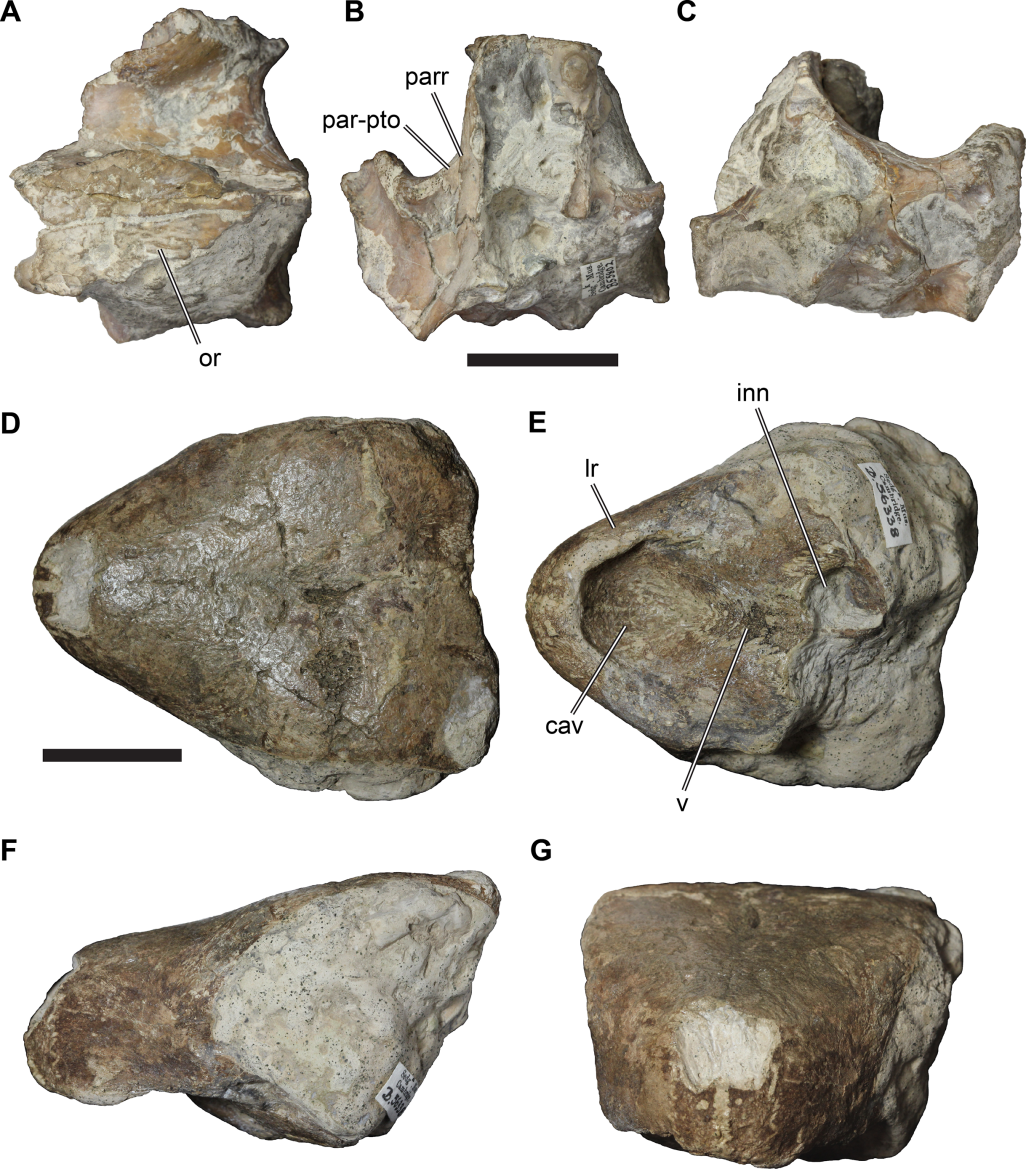
Cranial specimens from the Cambridge Greensand Member of the West Melbury Marly Chalk Formation that cannot be referred to *Rhinochelys*. (A) CAMSM B55802 in dorsal view; (B) CAMSM B55802 in anteroventral view; (C) CAMSM B55802 in posterior view; (D) CAMSM B56338 in dorsal view; (E) CAMSM B56338 in ventral view; (F) CAMSM B56338 in left lateral view; (G) CAMSM B56338 in anterior view. Scale bars equal 20 mm. Abbreviations: *cav*, cavity on the anterior surface of the triturating surface; *inn*, internal naris; *lr*, labial ridge; *par-pto*, parietal contribution to the processus trochlearis oticum; *parr*, parietal ridge; *v*, vomer.

**Figure 21:**
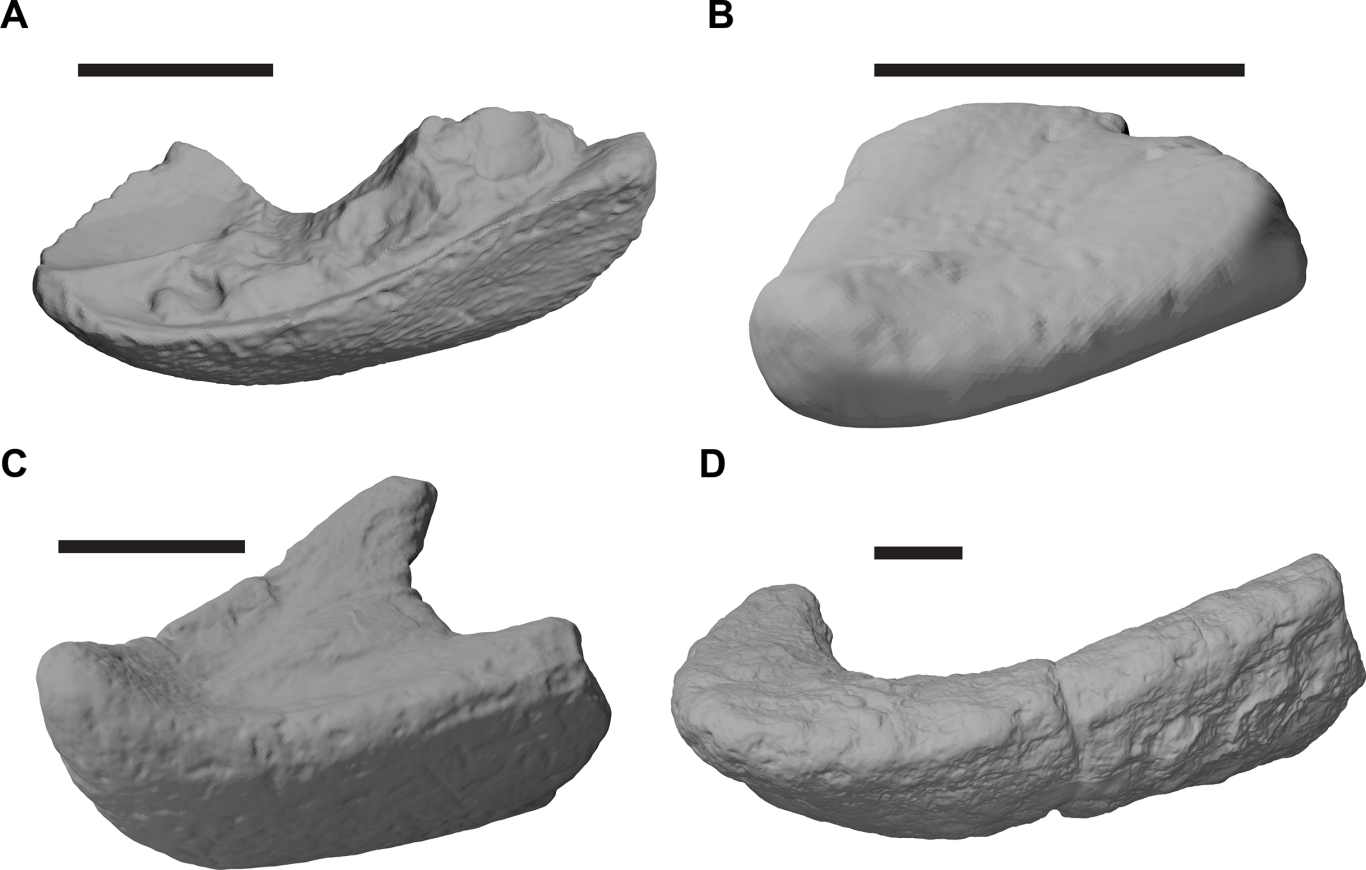
Comparisons of mandibular morphotypes from the Cambridge Greensand Member of the West Melbury Marly Chalk Formation. (A) *Rhinochelys pulchriceps* morphotype, 3D rendering of partial mandible of CAMSM B59560 in right anterodorsolateral view, reflected for comparison; (B) morphotype 1, 3D rendering of partial mandible of CAMSM B55848 in left anterodorsolateral view; (C) morphotype 2, 3D rendering of partial mandible of CAMSM B55860 in left anterodorsolateral view; (D) morphotype 3, 3D rendering of partial mandible of CAMSM B56586 in left anterodorsolateral view. Scale bars equal 20 mm.

**Figure 22:**
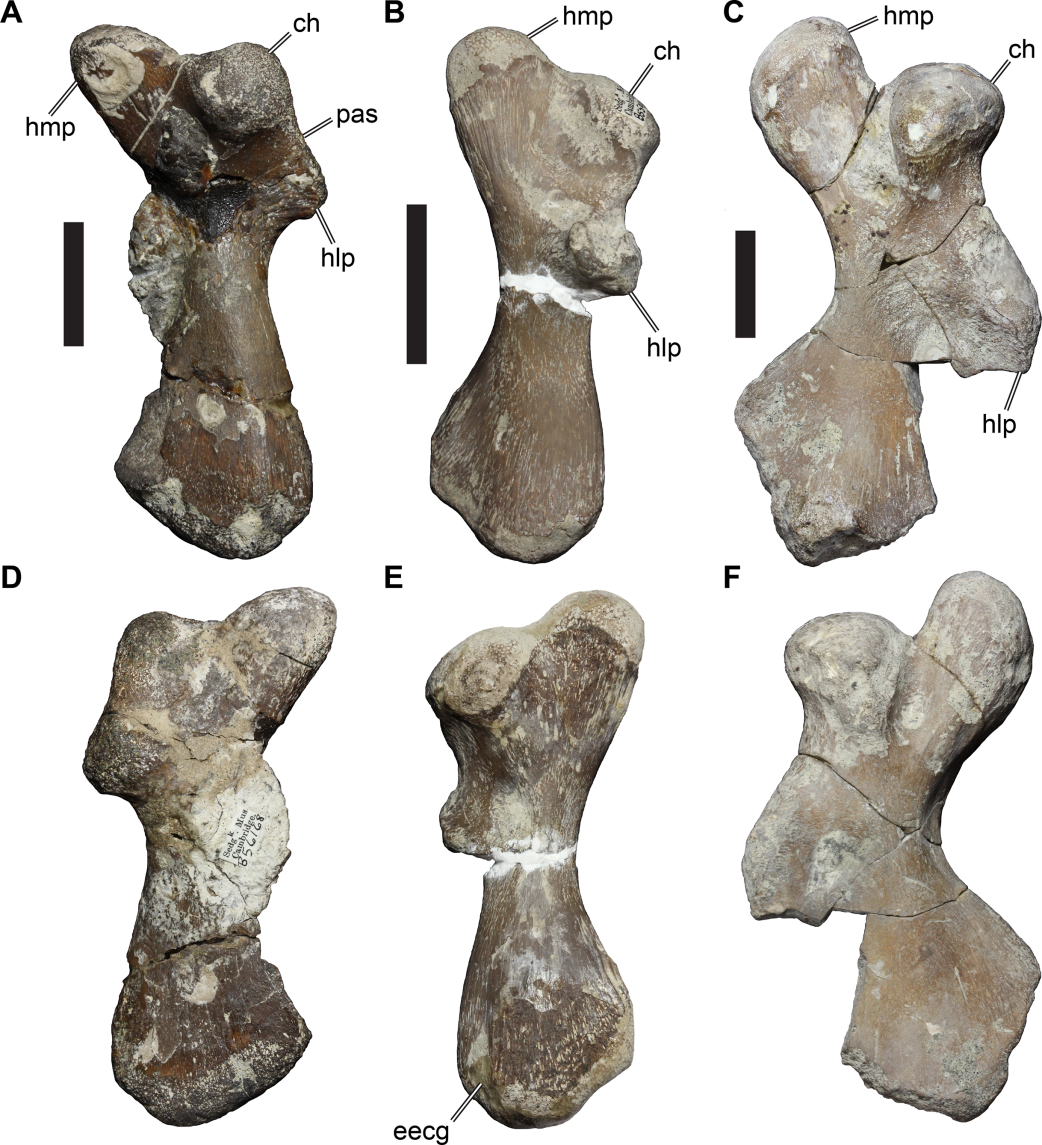
Comparisons of humeri morphotypes from the Cambridge Greensand Member of the West Melbury Marly Chalk Formation. (A) morphotype 1, left humerus of CAMSM B56168 in ventral view; (B) morphotype 2, left humerus of CAMSM B55987 in ventral view; (C) morphotype 3, left humerus of CAMSM B55988 in ventral view; (D) as in (A), but dorsal view; (E) as in (B), but dorsal view; (F) as (C), but dorsal view. Scale bars equal 20 mm. Abbreviations: *ch*, caput humeri; *eecg*, ectepicondylar groove; *hlp*, lateral process of humerus; *hmp*, medial process of humerus; *pas*, preaxial shoulder.

Turtle specimens from the Cambridge Greensand Member of the West Melbury Marly Chalk Formation indicate the presence of several distinct morphotypes among cranial remains that are distinct from *R. pulchriceps* and additional diversity among mandibular and postcranial materials. These include three cranial specimens (Fig. 20) and three distinct mandible morphotypes that are documented by a number of specimens (Fig. 21) in addition to those referred to *R. pulchriceps*. At least three humerus morphotypes are present (Fig. 22). Although the systematic identity of most of these specimens is unclear, and we do not provide a detailed taxonomic treatment, some can be assigned to the total group of Chelonioidea. The presence of total-group chelonioid material distinct from *R. pulchriceps* is important, as it prevents any firm assignment of carapaces and plastra currently referred to as *Cimochelys benstedti* (e.g. NHMUK PV OR39112, OR47210). While specimens of *R. pulchriceps* dominate the assemblage in terms of cranial material found, postcranial material seems to rarer, and does not show the same pattern of a dominant morphotype, making even tentative assignments of postcranial material to *R. pulchriceps* impossible.

#### Distinct braincase morphotype

CAMSM B55802 is a partial braincase (Figs. 20A–20C). The specimen is relatively large (estimated 55 mm skull width), and therefore larger than specimens of *R. pulchriceps*. CAMSM B55802 shows some osteological differences from all other specimens referred to *R. pulchriceps*. These are: (1) a clearly sculptured parietal dorsal surface; (2) a short lateral expansion of the parietal that contributes to the processus trochlearis oticum; (3) a strongly dorsally concave processus trochlearis oticum; and (4) a strongly developed ridge on the lateral surface of the descending process of the parietal, which is posteroventrally continuous with a ridge on the pterygoid, and which forms an anteroventrally facing trough on the anterior margin of the descending process of the parietal. The skull roof ornamentation of CAMSM B55802 is absent in all known chelonioids. The ornamentation of CAMSM B55802 consists of low, sinuous ridges, which differs from the tubercular ornamentation of solemydid turtles like *Helochelydra nopcsai*, which is known from the Early Cretaceous of the UK (Joyce et al., 2011). The ornamentation of CAMSM B55802 is more similar to that of *Pleurosternon bullockii* (UMCZ T1041; Evans & Kemp, 1975), which is also known from the Early Cretaceous of the UK, but also to carettochelyids such as *Allaeochelys libyca* (BSPG 191 II 130; Havlik, Joyce & Böhme, 2014) and *Carettochelys insculpta* (NHMUK 1903.7.10.1). All of these taxa have low ridges of various lengths on their parietals.

Additionally, following the anatomical considerations of Hooks (1998), we do not consider the braincase specimen CAMSM B94606 that was used for the braincase description presented by Collins (1970) to belong to *R. pulchriceps*. Unfortunately, we could not examine this specimen first-hand, because it is on long-term loan (M. Riley, 2018, personal communication).

#### Distinct rostral morphotype

CAMSM B56338 is a partially preserved skull (Figs. 20D–20G) referred to ‘*Trachydermochelys*’ on the specimen label. This skull shows numerous differences from *R. pulchriceps*, the most important being: (1) the presence of a secondary palate with a long vomerine contribution to the triturating surface; (2) the presence of massive, rounded labial ridges of the maxillae; (3) the presence of a deep medial cavity on the triturating surface, possibly indicating the presence of a mandibular hook; (4) the presence of medial processes of the jugal which contacts palatal elements; and (5) the presence of a posteriorly extended skull roof with no temporal emarginations. These features suggest that CAMSM B56338 is a total-group chelonioid, because this combination of features is unknown outside of the total group of Chelonioidea. However, CAMSM B56338 is probably not a protostegid due to the presence of a secondary palate and a medial jugal process, which is absent in all protostegids.

#### Mandibular morphotypes

The Cambridge Greensand Member assemblage includes many isolated mandibles that can be categorised into several morphotypes (mandibular morphotypes 1–3 herein), in addition to those with the morphology of *R. pulchriceps* described above (e.g. isolated mandibles CAMSM B55809, B55810, B55819, B56590, B56593; Fig. 21A). Isolated mandibles of *R. pulchriceps* occupy the same range of sizes as those of crania, with a maximum preserved dentary length <35 mm.

Mandibles of morphotype 1 (e.g. CAMSM B55836, B55847; Fig. 21B) are characterised by a relatively acute ‘mandibular angle’ of 45–50°, have an elongate and flat triturating surface, extremely low and broadly rounded labial margins, a low depth of the dentary, and a rounded anterior margin with a very low symphyseal hook. They differ from *R. pulchriceps* in showing no evidence of a symphyseal ridge and in the absence of well-defined labial ridges.

Mandibles of morphotype 2 (e.g. CAMSM B55860, B55854, B56587, B56589, B76722; Fig. 21C) are characterised by a strongly acute jaw angle of ∼35°, have an elongate triturating surface with a low but broad ridge that is limited to the posterior two–thirds of the triturating surface, the absence of labial ridges, a deep lateral surface of the dentary, and an upturned anterior end of the symphysis that forms a well-developed hook. Mandibles of this morphotype are generally large (e.g. 80 mm long in CAMSM B55860). This morphotype is similar to large protostegids such as *Archelon ischyros* (Wieland, 1900) and *Protostega gigas* (FMNH PR 2; FMNH P 27385) although the mandibular hook is much weaker in those taxa. Scavezzoni & Fischer (2018) described an isolated partial mandible that they assigned to *R. amaberti* (using the holotype specimen number, UJF-ID.11167, although this mandible was collected several years later than the cranium). This mandible is referable to our morphotype 2, as it has a small symphyseal hook and a weakly developed median ridge on the posterior end of the triturating surface (Scavezzoni & Fischer, 2018).

Mandibles of morphotype 3 (e.g. CAMSM B56301, B56586, B56592; Fig. 21D) have a relatively wide jaw angle >60°, an anteroposteriorly short triturating surface, a low labial ridge, and moderately deep rami of the dentary, which become mediolaterally narrower at their posterior end. Mandibles of morphotype 3 are much larger than the mandible of *R. pulchriceps*: despite their comparatively short triturating surface, the length of this surface exceeds the absolute size of *R. pulchriceps* mandibles.

#### Humerus morphotypes

A high diversity of chelonioid humeri from the Cambridge Greensand Member has been noted before (e.g. Hirayama, 1994). We recognise at least three distinct morphotypes. All humeri that we found in the collections of the CAMSM and NHMUK belong to total-group chelonioids, as the lateral process is positioned relatively far distally on the humeral shaft (a synapomorphy of total-group Chelonioidea; Zangerl, 1953b; Gaffney & Meylan, 1988; Hirayama, 1994). In humerus morphotype 1 (e.g. CAMSM B56167, CAMSM B56168; Figs. 22A and 22D), the humerus shaft is moderately flattened, and the distal end of the humerus is broadened. The caput humeri is rounded and positioned on the proximal surface of the humerus, being separated from the medial process by a shallow notch. A short preaxial shoulder on the anterior side of the proximal surface leads from the caput humeri to the lateral process, which is in a proximal positioned on the humeral shaft. An ectepicondylar foramen is not evident. Morphotype 1 is similar to the humeri of some cheloniids (e.g. *Eochelone brabantica*: Hirayama, 1994), but also some protostegids (e.g. *Chelosphargis advena*: Hirayama, 1994).

In humerus morphotype 2 (e.g. CAMSM B55987; Figs. 22B and 22E), the humeral shaft is more flattened than in morphotype 1 and the medial process is broader and extends further medially than in morphotype 1. A preaxial shoulder is absent, and the lateral process is a robust, knob-like structure that is further distally placed on the humeral shaft than in morphotype 1, but still in the proximal half of the humerus. A shallow ectepicondylar groove is apparent in some specimens. This humerus morphotype is similar in most shape aspects to AMNH FARB 1975, which has been referred to *Chelosphargis advena* (e.g. Hirayama, 1994, who referred to CAMSM B55987 as an ‘aberrant chelonioid’). However, in AMNH FARB 1975 the lateral process is pointed and roughly triangular, while the process is more massive in humerus morphotype 2.

Humerus morphotype 3 (e.g. CAMSM B55988; Figs. 22C and 22F) is distinctly different from morphotypes 1 and 2. Hirayama (1994) classified humeri from the Cambridge Greensand Member into comparative categories and referred CAMSM B55988 to ‘*Protostega’ anglica*. The specimens belonging to morphotype 3 are larger in absolute size than humeri of the other morphotypes and the humerus is strongly flattened along the shaft and the distal end. The medial process of the humerus is separated from the caput humeri by a deep notch and projects further proximally in regard to the caput humeri than in morphotypes 1–2. This is different in most specimens of large protostegids, such as *Protostega gigas* (FMNH P27482; FMNH UR 80; but similar to AMNH FARB 180) or *Archelon ischyros* (Wieland, 1896), which possess only a shallow notch between the caput and medial process. The lateral process is positioned on the mid-point of the anterior surface of the humerus shaft and is, therefore, in a more distal position than the lateral process of *Archelon ischyros* and *Protostega gigas*. This is similar to dermochelyids, in which the lateral process is associated with a prominent crest that extends onto the dorsal surface of the humerus shaft, which is absent in CAMSM B55988. The distal margin of the lateral process is damaged by breakage in CAMSM B55988, so that it is unclear if the process forms a tapered and distally directed tip as in dermochelyids.

In summary, the Cambridge Greensand Member of the West Melbury Marly Chalk Formation provides evidence for a minimum of four taxa in the assemblage (*R. pulchriceps* and at least three others based on mandibles). Associations between mandibles, humeri, and skulls are unknown, but it is possible that some of the reported material pertaining to different body parts belong to the same taxa. The assemblage is largely composed of total-group chelonioids, although the partial braincase CAMSM B55802 could not be identified taxonomically and might represent a non-chelonioid taxon. It is currently unclear if any of the reported specimens belong to taxa on the stem- or crown-group of Chelonioidea. In any case, the assemblage indicates a higher total-group chelonioid diversity than previously recognised, which prevents referring postcranial material to *R. pulchriceps* until associated material is found.

### Calibrated tree and stratigraphic congruence

**Figure 23:**
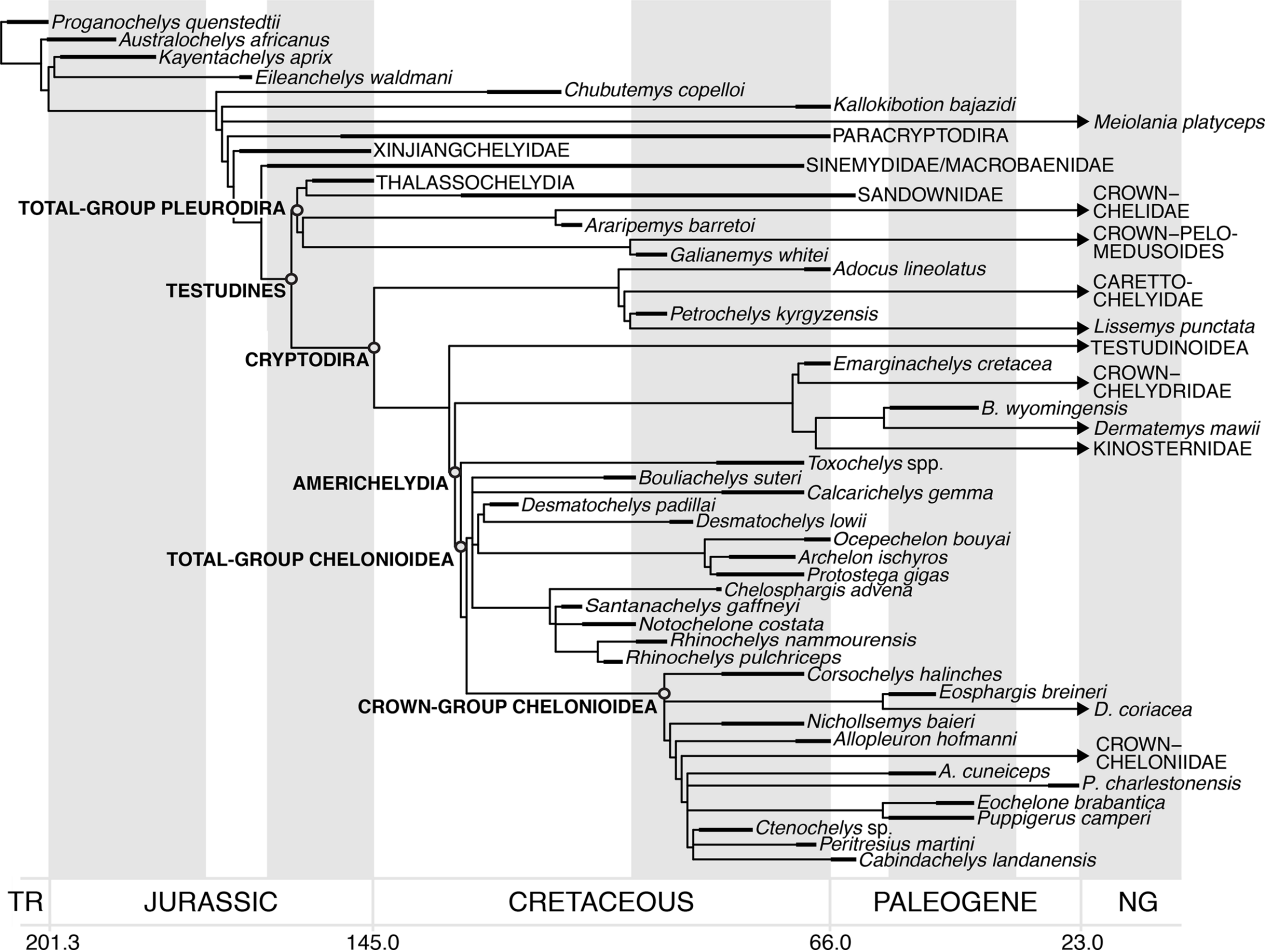
Simplified reduced strict consensus tree scaled to geologic time using range data for turtles.

Our recovery of protostegids as stem-group chelonioids positioned one node more crownward than *Toxochelys* sp. reduces ghost lineages for total-group cheloniids and dermochelyids in comparison to those implied by the alternative hypothesis of protostegids being the sister group to dermochelyids (see Cadena & Parham, 2015; Evers & Benson, 2019). This argument was recently made by Raselli (2018), who also found protostegids to be stem-group chelonioids. Previous studies have suggested origination times for the chelonioid crown-group around the Cretaceous/Palaeocene boundary (66.18 Ma; e.g. Joyce et al., 2013), and the error bars for the node age estimate for Chelonioidea by Joyce et al. (2013) extend into the Santonian. These estimates are clearly much younger than the oldest protostegids (Aptian, 120 Ma; Cadena & Parham, 2015), so finding protostegids to be outside of the chelonioid crown-group helps resolve that discrepancy. However, despite this apparent increase in stratigraphic congruence, the new placement for protostegids found in this study still implies long ghost lineages for crown-group chelonioids (c. 35 Ma), *Toxochelys* sp. (c. 45 Ma) and chelydroids (c. 60 Ma; Fig. 23; see also Evers & Benson, 2019).

Several recent studies have presented evidence that some mid-Late and Late Cretaceous fossils previously interpreted to be stem-group chelonioids can also be placed within the chelonioid crown-group (e.g. *Ctenochelys* sp.: Gentry, 2016; *Peritresius martini*: Gentry et al., 2018; *Allopleuron hofmanni*: Evers & Benson, 2019). This new information suggests that crown-group chelonioids might be older than typically inferred by node-dating methods such as employed by Joyce et al. (2013), or at least towards the older end of error bars on the node age estimates provided by those studies. If these Cretaceous taxa do indeed belong to the crown-group of Chelonioidea, then they provide minimum age constraints for the chelonioid crown-group that extend back to at least the Coniacian (88.8 Ma; Gentry et al., 2018; Fig. 23), based on the earliest occurrence of *Ctenochelys* sp. (Gentry et al., 2018). However, given the number of recent papers presenting re-interpretations of early chelonioid fossils and novel phylogenetic findings (Gentry, 2016; Evers & Benson, 2019; Gentry et al., 2018), it is clear that we are still far from a phylogenetic consensus regarding stem- and crown-group membership of fossil chelonioids. For now, therefore these older taxa described do not yet qualify as reliable calibration points according to the protocol of best practices presented by Parham et al. (2012) and used by Joyce et al. (2013). An alternative method that should be explored are analyses that estimate divergence times and phylogenies simultaneously and incorporate phylogenetic uncertainty, such as tip-dating (Pyron, 2011; Ronquist et al., 2012).

The Early Cretaceous age of protostegids requires some long ghost lineages within Americhelydia irrespective of their exact position within this clade. Although the earliest fossil evidence for chelydroids, crown-group chelonioids (e.g. *Ctenochelys* sp.) and the earliest branching stem-group chelonioid (*Toxochelys* sp.) all come from North America, it is conspicuous that North American americhelydian fossils, including protostegids such as *Calcarichelys gemma*, *Desmatochelys lowii*, *Archelon ischyros*, and *Protostega gigas*, only appear from the Coniacian (Late Cretaceous) onwards. Americhelydians most likely have a North American origin (e.g. Joyce et al., 2013; Crawford et al., 2015; Parham, Otero & Suárez, 2014; Cadena & Parham, 2015; Gentry, 2016; Pereira et al., 2017; Evers & Benson, 2019), but the oldest americhelydian fossils are protostegids from South America (*Desmatochelys padillai*: Cadena & Parham, 2015; *Santanachelys gaffneyi*: Hirayama, 1998), Europe (*R. pulchriceps*: Collins, 1970) and Australia (*Bouliachelys suteri*: Kear & Lee, 2006). The absence of Early Cretaceous americhelydians from North America can likely be explained by the limited exposure of terrestrial rocks from that time in this region (Evers & Benson, 2019). Therefore, the stratigraphic incongruence implied by chelonioid affinities for protostegids is possibly a discovery artefact.

### Evolution of flippers

**Figure 24:**
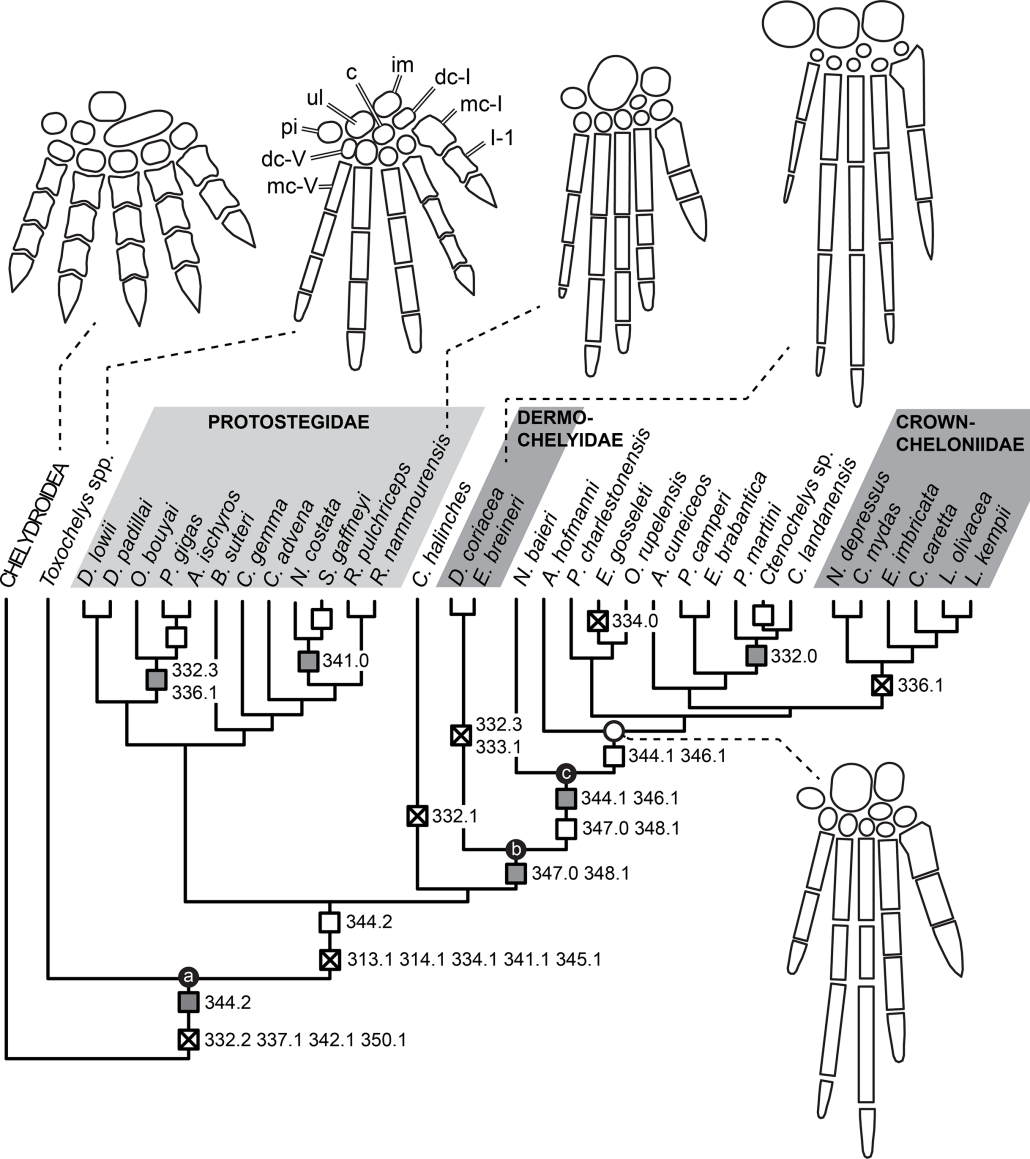
Simplified topology of chelonioids with character state transitions mapped onto the tree. The topology is the one used for character optimisation and represents one MPT from the original analysis that is consistent with the topology of a 50% majority rule consensus tree. Boxes with crosses represent unambiguous character transitions, open boxes represent DELTRAN, and shaded boxes represent ACCTRAN. Numbers above boxes represent character numbers, numbers below boxes show the apomorphic state. For plesiomorphic states, please refer to Table 4. Line drawings represent schematic flipper morphology for specific OTUs (top row), and the generalised flipper morphology of early cheloniids (bottom right). Note that Roman letters denote digits and Arabic numbers denote phalangeal position. Abbreviations: *c*, centrale; *dc*, distal carpal; *im*, intermedium; *mc*, metacarpal; *pi*, pisiform; *ulc*, ulnare.

The evolution of chelonioid flippers has been discussed in several studies (Hirayama, 1992, 1998; Kear & Lee, 2006; Joyce, 2007; Wieland, 1902), with different authors arriving at different conclusions regarding the homology vs. convergence of the flipper features observed in protostegids, dermochelyids, and cheloniids. Our expanded chelonioid taxon sampling and revision of characters specifically related to the marine ecology of chelonioids allowed us to re-interpret chelonioid flipper evolution in the context of our tree topology. We identified characters related to the pectoral girdle and forelimb that are related to the modification of chelonioid arms and hands into flippers. These characters encode structural modifications of the scapula (character 313), muscle attachment sites (characters 332, 336), relative lengths of bones (characters 314, 337, 334–336, 348), flattening of bones (character 350), flipper reinforcement by means of articular surface modification from ginglymoid joints to butt joints (characters 334, 341–342) and the phalangeal formula (character 347). These features are summarised in Table 4 and were mapped onto the tree topology that was used during the optimisation process (Fig. 24).

**Table 4:** Character evolution of traits relevant to the formation of flippers across the total group of Chelonioidea.

Character	Plesiomorphic state	Apomorphic state	Transition at node	Optimisation
313; internal angle of scapula	0	1	Protostegidae+++	Unambiguous
314; relative coracoid–humerus length	0	1	Protostegidae+++	Unambiguous
332; position of lateral humerus process	0	2	Total-group Chelonioidea	Unambiguous
	2	1	*Corsochelys halinches*	Unambiguous
	2	3	Dermochelyidae	Unambiguous
	2	3	*Ocepechelon* ++ *Protostega*	ACCTRAN
			*Archelon* + *Protostega*	DELTRAN
	2	0	*Peritresius* ++ *Ctenochelys*	ACCTRAN
			*Ctenochelys*	DELTRAN
333; anterior projection of lateral humeral process	0	1	Dermochelyidae	Unambiguous
334; distal humerus trochlea	0	1	Protostegidae+++	Unambiguous
	1	0	*Erquelinnesia*	Unambiguous
336; *M. latissimus* dorsi muscle scar	0	1	Crown-group Cheloniidae	Unambiguous
	0	1	*Ocepechelon* ++ *Protostega*	ACCTRAN
			*Archelon* + *Protostega*	DELTRAN
337; relative humerus–femur length	0	1	Total-group Chelonioidea	Unambiguous
341; rigid 1st–2nd digit articulations	0	1	Protostegidae+++	Unambiguous
	1	0	*Santanachelys* + *Notochelone*	ACCTRAN
			*Santanachelys*	DELTRAN
342; rigid 3rd–5th digit articulations	0	1	Total-group Chelonioidea	Unambiguous
344; relative ulnare–intermedium size	1	2	Total-group Chelonioidea	ACCTRAN
			Protostegidae+++	DELTRAN
	2	1	Total-group Cheloniidae	ACCTRAN
			*Allopleuron*+++	DELTRAN
345; relative proximal–distal carpal size	0	1	Protostegidae+++	Unambiguous
346; relative lengths of manual phalanges	0	1	Total-group Cheloniidae	ACCTRAN
			*Allopleuron*+++	DELTRAN
347; presence of 3rd phalanx on 5th digit	1	0	Crown-group Chelonioidea	ACCTRAN
			Total-group Cheloniidae	DELTRAN
348; longest manual digit	0	1	Crown-group Chelonioidea	ACCTRAN
			Total-group Cheloniidae	DELTRAN
350; flattening of carpals	0	1	Total-group Chelonioidea	Unambiguous

**Note:**

Note that ‘Taxon+++’ denotes a clade including that taxon and all more crownward positioned taxa, and that ‘Taxon A ++ Taxon B’ denotes the most inclusive clade including taxa A and B.

Our tree topology and character optimisation imply step-wise acquisition of the traits that characterise flippers. Many flipper character states are unambiguously optimised to appear at the base of the total-group chelonioid tree (Fig. 24) and are either shared among all total-group chelonioids, or are shared between protostegids and crown-group chelonioids, but are absent in *Toxochelys* sp., the earliest branching stem-group chelonioid. The traits that evolved earliest are: a shift in the position of the lateral process of humerus so that it is removed from the humeral head (332.2); a humerus that is elongated with respect to the femur (337.1); reduction of ginglymoid interphalangeal joints in the 3rd–5th manual digits (342.1); and a flattening of tarsal and carpal elements (350.1). One node more crownward, and thus shared by all total-group chelonioids but *Toxochelys* sp., our optimisation implies: the evolution of a wide internal angle of the scapula (313.1); relative elongation of the coracoid (314.1); reduction of the trochlea on the distal end of the humerus for the antebranchium (334.1); reduction of ginglymoid interphalangeal joints in the 1st and 2nd manual digits (341.1); and relative enlargement of proximal carpals with regard to distal carpals (345.1). Furthermore, the relatively large ulnare (344.2) evolves either at the base of total-group Chelonioidea (ACCTRAN) or in Protostegidae + more crownwardly positioned chelonioids (DELTRAN). This pattern of character evolution suggests that some fundamental biomechanical features typical for flippers in general (e.g. flattening of elements, lengthening of the humerus and reduction of mobility between individual flipper elements) as well as traits likely important for moving the flippers effectively (e.g. lateral humeral process position and internal scapula angle) evolved first.

It has been hypothesised that many of these features evolved convergently in at least protostegids and dermochelyids + cheloniids, but possibly in all three groups (e.g. Hirayama, 1998; Kear & Lee, 2006). However, hypotheses about these character state transitions are sensitive to the phylogenetic positions of individual taxa. For example, the recovery of *Toxochelys* sp., which lacks many of the derived flipper traits of other chelonioids (e.g. rigid interphalangeal articulations between the 1st and 2nd manual digits), as a stem-group cheloniid in Hirayama (1994, 1998) resulted in the optimisation of absence of these traits as plesiomorphic within cheloniids. The recovery of the Early Cretaceous protostegid *Santanachelys gaffneyi* on the stem-group of *Dermochelys coriacea* in the same analysis implied independent evolutionary transitions to a fully rigid flipper in cheloniids and dermochelyids (as *Santanachelys gaffneyi* has the same 1st and 2nd digit articulation pattern observed for *Toxochelys* sp.; Hirayama, 1998). *Toxochelys* sp. has been unambiguously accepted as a stem-group chelonioid in later studies and we find *Santanachelys gaffneyi* well-nested within Protostegidae (see also Evers & Benson, 2019). Our results therefore imply a single origin of fully rigid flippers but require a reversal within protostegids to accommodate *Santanachelys gaffneyi*. It is noteworthy that *Santanachelys gaffneyi* has only been described in a relatively short paper, which does not illustrate the flipper morphology in detail (Hirayama, 1998). To further elucidate some aspects of flipper evolution, the flippers of *Santanachelys gaffneyi* should be re-investigated, but this was beyond the scope of our study.

Our optimisation also suggests that the fundamental flipper bauplan outlined by the character state changes above was later modified in different ways by different groups of chelonioids (Fig. 24). For instance, the position of the lateral process of the humerus was independently shifted further distally (332.3) in dermochelyids and some protostegids (DELTRAN: *Archelon ischyros* + *Protostega gigas*; ACCTRAN: *Ocepechelon bouyai* ++ *Archelon ischyros*). Similarly, the muscle scar for the *M. latissimus dorsi* shifted distally on the humerus shaft (336.1) in crown-group cheloniids and convergently in *Archelon ischyros* + *Protostega gigas* (DELTRAN; ACCTRAN: *Ocepechelon bouyai* ++ *Archelon ischyros*).

A number of flipper traits appear first early in the evolution of cheloniids (Fig. 24). For example, the absence of a 3rd phalanx on the 5th manual digit (347.0) and the relative elongation of the 3rd digit with regard to the 4th (348.1) are optimised at the node of total-group cheloniids under DELTRAN, but could have evolved in crown-group chelonioids (ACCTRAN). Within total-group cheloniids, the second phalanges become elongated relative to the first phalanges (346.1) sometime during the early evolution of the group (ACCTRAN: *Nichollsemys baieri*+++; DELTRAN: *Allopleuron hofmanni*+++). Similar to the pattern observed for cheloniids, flipper modifications specific to dermochelyids appear in the early evolution of the group: the only fossil dermochelyid included in our study, *Eosphargis breineri* from the Ypresian of Denmark, already shows the mid-shaft position of the lateral humeral process (332.3) and the anterior projections of said lateral process (333.1), which is otherwise only seen in the extant leatherback *Dermochelys coriacea* (Fig. 24).

In summary, forelimb modifications related to the formation of flippers are concentrated at deep nodes and can thus be inferred to have happened early in the evolution of total-group chelonioids. Further modifications specific to cheloniids and dermochelyids are also observed early in the respective records of stem taxa. Protostegids generally retain the flipper morphology that evolved early among chelonioids more generally, as no flipper character state changes are observed along the lineage leading to the deeply nested *R. nammourensis*, which preserves complete flippers (Tong et al., 2006). However, some modifications that are optimised as being convergent with cheloniids (336.1; see above) or dermochelyids (332.3; see above) are observed in the gigantic taxa from the Late Cretaceous (*Archelon ischyros* and *Protostega gigas*). The lack of detailed knowledge of flipper morphology outside of these well-known protostegids currently precludes a more detailed investigation of the protostegid flipper.

## Conclusions

Our study represents the most detailed anatomical treatment of the cranial morphology of any protostegid to date. Besides providing digital models for six specimens of *Rhinochelys*, including the holotypes of the three British species considered valid by Collins (1970), we also evaluated other specimens to revise the taxonomy of the genus. Although anatomical variation was observed among specimens, qualitative assessment of the variable traits, as well as multivariate statistical analysis of skull measurements of a broad sample of specimens does not support the occurrence of clusters that could form the basis for objective species delimitation. Therefore, previously named species of *Rhinochelys* are synonymised, and only the type species, *R. pulchriceps*, is considered valid among the European species. Nevertheless, our assessment of non-cranial remains from the West Melbury Marly Chalk Formation indicates that several species of chelonioids are present in addition to *R. pulchriceps*. This prevents referral of protostegid postcranial bones from the assemblage to *R. pulchriceps* with any certainty.

We modified the recently published character-taxon matrix of Evers & Benson (2019) to specifically include more chelonioid taxa. Additionally, we revised the postcranial characters used in Evers & Benson (2019), based on new observations regarding body parts that are particularly modified in chelonioids, such as the forelimbs and the shell. The characters were also vetted to ensure that they adequately reflect primary hypotheses of homology. Analysis of this dataset removes protostegids from the chelonioid crown-group and places them onto the stem of chelonioids. This results from the absence of several synapomorphies of more crownwardly positioned chelonioids in protostegids.

The position of protostegids as stem-group chelonioids implies shorter ghost lineages for crown-group chelonioids, but still requires long ghost lineages for deeper divergences of americhelydians. Our ingroup relationships of protostegids are different to those reported in most previous studies: Rather than finding Early Cretaceous taxa as a paraphyletic grade leading to gigantic forms from the Late Cretaceous, we find two clades of protostegids, which largely correspond to Early and Late Cretaceous taxa. Although protostegids are removed from the crown-group of chelonioids, we find other evidence for the presence of crown-group chelonioids in the Late Cretaceous. For example, *Ctenochelys* sp., *Nichollsemys baieri*, *Allopleuron hofmanni*, *Peritresius martini*, and possibly others are found as stem-group cheloniids.

Our tree topology implies that most traits fundamental to modern chelonioid flippers evolved only once, early on the chelonioid stem-lineage. However, secondary modifications to the general flipper bauplan are observed at the base of crown-cheloniids and dermochelyids, as well as in the clade delimiting the gigantic protostegids *Archelon ischyros* and *Protostega gigas*. Although instances of convergent evolution between the chelonioid subgroups are apparent for individual characters, the flipper morphology of extant sea turtles is the result of a stepwise acquisition of derived characters that are concentrated at deep nodes in their phylogeny.

## Supplemental Information

10.7717/peerj.6811/supp-1 Supplemental Information 1 Supplementary text and figures. This document contains additional anatomical illustrations, character optimisations, and additioncal PCA.

10.7717/peerj.6811/supp-2 Supplemental Information 2 Character-taxon matrix. This files is the character-taxon matrix used for the phylogenetic analysis (nexus format).

10.7717/peerj.6811/supp-3 Supplemental Information 3 Character list. This document is the full character list and also includes by-character optimisations.

10.7717/peerj.6811/supp-4 Supplemental Information 4 Calibration ages. This file contains the age data used for the tree calibration, as well as sources for the data.

## Institutional abbreviations

AMNH: American Museum of Natural History, New York City, NY, USA
BSPG: Bayerische Staatsammlung für Paläontologies und Geologie, Munich, Germany
CAMSM: Sedgwick Museum of Earth Sciences, Cambridge, UK
FMNH: Field Museum of Natural History, Chicago, IL, USA
IRSNB: Royal Belgian Institute of Natural Sciences, Brussels, Belgium
KUVP: University of Kansas, Lawrence, KS, USA
NHMUK: Natural History Museum, London, UK
SMNS: Staatliches Museum für Naturkunde, Stuttgart, Germany
TMP: Royal Tyrell Museum, Drumheller, AB, Canada
UMZC: University Museum of Zoology, Cambridge, UK

## Additional Information and Declarations

### Data Availability

The following information was supplied regarding data availability: Data for this study (including CT volume and mesh files for segmented models) are available in MorphoSource: http://www.morphosource.org/Detail/ProjectDetail/Show/project_id/581 (Media objects M29973, M29983, M29984, M29986, M29987) and https://www.morphosource.org/Detail/MediaDetail/Show/media_id/22140 (Media object M221409). Additional data (supplementary text and figures, character-taxon matrix, character list, calibration ages) are available in the Supplemental Files.
