# Supplementary material for: Anatomy of Rhinochelys pulchriceps (Protostegidae) and marine adaptation during the early evolution of chelonioids
Source: PeerJ. 2019 May 1;7:e6811. doi: 10.7717/peerj.6811 (PMC6500378; doi:10.7717/peerj.6811)
Supplement: Supplemental Information 1 — This document contains additional anatomical illustrations, character optimisations, and additioncal PCA. [file peerj-07-6811-s001.pdf]

APPENDIX S1 to Evers, Barrett & Benson 2018: Anatomy of *Rhinochelys pulchriceps* (Protostegidae) and marine adaptation during the early evolution of chelonioids

Table of Contents

ADDITIONAL CT DATA INFORMATION	p. 2
ADDITIONAL ANATOMICAL ILLUSTRATIONS	p. 3
CHARACTER MODIFICATIONS	p. 24
SCORING SOURCES	p. 46
CHARACTER OPTIMIZATION	p. 51
Methods	p. 51
Results	p. 52
PCA DATA	p. 80
PCA USING MEASUREMENTS OF COLLINS (1970)	p. 83
Methods	p. 83
Results	p. 83
INSTITUTIONAL ABBREVIATIONS	p. 85
REFERENCES	p. 86

ADDITIONAL CT DATA INFORMATION

TABLE S1.1. Information about *Rhinochelys* specimens that were CT scanned for this study.

Specimen number	Holotype	Taxonomy (sensu Collins [1970])	Scanning facility	CT Scanner	Voxel size (mm)	Data availability	Reference
CAMSM B55775	*R. pulchriceps*	*R. pulchriceps*	NHMUK Imaging and Analysis Center	Nikon XT H 225 ST	0.0355	MorphoSource Media Group M29973	This study
NHMUK PV R2226	*R. elegans*	*R. elegans*	NHMUK Imaging and Analysis Center	Nikon XT H 225 ST	0.0351	MorphoSource Media Group M29987	This study
NHMUK PV OR43980	*R. cantabrigiensis*	*R. cantabrigiensis*	NHMUK Imaging and Analysis Center	Nikon XT H 225 ST	0.025	MorphoSource Media Group M29986	This study
CAMSM B55783	-	*R. cantabrigiensis*	NHMUK Imaging and Analysis Center	Nikon XT H 225 ST	0.0204	MorphoSource Media Group M22140	Evers & Benson (2018)
CAMSM B55776	-	*R. elegans*	NHMUK Imaging and Analysis Center	Nikon XT H 225 ST	0.0282	MorphoSource Media Group M29983	This study
NHMUK PV OR35197	-	*R. elegans*	NHMUK Imaging and Analysis Center	Nikon XT H 225 ST	0.0171	MorphoSource Media Group M29984	This study

ADDITIONAL ANATOMICAL ILLUSTRATIONS

The following illustrations are provided as additional guides for the description provided in the main text of this paper.

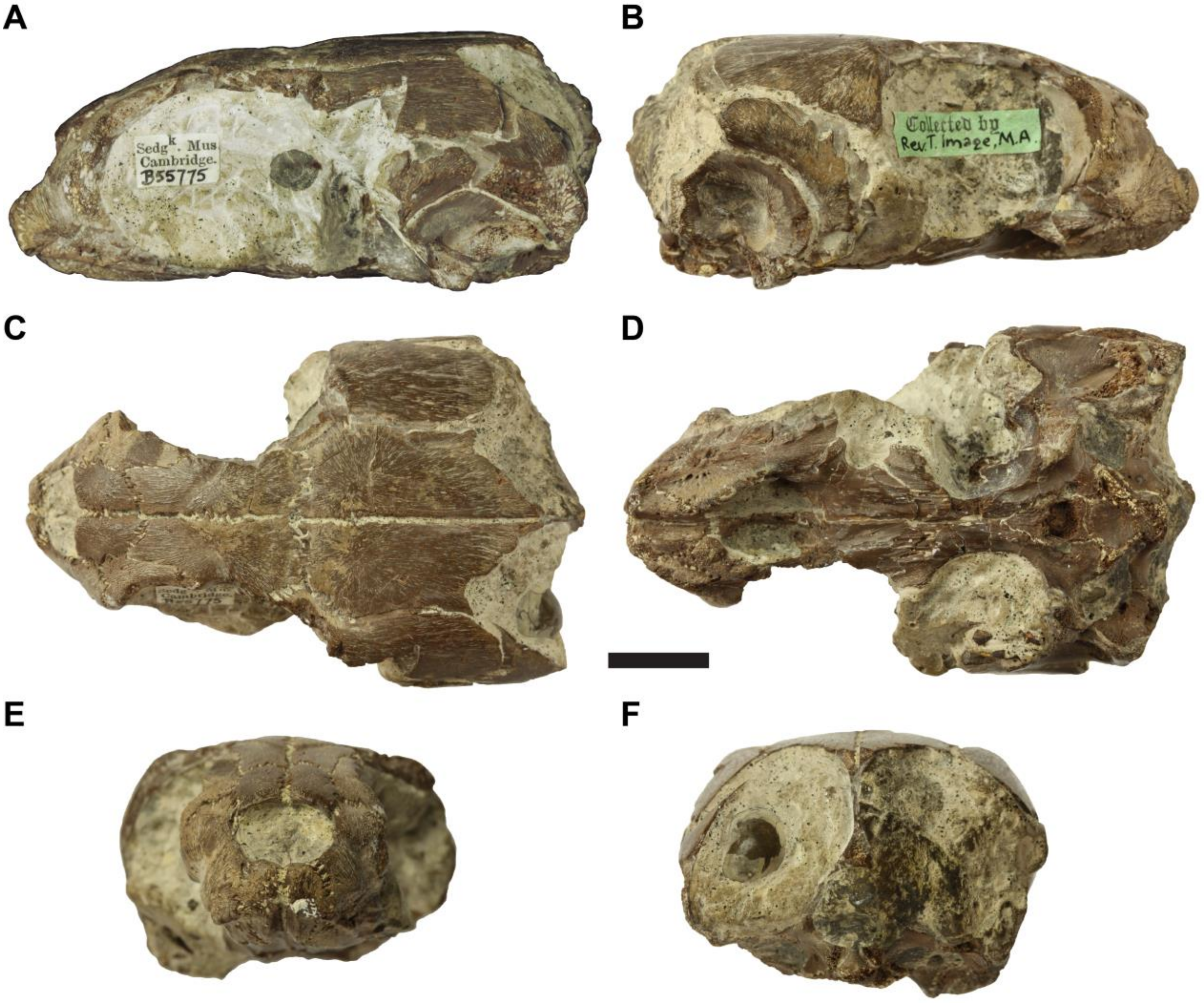

FIG. S1.1. Photographs of CAMSM B55775, the holotype of *R. pulchriceps*. **A**, left lateral view; **B**, right lateral view; **C**, dorsal view; **D**, ventral view; **E**, anterior view; **F**, posterior view. Scale bar equals 10 mm.

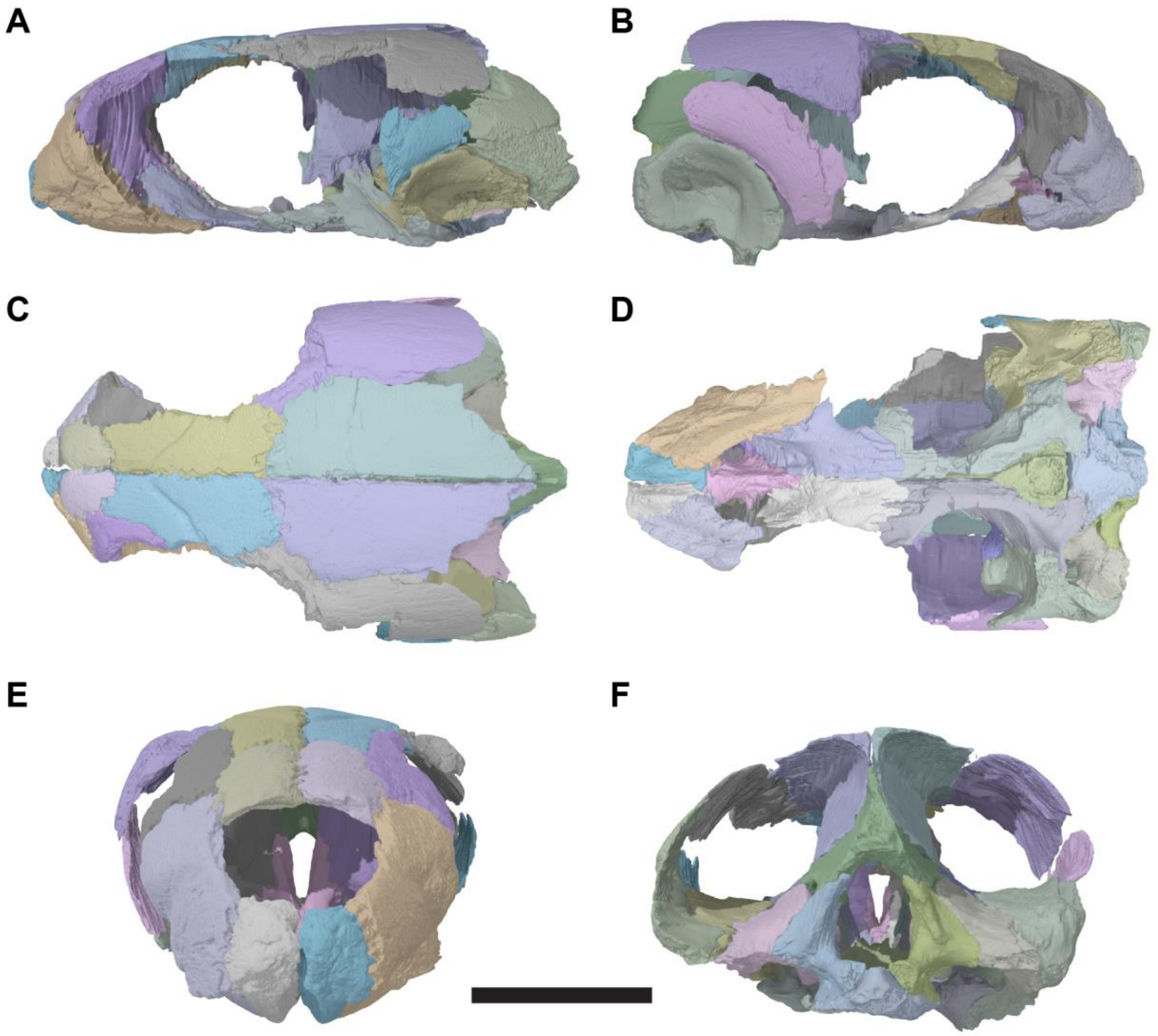

FIG. S1.2. 3D renderings of CAMSM B55775, the holotype of *Rhinochelys pulchriceps*. **A**, left lateral view; **B**, right lateral view; **C**, dorsal view; **D**, ventral view; **E**, anterior view; **F**, posterior view. Scale bar equals 20 mm.

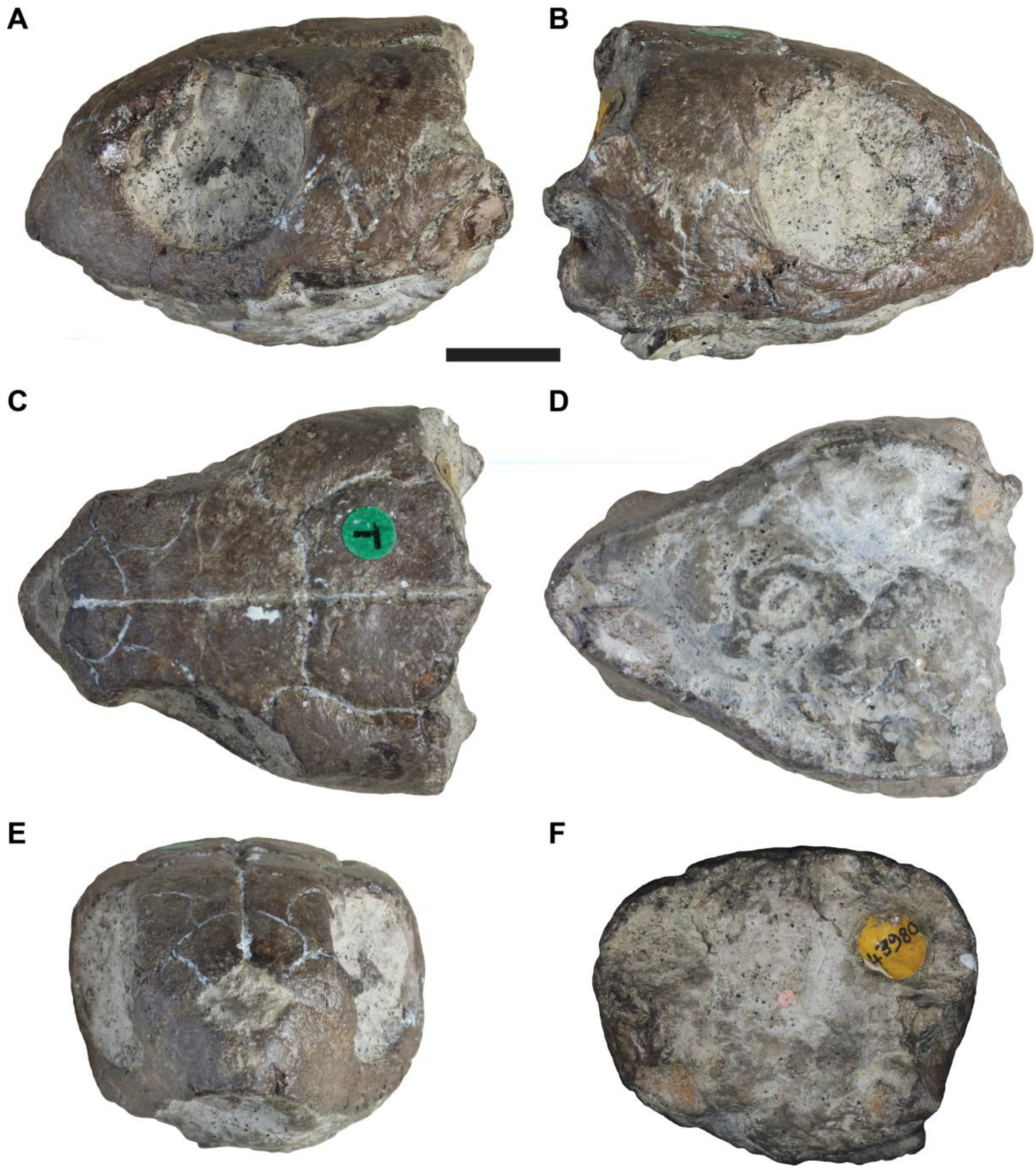

FIG. S1.3. Photographs of NHMUK 43980, the holotype of *R. cantabrigiensis*. **A**, left lateral view; **B**, right lateral view; **C**, dorsal view; **D**, ventral view; **E**, anterior view; **F**, posterior view. Scale bar equals 10 mm.

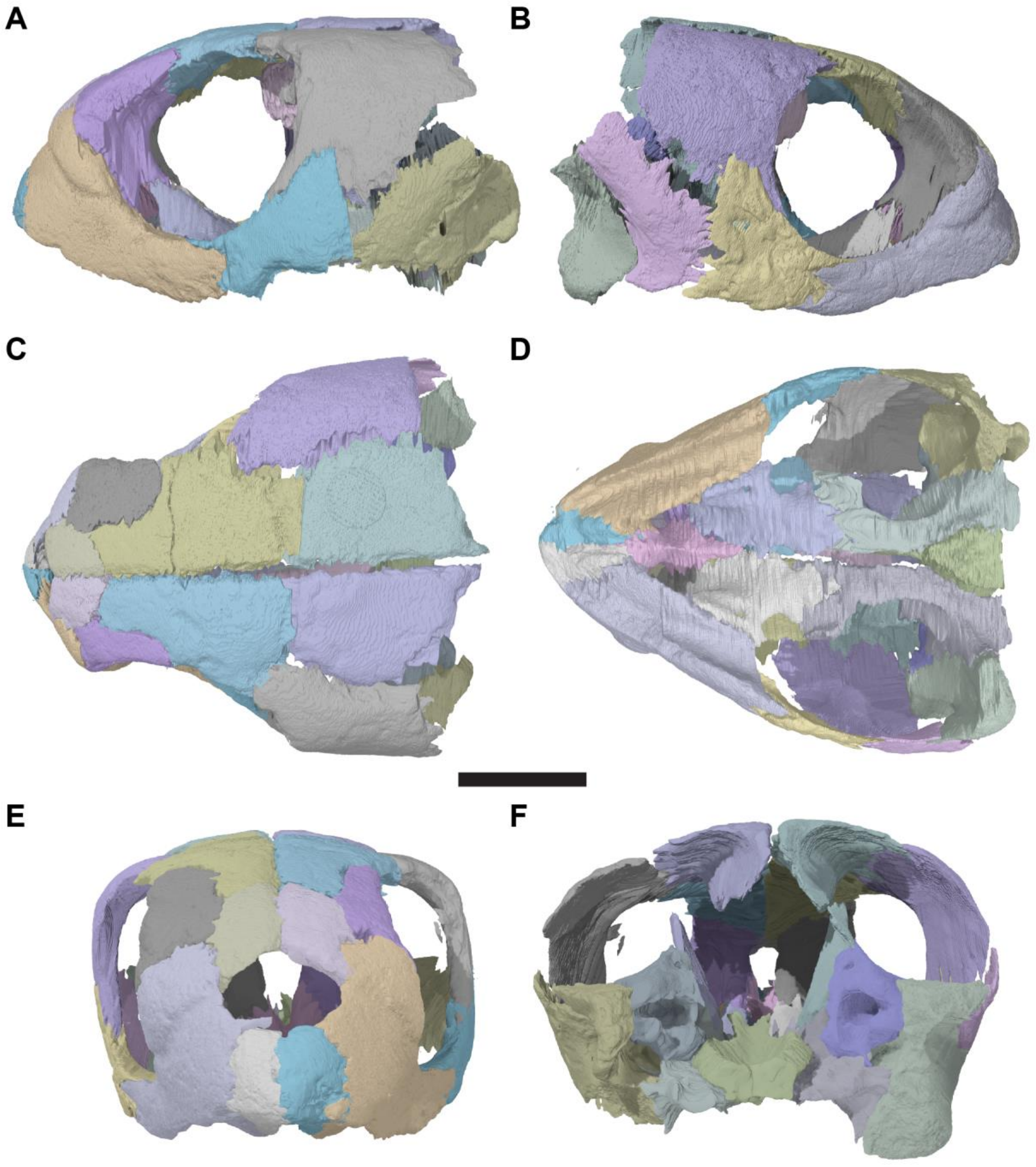

FIG. S1.4. 3D renderings of NHMUK PV OR43980, the holotype of *R. cantabrigiensis*. **A**, left lateral view; **B**, right lateral view; **C**, dorsal view; **D**, ventral view; **E**, anterior view; **F**, posterior view. Scale bar equals 10 mm.

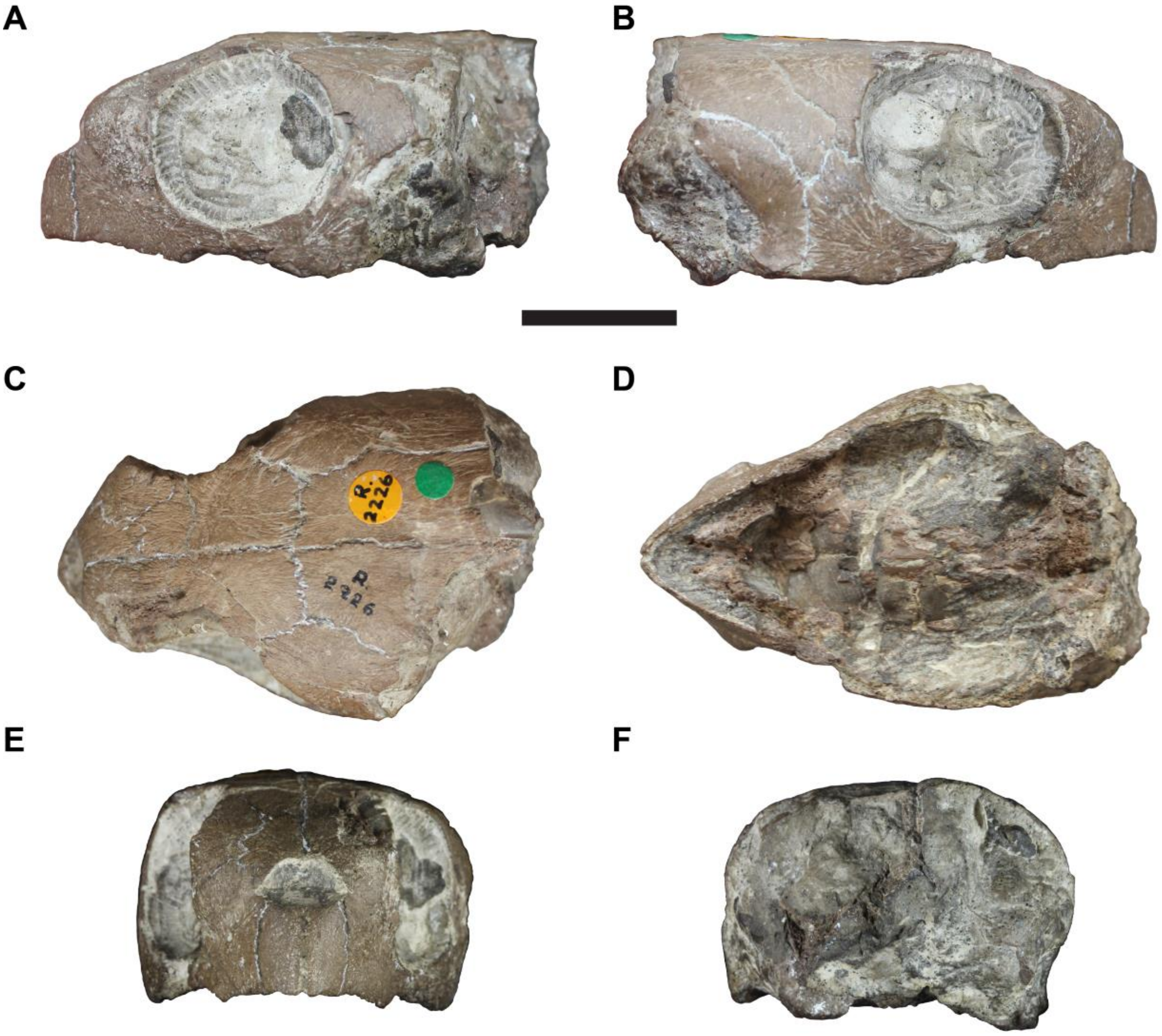

FIG. S1.5. Photographs of NHMUK 2226, the holotype of *R. elegans*. **A**, left lateral view; **B**, right lateral view; **C**, dorsal view; **D**, ventral view; **E**, anterior view; **F**, posterior view. Scale bar equals 20 mm.

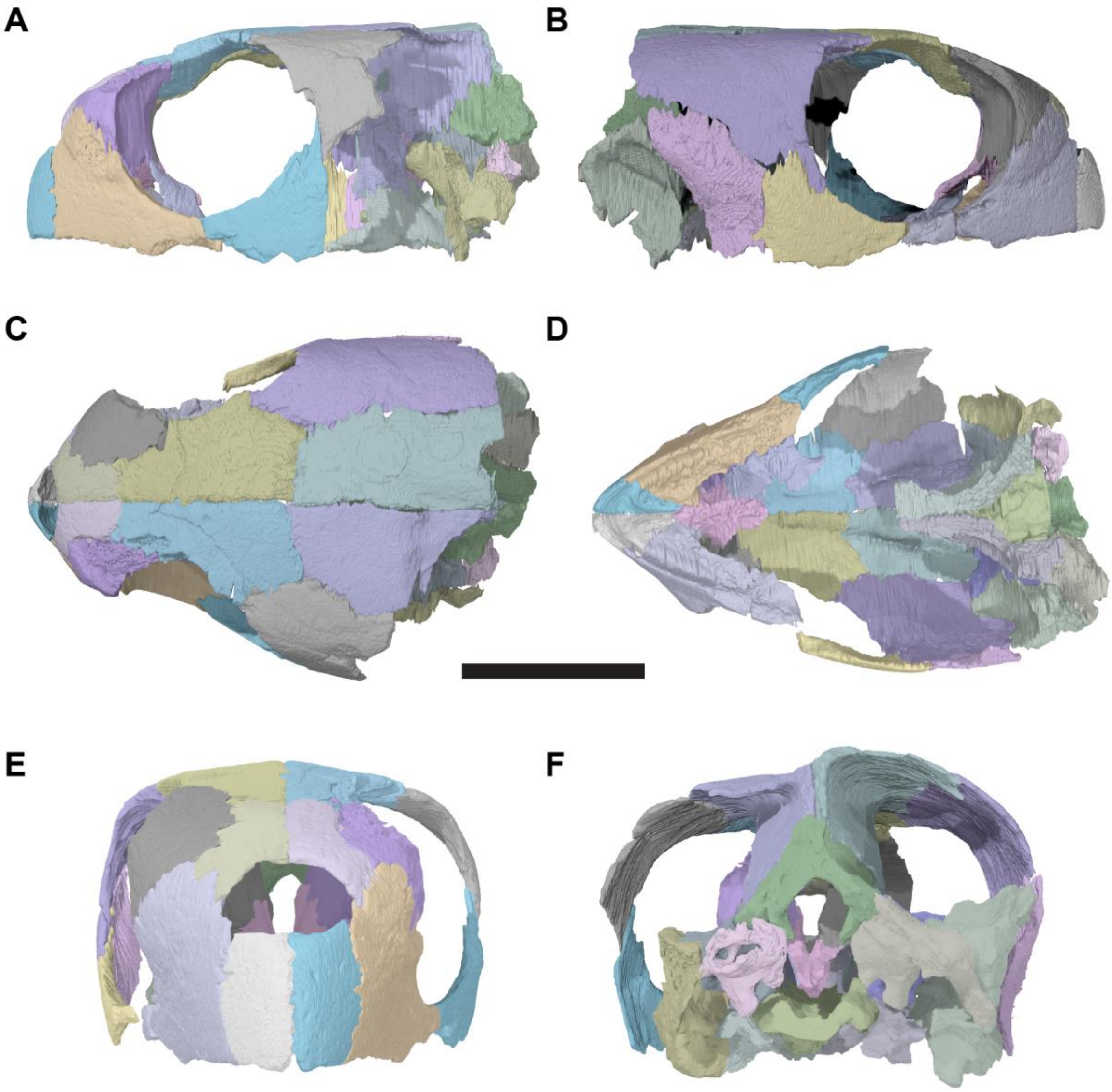

FIG. S1.6. 3D renderings of NHMUK PV R2226, the holotype of *R. elegans*. **A**, left lateral view; **B**, right lateral view; **C**, dorsal view; **D**, ventral view; **E**, anterior view; **F**, posterior view. Scale bar equals 20 mm.

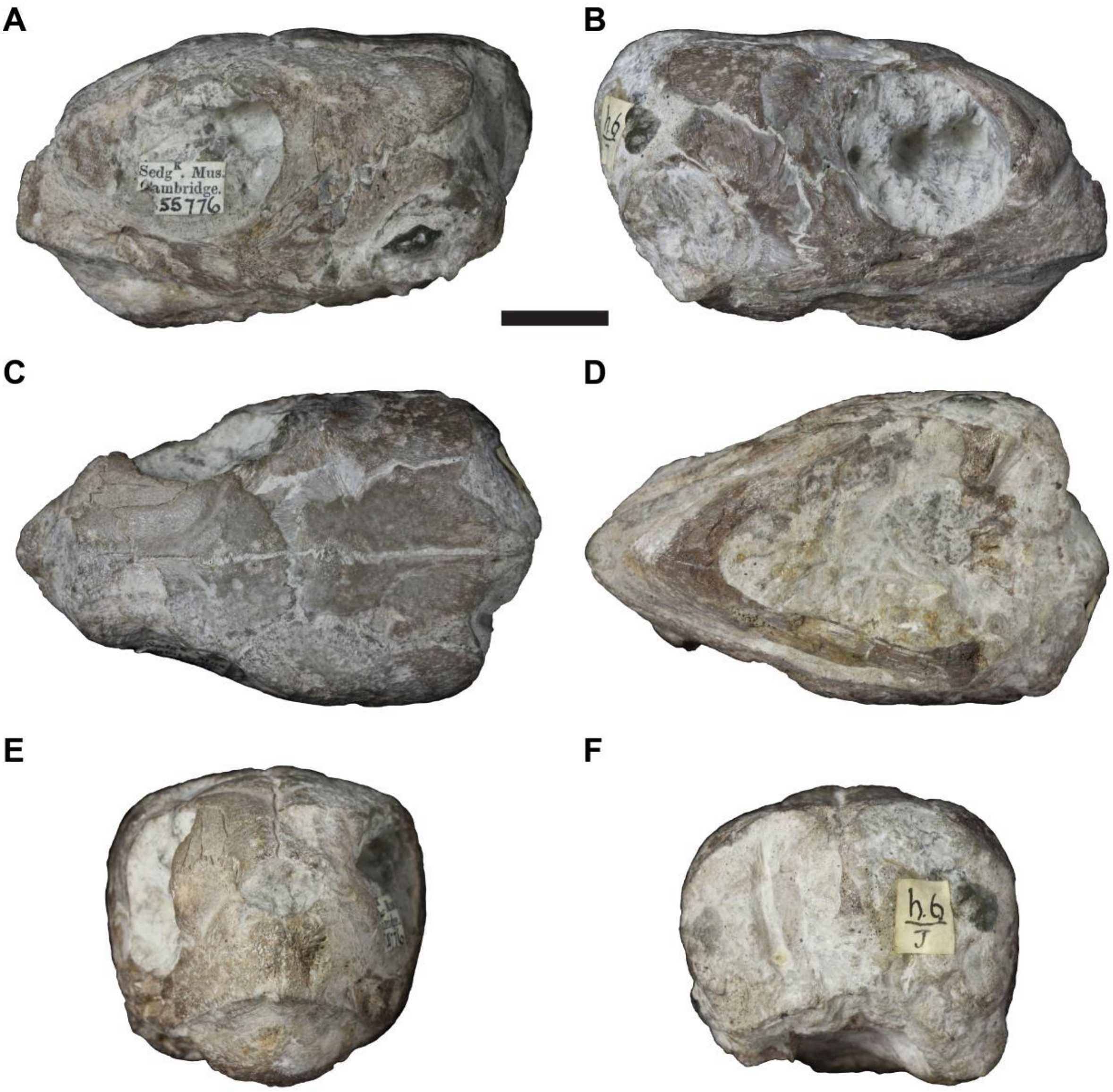

FIG. S1.7. Photographs of CAMSM B55776. **A**, left lateral view; **B**, right lateral view; **C**, dorsal view; **D**, ventral view; **E**, anterior view; **F**, posterior view. Scale bar equals 10 mm.

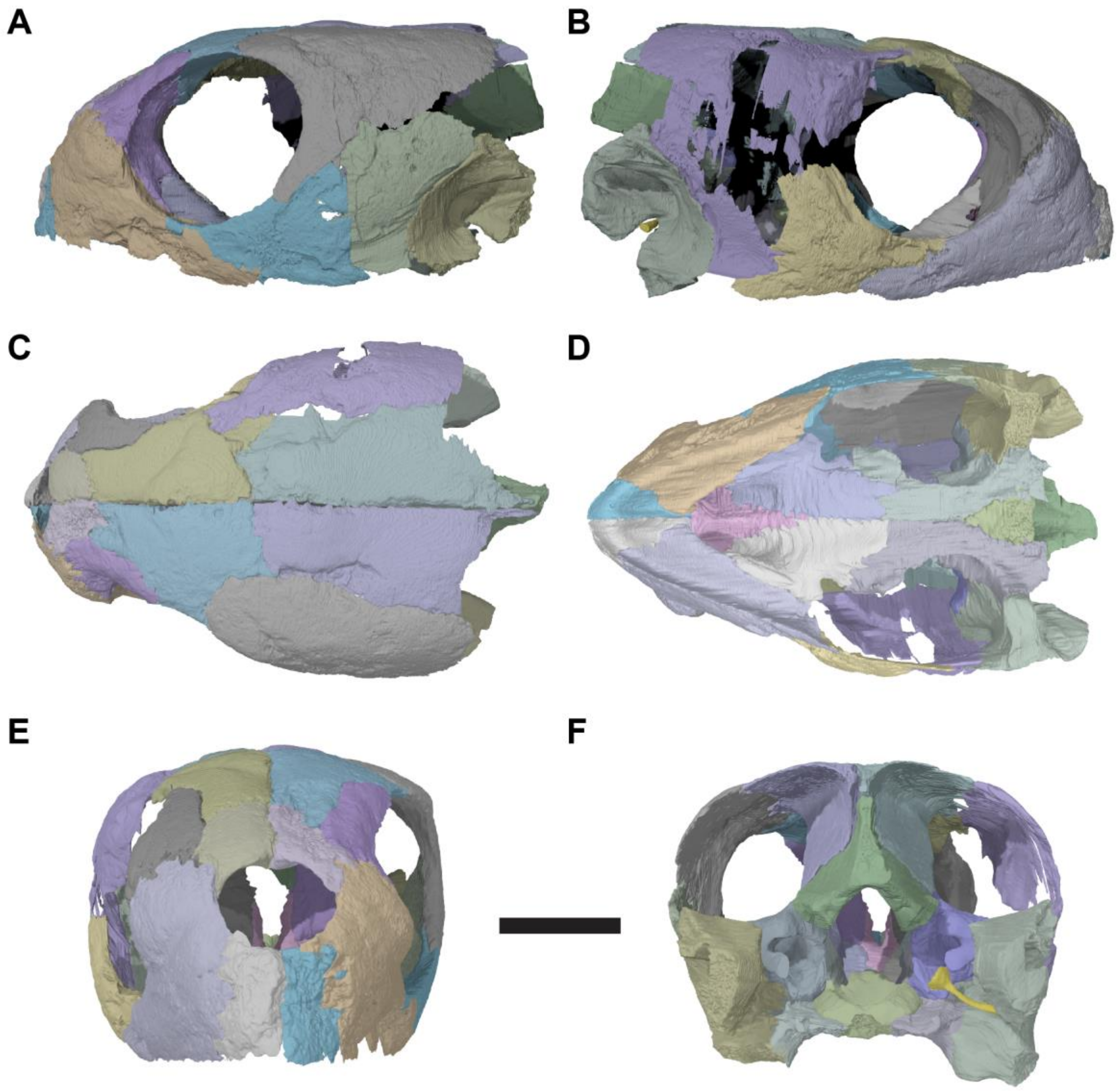

FIG. S1.8. 3D renderings of CAMSM B55776. **A**, left lateral view; **B**, right lateral view; **C**, dorsal view; **D**, ventral view; **E**, anterior view; **F**, posterior view. Scale bar equals 10 mm.

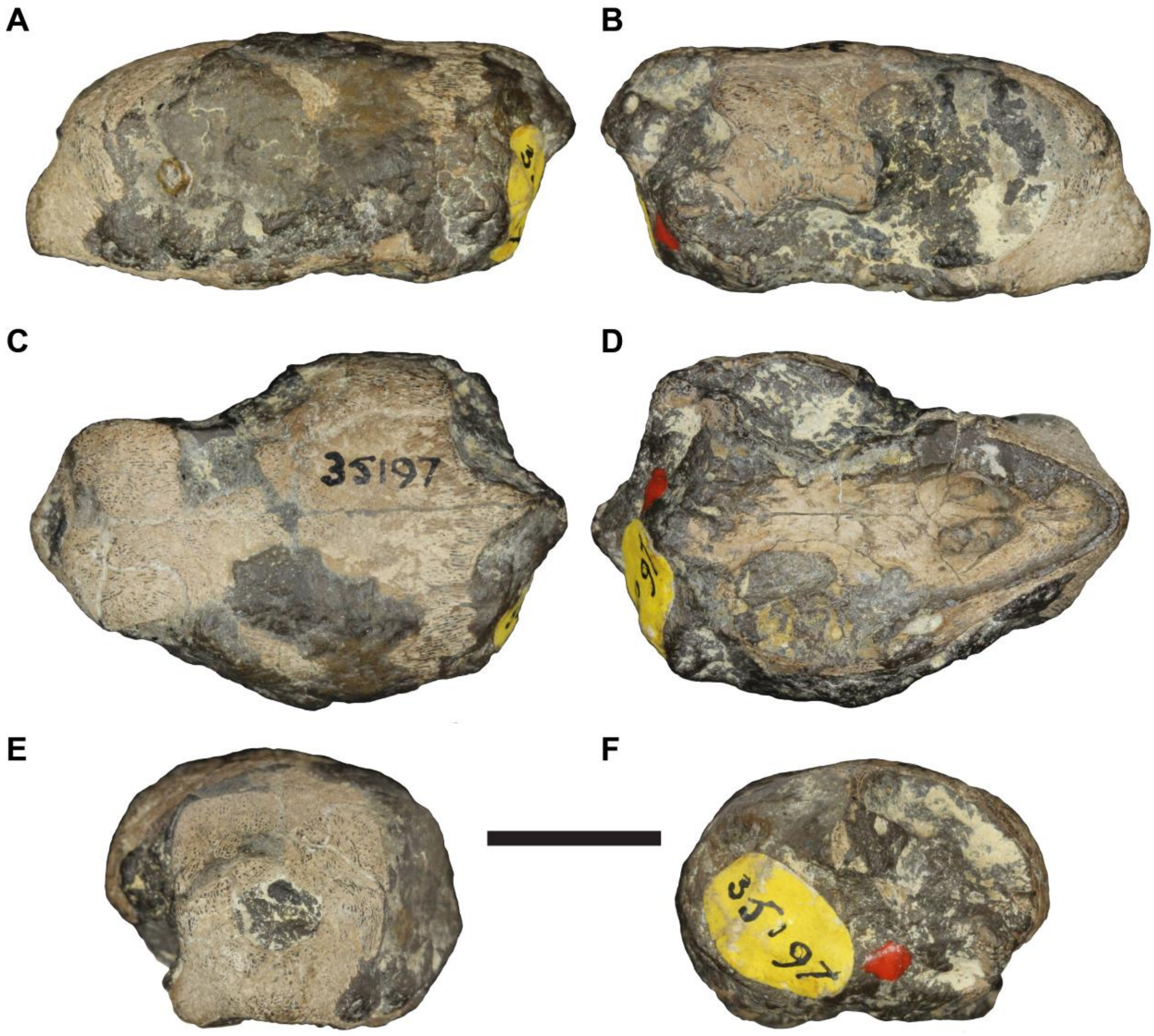

FIG. S1.9. Photographs of NHMUK 35197. **A**, left lateral view; **B**, right lateral view; **C**, dorsal view; **D**, ventral view; **E**, anterior view; **F**, posterior view. Scale bar equals 10 mm.

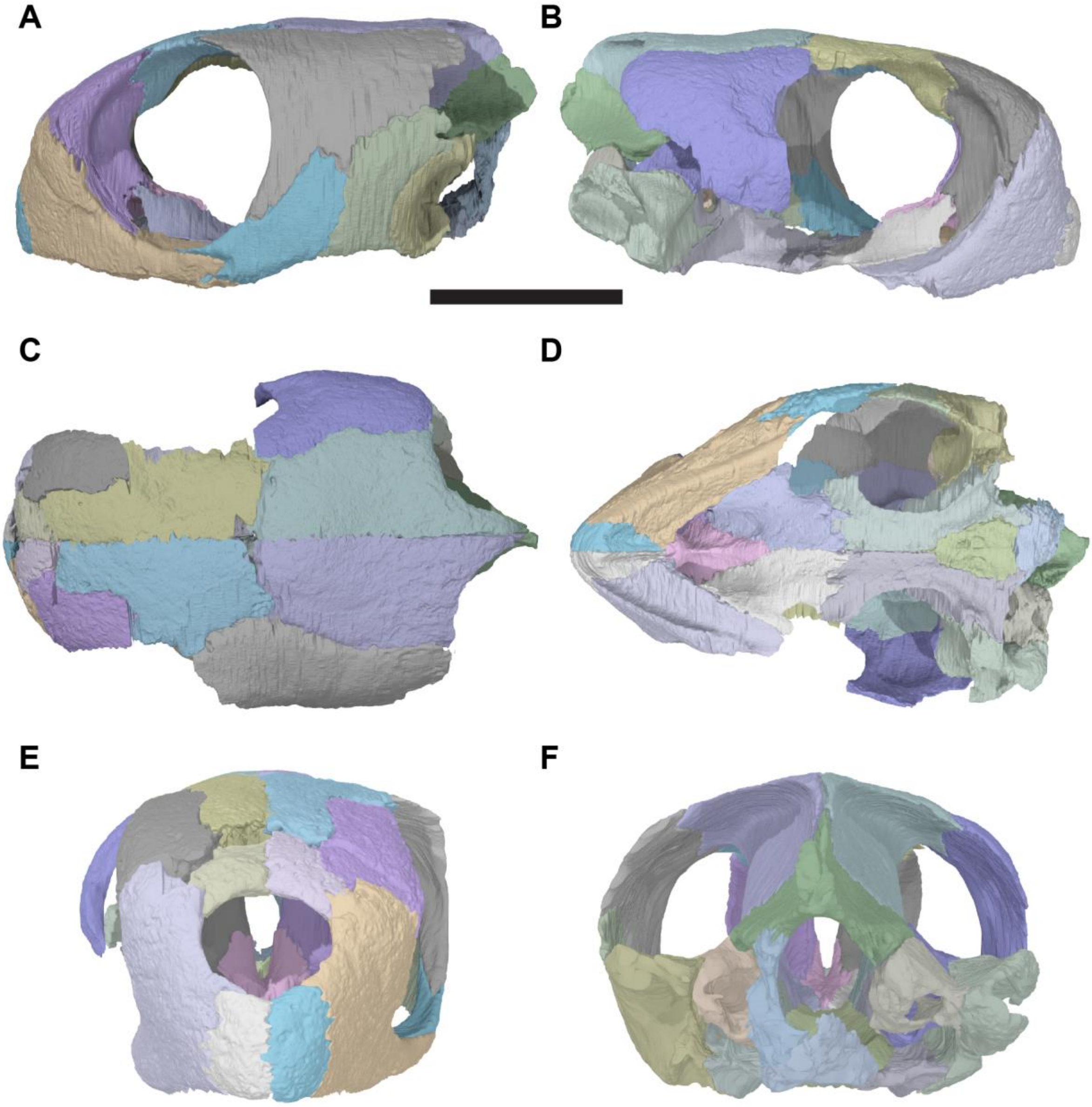

FIG. S1.10. 3D renderings of NHMUK R35197. **A**, left lateral view; **B**, right lateral view; **C**, dorsal view; **D**, ventral view; **E**, anterior view; **F**, posterior view. Scale bar equals 10 mm.

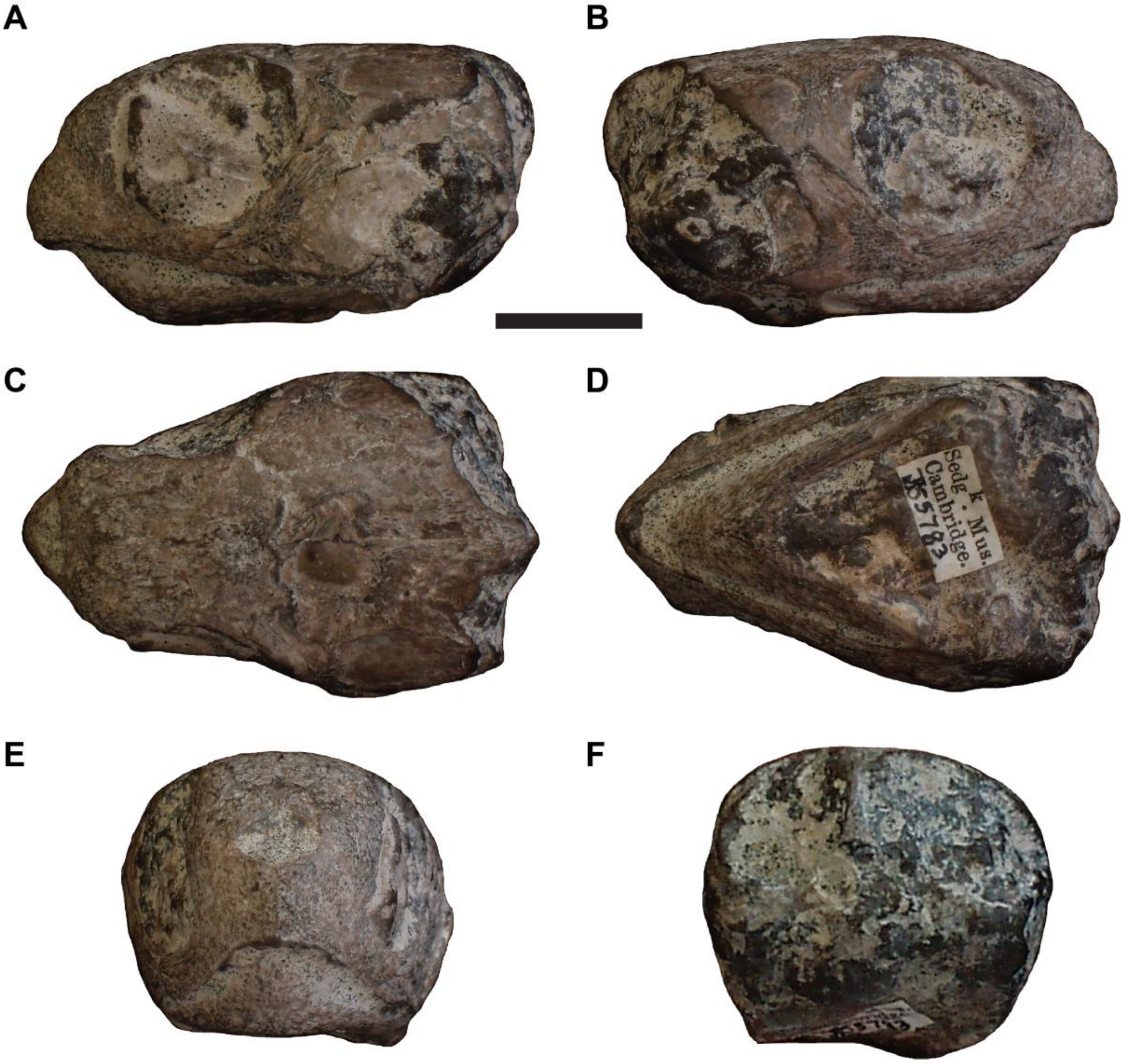

FIG. S1.11. Photographs of CAMSM B55783. **A**, left lateral view; **B**, right lateral view; **C**, dorsal view; **D**, ventral view; **E**, anterior view; **F**, posterior view. Scale bar equals 10 mm.

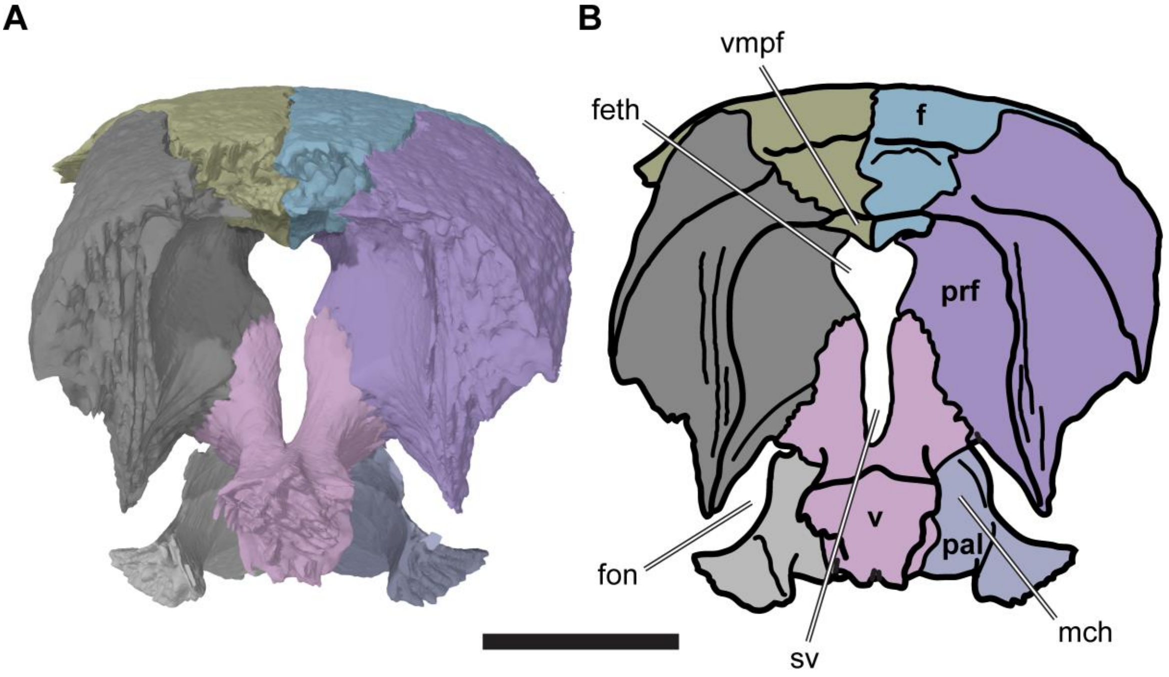

FIG. S1.12. Anterior view of partial anterior part of the cranium of CAMSM B55783 showing parts of the nasal capsule. **A**, 3D rendering; **B**, interpretative line drawing. Scale bar equals 5 mm. Note that bones are labelled in bold. Abbreviations: *f*, frontal; *feth*, fissura ethmoidalis; *fon*, foramen orbito-nasale; *mch*, meatus choane; *prf*, prefrontal; *pal*, palatine; *sv*, sulcus vomeri; *v*, vomer; *vmpf*, ventromedial process of frontal.

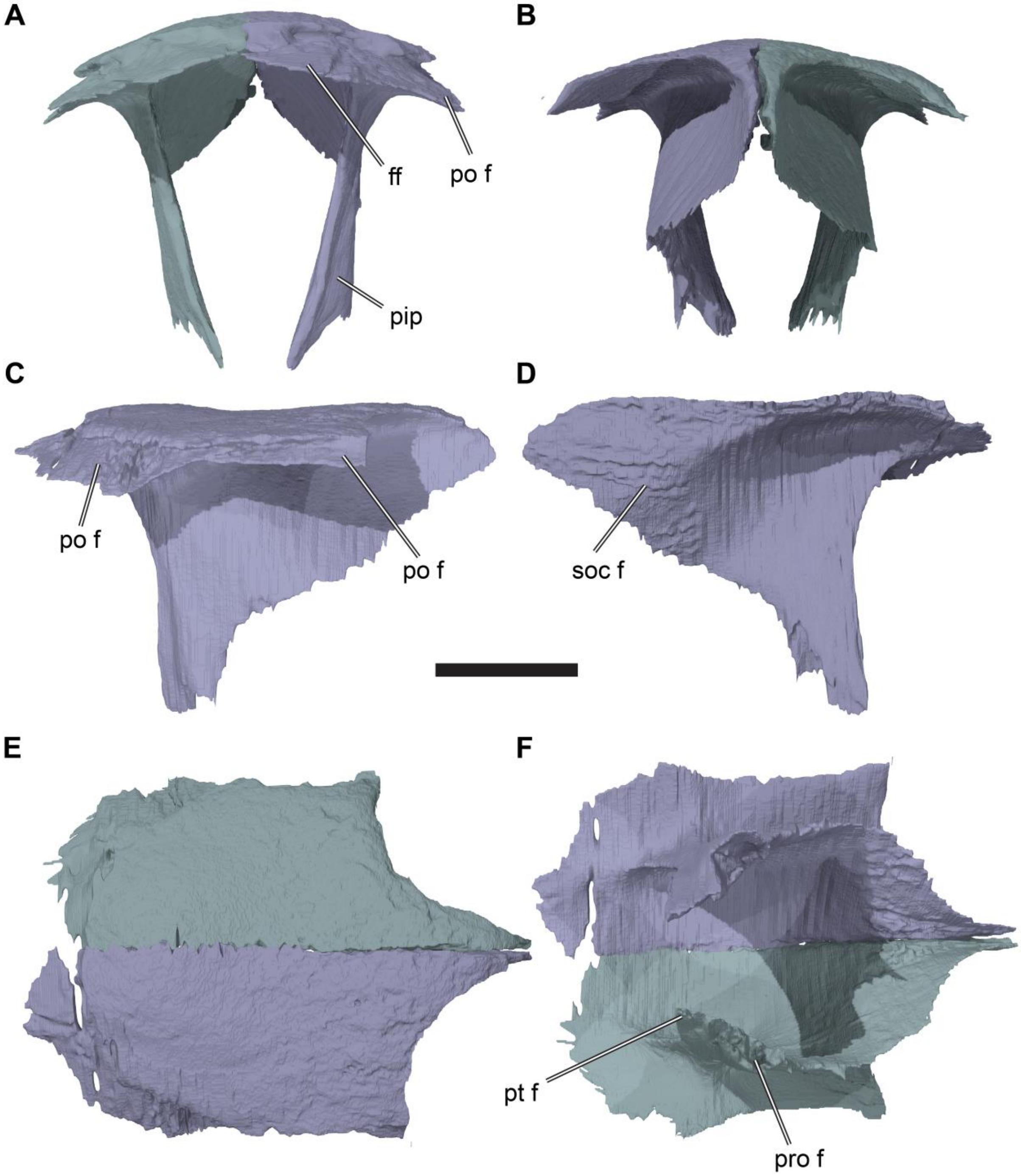

FIG. S1.13. 3D rendering of the left and right parietals of NHMUK R35197. Scale bar equals 5 mm.

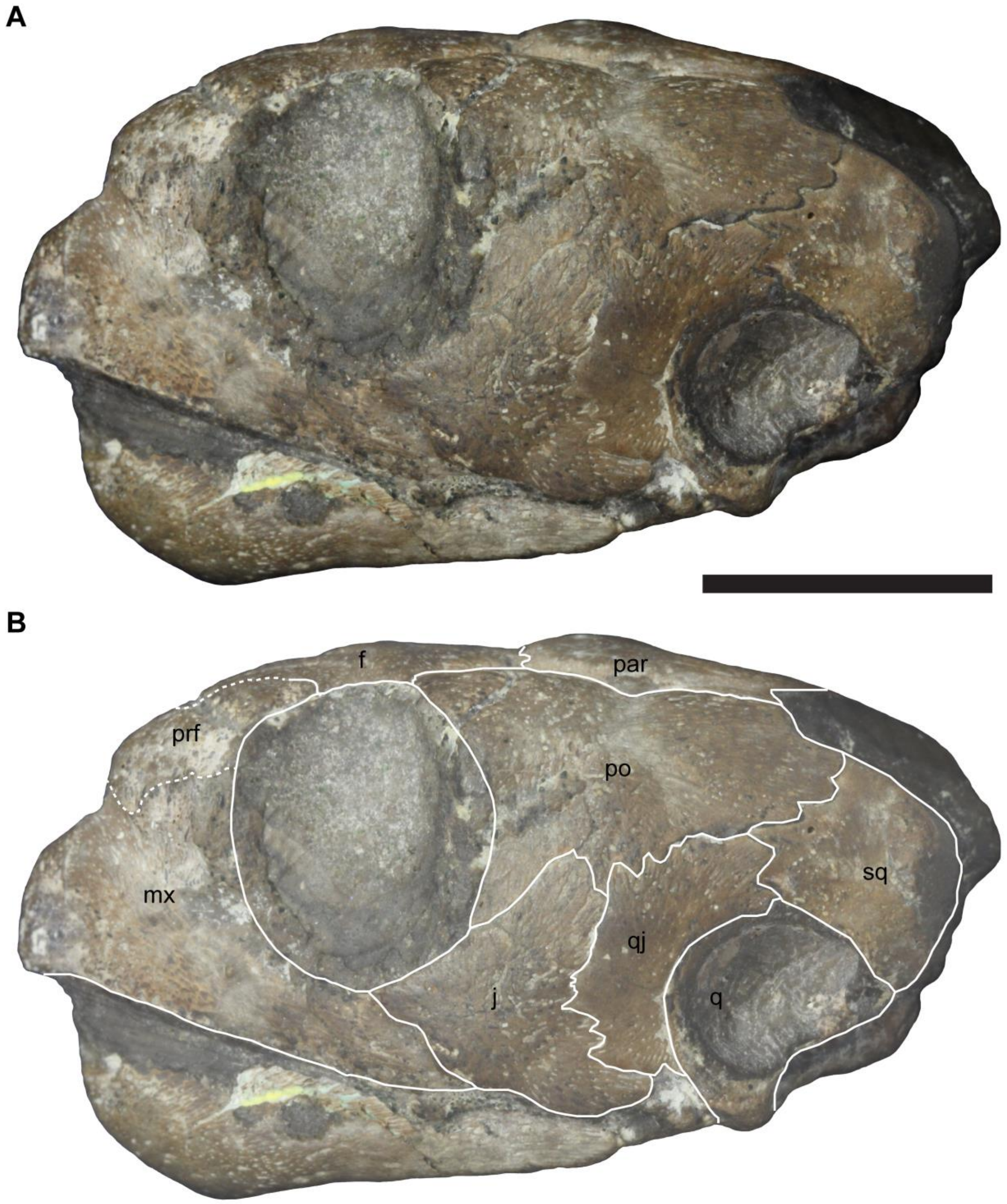

FIG. S1.14. Left lateral side of the cranium of CAMSM B55791, showing the contacts of the squamosal. **A**, Photograph; **B**, Photograph with sutures indicated. Dashed lines represent uncertainity about the exact placement of the suture. Scale bar equals 10 mm. Abbreviations: *f*, frontal; *j*, jugal; *mx*, maxilla; *par*, parietal; *po*, postorbital; *prf*, prefrontal; *q*, quadrate; *qj*, quadratojugal; *sq*, squamosal.

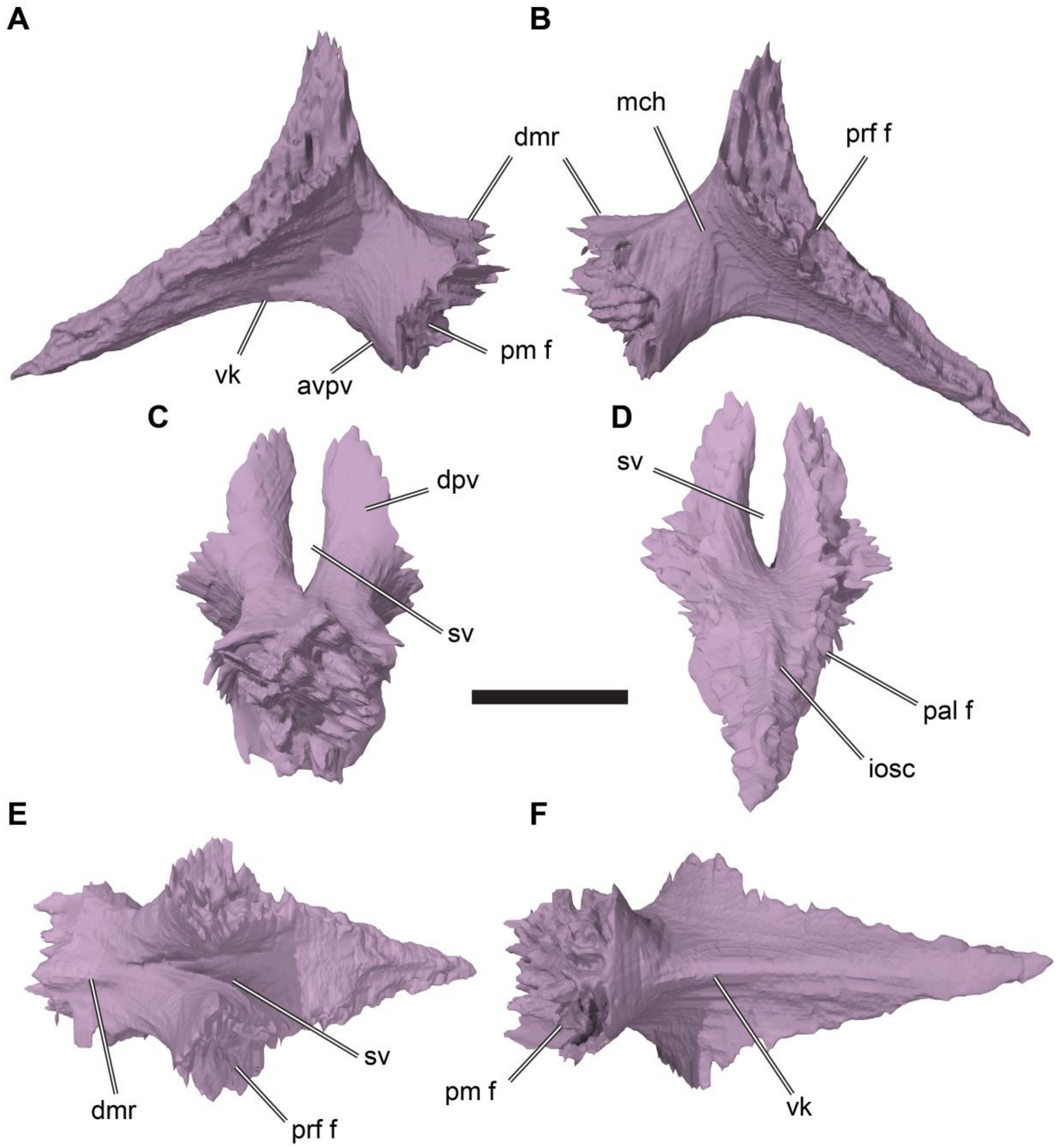

FIG. S1.15. 3D rendering of vomer of CAMSM B5583. **A**, right lateral view; **B**, left lateral view; **C**, anterior view; **D**, posterior view; **E**, dorsal view; **F**, ventral view. Scale bar equals 3 mm. Abbreviations: *avpv*, anteroventral process of vomer; *dmr*, dorsal medial ridge of vomer; *dvp*, dorsal process of vomer; *iosc*, interorbital septum crest; *mch*, meatus choanae; *pm f*, facet for premaxilla; *prf f*, facet for prefrontal; *sv*, sulcus vomeri; *vk*, vomer keel;

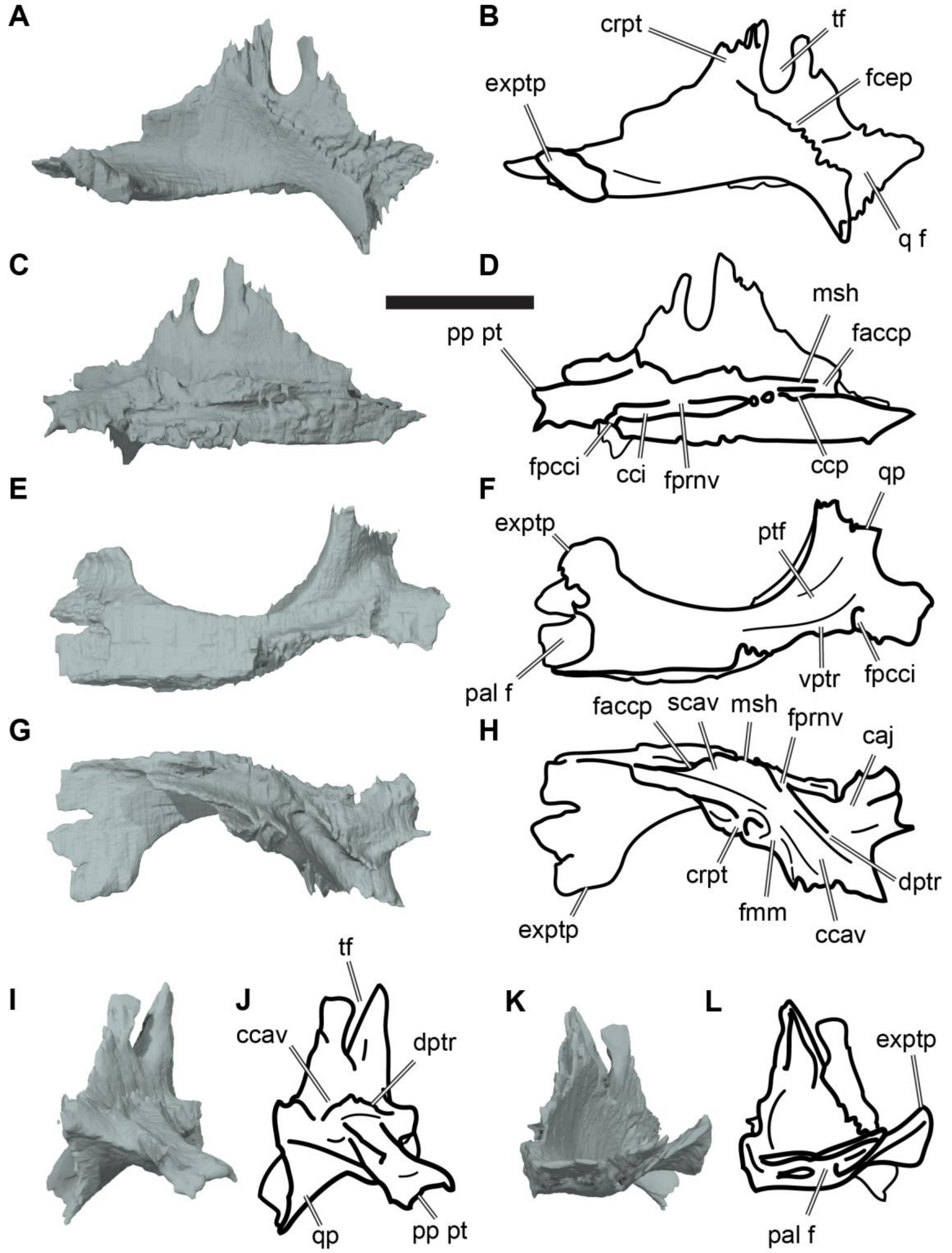

FIG. S1.16. Left pterygoid of CAMSM B5583. **A**, 3D rendering in lateral view; **B**, interpretative line drawing of A; **C**, 3D rendering in medial view; **D**, interpretative line drawing of B; **E**, 3D rendering in ventral view; **F**, interpretative line drawing of E; **G**, 3D rendering in dorsal view; **H**, interpretative line drawing of H; **I**, 3D rendering in posterior view; **J**, interpretative line drawing of I; **K**, 3D rendering in anterior view; **L**, interpretative line drawing of K. Scale bar equals 5 mm. Abbreviations: *caj*, cavum acustico-jugulare; *ccav*, canalis cavernosus; *cci*, canalis caroticus internus; *ccp*, canalis caroticus palatinus; *crpt*, crista pterygoidei; *dptr*, dorsal pterygoid ridge; *exptp*, external pterygoid process; *faccp*, foramen anterius canalis carotici palatinum; *fcep*, fossa cartilaginis expipterygoidei; *fpcci*, foramen posterius canalis carotici interni; *fprnv*, foramen pro ramo nervi vidani; *msh*, medial shelf; *pal f*, facet for palatine; *pp pt*, posterior process of pterygoid; *ptf*, pterygoid fossa; *q f*, facet for quadrate; *qp*, quadrate process of pterygoid; *scav*, sulcus cavernosus; *tf*, trigeminal foramen; *vptr*, ventral pterygoid ridge.

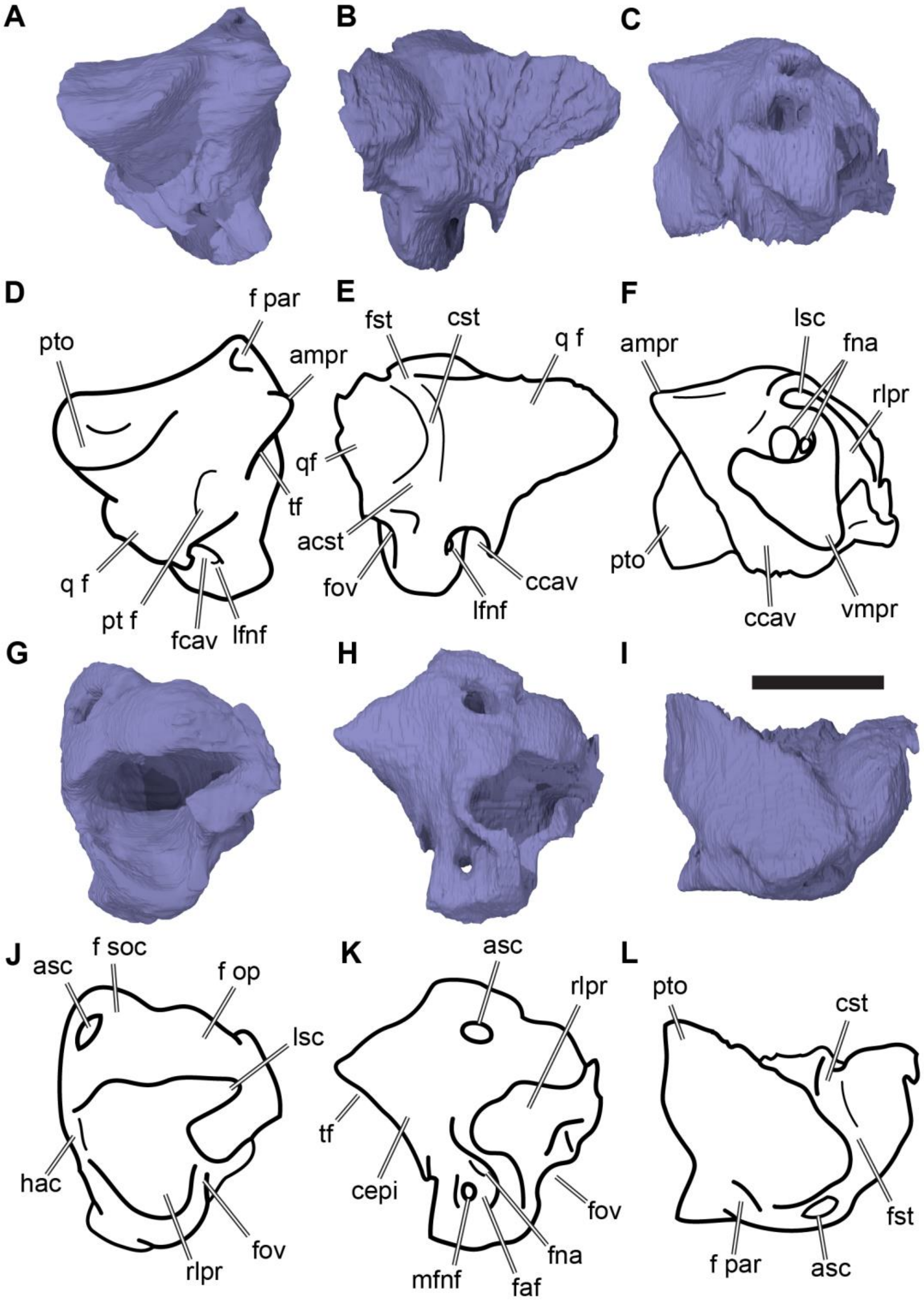

FIG. S1.17. Right prootic of CAMSM B55775. **A**, 3D rendering in anterior view; **B**, 3D rendering in medial view; **C**, 3D rendering in ventral view; **D**, interpretative line drawing of A; **E**, interpretative line drawing of B; **F**, interpretative line drawing of C; **G**, 3D rendering in posterior view; **H**, 3D rendering in medial view; **I** 3D rendering in dorsal view; **J**, interpretative line drawing of G; **K**, interpretative line drawing of H; **L**, interpretative line drawing of I. Scale bar equals 5 mm. Abbreviations: *acst*, aditus canalis stapedio-temporalis; *asc*, anterior semicircular canal; *ampr*, anteromedial process of prootic; *ccav*, canalis cavernosus; *cepi*, cavum epiptericum; *cst*, canalis stapedio-temporalis; *faf*, fossa acustico-facialis; *fcav*, foramen cavernosum; *fna*, foramina nervi acustici; *f par*, facet for parietal; *f op*, facet for opisthotic; *fov*, fenestra ovalis; *fst*, foramen stapedio-temporale; *f soc*, facet for supraoccipital; *hac*, hiatus acusticus; *lfnf*, lateral foramen nervi facialis; *mfnf*, medial foramen nervi facialis; *lsc*, lateral semicircular canal; *pt f*, facet for pterygoid; *pto*, processus trochlearis oticum; *q f*, facet for quadrate; *rlpr*, recessus labyrinthicus prooticus; *tf*, trigeminal foramen; *vmpr*, ventromedial process of prootic.

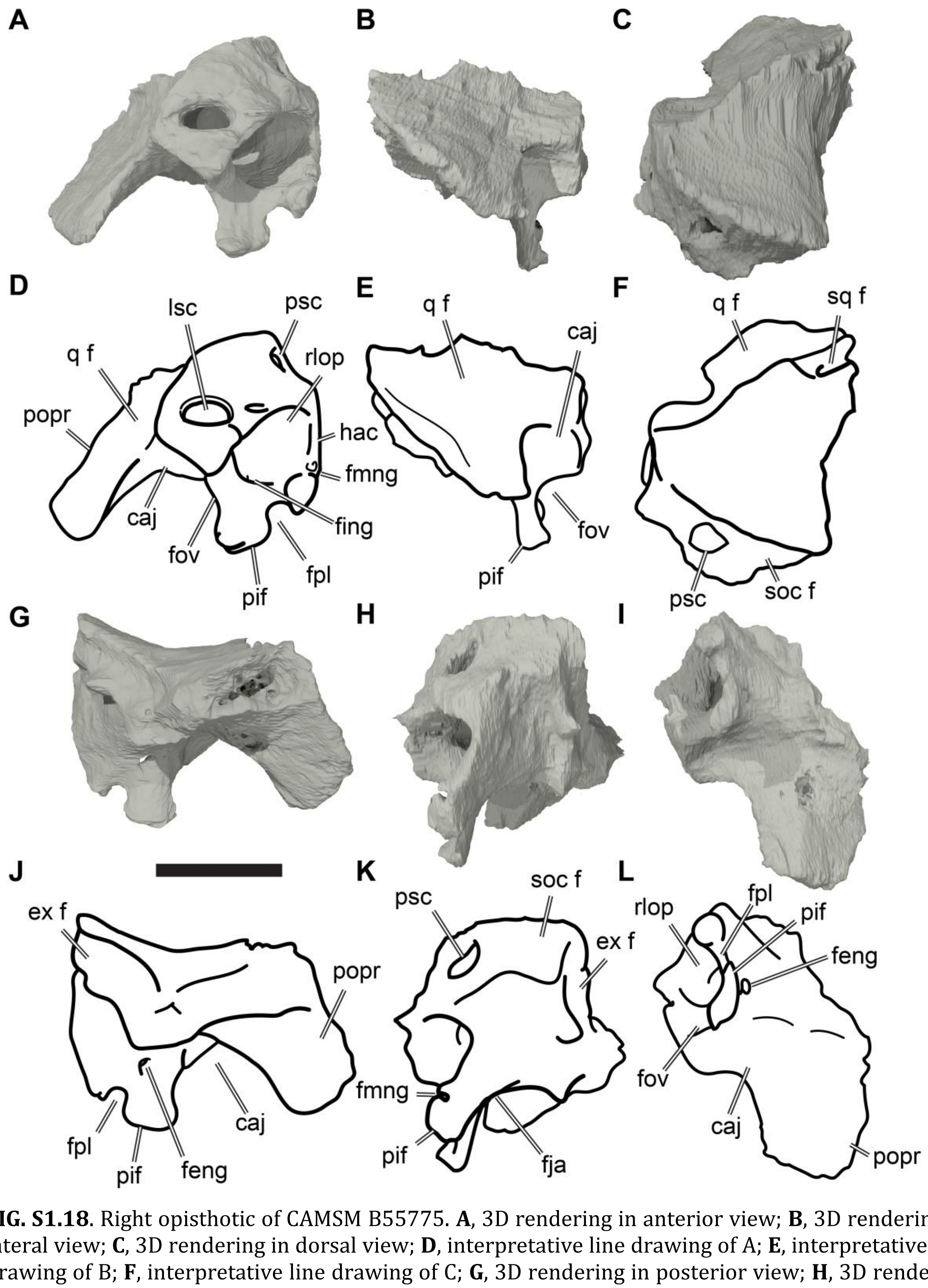

FIG. S1.18. Right opisthotic of CAMSM B55775. **A**, 3D rendering in anterior view; **B**, 3D rendering in lateral view; **C**, 3D rendering in dorsal view; **D**, interpretative line drawing of A; **E**, interpretative line drawing of B; **F**, interpretative line drawing of C; **G**, 3D rendering in posterior view; **H**, 3D rendering in medial view; **I**, 3D rendering in ventral view; **J**, interpretative line drawing of G; **K**, interpretative line drawing of H; **L**, interpretative line drawing of I. Scale bar equals 5 mm. Abbreviations: *caj*, cavum acustico-jugulare; *feng*, foramen externum nervi glosspharyngei; *fing*, foramen internum nervi glossopharyngei; *fja*, foramen jugulare anterius; *fmng*, foramen medialis nervi glosspharyngei; *fov*, fenestra ovalis; *fpl*, fenestra perilymphatica; *ex f*, facet for exoccipital; *hac*, hiatus acusticus; *lsc*, lateral semicircular canal; *pif*, processus interfenestralis; *popr*, paroccipital process of opisthotic; *psc*, posterior semicircular canal; *q f*, facet for quadrate; *rlop*, recessus labyrinthicus opisthoticus; *soc f*, facet for supraoccipital; *sq f*, facet for squamosal.

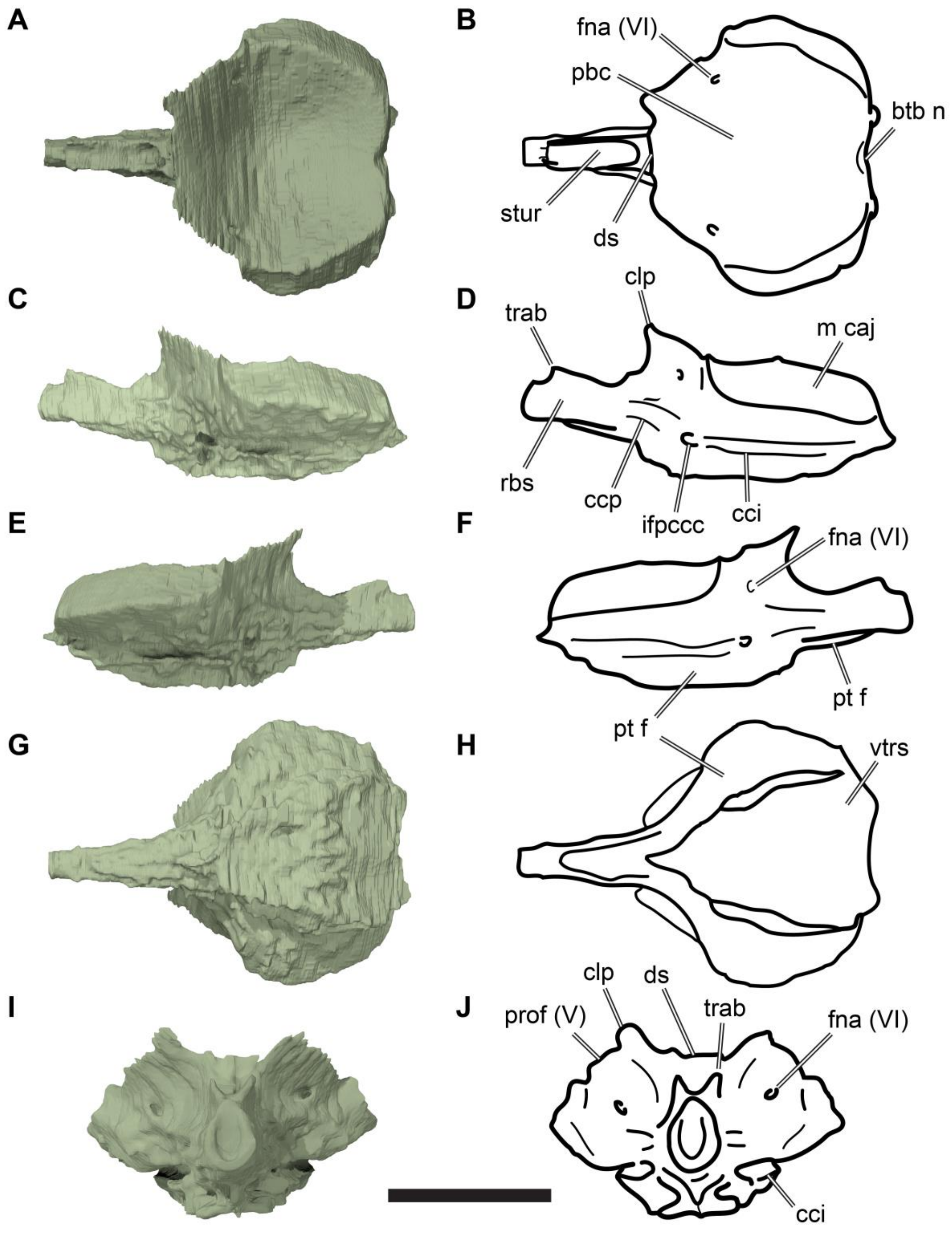

FIG. S1.19. **A**, 3D rendering in dorsal view; **B**, interpretative line drawing of B; **C**, 3D rendering in left lateral view; **D**, interpretative line drawing of C; **E**, 3D rendering in right lateral view; **F**, interpretative line drawing of E; **G**, 3D rendering in ventral view; **H**, interpretative line drawing of H; **I**, 3D rendering in anterior view; **J**, interpretative line drawing of I. Scale bar equals 3 mm. Abbreviations: *btb n*, basis tuberculi basialis notch; *cci*, canalis caroticus internus; *ccp*, canalis caroticus palatinum; *clp*, clinoid process; *ds*, dorsum sellae; *fna (VI)*, foramen nervi abducentis; *ifpccc*, internal foramen posterius canalis carotici cerebralis; *m caj*, margin of parabasisphenoid facing cavum acustico-jugulare; *pbc*, parabasisphenoid cup; *prof (V)*, prootic foramen; *pt f*, facet for pterygoid; *rbs*, rostrum basisphenoidale; *stur*, sella turcica; *trab*, trabeculae; *vtrs*, ventral triangular surface.

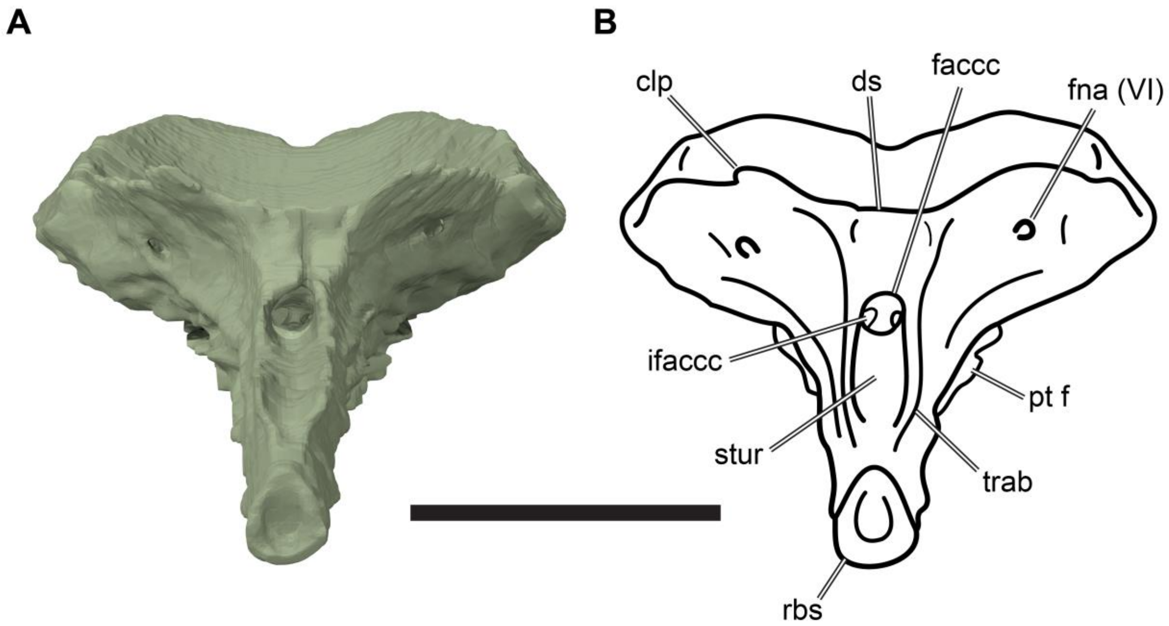

FIG. S1.20. Anterodorsal view of parabasisphenoid of NHMUK 35197. **A**, 3D rendering; **B**, interpretative line drawing. Scale bar equals 3 mm. Abbreviations: *clp*, clinoid process; *ds*, dorsum sellae; *faccc*, foramen anterius canalis carotici cerebralis; *fna (VI)*, foramen nervi abducentis; *ifaccc*, internal foramen anterius canalis carotici cerebralis; *pt f*, pterygoid facet; *rbs*, rostrum basisphenoidale; *stur*, sella turcica; *trab*, trabecular.

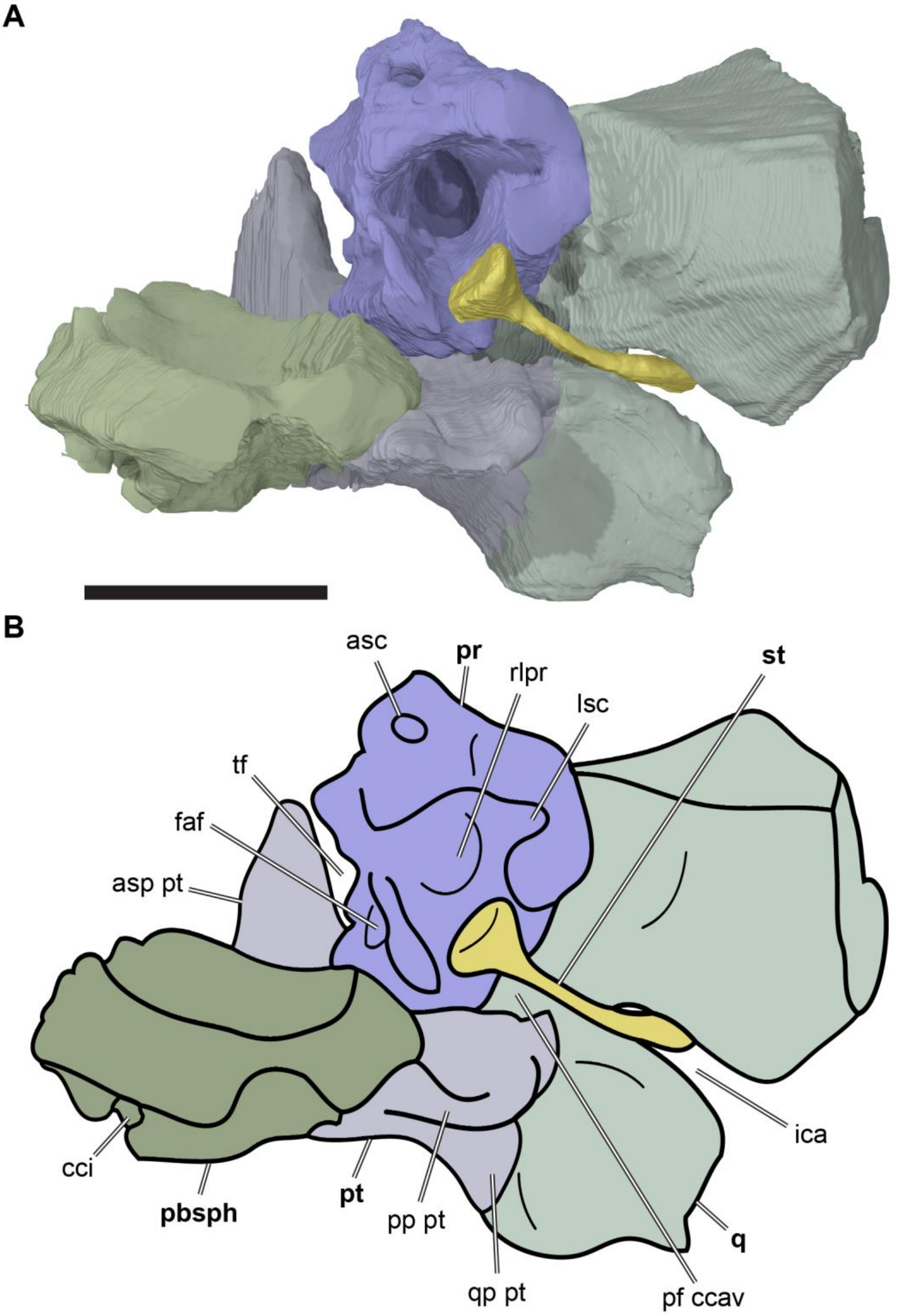

FIG. S1.21. Posteromedial view of partial otic capsule and cavum acustico-jugulare of CAMSM B55776 with stapes *in situ*. **A**, 3D rendering; **B**, interpretative line drawing. Scale bar equals 5 mm. Note that bones are labelled in bold. Abbreviations: *asc*, anterior semicircular canal; *asp pt*, ascending process of pterygoid; *cci*, canalis caroticus internus; *faf*, fossa acustico-facialis; *ica*, incisura columella auris; *lsc*, lateral semicircular canal; *pbsph*, parabasisphenoid; *pf ccav*, posterior foramen for the canalis cavernosus; *pp pt*, posterior process of pterygoid; *pr*, prootic; *pt*, pterygoid; *q*, quadrate; *qp pt*, quadrate process of pterygoid; *rlpr*, recessus labyrinthicus prooticus; *st*, stapes; *tf*, trigeminal foramen.

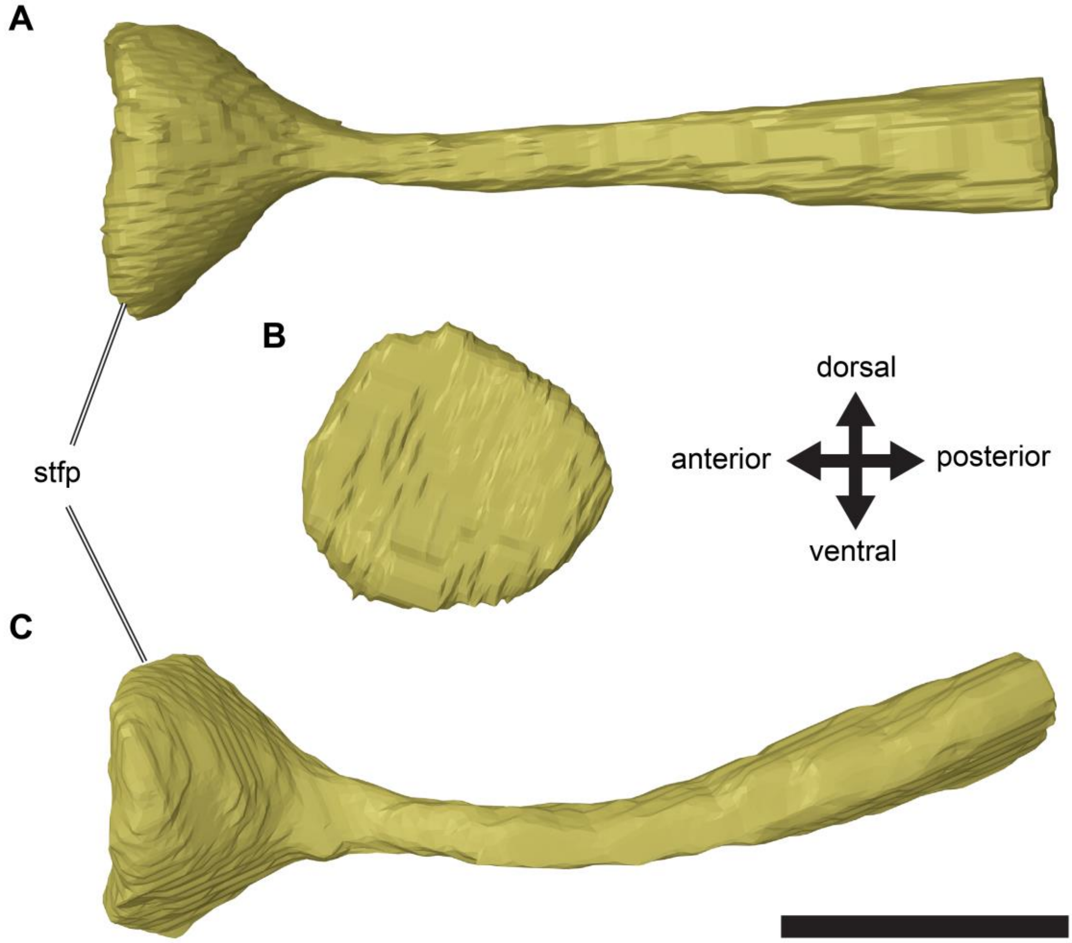

FIG. S1.22. 3D renderings of stapes of CAMSM B55776. **A**, dorsal view; **B**, medial view; **C**, posterior view. Scale bar equals 2 mm. Abbreviations: *stfp*, stapedial footplate.

CHARACTER MODIFIACTIONS

Cranial character modifications

Our observations for *Rhinochelys pulchriceps* show that this taxon has a peculiar articulation between the pterygoid and the basisphenoid (e.g. main text Fig. 15E–F). The pterygoid of *Rhinochelys* has a posterodorsally facing, concave socket that receives an anterolateral knob-like process of the basioccipital. Hooks (1998) described this feature for *Calcarichelys gemma* and *Protostega gigas* and found it to be a synapomorphy of Protostegidae (excluding *Rhinochelys*). To our knowledge, the respective character (Hooks 1998: character 10) has not been used in other matrices, but we include it here:

Character 93: Basioccipital, anterolateral edge of basioccipital with knob-like processes fitting into sockets on the posterior processes of the pterygoids: 0 = absent; 1 = present.

We also code another new cranial character, based on the observations of Collins (1970), Tong *et al.* (2006), Scavezzoni & Fischer (2018) and this study, regarding the peculiar antorbital bulge found in *Rhinochelys pulchriceps* and *Rhinochelys nammourensis*, but no other protostegid (e.g. main text Figs 1, 2B, D; Data S1: Figs S.1–11).

Character 10 (new): Prefrontal, preorbital bulge formed between the prefrontal and premaxilla: 0 = absent; 1 = present.

Postcranial character modifications

Costal characters. Previously, variation regarding the costo-peripheral fontanelles was coded in a single multistate character encoding both the presence vs. absence of fontanelles, plus various aspects of their morphology if present (Evers & Benson 2018*a*: character 210; Cadena & Parham 2015: character 132). Here, we have modified the coding to consider differences in the arrangement of costo-peripheral fontanelles and their presence/absence separately. Adopting this coding strategy results in three different characters (character 212–214). The first character considers the presence vs.

absence of fontanelles, which we regard as a single observation on one level of homology, following the coding strategy of Hawkins, Hughes & Scotland (1997). Contrary to the postcranial scorings in Evers & Benson (2018*a*), which were adopted from Cadena & Parham (2015), we scored chelydrids, as well as some trionychids and *Araripemys*, as having fontanelles. The presence of fontanelles in chelydrids is unambiguous (see also Joyce 2016). For trionychids, costo-peripheral fontanelles are harder to define because this group lacks peripherals (Meylan 1987; except for *Lissemys punctata*). However, many trionychid taxa have distal rib ends that extend laterally beyond the margin of the costals, therefore creating 'fontanelles' that are laterally open. In this study, we scored trionychid taxa as possessing fontanelles when the distal rib end is visible beyond the costal margin in dorsal or ventral view. Our second costal character captures variation regarding the position of the costo-peripheral fontanelles: in many chelonioid taxa (e.g. *Toxochelys*, *Allopleuron*, *Caretta*; Fig. S1.23B, D–E), costo-peripheral fontanelles are present along all peripherals, whereas fontanelles are present only in certain parts of the carapace (usually the central parts) in many other taxa, such as the thalassemydid *Palaeomedusa* (Fig. S1.23C), the chelydrids *Macrochelys* and *Chelydra*, and some trionychids. We also add a caveat concerning the ontogenetic stage of the fossils considered for scoring: in some taxa, such as *Plesiochelys etalloni* and *Puppigerus camperi*, fonatenelles are present along the lateral margin of the costals in juvenile and subadult specimens but are entirely absent in large specimens (Fig. S1.23B). This differs from how these taxa were scored in many previous studies (e.g. Cadena & Parham 2015). Our third character captures variation in the shape of the fontanelle between the first costal, nuchal and the first few peripherals. This fontanelle is usually only present in those taxa that have fontanelles along the entire costo-peripheral series. However, in *Toxochelys, Corsochelys* and *Erquelinnesia* this fontanelle is reduced in size (Fig. S1.23B), with the first costal nearly contacting the posterior margin of the first peripherals.

Character 212: <u>Costals, lateral ossification</u>: 0 = all costals fully ossified laterally with strong sutural contact with peripherals, lack of dorsal exposure of distal end of costal ribs and absence of costo-peripheral fontanelles; 1 = lateral sutural contact contact between costals and peripherals absent in at least parts of the costo-peripherial series, resulting in the presence of costo-peripheral fontanelles and/or the exposure of the distal rib ends.

Character 213: Costals, position of costo-peripheral fontanelles and exposure of dorsal rib ends: 0 = limited to parts of the carapace; 1 = fontanelles and exposed rib ends present and retained in adults between all costals and along the anterior margin of the first costal. Scored as inapplicable for taxa that lack costo-peripheral fontanelles (character 212.0).

Previous character definition: Character 210 (Evers & Benson 2018*a*); character 132 (Cadena & Parham 2015). Costals, distal rib end and lateral ossification of the costal: 0 = costals fully ossified laterally with strong sutural contact with peripherals, lack of dorsal exposure of distal end of costal ribs; 1 = costals fully ossified laterally with strong sutural contact with peripherals, distal end of costal ribs exposed on dorsal surface and surrounded by the peripheral; 2 = costals lack lateral ossification, allowing the dorsal exposure of the distal end of ribs and the development of fontanelles only at the most anterior and posterior costals; 3 = costals with extreme lost of lateral ossification, allowing the dorsal exposure of the distal end of ribs, in almost all series of costals.

Character 214 (new): Costal, fontanelle along anterior margin of costal 1: 0 = anterior margin of first costal positioned very close to nuchal and/or anterior-most peripherals, reducing the fontanelle to an anteroposteriorly narrow, slot-like opening; 1 = extensive fontanelle between first costal and anterior margin of carapace. Scored inapplicable for taxa without costo-peripheral fontanelles (character 212.0) or when costo-peripheral fontanelles are absent along the first costal.

We also observed further variation in the shape of the costal bones. The costal bones are mediolaterally relatively wide and anteroposteriorly narrow, forming rectangular plate-like structures in most turtles, including most chelonioids. However, in some taxa with a heavily reduced carapace, such as *Eosphargis breineri* or *Allopleuron hofmanni*, the costals are significantly reduced in mediolateral width, and the posterior costals are approximately as wide as they are long (Fig. S1.23E). We included this observation as a separate character:

Character 215 (new): Posterior costals, shape: 0 = rectangular, much wider mediolaterally than long anteroposteriorly; 1 = square or hexagonal, as wide as long.

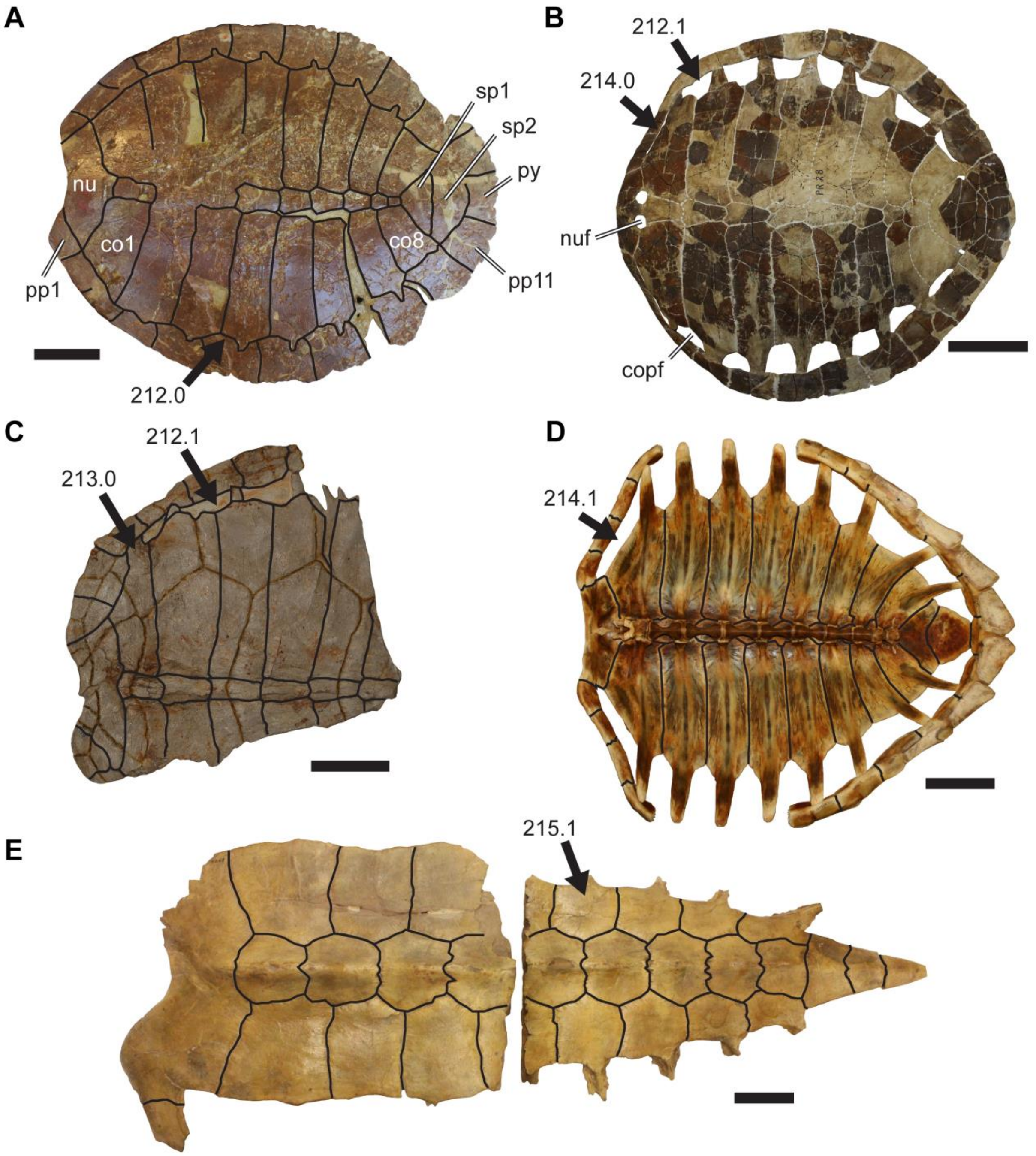

FIG. S1.23. Carapaces of selected turtles illustrating the variability of carapacial fontanelles. **A**, dorsal view of carapace of *Puppigerus camperi* (IRSNB R 0072); **B**, dorsal view of carapace of *Toxochelys* sp. (FMNH PR 28); **C**, dorsal view of partial carapace of *Palaeomedusa testa* (BSPG AS I 818); **D**, ventral view of carapace of *Caretta caretta* (AMNH 129869); **E**, dorsal view of partial carapace of *Allopleuron hofmanni* (IRSNB R 0008). Scale bars in A & C equal 5 cm, all other scale bars equal 10 cm. Numbers represent characters and character states. Abbreviations: *co*, costal; *copf*, costo-peripheral fonatnelle; *nu*, nuchal; *nuf*, nuchal fonatanelle; *pp*, peripheral; *py*, pygal; *sp*, suprapygal.

Entoplastron and epiplastron characters. In previous analyses, the definitions for several characters describing entoplastron shape repeated information from other characters or differed from our observations. Previously, Evers & Benson (2018*a*: character 240) adopted a character (character 156 of Cadena & Parham 2015; character 79 of Joyce 2007) that was originally defined to capture a peculiar feature appearing in the entoplastron of several stem-turtles, including Late Triassic taxa such as *Proganochelys quenstedti* and *Proterochersis robusta*. These taxa have a long posteriorly directed process that overlaps the hypoplastra on the dorsal surface of the plastron and sometimes reaches the mesoplastra (Fig. S1.24B). We deleted this character from our modified matrix, because the presence of a posteriorly elongate ('dagger-shaped') entoplastral process is also described in state 0 of character 242 of Evers & Benson (2018*a*; originally from Cadena & Parham 2015: character 158). As a solution to coding the morphological information of the entoplastron, we modified the previous definition of the entoplastron shape character (Evers & Benson 2018*a*: character 242) to include more anatomical descriptive statements, whereas the shapes were previously defined solely as geometric shapes (e.g. 'diamond', 'T-shaped', *etc.*).

Character 246: Entoplastron, shape of the entoplastron: 0 = 'dagger-shaped', with dorsoventrally thick anterior end and long posterior process that extends along the dorsal surface of the plastron and sometimes reaches the mesoplastra; 1 = plate-like and diamond-shaped or hexagonal in ventral view, with all margins of subequal length; 2 = 'T'-shaped or triangular, i.e. entoplastron has a mediolaterally expanded anterior end and a progressively narrowing posterior process; 3 = strap-like and 'V'-shaped, with posterolateral processes diverging from the midline of the plastron.

Previous character definitions:

(1) Character 240 (Evers & Benson 2018*a*); character 156 (Cadena & Parham 2015). Entoplastron, size of the posterior entoplastral process: 0 = posterior process long, reaching as far posteriorly as the mesoplastra; 1 = posterior process reduced in length.

(2) Character 242 (Evers & Benson 2018*a*); character 158 (Cadena & Parham 2015). Entoplastron, shape of the entoplastron in ventral view: 0 = dagger-shaped; 1 = massive diamond-shaped; 2 = T- shaped, longer than wide; 3 = T-shaped, wider than long, forming broad lateral wings; 4 = strap like and V-shaped.

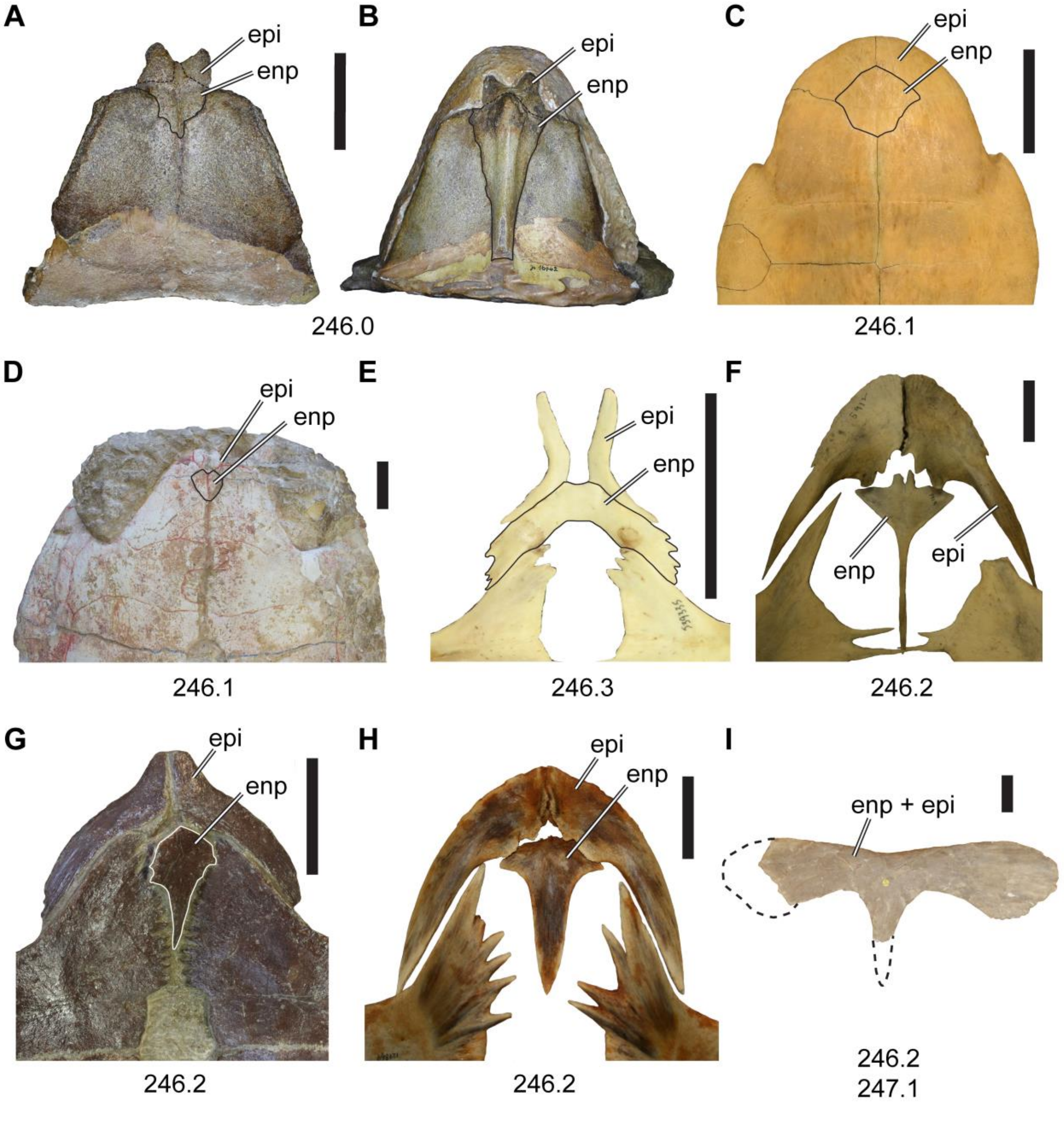

FIG. S1.24. Entoplastra of selected turtles. **A**, ventral view of *Proterochersis robusta* (SMNS 16442); **B**, dorsal view of *Proterochersis robusta* (SMNS 16442); **C**, ventral view of *Podocnemis sextuberculata* (NHMUK 16075); **D**, ventral view of *Plesiochelys etalloni* (MAJ-2005-11-1); **E**, ventral view of *Pelodiscus sinensis* (USNM 539335); **F**, ventral view of *Chelonia mydas* (AMNH 5912); **G**, ventral view of *Puppigerus camperi* (IRSNB R 0073); **H**, ventral view of *Caretta caretta* (AMNH 129869); **I**, ventral view of *Protostega* sp. (NHMUK R 5433). All scale bars equal 5 cm. Numbers represent characters and character states. Abbreviations: *enp*, entoplastron; *epi*, epiplastron.

In addition to the above revisions, we only included one state for a 'T-shaped' entoplastron in our character 246. The two distinct states (states 2 and 3) for a 'T-shaped' entoplastron from the previous definition were combined to a single state (new state 2) to better reflect the homology proposition of having a 'T-shaped' entoplastron versus other possible shapes of this element. The previous state 3 was scored only for *Protostega* and *Archelon* in the matrix of Evers & Benson (2018*a*), following Cadena & Parham (2015). These taxa were said to have extensive lateral wings of the entoplastron (Fig. S1.24I) that are different from the moderate wings of *Chelonia* (Fig. S1.24F) or the small wings of *Puppigerus* or *Caretta* (Fig. S1.24F–G). Furthermore, the same morphology is present in *Calcarichelys gemma* (Hooks 1998). However, it seems that these lateral wings result from fusion of the entoplastron with the epiplastra (e.g. Hirayama 1994; Tong *et al.* 2006). Evidence for this hypothesis comes from several taxa, in which the entoplastron and epiplastra are preserved and present as separate elements, but in which these elements in combination form a shape that is identical to that present in *Archelon*, *Protostega* and *Calcarichelys*. Examples for this include *Chelosphargis advena* (Zangerl 1953*a*) and *Rhinochelys nammourensis* (Tong *et al.* 2006). The epiplastra of these taxa are expanded into wing-like processes at their lateral ends, but the entoplastron itself is very similar to some cheloniids (such as *Chelonia mydas*), in which the lateral entoplastron processes are moderately expanded and a long posterior process is present. Therefore, we scored *Chelosphargis advena* and *Rhinochelys nammourensis* as having state 2 for character 246. In *Archelon*, *Protostega* and *Calcarichelys*, in which the entoplastron and epiplastra are fused to an entepiplastron, the exact shape of the entoplastron part of the entepiplastron is not discernable due to the absence of sutures. However, the posterior parts of the entepiplastra of *Archelon*, *Protostega* and *Calcarichelys* are indistinct from the entoplastra of other chelonioids, including *Caretta, Chelonia, Rhinochelys* and *Chelosphargis*. Thus, we think it is justified to merge the previously distinct states for 'T-shaped' entoplastra into a single state. However, we added a new character that considers the fusion of the entoplastron with the epiplastra so that the morphological similarity of *Calcarichelys*, *Protostega* and *Archelon* is still considered in our matrix:

> **Character 247 (new):** <u>Entepiplastron</u>: 0 = absent, entoplastron and epiplastra are separate elements; 1 = present, entoplastron is fused with epiplastra, resulting in a laterally extremely expanded entepiplastron wings.

Furthermore, the laterally expanded epiplastra, which are present in a number of protostegids irrespective of whether they are fused to the entoplastron or not, such as *Rhinochelys nammourensis*, *Chelosparhis advena* or *Protostega gigas*, need to be considered as a separate character, because the similarity between the epiplastra of the these taxa is not encoded in previous studies. Therefore, we modified the scorings and definition of character 244 of Evers & Benson (2018*a*) (character 249 of this study; character 160 of Cadena & Parham 2015). This character had a state (state 2) that described the shape of the epiplastra for *Archelon* and *Protostega*, which were the only taxa contained within in the matrices of Cadena & Parham (2015) and Evers & Benson (2018*a*) scored with this state. According to our observations, *Archelon ischryos*, *Protostega gigas*, *Rhinochelys nammourensis*, *Chelospargis advena*, *Calcarichelys gemma* and *Sanatanachelys gaffneyi* have the same epilastron shape. The condition is unclear in *Desmatochelys lowii*, as the lateral margins of the epiplastra are broken (Zangerl & Sloan 1960). We further modified the definition of this character to encode variation in epiplastron shape only. Previously, this character included observations on the contact of the epiplastra with one another, which is already encoded in a different character (character 239 of Evers & Benson 2018*a*; character 155 of Cadena & Parham 2018; character 244 of this study). Besides the changes described above, we added an additional state that distinguishes 'elongate' epiplastron morphologies. In previous matrices (e.g. Cadena & Parham 2015; Evers & Benson 2018*a*), the vast majority of taxa was scored as having 'elongate' epilastra, despite considerate variation between, for example, the 'elongate' epiplastra of cheloniids and chelydrids on one hand, and trionychids on the other (Fig. S1.25B, D).

Character 249: <u>Epiplastra, shape</u>: 0 = epiplastra squarish in shape and forming parts of the anterior plastral lobe; 1 = epiplastra elongate, become narrower posteriorly along the anterolateral margin of the hyoplastron, and with gently convex lateral margin; 2 = epiplastra are laterally strongy expanded to a wing-like shape; 3 = epiplastra rod-like and anteriorly as narrow as posteriorly, with concave lateral margin.

Previous character definition: Character 244 (Evers & Benson 2018*a*); character 160 (Cadena & Parham 2015). Epiplastra, shape and contact of epiplastra: 0 =

epiplastra squarish in shape, lack a contact between each other due to the narrow participation of the antoplastron in the anterior plastral lobe edge; 1 = epiplastra elongate in shape, with medial contact located anterior to the entoplastron; 2 = epiplastra squarish in shape, lack of medial contact due to the extensive anterior projections of the entoplastron.

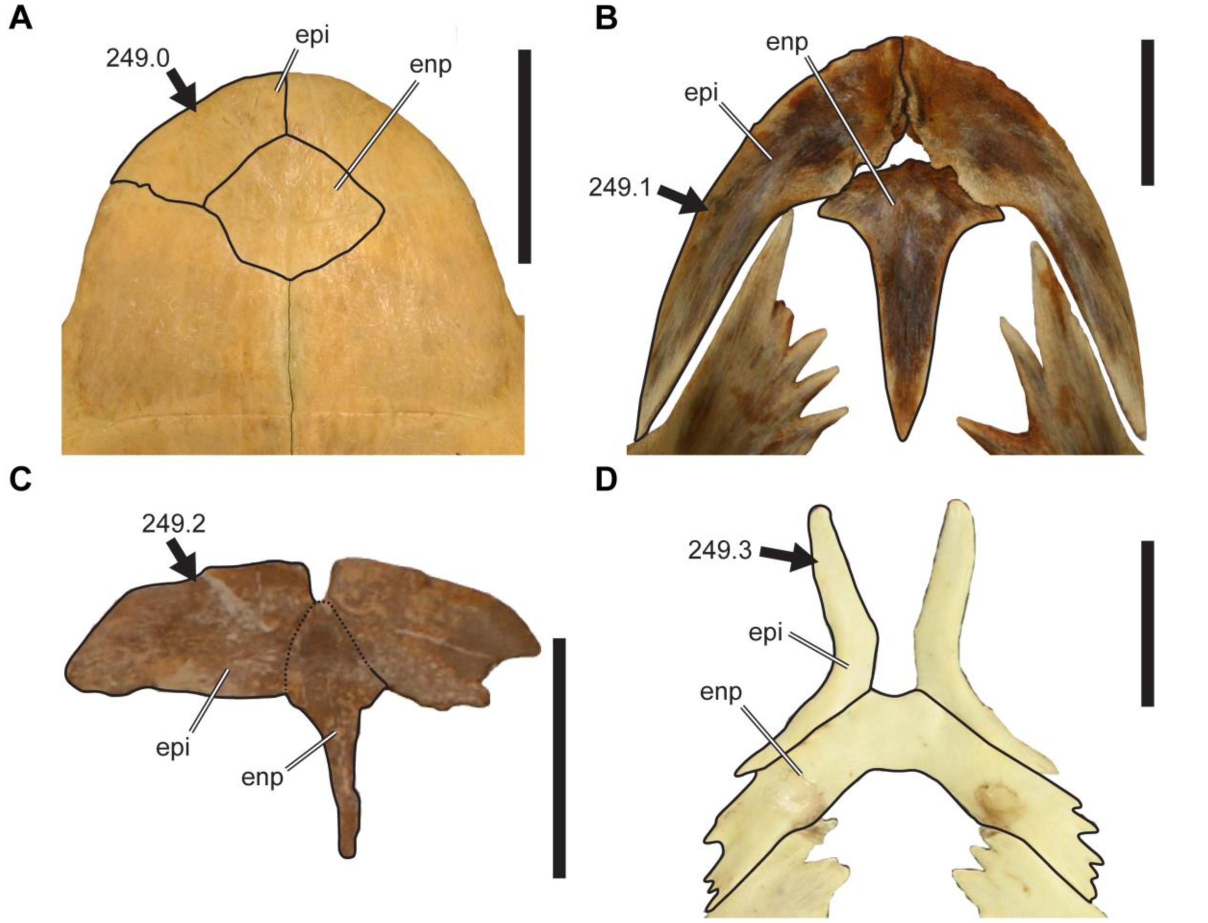

Fig. S1.25. Epiplastra of selected turtles. **A**, ventral view of *Podocnemis sextuberculata* (NHMUK 16075); **B**, ventral view of *Caretta caretta* (AMNH 129869); **C**, ventral view of *Chelosphargis advena* (KUVP 1219); **D**, ventral view of *Pelodiscus sinensis* (USNM 539335). Scale bars in A–B equal 5 cm, scale bar in D equals 2 cm. Scale bar in C equals approximately 3 cm, as the specimen dimensions were estimated from Zangerl (1953). Numbers represent characters and character states. Abbreviations: *enp*, entoplastron; *epi*, epiplastron.

Xiphiplastron characters. In extant chelonioids, as well as some fossil taxa (e.g. *Allopleuron, Eopshargis*), the xiphiplastra are anteroposteriorly elongate rods that are separated from each other by a posterior fontanelle and have no midline contact (Fig. S1.26D). This state has commonly been coded for all chelonioids (e.g. *Puppigerus*: see Cadena & Parham 2015). However, examination of fossil chelonioids shows that the xiphiplastra in many fossil sea turtles vary substantially from the condition observed in

modern forms. For example, in *Puppigerus* (Fig. S1.26B), *Toxochelys* and Early Cretaceous protostegids such as *Santanachelys*, the xiphiplastra are plate-like structures that articulate with one another over their entire anteroposterior length and form a posterior plastral lobe. In other species, such as *Eochelone* (Fig. S1.26C), the plastral lobe is still present, but the xiphiplastra are separated from one another anteriorly and are more elongate and thus more similar to those of modern chelonioids, despite being not quite as narrow as in *Caretta* (Fig. S1.26D). However, all chelonioids share an oblique suture of the xiphilastron with the hypoplastron, whereby the xiphiplastron has an anterolateral process that extends along the posterolateral margin of the hypoplastron. Some taxa, such as Late Cretaceous protostegids (*Desmatochelys*, *Archelon*, *Protostega*), have rod-like xiphiplastra similar to those of extant chelonioids, but they are sutured to each other posteriorly along the midline. To capture this variability, we coded three characters that describe distinct homologous features: the general shape of the xiphiplastron (character 260, plate like vs. rod-like); the nature of the suture with the hypoplastron (character 261, horizontal vs. oblique); and the inter-xiphiplastron contact resulting in a posterior plastral lobe (character 262, present vs. absent). We deleted the previous xiphiplastron shape character (character 255 of Evers & Benson 2018*a*; character 169 of Cadena & Parham 2015).

Character 260: <u>Xiphiplastra, shape of xiphiplastra</u>: 0 = triangular, trapezoidal, or rectangular plate-like element; 1 = anteroposterior elongate rods.

Character 261: <u>Xiphiplastra, articulation with hypoplastron</u>: 0 = the xiphiplastra articulate with the hypoplastra along an anteriorly facing margin, forming a mediolaterally broad suture; 1 = the xiphiplastra have an elongate anterolateral process articulating along the posterolateral margin of the hypoplastron, resulting in an oblique suture, and the hypoplastra extend posteriorly along the anteromedial margin of the xiphiplastra.

Character 262: <u>Xiphiplastra, posteriorly in contact with one another, often sutured along the midline and forming a plastral lobe</u>: 0 = present; 1 = absent.

Previous character definition: Character 255 (Evers & Benson 2018*a*); character 169 (Cadena & Parham 2015): character 169). Xiphiplastra, shape of xiphiplastra:

0 = almost triangular to trapezoidal, with lateral straight to convex margin; 1 = rectangular elongated in shape, coupled forming together with the hypoplastron a very narrow posterior plastral lobe; 2 = narrow struts, separacted by the posterior fontanelle.

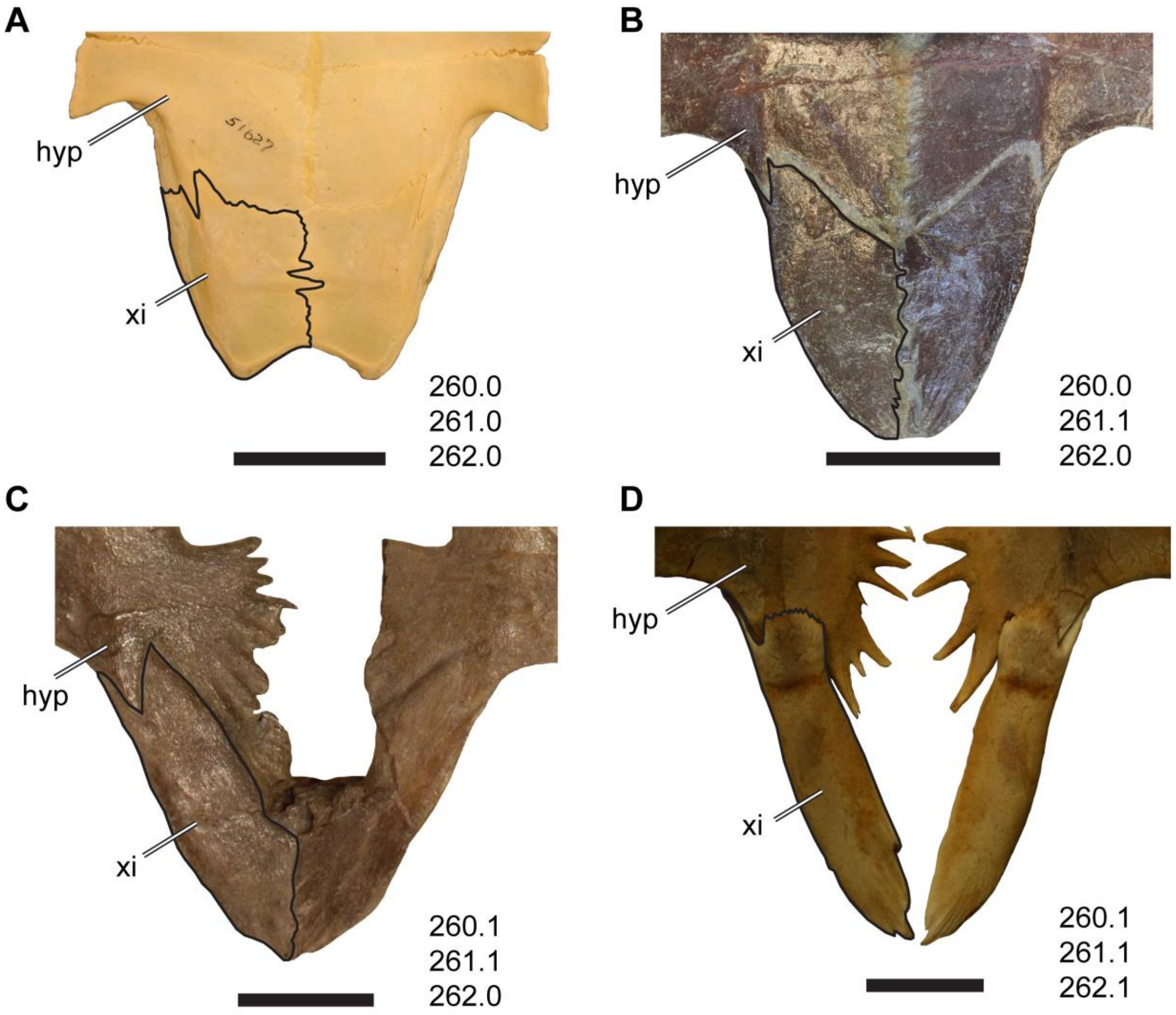

FIG. S1.26. Ventral view of posterior part of plastron of selected turtles. **A**, *Platysternon megacephalum* (FMNH 51627); **B**, *Puppigerus camperi* (IRSNB R 0073); **C**, *Eochelone brabantica* (IRSNB R 0061); **D**, *Caretta caretta* (AMNH 129869). Scale bar in A equals 3 cm, all other scale bars equal 5 cm. Black outlines denote the shape of the right xiphiplastron. Numbers represent characters and character states. Abbreviations: *hyp*, hypoplastron; *xi*, xiphiplastron.

Plastral serrations. Evers & Benson (2018*a*) simplified a complex multistate character regarding the configuration of plastral fontanelles, the hyo-hypolastron contact and the axillar and inguinal notches that had been proposed by Cadena & Parham (2015: character 153) into a series of separate characters (characters 235–237 of Evers & Benson 2018*a*). However, during our review of the scorings for chelonioids, we observed additional variation concerning the hyo- and hypoplastron serrations, which warrant a further modification to character 236 of Evers & Benson (2018*a*).

Serrated margins to the hyo- and hypoplastra are variably developed among turtles and are present in multiple groups, including trionychians, some thalassochelydians and chelonioids. To better capture the degree of variation in serrations, we added an additional state to our character 241 (= character 236 of Evers & Benson 2018*a*) that captures the hyo- and hypoplastron shape of most chelonioids (Fig. S1.27C) and most thalassochelydians with serrations (Fig. S1.27B). These taxa have strong serrations, which are limited to the anterolateral, anteromedial, posterolateral and posteromedial corners of the hyo- and hypoplastra. 'Star-shaped' hyo- and hypopastra with extreme serration along their almost entire bone margins are present in some protostegids such as *Archelon* and *Protostega* (Fig. S1.27D). We scored *Allopleuron hofmanni* as state 1, because despite its extremely long hyo- and hypoplastral serrations these are limited to those margins facing the opposite hyo- and hypoplastra, the entoplastron, xiphiplastra and the peripherals (e.g. Mulder 2003). The chelonioids *Puppigerus* (Fig. 39A) and *Eochelone* are scored as having state 0, because their serrations are only very weakly developed.

Character 241: <u>Plastron, hyo-hypoplastra serrations</u>: 0 = serrations on the lateral and medial margins absent or weakly developed; 1 = strong serrations present along the surfaces that face other bones, but serrations are absent along the margin of the central fontanelle and the lateral contact area of hyo- and hypoplastro; 2 = strong serrations along all margins but the anterolateral margin of the hyoplastra and the posterlateral margin of the hypoplastra present, giving these elements a 'star-shaped' appearance.

*Previous character definition***:** Character 236 (Evers & Benson 2018*a*), which was modified from character 153 of Cadena & Parham (2015). Plastron, hyo-hypoplastra serrations: 0 = serrations on the lateral and medial margins absent or weakly developed; 1 = strong serrations along medial and lateral margins present.

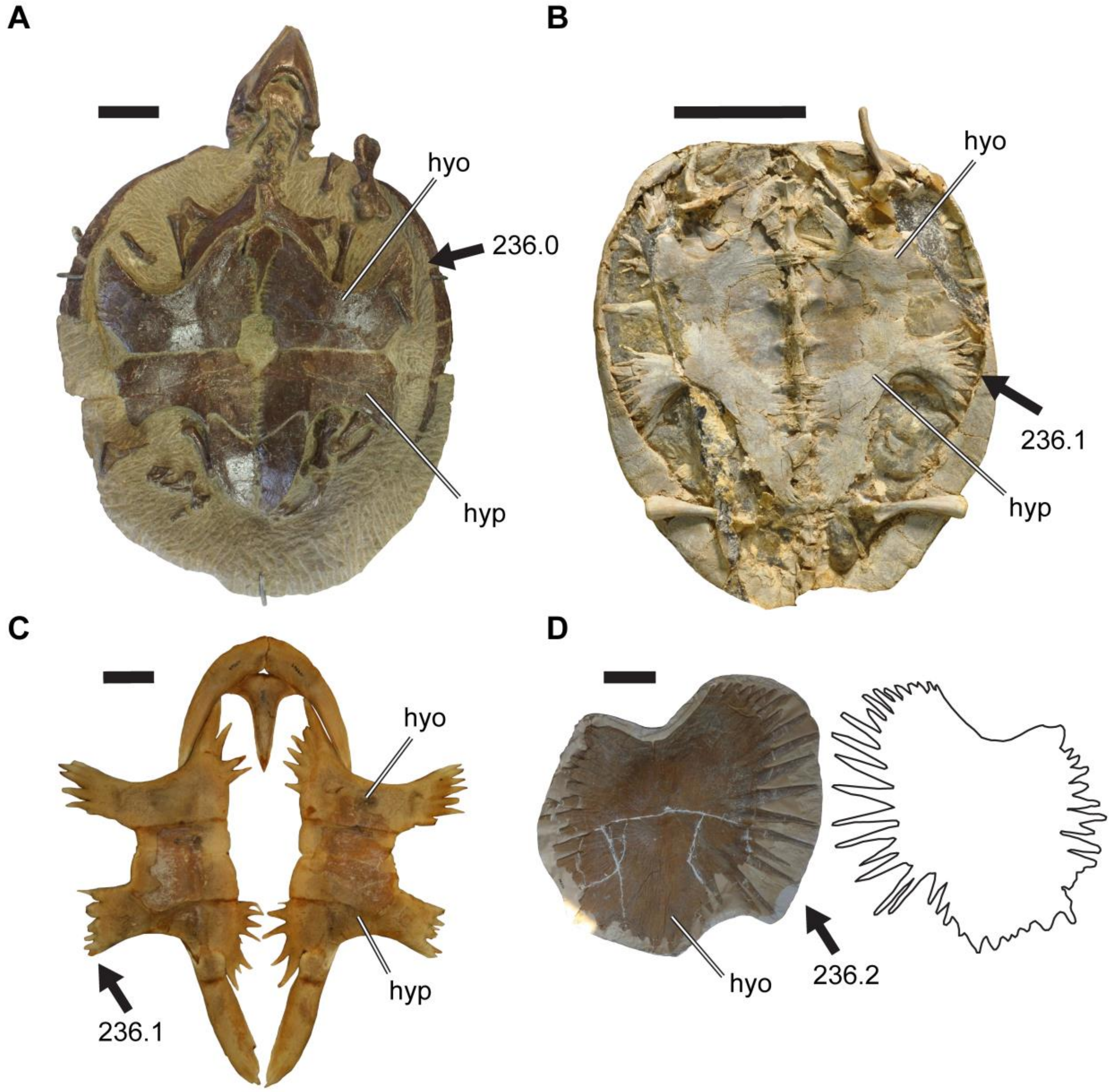

FIG. S1.27. Ventral view of plastral elements of selected turtles. **A**, ventral view of plastron of *Puppigerus camperi* (IRSN B 0073); **B**, ventral view of plastron of *Eurysternum wagleri* (CM 3409); **C**, ventral view of plastron of *Caretta caretta* (AMNH 129869); **D**, ventral view of right hyoplastron of *Protostega gigas* (FMNH PR 680). Note that outline of left hyoplastron in D is mirrored from the right element and added to give a better overview of the partial plastral arrangement. Numbers represent characters and character states. Scale bar in D is 10 cm, all other scale bars equal 5 cm. Abbreviations: *hyo*, hyoplastron; *hyp*, hypoplastron.

Humerus characters. The matrix of Evers & Benson (2018*a*) adopted six characters (characters 325–330) from Cadena & Parham (2015: characters 237–242) that captured variation in the lateral processes of the humerus. However, as noted by previous authors (e.g. Bardet *et al.* 2013), some widely-used characters regarding humerus morphology have not been completely explained (e.g. Hirayama 1998; Parham & Pyenson 2010; Bardet *et al.* 2013). Most originated with the observations of Hirayama (1994), who coded a series of characters to capture what he called the 'cheloniid',

‘dermochelyid’ and ‘protostegid’ humeri. A conspicuous feature of chelonioid humeri is the position and shape of the lateral process, which in turtles is usually a rounded, knob-like process on the anterior surface of the proximal humerus (Fig. S1.28A, C) that is separated from the humeral head by a small notch or a bony ridge termed the preaxial ‘shoulder’ (Gaffney 1990). However, in chelonioids the lateral process is positioned more distally along the humeral shaft (Fig. S1.28D–G) and is therefore separated from the proximal end of the humerus, and the preaxial shoulder is either absent, or relatively low. The shift in position of the lateral process is apparent in the stem-group chelonioid *Toxochelys*, in which the lateral process is only moderately distally removed from the humeral head, but which retains a preaxial shoulder (e.g. Hirayama 1992; SWE, pers. obs. of FMNH P27403; Fig. S1.28D).

In cheloniids, including Creteacous taxa such as *Allopleuron hofmanni* (e.g. Mulder 2003) and most protostegids (e.g. ?*Chelosphargis advena*: AMNH FARB 1975; *Desmatochelys padillai*: Cadena & Parham 2015), the lateral process is positioned further distally on the shaft of the humerus and the preaxial shoulder is absent. In *Dermochelys coriacea* (e.g. Völker 1913; Nielsen 1963; Hirayama 1992; Wyneken 2001), *Eosphargis breineri* (Nielsen 1963; pers. obs. SWE of FUR N 1450) and the gigantic Late Cretaceous protostegids from North America (e.g. *Archelon ischryos*: Wieland 1896; *Protostega gigas*: AMNH FARB 1503; Case 1897), the lateral process is positioned near mid-shaft (Fig. S1.28F–G). The lateral process is generally rounded (e.g. *Protostega*; Fig. 40F) and can be almost hemispherical. This is different in *Dermochelys* and *Eosphargis*, in which the lateral process tapers to form a distally recurved tip, a feature that is only observed in these two taxa among our taxon sampling (Fig. S1.28G). Whereas the above-mentioned morphological variation is encoded in characters 331 (preaxial shoulder; character 324 of Evers & Benson 2018*a*), 332 (position of lateral process; character 325 of Evers & Benson 2018*a*) and 333 (tip-like expansion of lateral process; character 330 of Evers & Benson 2018*a*), a number of additional characters (326–329 in Evers & Benson 2018*a*, originally taken from Cadena & Parham 2015) have been used previously to express shape variation of the lateral process. However, we could not understand the characters or reproduce the scorings of previous authors based on our literature research and personal examination of specimens. Also, we think that variation in humerus morphology is adequately captured in the remaining characters. Therefore, we deleted characters 326–329 of Evers & Benson (2018*a*) from our matrix. The only characters pertaining to the lateral process of the humerus used in this study are:

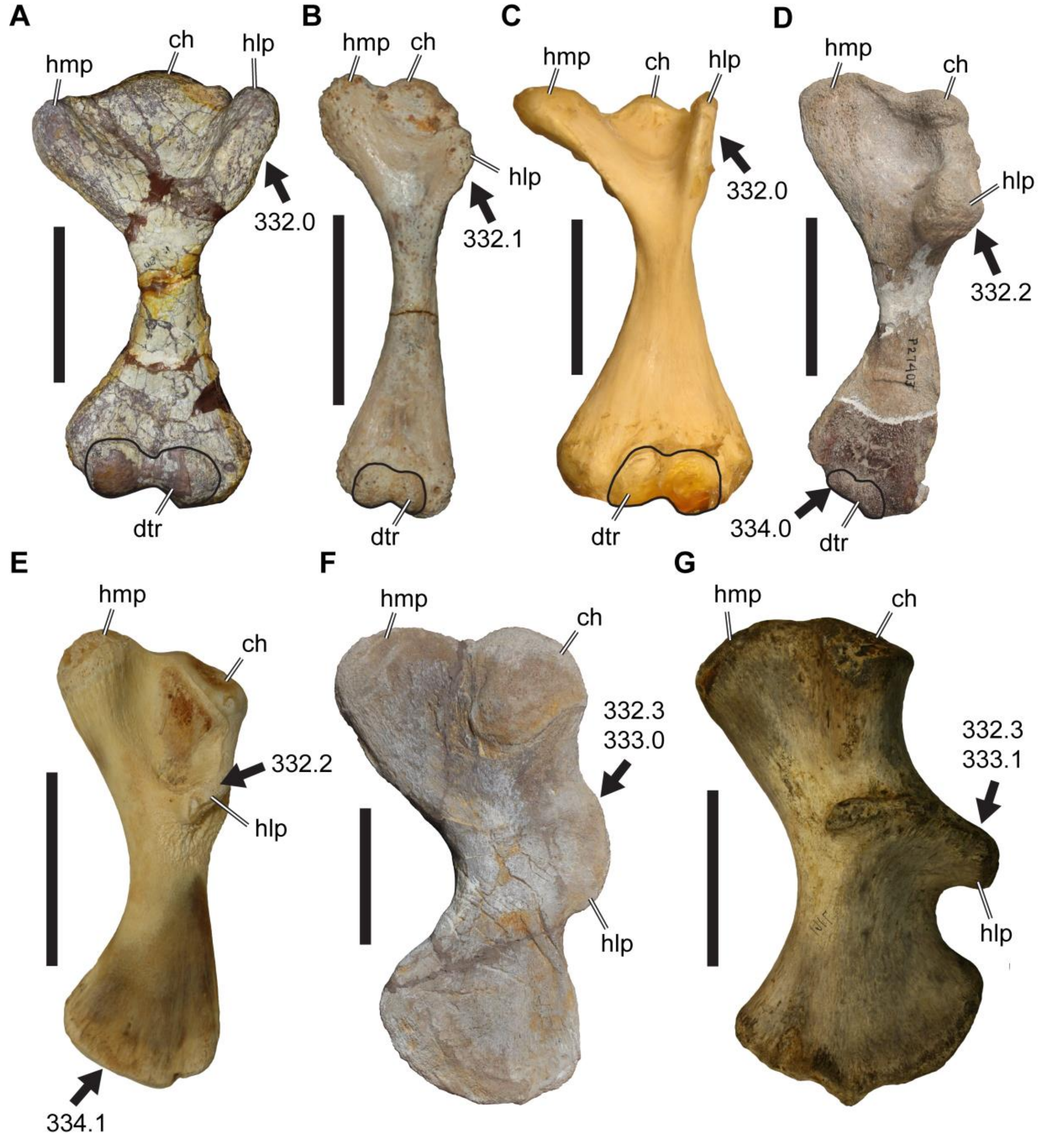

FIG. S1.28. Humeri of selected turtles. **A**, right humerus of *Proganochelys quenstedti* (SMNS 16980) in ventral view, reflected for comparison; **B**, left humerus of *Sinemys gamera* (IVPP unnumbered) in ventral view; **C**, right humerus of *Chelydra serpentina* (FMNH 22056) in ventral view, reflected for comparison; **D**, left humerus of *Toxochelys* sp. (FMNH P27403) in ventral view; **E**, right humerus of *Caretta caretta* (AMNH 129869) in ventral view, reflected for comparison; **F**, left humerus of *Protostega gigas* (FMNH UR80) in ventral view; **G**, right humerus of *Dermochelys coriacea* (AMNH 7161) in ventral view, reflected for comparison. Scale bars in A, D, E–F equal 5 cm, scale bar in B equals 2 cm, scale bar in C equals 3 cm, scale bar in G equals 10 cm. Note that distal trochleae are outlined in A–D. Numbers represent characters and character states. Abbreviations: *ch*, humeral head; *dtr*, distal trochlea; *hlp*, lateral process of humerus; *hmp*, medial process of humerus.

Character 332: Humerus, lateral process of humerus: 0 = abuts caput humeri; 1 = slightly separated from caput humeri; 2 = located distal to caput humeri but along proximal end of shaft; 3 = located at middle of humeral shaft.

Character 333: Humerus, prominent anterior projection of lateral process: 0 = absent; 1 = present.

We coded an additional humeral character that has, to our knowledge, not been discussed previously: in crown-group chelonioids, as well as a range of fossil chelonioids, the distal surface of the humerus lacks a distinct trochlea for articulation with the antebranchium (Fig. S1.28E–G), whereas non-chelonioid turtles (Fig. S1.28A–C), as well as some chelonioids (e.g. *Toxochelys*: Fig. S1.28D; *Erquelinnesia*: IRSNB R 0067), have a distinct trochlea (Fig. S1.28A–B).

Character 334 (new): Humerus, distal articulation: 0 = articular surface forms distinct trochlea; 1 = rounded epiphyseal surface without clearly defined articulation facets.

Manus characters. Tong *et al.* (2006) discussed variation in the structure of the manus among chelonioids, but most of this variation has never been coded as phylogenetic characters. We add new characters to encode this variation here. To illustrate the manus morphology of chelonioids, as well as non-chelonioid turtles, we provide summary illustrations in Figs S1.29 and S1.30.

Tong *et al.* (2006) noticed that the ulnare of protostegids is much larger than the intermedium, whereas both these proximal carpals are subequally sized in dermochyids and cheloniids. This character was already included in previous studies and is retained herein as character 344 (character 340 of Evers & Benson 2018*a*; character 251 of Cadena & Parham 2015). We also include a new character that compares the sizes of the proximal carpals to the distal carpals, as that these bones are subequal in size in the stem-chelonioid *Toxochelys* (Fig. S1.30B), whereas the proximal carpals are generally larger than the distal ones in other chelonioids, including protostegids (Fig. S1.30C–F).

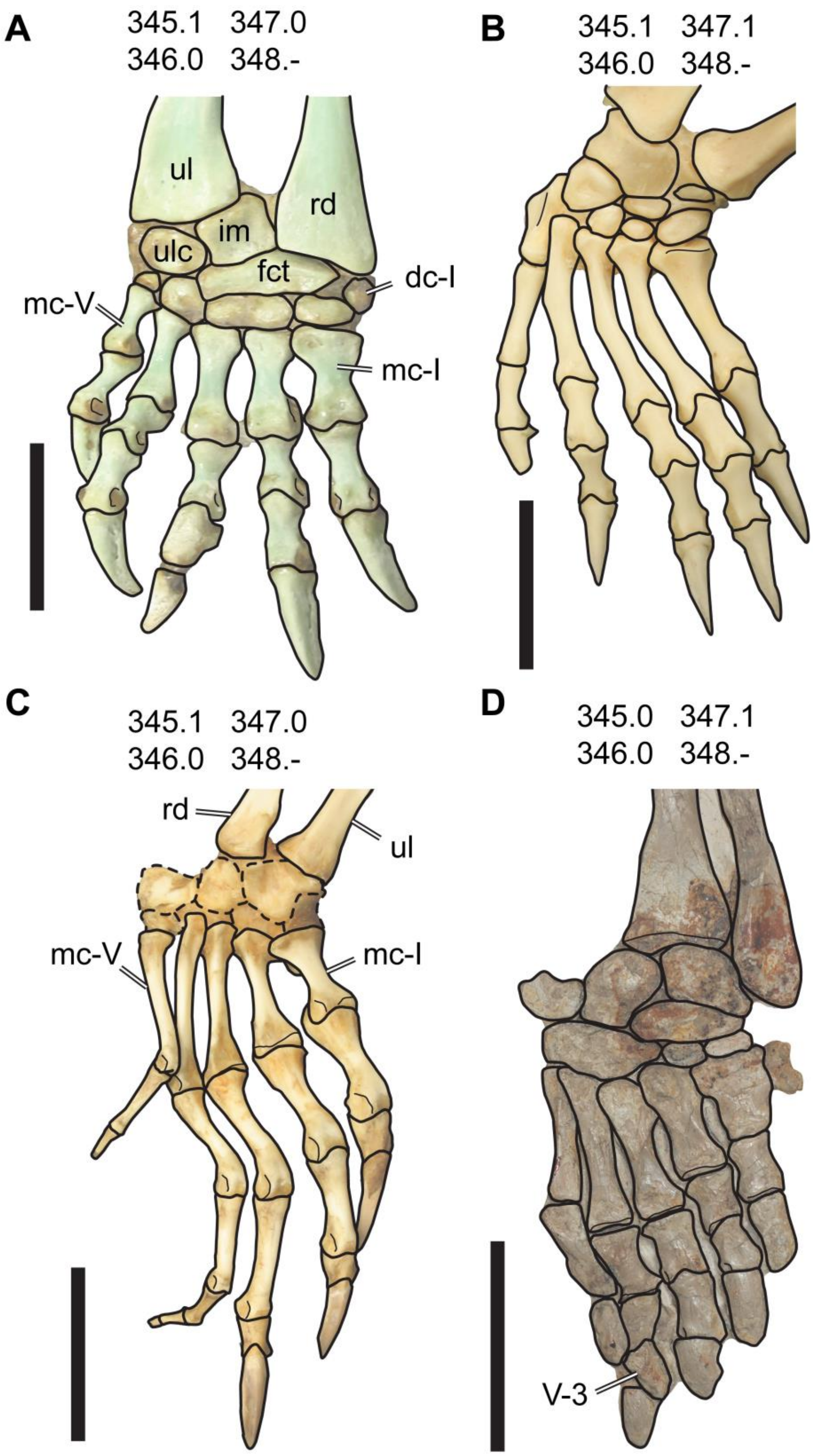

FIG. S1.29. Hands of selected non-americhelydian turtles. **A**, right manus of *Emys blandingii* (USNM 220869); **B**, right manus of *Chelus fimbriatus* (USNM 64154); **C**, right manus of *Apalone ferox* (USNM 71069); **D**, right manus of *Palaeomedusa testa* (BSPG AS I 818). Scale bar in A equals 10 mm, scale bars in B–D equal 30 mm. Note that Roman letters denote digits and Arabic numbers denote phalangeal position. Numbers represent characters and character states. Abbreviations: *dc*, distal carpal; *fct*, fused centralia; *im*, intermedium; *mc*, metacarpal; *pi*, pisiform; *rd*, radius; *ul*, ulna; *ulc*, ulnare.

Character 345 (new): Size of proximal carpals vs. distal carpals: 0 = proximal carpals are of similar size with respect to distal carpals; 1 = proximal carpals are much larger than distal carpals.

Tong *et al.* (2006) noticed various differences in the digits of the chelonioid subgroups, (i.e. the dermochelyids, protostegids and cheloniids). For example, the second phalanx of the 3rd and 4th digits is long compared to the 1st phalanx in cheloniids (Fig. S1.30D–E), but not in protostegids (Fig. S1.30C), dermochelyids (Fig. S1.30F) or non-chelonioid americhelydians (Fig. S1.30A). Furthermore, protostegids have a third manual phalanx on their fifth digit (Fig. 42C), resulting in a manus phalangeal formula of 2-3-3-3-3 (Tong *et al.* 2006). The V-3 is absent in dermochelyids (Fig. S1.30F) or cheloniids (Fig. S1.30D–E), but present in *Toxochelys* and extant and extinct chelydroids (e.g. Fig. S1.30A). Another observation is that the 4th digit is the longest in protostegids and *Toxochelys* (Fig. S1.30B–C), whereas it is the 3rd in dermochelyids (Fig. S1.30F) and cheloniids (Fig. S1.30D–E). We included these observations as three characters:

Character 346 (new): Relative lengths of manual phalanges on the 3rd and 4th digit: 0 = the 1st phalanx is longer than or equally long as the 2nd phalanx; 1 = the 2nd phalanx is longer than the 1st phalanx. This character is scored as inapplicable when the manus digits only have two phalanges (i.e. the second phalanx is an ungual).

Character 347 (new): 3rd phalanx on 5th manual digit: 0 = absent; 1 = present.

Character 348 (new): Longest digit in the manus: 0 = 4th digit; 1 = 3rd digit. This character is scored as inapplicable when the 3rd and 4th digits are equally long.

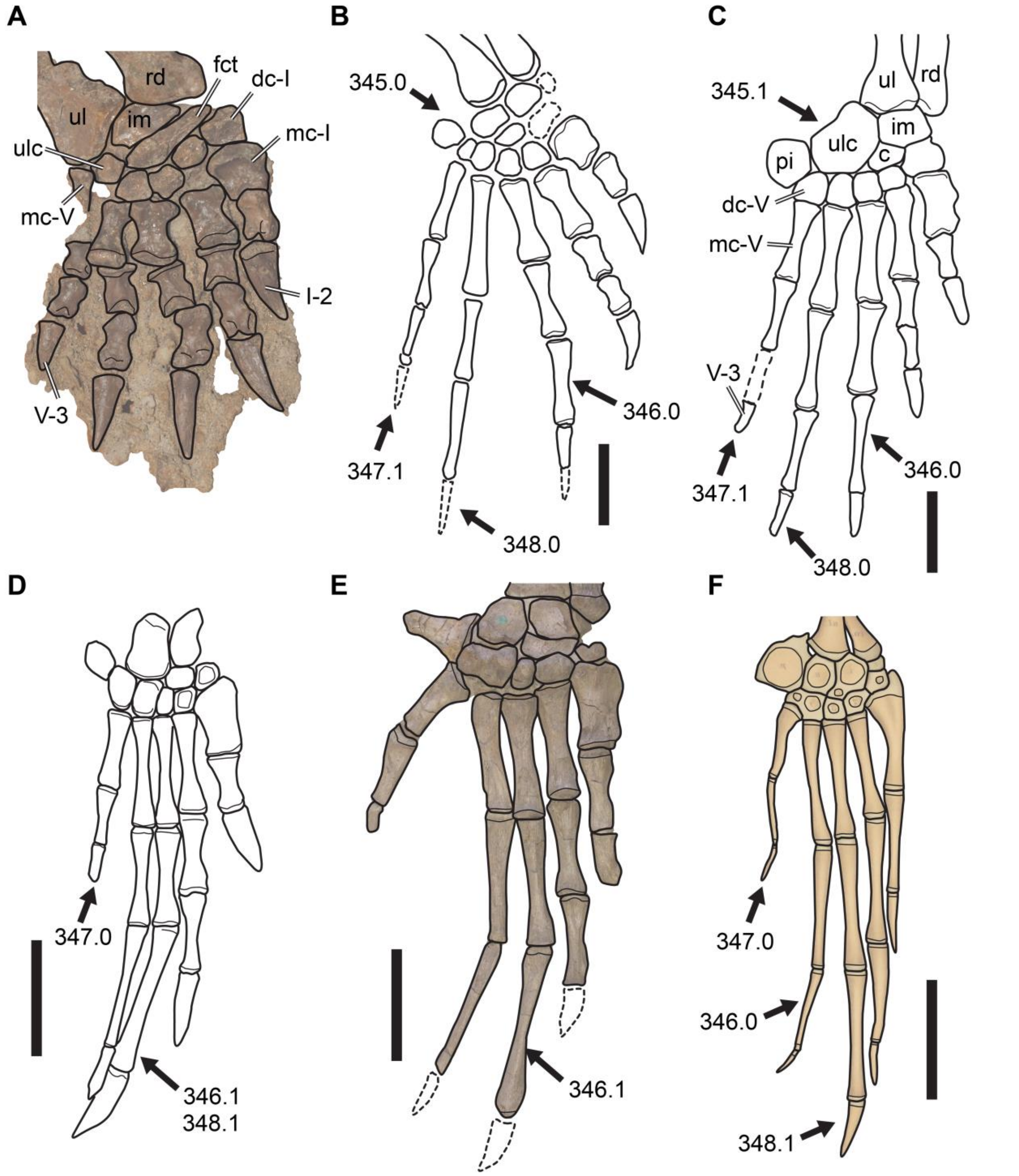

FIG. S1.30. Hands of selected americhelydian turtles. **A**, left manus of *Emarginchelys cretacea* (KUVP 23488), reflected for comparison; **B**, left manus of *Toxochelys latiremis* (YPM 2491), reflected for comparison and re-drawn from Wieland (1902); **C**, right manus *Protostega gigas* (CMNH 1421), re-drawn from Wieland (1906); **D**, left manus of *Eretmochelys imbricata* (MNHN Pal 1934-563), reflected for comparison; **E**, right manus of *Allopleuron hofmanni* (NHMUK PV 42893); **F**, left manus of *Dermochelys coriacea* (unnumbered), reflected for comparison and re-drawn from Völker (1913). Scale bar in A approximates 3 cm (estimated from Whetstone 1978); scale bar in B approximates 5 cm (estimated from Wieland 1902); scale bar in C approximates 10 cm (estimated from Wieland 1906); scale bar in D equals 3 cm, scale bar in E equals 10 cm; scale bar in F approximates 20 cm (estimated from Völker 1913). Note that Roman letters denote digits and Arabic numbers denote phalangeal position. Numbers represent characters and character states. Abbreviations: *c*, centrale; *dc*, distal carpal; *fct*, fused centralia; *im*, intermedium; *mc*, metacarpal; *pi*, pisiform; *rd*, radius; *ul*, ulna; *ulc*, ulnare.

Femur characters. The only femoral character in the matrix of Evers & Benson (2018*a*: character 344; taken from Cadena & Parham 2015: character 255) was originally taken from Hirayama's (1998; character 79) chelonioid phylogeny. The character reflects the webbing between the femoral trochanters in chelonioids, which is generally absent in non-chelonioid turtles, in which the trochanters are distinct processes that are not interconnected. However, in the previous character, variation in the depth of the intertrochanteric ridge was considered in the same character as the absence of the ridge. Here, we separate these observations into individual characters:

Character 352: Femur, femoral trochanters: 0 = distinct, and separated from one another; 1 = connected by a ridge.

Character 353: Femur, intertrochanteric ridge: 0 = ridge is low and concave, creating a notch between the major and minor trochanter; 1 = ridge is high and obliterates intertrochanteric notch, and the proximal surface of the trochanters and their connecting ridge forms a continuous surface. This character is scored as inapplicable when an intertrochanteric ridge is absent (character 352.0)

Previous character definition: Character 344 (Evers & Benson 2018*a*); character 255 (Cadena & Parham 2015). Femur, femoral trochanters: 0 = distinct, and separated from one another; 1 = fossa obliterated, space between trochanters not concave, but notch present; 2 = fossa obliterated, trochanters connected by bony ridge without a notch.

In addition, we observed that the major trochanter and the femoral head form a continuous surface primitively in turtles like *Proganochelys quenstedti* (Fig. S1.31A, D) and also in crown group sea turtles (Fig. S1.31I, L) and some fossil chelonioids (Fig. S1.31H, K), but not in the stem-group chelonioid *Toxochelys* (Fig. 43G, J) or non-chelonioid crown-group turtles (Fig. S1.31B, E). We coded these observations in a separate character:

Character 354 (new): Femur, connection between femoral head surface and the major trochanter: 0 = the femoral head and major trochanter have distinct

proximal surfaces separated by a deep notch; 1 = the femoral head surface slopes toward the major trochanter and forms a continuous proximal surface with it.

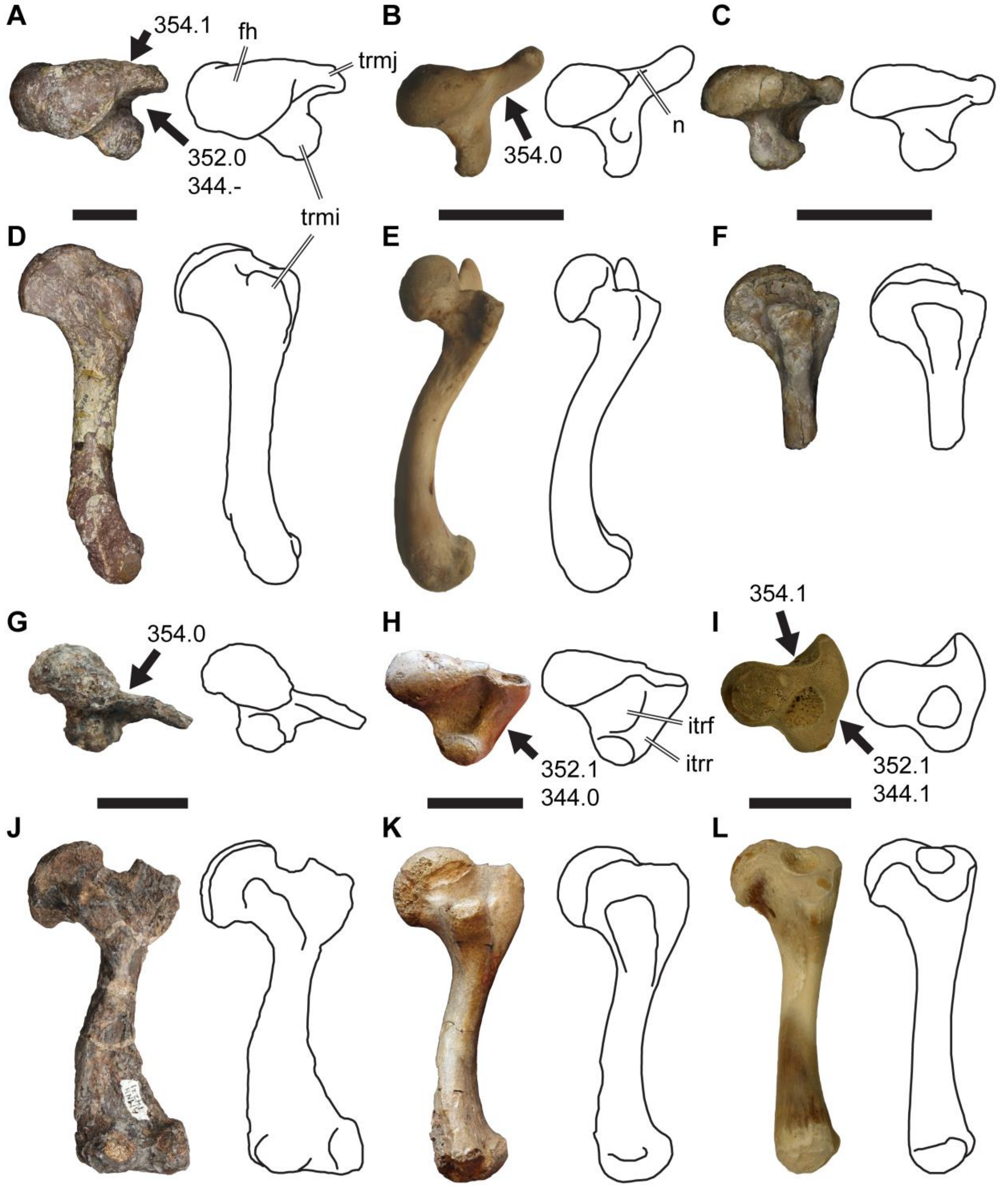

FIG. S1.31. Photographs and interpretative line drawings of femora in proximal (top row and third row) and anterior (second and bottom row). **A & D**, right femur of *Proganochelys quenstedti* (SMNS 16980); **B & E**, right femur of *Apalone spinifera* (YPM R190893); **C & F**, partial left femur of *Jurassichelon oleronensis* (PIMUZ AIII 514), reflected for comparisons; **G & J**, right femur of *Toxochelys browni* (AMNH FARB 14221); **H & K**, left femur of *Eochelone brabantica* (IRSNB R 0001), reflected for comparisons; **I & L**, right femur of *Caretta caretta* (AMNH 129869). All scale bars equal 3 cm. Abbreviations: *fh*, femoral head; *itrf*, intertrochanteric fossa; *itrr*, intertrochanteric ridge; *n*, notch between femoral head and major trochanter; *trmi*, minor trochanter; *trmj*, mahor trochanter.

SCORING SOURCES

TABLE S1.2. Sources for scorings of our character-taxon matrix.

Taxon	Specimen(s) used for character scoring	References used for character scoring	Personal observation of taxon	CT
Adocus lineolatus	**CCM 60-15**	Meylan & Gaffney 1989; Cadena & Parham 2015	no	yes
Allaeochelys libyca	**BSPG 1991 II 130**	Havlik, Joyce & Böhme 2014	yes	yes
Allopleuron hoffmanni	IRSNB R 0008, 3668; **NHMUK PV OR42913**	Mulder 2003	yes	yes
Angolachelys mbaxi	MGUAN-PA 002, 296	Mateus *et al.* 2009; Myers *et al.* 2018	no	no
Annemys sp.	**IVPP V18106**	Brinkman *et al.* 2013	no	yes
Annemys levensis	PIN 4636-4-2	Shukanov 2000; Rabi *et al.* 2014	no	no
Annemys latiens	PIN 4636-6-1	Rabi *et al.* 2014	no	no
Apalone spinifera emoryi	**FMNH 22178**	no additional references used	yes	yes
Araripemys barretoi	**AMNH FARB 30778**	Meylan 1996; Gaffeny *et al.* 2011	yes	yes
Archelon ischyros	YPM 3000	Wieland 1896, 1900, 1902	yes	no
Argillochelys cuneiceps	**NHMUK PV OR41636**	no additional references used	yes	yes
Arundelemys dardeni	**USNM 497740**	Lipka *et al.* 2006	no	yes
Australochelys africanus	**BP/1/4933**	Gaffney & Kitching 1995	yes	yes
Baptemys wyomingensis	YPM 374, 3758; DMNH 511	Meylan & Gaffney 1989; Knauss 2014	no	no
Bouliachelys suteri	**QMF 31669**	Kear & Lee 2006	no	yes
Brachyopsemys tingitana	AMNH FARB 30001; **AMNH FARB 30612**	Tong & Meylan 2013	yes	yes
Cabindachelys landanensis	**MGUAN-PA 298;** MRAC 4796	Myers *et al*. 2018	no	yes
Calcarichelys gemma	RMM 3216	Zangerl 1953; Hooks 1998; Cadena & Parham 2015	no	no
Caretta caretta	**NHMUK 1938.1.9.1**	no additional references used	yes	yes

Taxon	Specimen(s) used for character scoring	References used for character scoring	Personal observation of taxon	CT
Carettochelys insculpta	**NHMUK 1903.7.10.1**	no additional references used	yes	yes
Chelodina oblonga	**NHMUK 64.12.22**	no additional references used	yes	yes
*Chelodina longicollis**	unknown (specimen number not given in Cadena & Parham 2015)	Cadena & Parham 2015	no	no
Chelonia mydas	**NHMUK 1969.776**	no additional references used	yes	yes
Chelonoidis sp.	**SMF 67582**	no additional references used	no	yes
*Chelonoidis chilensis**	unknown (specimen number not given in Cadena & Parham 2015)	Cadena & Parham 2015	no	no
Chelosphargis advena	KUVP 1209	Zangerl 1953; Cadena & Parham 2015	no	no
Chelus fimbriatus	**NHMUK 81.9.27.4**	no additional references used	yes	yes
Chelydra serpentina	**SMF 32846**	no additional references used	no	yes
Chrysemys picta	**NHMUK 76.1.31.19**	no additional references used	yes	yes
Chubutemys copelloi	MPEF-PV1236	Gaffney et al. 2007; Sterli, de la Fuente & Umazano 2015	no	no
Corsochelys halinches	**FMNH PR 249**	Zangerl 1960	yes	yes
Ctenochelys sp.	**AMNH FARB 6137**, 234**; FMNH PR444, PR 1047**, PR97, P27352, P27337, P27366; MSC 35085; USNM 357166	Matzke 2007; Gentry 2016	yes	yes
Dermatemys mawii	**SMF 59463**	no additional references used	no	yes
Dermochelys coriacea	**FMNH 171756; UMZC R3031**	no additional references used	yes	yes
Desmatochelys lowii	**KUVP 1200**	no additional references used	no	yes
Desmatochelys padillai	FCG-CBP 01, 13, 15, 39, 40; UCMP 38345, 382456	Cadena & Parham 2015	no	no
Dracochelys bicuspis	IVPP V4075; IVPP V12091	Gaffney & Ye 1992; Brinkman 2001	no	no
Eileanchelys waldmanni	**NMS.G.2004.31.15**; NMS.G.2004.31.16a-f	Anquetin 2010	yes	yes
Elseya dentata	**NHMUK 76.5.19.77**	no additional references used	yes	yes
Emarginachelys cretacea	KUVP 23488	Whetstone 1978	no	no
Emys orbicularis	**WGJ1987a**	no additional references used	no	yes
Eochelone brabantica	**IRSNB R0001, 0061, 0062;** IRSNB NIG 8475, 18733**; NHMUK PV**	Casier 1968	yes	yes

Taxon	Specimen(s) used for character scoring	References used for character scoring	Personal observation of taxon	CT
	**OR37213**			
Eosphargis breineri	FUM-N-1450	Nielsen 1959, 1963	yes	no
Eretmochelys imbricata	**FMNH 22242**	no additional references used	yes	yes
Eubaena cephalica	**DMNH 96004**; AMNH FARB 4948, 2602, 2604, 2606; YPM 1785	Gaffney 1972, 1979; Gaffney 1982*a*, *b*; Rollot, Lyson & Joyce 2018	no	no
Erquelinnesia gosseleti	IRSNB R1563, 0067–68, 0070–71, 1642–43, 1650	Zangerl 1971	yes	no
Galianemys whitei	**AMNH FARB 29987**	Gaffney, Tong & Meylan 2002, 2006	yes	yes
Geoclemys hamiltoni	**NHMUK 87.9.30.1**	no additional references used	yes	yes
Glyptops plicatulus	AMNH FARB 336; YPM 1784, 4717, 5821	Gaffney 1979	no	no
Gopherus polyphemus	**FMNH 211815**	no additional references used	yes	yes
Judithemys sukhanovi	TMP 87.2.1	Parham & Hutchinson 2003	no	no
Jurassichelon oleronensis	**PIMUZ AIII 514**	Rieppel 1980	yes	yes
Kallokibotion bajazidi	NHMUK PV R4918; **NHMUK PV R4921**; **NHMUK PV R4925**	Gaffney & Meylan 1992	yes	yes
Kayentachelys aprix	**TMM 4370-2,** 43653-1; MCZ 8914-8917; MNA V1558, V2664	Sterli & Joyce 2007; Gaffney & Jenkins 2010	no	yes
Kinosternon suburum hippocrepis	**FMNH 211711**	no additional references used	yes	yes
*Kinosternon flavescens**	unknown (specimen number not given in Cadena & Parham 2015)	Cadena & Parham 2015	no	no
Kirgizemys dmitrievi	ZIN PH7/15	Danilov *et al*. 2006	no	no
Kirgizemys hoburensis	PIN 3334-4; PIN 3334-35; PIN 3334-36	Sukhanov 2000	no	no
Lepidochelys kempii	**M009/08**	Jones *et al*. 2012	no	yes
Lepidochelys olivacea	**SMNS 11070**	no additional references used	yes	yes
Levyachelys cipadi	FCG-CBP-71; SMU 75377, 72852, 74982, 75327; FWMSH 93B-17	Vineyard 2009; Cadena 2015	no	no
Lissemys punctata	SMF 74141	no additional references used	no	yes

Taxon	Specimen(s) used for character scoring	References used for character scoring	Personal observation of taxon	CT
Macrochelys temminckii	**FMNH 22111**	no additional references used	yes	yes
Meiolania platyceps	**NHMUK PV R682**; specimens listed in Gaffney 1983	Gaffney 1983	yes	yes
Natator depressus	**R112123**	Jones *et al.* 2012	no	yes
Nichollsemys baieri	**TMP 97.99.1**	Brinkman *et al.* 2006; Cadena & Parham 2015	no	yes
Notochelone costata	**NHMUK PV R9590**	no additional references used	yes	yes
Ocepechelon bouyai	OCPDEK/GE516	Bardet *et al*. 2013	no	no
Oligochelone rupelensis	IRSNB VERT 18733-03906	no additional references used	yes	no
Ordosemys sp.	**IVPP V12092**	Brinkman & Wu 1999	no	yes
Pelodiscus sinensis	**IW576-2**	no additional references used	no	yes
Pelomedusa subrufa	**SMF 70504**	no additional references used	yes	yes
Peritresius martini	ALMNH 6191	Gentry *et al.* 2018	no	no
Petrochelys kyrgyzensis	**ZIN-PHT-F67-218**	Vitek *et al.* 2018	no	yes
Phrynops geoffranus	**SMF 45470**	no additional references used	no	yes
Platysternon megacephalum	**SMF 69684**	no additional references used	no	yes
Plesiochelys bigleri	**MJSN TCH007-252**; specimens listed in Pünterer et al. 2017	Pünterer, Anquetin & Billot-Bruyat 2017	yes	yes
Plesiochelys planicpes	**OUMNH J1582**	Gaffney 1975*a*; Gaffney 1976	yes	yes
Plesiochelys etalloni	**MNB 435**; MAJ 2005-11-1	Gaffney 1975*a*; Gaffney 1976; Anquetin, Deschamps & Claude 2014	yes	yes
Pleurosternon bullockii	**UMZC T1041**	Evans & Kemp 1975	no	yes
Podocnemis unifilis	**FMNH 45657**	no additional references used	yes	yes
*Podocnemis expansa**	unknown (specimen number not given in Cadena & Parham 2015)	Cadena & Parham 2015	no	no
Portlandemys mcdowelli	**NHMUK PV R2914**	no additional references used	yes	yes
Procolpochelys	CCNHM 893, 300.1	Weems & Sanders 2014; Weems & Brown	no	no

Taxon	Specimen(s) used for character scoring	References used for character scoring	Personal observation of taxon	CT
charlestonensis		2017		
Proganochelys quenstedti	SMNS 15759, 16980	Gaffney 1990	yes	no
Protostega gigas	AMNH FARB 1502, 1503; AUMP 412; CMNH 1421; FMNH PR2, P27385;	Case 1897; Williston 1902; Wieland 1906; Hay 1908; Hooks 1998	yes	no
Puppigerus camperi	**IRSNB R0074–76;** IRSNB R0072–73, 0077, 0081**; NHMUK PV OR38955, R14375**	Moody 1974	yes	yes
Rhinochelys nammourensis	MSNM V3933; specimens listed in Tong *et al.* 2006	Tong *et al.* 2006; Cadena & Parham 2015	no	no
Rhinochelys pulchriceps	**CAMSM B55775, B55783, B55776; NHMUK PV OR43980, R2226, OR35197**	no additional references used	yes	yes
Sandownia harrisi	**MIWG 3480**	Meylan *et al*. 2000	yes	yes
Sinemys gamera	IVPP V9532-11	Brinkman & Peng 1993	no	no
Sinemys lens	IVPP V9533-1; IVPP V9533-3	Brinkman & Peng 1993	no	no
Solnhofia parsoni	**TM 4023;** JM SCHA 70	Gaffney 1975*b*; Joyce 2000	yes	yes
Staurotypus salvinii	**NHMUK 1879.1.7.5**	no additional references used	yes	yes
*Staurotypus triporcatus**	unknown (specimen number not given in Cadena & Parham 2015)	Cadena & Parham 2015	no	no
Sternotherus minor	**FMNH 211696**	Bever 2009	yes	yes
*Sternotherus odoratus**	unknown (specimen number not given in Cadena & Parham 2015)	Cadena & Parham 2015	no	no
Testudo marginata	**FMNH 51672**	no additional references used	yes	yes
*Testudo hermanni**	unknown	Cadena & Parham 2015	no	no
Toxochelys sp.	AMNH FARB 1496, **5118**; FMNH UR3; **FMNH PR219,** PR648	Hay 1896; Nicholls 1988	yes	yes
Xinjiangchelys radiplicatoides	**IVPP V9539-1**	Brinkman et al. 2013	no	yes
Xinjiangchelys wusu	PMOL-SGPA0100-1; PMOL-SGPA0100-3	Rabi *et al.* 2013	no	no

*Species indicated by asterisk were used only for postrcanial scores.

Bold specimen numbers indicate specimens for which CT scans were available.

CHARACTER OPTIMIZATION

Methods

Because character optimization should be carried out on a fully bifurcated tree, a single MPT was selected from all MPTs. To select an MPT for optimisation, we computed a 50% majority rule consensus tree from the MPTs gained from the original analysis (Fig. S1.32). The topology of this majority rule consensus tree was used as a constraint in PAUP* for Macintosh (Swofford 2002) to find all MPTs compatible with the majority consensus rule topology. One of these MPTs was chosen at random for the optimisation. The topology of this tree is shown in Fig. S1.33.

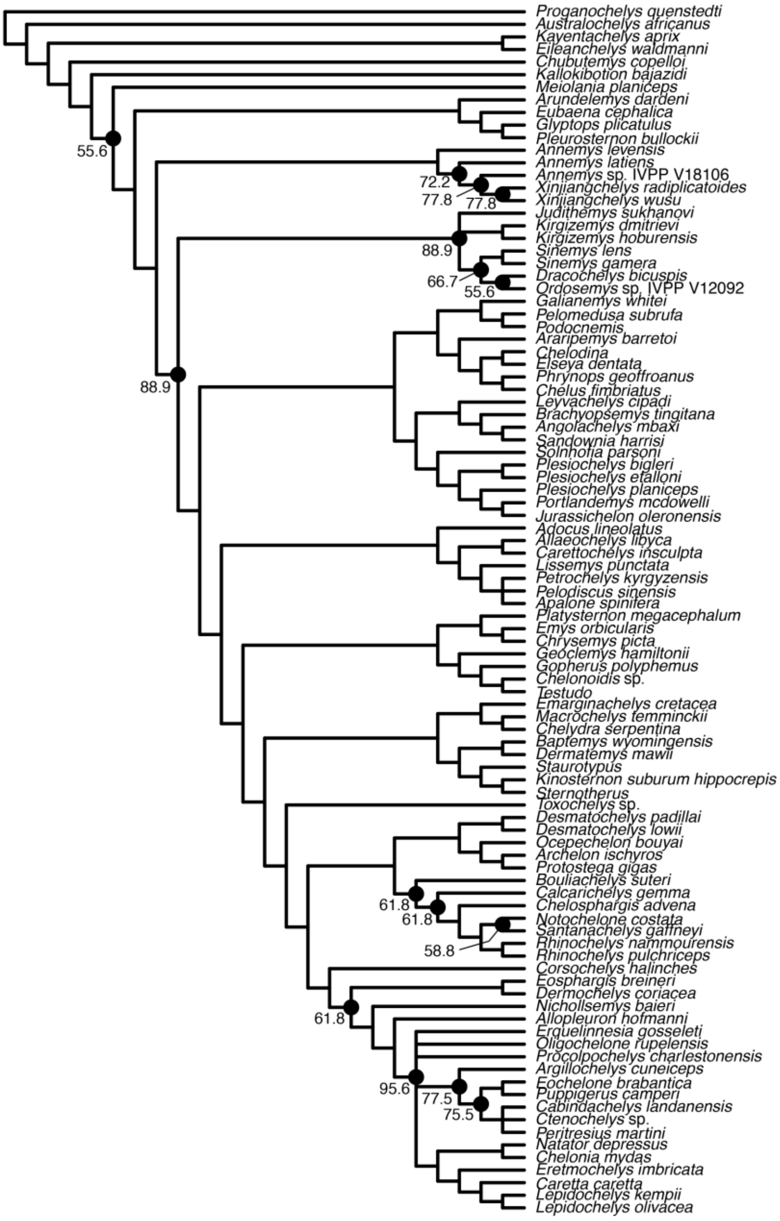

FIG. S1.32. 50%-Majority rule consensus tree. For clarity, only nodes that have been recovered in less than 100% of the MPTs have node labels indicating the proportion (in %) with which the respective nodes have been found. Unresolved nodes are those for which no resolved bifurcation was found in at least 50% of the MPTs.

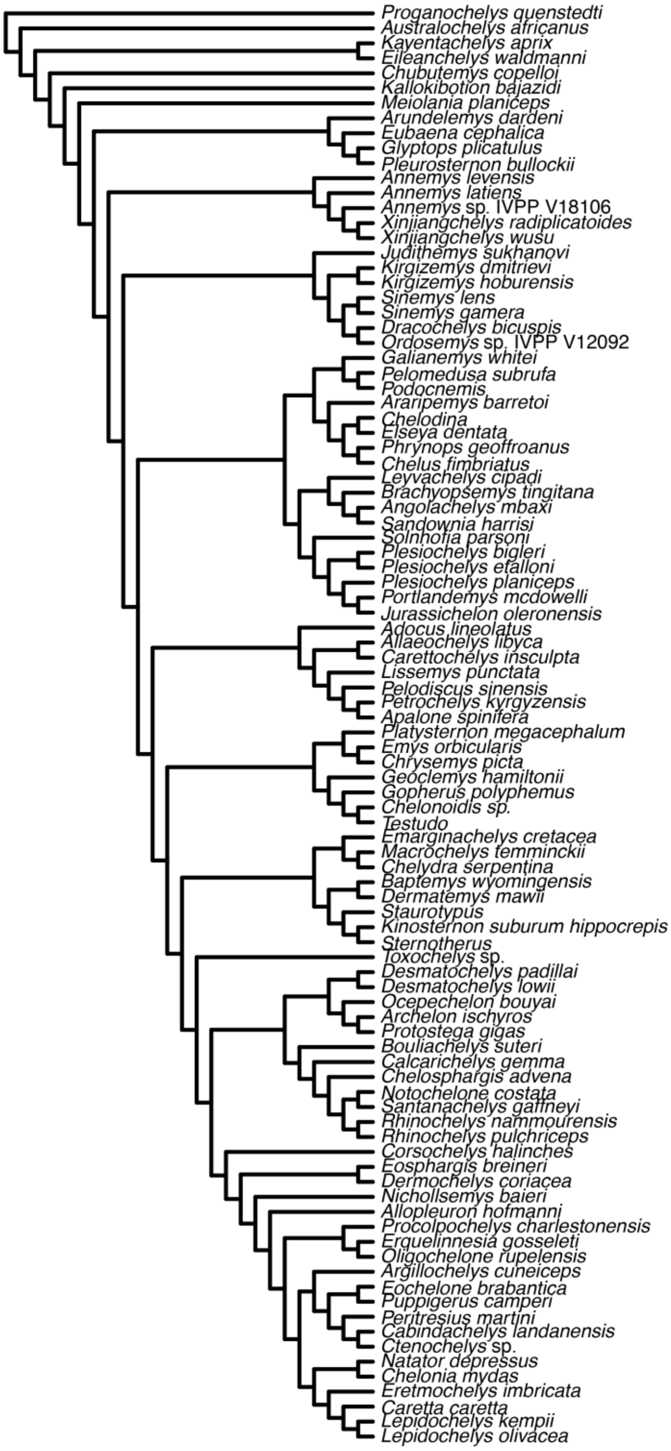

FIG. S1.33. MPT used for optimization of characters.

Results

The optimizations are listed here for all nodes of the MPT (Fig. S1.33). 'Unambiguous' synapomorphies are those found both under ACCTRAN and DELTRAN optimization. For unnamed nodes in our tree, we use a code to describe clades. The notation 'Taxon+++' indicates the clade comprising the named taxon as well as all taxa that a re more crownwardly positioned and member of the clade. '(Taxon A + Taxon B)' denotes the most inclusive clade including taxa A and B.

Australochelys+++:
DELTRAN:
CH64: 0->1, CH87: 0->1, CH91: 0->1, CH143: 0->1, CH145: 0->1;
ACCTRAN:
CH12: 0->1, CH25: 0->1, CH26: 0->1, CH46: 0->1, CH50: 0->1, CH62: 0->1, CH64: 0->1, CH73: 0->1, CH75: 0->1, CH87: 0->1, CH90: 0->1, CH91: 0->1, CH134: 0->1, CH142: 0->1, CH143: 0->1, CH145: 0->1, CH162: 0->1, CH204: 0->1, CH207: 0->1, CH226: 0->1, CH227: 0->1, CH235: 0->1, CH244: 0->1, CH245: 0->1, CH268: 0->1, CH303: 0->1, CH308: 0->1, CH310: 0->1, CH315: 0->1, CH317: 0->1, CH320: 0->1, CH327: 0->1, CH329: 0->1, CH344: 0->1.

(*Kayentachelys* + *Eileanchelys*)+++:
Unambiguous:
CH5: 1->0, CH9: 0->1, CH11: 0->1, CH67: 1->0, CH78: 0->1, CH109: 0->1, CH115: 1->0, CH120: 0->1, CH132: 0->2, CH141: 2->1;
DELTRAN:
CH26: 0->1, CH50: 0->1, CH62: 0->1, CH75: 0->2, CH162: 0->1, CH207: 0->1, CH226: 0->1, CH227: 0->1, CH235: 0->1, CH245: 0->1, CH268: 0->1, CH308: 0->1, CH310: 0->1, CH315: 0->1, CH317: 0->1, CH320: 0->1, CH327: 0->1, CH329: 0->1;
ACCTRAN:
CH21: 0->1, CH75: 1->2, CH77: 0->1, CH167: 0->1.

Chubutemys+++:
Unambiguous:
CH22: 0->1, CH66: 0->2, CH80: 0->1, CH106: 0->1, CH112: 0->1, CH121: 0->1, CH147: 0->1, CH309: 0->1;
DELTRAN:
CH21: 0->1, CH46: 0->1, CH77: 0->2, CH90: 0->1, CH167: 0->1;
ACCTRAN:
CH25: 1->0, CH59: 1->0, CH77: 1->2, CH89: 0->1, CH123: 0->1, CH124: 0->1, CH134: 1->0, CH142: 1->0, CH202: 0->1, CH246: 0->1, CH256: 0->1, CH301: 0->1, CH304: 0->1, CH305: 0->2, CH354: 1->0.

Kallokibotion+++:
Unambiguous:
CH92: 0->1, CH108: 0->1, CH109: 1->2, CH122: 0->2;
DELTRAN:
CH89: 0->1, CH202: 0->1, CH204: 0->1, CH244: 0->1.

Meiolania+++:
Unambiguous:
CH98: 0->1, CH128: 0->1, CH195: 0->1, CH233: 0->1;
DELTRAN:
CH305: 0->2, CH354: 1->0;
ACCTRAN:
CH149: 0->1, CH230: 0->1, CH253: 0->1, CH254: 0->1.

Paracryptodira+++:
Unambiguous:
CH17: 1->0, CH19: 0->2, CH35: 0->1, CH48: 0->1, CH102: 0->1, CH121: 1->0, CH312: 0->2, CH335: 0->1;
DELTRAN:
CH12: 0->1, CH230: 0->1, CH246: 0->1, CH303: 0->1;
ACCTRAN:
CH23: 0->1, CH111: 0->1, CH123: 1->0, CH124: 1->0, CH181: 0->1, CH208: 0->1, CH294: 0->1, CH306: 0->1, CH319: 1->0, CH326: 0->1, CH340: 0->1, CH347: 0->1.

Xinjiangchelyidae+++:
Unambiguous:
CH3: 0->1, CH14: 0->1, CH117: 0->1, CH122: 2->0, CH148: 0->1, CH229: 0->1, CH240: 0->1;
DELTRAN:
CH23: 0->1, CH59: 1->0, CH73: 0->1, CH149: 0->1, CH254: 0->1, CH294: 0->1, CH340: 0->1, CH347: 0->1;
ACCTRAN:
CH4: 0->1, CH39: 0->1, CH41: 0->1, CH177: 0->1, CH180: 0->1, CH253: 1->0, CH282: 0->1, CH283: 0->1.

Sinemydidae/Macroabenidae+++:
Unambiguous:
CH144: 1->0, CH187: 0->1, CH233: 1->0, CH285: 0->1, CH299: 0->2, CH316: 0->1;
DELTRAN:
CH4: 0->1, CH177: 0->1, CH281: 0->1, CH301: 0->1, CH304: 0->1, CH306: 0->1;
ACCTRAN:
CH16: 0->1, CH24: 0->1, CH36: 0->1, CH91: 1->2, CH111: 1->0, CH281: 0->1.

Testudines:
Unambiguous:
CH80: 1->2, CH125: 1->0, CH130: 0->1, CH143: 1->0, CH149: 1->2, CH182: 0->1, CH280: 0->1, CH305: 2->1;
DELTRAN:
CH41: 0->1, CH91: 1->2, CH181: 0->1, CH326: 0->1, CH344: 0->1;
ACCTRAN:
CH1: 0->1, CH29: 0->1, CH39: 1->0, CH101: 0->1, CH129: 1->0, CH208: 1->0, CH210: 0->1, CH330: 0->1.

Cryptodira:
Unambiguous:
CH14: 1->0, CH70: 0->1, CH97: 0->1, CH165: 1->0, CH291: 0->1, CH293: 0->1, CH295: 0->1, CH302: 0->1;
DELTRAN:
CH1: 0->1, CH16: 0->1, CH29: 0->1, CH36: 0->1, CH101: 0->2, CH129: 1->0, CH180: 0->1, CH283: 0->1;
ACCTRAN:
CH35: 1->0, CH76: 0->1, CH101: 1->2, CH178: 0->1, CH193: 0->1, CH213: 0->1, CH258: 0->1, CH297: 0->1.

Durocryptodira:
Unambiguous:
CH253: 0->1, CH266: 0->1;
DELTRAN:
CH76: 0->1, CH282: 0->1;
ACCTRAN:
CH117: 1->0, CH151: 0->1, CH210: 1->0.

Americhelydia:
Unambiguous:
CH66: 2->1, CH68: 0->1, CH248: 0->1;
DELTRAN:
CH35: 1->0, CH330: 0->1;
ACCTRAN:
CH24: 1->0, CH178: 1->0, CH179: 0->1, CH182: 1->0, CH223: 0->1, CH252: 0->1, CH325: 0->1.

total-group Chelonioidea:
Unambiguous:
CH36: 1->0, CH40: 0->1, CH70: 1->0, CH96: 1->0, CH107: 0->1, CH134: 0->2, CH141: 1->2, CH165: 0->1, CH196: 0->1, CH212: 0->1, CH236: 0->1, CH240: 1->0, CH241: 0->1, CH246: 1->2, CH249: 0->1, CH261: 0->1, CH302: 1->0, CH332: 0->2, CH337: 0->1, CH338: 0->1, CH342: 0->1, CH350: 0->1;
DELTRAN:
CH117: 1->0, CH182: 1->0, CH213: 0->1, CH223: 0->1, CH252: 0->1;
ACCTRAN:
CH1: 1->0, CH19: 2->0, CH29: 1->0, CH58: 0->1, CH59: 0->1, CH97: 1->0, CH101: 2->0, CH128: 1->0, CH144: 0->1, CH151: 1->0, CH211: 0->1, CH237: 0->1, CH270: 0->1, CH291: 1->0, CH293: 1->0, CH295: 1->0, CH297: 1->0, CH298: 0->1, CH344: 1->2.

Protostegidae+++:
Unambiguous:
CH142: 0->1, CH163: 0->1, CH224: 0->1, CH260: 0->1, CH313: 0->1, CH314: 0->1, CH328: 1->0, CH334: 0->1, CH341: 0->1, CH345: 0->1, CH352: 0->1, CH354: 0->1;
DELTRAN:
CH19: 2->0, CH128: 1->0, CH144: 0->2, CH237: 0->1, CH291: 1->0, CH295: 1->0, CH325: 0->1, CH344: 1->2;
ACCTRAN:
CH28: 1->0, CH82: 0->1, CH88: 0->1, CH140: 0->2, CH144: 1->2, CH214: 0->1, CH242: 0->1, CH331: 0->1.

Protostegidae:
Unambiguous:
CH63: 0->1, CH76: 1->0, CH93: 0->1, CH101: 0->1, CH105: 0->1, CH118: 0->2, CH222: 1->0, CH249: 1->2, CH339: 0->1;
DELTRAN:
CH1: 1->0, CH28: 1->0, CH59: 0->1, CH82: 0->1, CH140: 0->2, CH214: 0->1, CH242: 0->1, CH293: 1->0;
ACCTRAN:
CH50: 1->0, CH58: 1->0, CH95: 0->1, CH179: 1->0, CH191: 0->1, CH298: 1->0.

(*Bouliachelys* + *Rhinochelys*):
Unambiguous:
CH23: 1->0, CH176: 0->1;
DELTRAN:
CH50: 1->0, CH88: 0->1, CH95: 0->1;
ACCTRAN:
CH17: 0->1, CH226: 1->0.

(*Calcarichelys* + *Rhinochelys*):
Unambiguous:
CH154: 1->0, CH163: 1->0;
DELTRAN:
CH191: 0->1;
ACCTRAN:
CH98: 1->0.

(*Chelosphargis* + *Rhinochelys*):
Unambiguous:
CH4: 1->0;
DELTRAN:

CH17: 0->1, CH226: 1->0.

(*Santanachelys* ++ *Rhinochelys*):
Unambiguous:
CH15: 1->2, CH260: 1->0;
DELTRAN:
CH97: 1->0;
ACCTRAN:
CH187: 1->0.

(*Rhinochelys pulchriceps* + *Rhinochelys nammourensis*):
Unambiguous:
CH10: 0->1;
ACCTRAN:
CH96: 0->1, CH98: 0->1, CH142: 1->0, CH213: 1->0, CH219: 1->0.

Rhinochelys pulchriceps:
DELTRAN:
CH96: 0->1, CH142: 1->0, CH187: 1->0.

Rhinochelys nammourensis:
Unambiguous:
CH17: 1->0;
DELTRAN:
CH213: 1->0, CH219: 1->0.

(*Santanachelys* + *Notochelone*):
DELTRAN:
CH98: 1->0;
ACCTRAN:
CH70: 0->1, CH76: 0->1, CH82: 1->0, CH115: 0->1, CH134: 2->0, CH191: 1->0, CH229: 1->0, CH230: 1->0, CH237: 1->0, CH338: 1->0, CH341: 1->0.

Santanachelys gaffneyi:
Unambiguous:
CH107: 1->0, CH150: 1->0;
DELTRAN:
CH76: 0->1, CH191: 1->0, CH229: 1->0, CH230: 1->0, CH237: 1->0, CH338: 1->0, CH341: 1->0.

Notochelone costata:
Unambiguous:
CH68: 1->0, CH127: 0->1;
DELTRAN:
CH70: 0->1, CH82: 1->0, CH115: 0->1, CH134: 2->0.

Chelosphargis advena:
Unambiguous:
CH144: 2->1.

Calcarichelys gemma:
Unambiguous:
CH1: 0->1, CH106: 1->0, CH247: 0->1;
DELTRAN:
CH98: 1->0.

Bouliachelys suteri:
Unambiguous:
CH9: 1->0, CH12: 1->0, CH77: 2->0, CH84: 0->1, CH124: 0->1, CH135: 0->1;
DELTRAN:
CH179: 0->1;
ACCTRAN:
CH97: 0->1, CH179: 0->1.

(*Desmatochelys* ++ *Protostega*):
Unambiguous:
CH4: 1->0, CH117: 0->1, CH130: 1->0, CH196: 1->0, CH241: 1->2;
DELTRAN:
CH331: 0->1;
ACCTRAN:
CH7: 0->1, CH127: 0->1, CH133: 0->1, CH281: 1->0.

(*Ocepechelon* ++ *Protostega*):
Unambiguous:
CH16: 1->0, CH68: 1->0, CH95: 1->2;
ACCTRAN:
CH3: 1->0, CH88: 1->0, CH106: 1->0, CH188: 0->1, CH215: 0->1, CH219: 1->0, CH247: 0->1, CH263: 0->1, CH332: 2->3, CH336: 0->1.

Ocepechelon bouyai:
Unambiguous:
CH14: 0->1, CH39: 0->1, CH49: 0->1, CH59: 1->0, CH93: 1->0, CH112: 1->0, CH144: 2->1;
DELTRAN:
CH3: 1->0, CH7: 0->1, CH50: 1->0, CH106: 1->0.

(*Protostega* + *Archelon*):
Unambiguous:
CH1: 0->1, CH12: 1->0, CH53: 0->1, CH66: 1->0, CH100: 0->2, CH104: 0->1, CH118: 2->0;
DELTRAN:
CH188: 0->1, CH191: 0->1, CH215: 0->1, CH219: 1->0, CH247: 0->1, CH263: 0->1, CH332: 2->3, CH336: 0->1;
ACCTRAN:
CH7: 1->0, CH41: 1->0, CH76: 0->1, CH81: 0->1, CH150: 1->0, CH154: 1->0.

Protostega gigas:
Unambiguous:
CH187: 1->0;
DELTRAN:
CH81: 0->1, CH150: 1->0, CH154: 1->0, CH319: 1->0;
ACCTRAN:
CH281: 0->1.

Archelon ischyros:
Unambiguous:
CH45: 0->1;
DELTRAN:
CH41: 1->0, CH76: 0->1, CH281: 1->0;
ACCTRAN:
CH319: 0->1.

(*Desmatochelys lowii* + *Desmatochelys padillai*):

Unambiguous:
CH20: 0->1, CH77: 2->1, CH224: 1->0;
DELTRAN:
CH7: 0->1, CH281: 1->0;
ACCTRAN:
CH50: 0->1, CH191: 1->0, CH210: 0->1.

Desmatochelys lowii:
Unambiguous:
CH41: 1->0, CH200: 0->1;
DELTRAN:
CH88: 0->1, CH127: 0->1, CH133: 0->1, CH210: 0->1, CH211: 0->1;
ACCTRAN:
CH95: 1->0, CH97: 0->1.

Desmatochelys padillai:
Unambiguous:
CH19: 0->1, CH101: 1->2, CH154: 1->0;
DELTRAN:
CH95: 0->1, CH97: 1->0.

Corsochelys+++:
Unambiguous:
CH16: 1->0, CH41: 1->0, CH106: 1->0, CH115: 0->1, CH216: 0->1;
ACCTRAN:
CH1: 0->1, CH59: 1->0, CH121: 0->1, CH188: 0->1, CH262: 0->1, CH263: 0->1, CH292: 0->1, CH293: 0->1, CH294: 1->0, CH330: 1->0, CH347: 1->0, CH348: 0->1.

crown-group Chelonioidea:
Unambiguous:
CH23: 1->0, CH146: 0->1;
DELTRAN:
CH58: 0->1, CH144: 2->1, CH214: 0->1, CH262: 0->1, CH292: 0->1, CH298: 0->1, CH330: 1->0, CH331: 0->1, CH347: 1->0, CH348: 0->1;
ACCTRAN:
CH127: 0->1, CH137: 0->1, CH144: 2->1, CH215: 0->1, CH217: 0->2.

Dermochelyidae:
Unambiguous:
CH12: 1->0, CH17: 0->1, CH54: 0->1, CH95: 0->2, CH98: 1->0, CH100: 0->2, CH112: 1->0, CH151: 0->1, CH332: 2->3, CH333: 0->1;
DELTRAN:
CH28: 1->0, CH188: 0->1, CH242: 0->1, CH263: 0->1, CH294: 1->0, CH319: 1->0;
ACCTRAN:
CH6: 0->1, CH15: 1->0, CH22: 1->0, CH72: 0->1, CH80: 2->1, CH82: 1->0, CH122: 0->1, CH126: 0->1, CH140: 2->0, CH185: 0->2, CH202: 1->0, CH243: 0->1, CH338: 1->0.

Dermochelys coriacea:
Unambiguous:
CH26: 1->0, CH43: 0->1, CH68: 1->0, CH96: 0->1, CH203: 0->1, CH206: 0->1, CH241: 1->0, CH353: 0->1;
DELTRAN:
CH6: 0->1, CH15: 1->0, CH22: 1->0, CH72: 0->1, CH80: 2->1, CH88: 0->1, CH122: 0->3, CH126: 0->1, CH179: 0->1, CH185: 0->2, CH243: 0->1, CH338: 1->0;
ACCTRAN:

CH121: 1->0, CH122: 1->3, CH137: 1->0.

Eosphargis breineri:
Unambiguous:
CH92: 1->0, CH103: 0->1, CH138: 0->1, CH191: 0->1;
DELTRAN:
CH59: 0->1, CH121: 0->1, CH122: 0->1, CH137: 0->1, CH202: 1->0, CH215: 0->1;
ACCTRAN:
CH59: 0->1.

total-group Cheloniidae:
Unambiguous:
CH66: 1->0, CH104: 0->1, CH111: 0->1, CH163: 1->0;
DELTRAN:
CH82: 0->1, CH121: 0->1, CH127: 0->1, CH137: 0->1, CH140: 0->2;
ACCTRAN:
CH28: 0->1, CH130: 1->0, CH178: 0->1, CH242: 1->0, CH294: 0->1, CH319: 0->1, CH344: 2->1, CH346: 0->1.

Allopleuron+++:
Unambiguous:
CH56: 0->1, CH57: 0->1, CH68: 1->2, CH69: 0->1, CH103: 0->1;
DELTRAN:
CH344: 2->1, CH346: 0->1;
ACCTRAN:
CH29: 0->1, CH88: 1->0, CH97: 0->1, CH101: 0->2.

(*Erquelinnesia* ++ *Lepidochelys*):
Unambiguous:
CH17: 0->1, CH45: 0->1, CH50: 1->0, CH95: 0->2, CH224: 1->0;
DELTRAN:
CH178: 0->1, CH217: 0->2;
ACCTRAN:
CH130: 0->1, CH188: 1->0, CH215: 1->0, CH263: 1->0, CH326: 1->0.

(*Puppigerus* ++ *Lepidochelys*):
Unambiguous:
CH330: 0->1;
DELTRAN:
CH319: 1->0;
ACCTRAN:
CH176: 0->1, CH319: 1->0.

crown-group Cheloniidae:
Unambiguous:
CH6: 0->1, CH152: 0->1, CH217: 2->1, CH302: 0->1, CH336: 0->1, CH353: 0->1;
DELTRAN:
CH88: 0->1, CH179: 0->1;
ACCTRAN:
CH59: 0->2, CH70: 0->1, CH72: 0->1, CH88: 0->1, CH326: 0->1.

(*Eretmochelys* ++ *Lepidochelys*):
Unambiguous:
CH8: 0->1, CH30: 1->0, CH115: 1->0;
DELTRAN:

CH72: 0->1;
ACCTRAN:
CH176: 1->0.

(*Caretta* ++ *Lepidochelys*):
Unambiguous:
CH234: 0->1, CH313: 1->0;
ACCTRAN:
CH12: 1->0, CH59: 2->0, CH128: 0->1, CH144: 1->2, CH184: 0->1, CH191: 0->1, CH207: 1->0, CH270: 1->0.

(*Lepidochelys olivacea* + *Lepidochelys kempii*):
Unambiguous:
CH146: 1->0, CH204: 1->0;
DELTRAN:
CH70: 0->1, CH176: 0->1;
ACCTRAN:
CH176: 0->1.

Lepidochelys olivacea:
Unambiguous:
CH102: 1->0, CH134: 2->1, CH175: 0->1;
DELTRAN:
CH12: 1->0, CH144: 1->2, CH184: 0->1, CH191: 0->1;
ACCTRAN:
CH128: 1->0.

Lepidochelys kempii:
Unambiguous:
CH30: 0->1, CH53: 0->1, CH104: 1->0, CH179: 1->0;
DELTRAN:
CH59: 0->2, CH128: 0->1;
ACCTRAN:
CH12: 0->1, CH59: 0->2, CH144: 2->1, CH184: 1->0, CH191: 1->0.

Caretta caretta:
Unambiguous:
CH61: 0->1, CH65: 0->1, CH100: 0->1, CH163: 0->1, CH223: 1->0;
DELTRAN:
CH12: 1->0, CH128: 0->1, CH144: 1->2, CH184: 0->1, CH191: 0->1, CH207: 1->0;
ACCTRAN:
CH70: 1->0.

Eretmochelys imbricata:
Unambiguous:
CH118: 0->1, CH122: 0->1, CH134: 2->0, CH177: 1->0, CH226: 1->0;
DELTRAN:
CH59: 0->2, CH70: 0->1, CH270: 0->1.

(*Chelonia* + *Natator*):
Unambiguous:
CH80: 2->1, CH180: 1->0;
DELTRAN:
CH59: 0->2, CH176: 0->1, CH270: 0->1.

Chelonia mydas:
Unambiguous:
CH100: 0->2, CH102: 1->0, CH103: 1->0, CH104: 1->0, CH117: 0->1, CH122: 0->1, CH126: 0->1, CH128: 0->1, CH134: 2->0, CH141: 2->0, CH191: 0->1, CH217: 1->0;
ACCTRAN:
CH70: 1->0, CH72: 1->0.

Natator depressus:
Unambiguous:
CH76: 1->0, CH144: 1->2, CH177: 1->0, CH184: 0->1, CH226: 1->0, CH355: 0->1;
DELTRAN:
CH70: 0->1, CH72: 0->1.

(*Argillochelys* ++ *Puppigerus*):
Unambiguous:
CH117: 0->1;
DELTRAN:
CH176: 0->1;
ACCTRAN:
CH179: 1->0, CH262: 1->0, CH328: 0->1, CH355: 0->1.

(*Puppigerus* ++ *Ctenochelys*):
Unambiguous:
CH25: 0->1;
DELTRAN:
CH262: 1->0, CH328: 0->1, CH355: 0->1;
ACCTRAN:
CH104: 1->0, CH142: 1->0.

(*Puppigerus* + *Eochelone*):
Unambiguous:
CH115: 1->0, CH237: 1->0, CH241: 1->0;
DELTRAN:
CH142: 1->0;
ACCTRAN:
CH122: 0->1, CH294: 1->0.

Puppigerus camperi:
Unambiguous:
CH92: 1->0, CH102: 1->0, CH146: 1->0, CH151: 0->1, CH175: 0->1, CH212: 1->0, CH223: 1->0, CH260: 1->0;
DELTRAN:
CH88: 0->1, CH122: 0->1, CH294: 1->0, CH326: 1->0;
ACCTRAN:
CH88: 0->1, CH104: 0->1.

Eochelone brabantica:
Unambiguous:
CH56: 1->0, CH57: 1->0, CH58: 1->0, CH66: 0->1, CH68: 2->1, CH134: 2->0, CH140: 2->1, CH176: 1->0, CH330: 1->0;
DELTRAN:
CH104: 1->0, CH122: 0->2;
ACCTRAN:
CH122: 1->2.

(*Ctenochelys* ++ *Peritresius*):
Unambiguous:
CH190: 0->1, CH191: 0->1, CH195: 1->0, CH200: 0->1, CH226: 1->0;
ACCTRAN:
CH14: 0->1, CH16: 0->1, CH45: 1->0, CH95: 2->0, CH101: 2->0, CH103: 1->0, CH127: 1->0, CH179: 0->1, CH286: 1->0, CH316: 1->0, CH332: 2->0.

Peritresius martini:
Unambiguous:
CH230: 1->0, CH279: 0->1, CH297: 0->1, CH319: 0->1.

(*Ctenochelys* + *Cabindachelys*):
DELTRAN:
CH14: 0->1, CH45: 1->0, CH95: 2->0, CH101: 2->0, CH103: 1->0, CH104: 1->0, CH127: 1->0;
ACCTRAN:
CH217: 2->0.

Ctenochelys sp.:
Unambiguous:
CH6: 0->1, CH17: 1->0, CH59: 0->1, CH66: 0->1, CH67: 0->1, CH68: 2->1, CH106: 0->1, CH140: 2->1, CH163: 0->1, CH164: 0->1;
DELTRAN:
CH16: 0->1, CH179: 0->1, CH217: 2->0, CH286: 1->0, CH316: 1->0, CH332: 2->0;
ACCTRAN:
CH142: 0->1.

Cabindachelys landanensis:
Unambiguous:
CH22: 1->2;
DELTRAN:
CH88: 0->1, CH97: 1->0, CH142: 1->0;
ACCTRAN:
CH88: 0->1, CH97: 1->0.

Argillochelys cuneiceps:
Unambiguous:
CH59: 0->1, CH151: 0->1, CH156: 1->0.

(*Procolpochelys* ++ *Erquelinnesia*):
Unambiguous:
CH175: 0->1;
ACCTRAN:
CH30: 1->0, CH100: 0->1, CH102: 1->0.

Procolpochelys charlestonensis:
Unambiguous:
CH204: 1->0, CH213: 1->0, CH222: 1->0, CHs 199: 0->1.
DELTRAN:
CH30: 1->0, CH100: 0->1, CH102: 1->0, CH179: 0->1.

(*Oligochelone* + *Erquelinnesia*):
DELTRAN:
CH326: 1->0;
ACCTRAN:
CH14: 0->1, CH49: 0->1, CH179: 1->0.

Erquelinnesia gosseleti:
Unambiguous:
CH214: 1->0, CH230: 1->0, CH334: 1->0;
DELTRAN:
CH14: 0->1, CH49: 0->1.

Allopleuron hofmanni:
Unambiguous:
CH26: 1->0, CH43: 0->1, CH77: 2->1, CH80: 2->1, CH81: 0->1, CH85: 0->1, CH133: 0->1, CH165: 1->0, CH177: 1->0, CH187: 1->0, CH191: 0->1, CH196: 1->0, CH226: 1->0;
DELTRAN:
CH130: 1->0, CH188: 0->1, CH215: 0->1, CH263: 0->1.

Nichollsemys baieri:
Unambiguous:
CH19: 0->1, CH35: 0->1, CH63: 0->1, CH90: 1->0, CH105: 0->1, CH125: 0->1, CH128: 0->1, CH152: 0->1;
DELTRAN:
CH29: 1->0, CH88: 0->1, CH97: 1->0, CH101: 2->0, CH130: 1->0.

Corsochelys halinches:
Unambiguous:
CH14: 0->1, CH119: 0->1, CH129: 0->1, CH140: 2->1, CH157: 0->1, CH285: 1->0, CH332: 2->1;
DELTRAN:
CH294: 1->0;
ACCTRAN:
CH88: 1->0, CH214: 1->0, CH331: 1->0.

Toxochelys sp.:
Unambiguous:
CH14: 0->1, CH39: 0->1, CH66: 1->0, CH137: 0->1, CH146: 0->1, CH174: 0->1, CH184: 0->1, CH200: 0->1, CH275: 0->1, CH277: 0->1, CH278: 0->1, CH306: 1->0;
DELTRAN:
CH1: 1->0, CH19: 2->1, CH29: 1->0, CH58: 0->1, CH59: 0->1, CH97: 1->0, CH101: 2->0, CH144: 0->1, CH179: 0->1, CH293: 1->0, CH298: 0->1, CH319: 1->0;
ACCTRAN:
CH19: 0->1.

Chelydroidea:
Unambiguous:
CH156: 1->0, CH181: 1->0, CH197: 0->1, CH222: 1->0;
DELTRAN:
CH151: 0->1, CH297: 0->1;
ACCTRAN:
CH60: 0->1, CH117: 0->1, CH190: 0->1, CH191: 0->1, CH213: 1->0, CH239: 0->1, CH272: 0->1, CH326: 1->0.

total-group Chelydridae:
Unambiguous:
CH82: 0->1, CH249: 0->2, CH276: 0->1;
ACCTRAN:
CH41: 1->0, CH127: 0->1, CH176: 0->1, CH202: 1->0, CH226: 1->0, CH305: 1->2, CH306: 1->0, CH325: 1->0, CH349: 0->1.

crown-group Chelydridae:
Unambiguous:
CH12: 1->0, CH36: 1->0, CH53: 0->1, CH68: 1->0, CH78: 1->2, CH96: 1->0, CH101: 2->0, CH115: 0->1, CH212: 0->1, CH236: 0->1, CH246: 1->2, CH260: 0->1, CH261: 0->1, CH277: 0->1;
DELTRAN:
CH127: 0->1, CH176: 0->1, CH179: 0->1, CH182: 1->0, CH202: 1->0, CH226: 1->0, CH252: 0->1, CH305: 1->2, CH306: 1->0, CH319: 1->0;
ACCTRAN:
CH190: 1->0, CH191: 1->0.

Chelydra serpentina:
Unambiguous:
CH9: 1->0, CH25: 0->1, CH29: 1->0, CH59: 0->1, CH125: 0->1, CH143: 0->1, CH198: 1->2, CH222: 0->2;
DELTRAN:
CH272: 0->1;
ACCTRAN:
CH41: 0->1, CH326: 0->1.

Macrochelys temminckii:
Unambiguous:
CH66: 1->0, CH85: 0->1, CH118: 0->1, CH141: 1->0, CH144: 0->1, CH151: 1->0, CH156: 0->1, CH227: 1->0, CH304: 1->2;
DELTRAN:
CH41: 1->0, CH258: 0->1, CH326: 1->0, CH349: 0->1.

Emarginachelys cretacea:
Unambiguous:
CH30: 1->0, CH59: 0->2, CH109: 2->1, CH111: 0->1, CH324: 0->1, CH345: 0->1;
DELTRAN:
CH41: 1->0, CH60: 0->1, CH190: 0->1, CH191: 0->1.

Kinosternoidea:
Unambiguous:
CH22: 1->2, CH23: 1->0, CH55: 0->1, CH118: 0->2, CH125: 0->1, CH208: 0->1, CH235: 1->0, CH273: 0->1;
DELTRAN:
CH325: 0->1, CH326: 1->0;
ACCTRAN:
CH122: 0->2, CH124: 0->1, CH159: 0->1, CH179: 1->0, CH182: 0->1, CH185: 0->1, CH210: 0->1, CH252: 1->0, CH288: 2->1, CH319: 0->1, CH323: 0->1, CH328: 1->0.

Kinosternidae:
Unambiguous:
CH12: 1->0, CH18: 0->1, CH27: 0->1, CH33: 0->1, CH133: 0->1, CH207: 1->2, CH238: 0->1, CH253: 1->2, CH275: 0->1, CH279: 0->1, CH300: 0->1, CH324: 0->1;
DELTRAN:
CH159: 0->1, CH185: 0->1, CH190: 0->1, CH191: 0->1, CH193: 0->1, CH239: 0->1, CH258: 0->1, CH288: 2->1, CH323: 0->1, CH328: 1->0;
ACCTRAN:
CH43: 0->1, CH68: 1->2, CH69: 0->1, CH78: 1->2, CH102: 1->0, CH345: 0->1.

Kinosterninae:
Unambiguous:
CH25: 0->1, CH186: 0->1, CH209: 0->1, CH243: 0->1, CH270: 0->1, CH271: 0->1;

DELTRAN:
CH210: 0->1;
ACCTRAN:
CH124: 1->0.

Sternotherus:
Unambiguous:
CH98: 1->0, CH100: 0->1, CH117: 1->0, CH118: 2->0, CH130: 1->0, CH139: 0->1, CH177: 1->0, CH211: 0->1;
DELTRAN:
CH68: 1->2, CH69: 0->1, CH78: 1->2, CH122: 0->2, CH345: 0->1;
ACCTRAN:
CH43: 1->0, CH102: 0->1.

Kinosternon suburum hippocrepis:
Unambiguous:
CH112: 1->0, CH116: 0->1, CH127: 0->1, CH143: 0->1, CH161: 1->0, CH183: 0->1, CH208: 1->0, CH326: 0->1, CHis 53: 0->1.
DELTRAN:
CH43: 0->1, CH102: 1->0, CH182: 1->0.
ACCTRAN:
CH68: 2->1, CH69: 1->0, CH78: 2->1, CH122: 2->0, CH182: 1->0.

Staurotypus:
Unambiguous:
CH39: 0->1, CH50: 1->0, CH82: 0->1, CH107: 0->1, CH141: 1->0;
DELTRAN:
CH43: 0->1, CH68: 1->2, CH69: 0->1, CH78: 1->2, CH102: 1->0, CH122: 0->2, CH124: 0->1, CH319: 1->0;
ACCTRAN:
CH319: 1->0.

total-group Dermatemydidae:
Unambiguous:
CH35: 0->1, CH59: 0->2, CH80: 2->1, CH98: 1->0, CH158: 0->1, CH177: 1->0, CH178: 0->1, CH248: 1->0, CH251: 0->1;
ACCTRAN:
CH128: 1->0, CH258: 1->0.

Dermatemys mawii:
Unambiguous:
CH65: 0->1, CH106: 1->0, CH117: 1->0, CH141: 1->0, CH259: 0->1, CH270: 0->1, CH287: 1->0;
DELTRAN:
CH124: 0->1, CH128: 1->0, CH185: 0->1, CH210: 0->1;
ACCTRAN:
CH60: 1->0, CH122: 2->0, CH190: 1->0, CH191: 1->0, CH272: 1->0, CH323: 1->0.

Baptemys wyomingensis:
Unambiguous:
CH41: 1->0, CH121: 0->1, CH144: 0->1, CH151: 1->0, CH156: 0->1, CH174: 0->1, CH186: 0->1, CH257: 0->1;
DELTRAN:
CH60: 0->1, CH122: 0->2, CH182: 1->0, CH190: 0->1, CH191: 0->1, CH272: 0->1, CH323: 0->1;
ACCTRAN:
CH182: 1->0, CH185: 1->0, CH210: 1->0.

Testudinoidea:
Unambiguous:
CH98: 1->0, CH125: 0->1, CH177: 1->0, CH259: 0->1, CH289: 0->1;
DELTRAN:
CH151: 0->1;
ACCTRAN:
CH12: 1->0, CH80: 2->1, CH118: 0->2, CH199: 0->1, CH279: 0->1, CH319: 0->1.

Emysternia:
Unambiguous:
CH226: 1->0, CH345: 0->1;
DELTRAN:
CH118: 0->2.

Emydidae:
Unambiguous:
CH106: 1->0, CH123: 0->1, CH156: 1->0, CH222: 1->0;
DELTRAN:
CH199: 0->1, CH297: 0->1, CH330: 0->1;
ACCTRAN:
CH35: 0->1, CH347: 1->0.

Chrysemys picta:
Unambiguous:
CH23: 1->0, CH39: 0->1, CH59: 0->2, CH69: 0->1, CH115: 0->1, CH122: 0->1, CH124: 0->1, CH128: 1->0, CH235: 1->0, CH251: 0->1, CH257: 0->1, CH274: 0->1, CH290: 0->1;
DELTRAN:
CH117: 1->0, CH178: 0->1, CH279: 0->1;
ACCTRAN:
CH12: 0->1, CH80: 1->2, CH258: 1->0.

Emys orbicularis:
Unambiguous:
CH29: 1->0, CH66: 2->0, CH73: 1->0, CH92: 1->0, CH143: 0->1, CH146: 0->1, CH238: 0->1, CH259: 1->0, CH272: 0->1;
DELTRAN:
CH12: 1->0, CH24: 0->1, CH80: 2->1, CH258: 0->1, CH347: 1->0;
ACCTRAN:
CH117: 0->1.

Platysternon megacephalum:
Unambiguous:
CH17: 0->1, CH19: 2->0, CH33: 0->1, CH36: 1->0, CH41: 1->0, CH44: 0->1, CH53: 0->1, CH59: 0->1, CH66: 2->1, CH76: 1->0, CH78: 1->2, CH96: 1->0, CH102: 1->0, CH103: 0->1, CH111: 0->1, CH126: 0->1, CH127: 0->1, CH140: 0->2, CH141: 1->0, CH144: 0->1, CH161: 1->0, CH165: 0->1, CH182: 1->0, CH183: 0->1, CH186: 0->1, CH202: 1->0, CH208: 0->1, CH252: 0->1, CH258: 1->2, CH282: 1->0, CH305: 1->2, CH306: 1->0;
DELTRAN:
CH12: 1->0, CH35: 1->0, CH80: 2->1, CH117: 1->0, CH319: 1->0;
ACCTRAN:
CH24: 1->0, CH178: 1->0, CH199: 1->0, CH279: 1->0, CH297: 1->0, CH319: 1->0, CH330: 1->0.

Testuguria:
Unambiguous:

CH67: 0->1, CH73: 1->0, CH157: 0->1, CH181: 1->0, CH235: 1->0, CH250: 1->0, CH251: 0->1, CH274: 0->1;
DELTRAN:
CH24: 0->1, CH199: 0->1, CH279: 0->1, CH297: 0->1;
ACCTRAN:
CH18: 0->1, CH180: 1->0, CH257: 0->1, CH354: 0->1.

Geoclemys hamiltonii:
Unambiguous:
CH27: 0->1, CH43: 0->1, CH68: 0->1, CH69: 0->1, CH122: 0->3, CH123: 0->1, CH190: 0->1, CH191: 0->1, CH208: 0->1, CH272: 0->1, CH334: 0->1;
DELTRAN:
CH12: 1->0, CH18: 0->1, CH35: 1->0, CH117: 1->0, CH118: 0->2, CH178: 0->1, CH257: 0->1, CH258: 0->1, CH330: 0->1, CH354: 0->1;
ACCTRAN:
CH80: 1->2.

Testudinidae:
Unambiguous:
CH28: 1->0, CH59: 0->2, CH77: 2->1, CH78: 1->2, CH143: 0->1, CH156: 1->0, CH202: 1->0, CH253: 1->0;
DELTRAN:
CH80: 2->1, CH180: 1->0;
ACCTRAN:
CH12: 0->1, CH35: 0->1, CH97: 1->0, CH106: 1->0, CH107: 0->1, CH117: 0->1, CH118: 2->0, CH133: 0->2, CH218: 0->1, CH258: 1->0, CH330: 1->0, CH340: 1->0, CH349: 0->1.

(*Testudo* + *Chelonoidis*):
Unambiguous:
CH76: 1->0, CH182: 1->0;
DELTRAN:
CH18: 0->1;
ACCTRAN:
CH178: 1->0, CH257: 1->0.

Testudo:
Unambiguous:
CH43: 0->1, CH67: 1->0, CH102: 1->0, CH115: 0->1, CH123: 0->1, CH185: 0->1;
DELTRAN:
CH106: 1->0, CH218: 0->1, CH330: 0->1, CH340: 1->0, CH349: 0->1;
ACCTRAN:
CH97: 0->1, CH133: 2->0, CH330: 0->1.

Chelonoidis sp.:
Unambiguous:
CH14: 0->1, CH41: 1->0, CH163: 0->1, CH199: 1->0, CH208: 0->1, CH226: 1->0, CH235: 0->1, CH250: 0->1, CH251: 1->0, CH252: 0->1, CH258: 0->2, CH274: 1->0, CH279: 1->0, CH282: 1->0, CH297: 1->0, CH305: 1->2, CH306: 1->0, CH319: 1->0;
DELTRAN:
CH97: 1->0, CH107: 0->1, CH133: 0->2;
ACCTRAN:
CH106: 0->1, CH218: 1->0, CH340: 0->1, CH349: 1->0.

Gopherus polyphemus:
Unambiguous:

CH22: 1->2, CH23: 1->0, CH55: 0->1, CH72: 0->1, CH104: 0->1, CH126: 0->1, CH128: 1->0, CH141: 1->0;
DELTRAN:
CH97: 1->0, CH106: 1->0, CH133: 0->2, CH178: 0->1, CH218: 0->1, CH257: 0->1, CH340: 1->0, CH349: 0->1;
ACCTRAN:
CH18: 1->0.

total-group Trionychia:
Unambiguous:
CH18: 0->1, CH22: 1->2, CH23: 1->0, CH55: 0->1, CH118: 0->1, CH186: 0->1, CH287: 1->0, CH324: 0->1;
DELTRAN:
CH39: 0->1;
ACCTRAN:
CH25: 0->1, CH39: 0->1, CH60: 0->1, CH121: 0->1, CH127: 0->1, CH193: 1->2, CH222: 1->0, CH233: 0->1, CH264: 0->1, CH272: 0->1, CH274: 0->1, CH278: 0->1, CH330: 1->0.

crown-group Trionychia:
Unambiguous:
CH6: 0->1, CH49: 0->1, CH50: 1->0, CH63: 0->1, CH65: 0->1, CH72: 0->1, CH78: 1->2, CH96: 1->2, CH100: 0->1, CH105: 0->1, CH114: 0->1, CH115: 0->1, CH134: 0->1, CH192: 0->1, CH224: 0->1, CH263: 0->1, CH325: 0->1, CH328: 1->0, CH334: 0->1, CH345: 0->1;
DELTRAN:
CH25: 0->1, CH35: 1->0, CH76: 0->1, CH127: 0->1, CH193: 0->2, CH210: 0->1, CH319: 1->0;
ACCTRAN:
CH183: 0->1, CH189: 0->1, CH207: 1->0, CH253: 0->2, CH282: 1->0, CH297: 1->0, CH343: 0->1.

Trionychidae:
Unambiguous:
CH14: 0->1, CH27: 0->1, CH31: 0->1, CH41: 1->0, CH43: 0->1, CH51: 0->1, CH52: 0->1, CH61: 0->1, CH81: 0->1, CH106: 1->0, CH150: 1->0, CH188: 0->1, CH206: 0->1, CH221: 0->1, CH236: 0->1, CH237: 0->1, CH246: 1->3, CH248: 0->1, CH249: 0->3, CH252: 0->1, CH286: 1->2, CH331: 0->1, CH351: 0->1;
DELTRAN:
CH330: 0->1, CH343: 0->1;
ACCTRAN:
CH121: 1->0, CH178: 1->0, CH180: 1->0, CH330: 0->1.

Trionychinae:
Unambiguous:
CH212: 0->1;
DELTRAN:
CH180: 1->0, CH183: 0->1, CH213: 0->1;
ACCTRAN:
CH165: 0->1, CH324: 1->0.

(*Apalone* + *Petrochelys*):
DELTRAN:
CH165: 0->1;
ACCTRAN:
CH59: 0->1, CH97: 1->0, CH347: 1->0.

Apalone spinifera:
DELTRAN:

CH59: 0->1, CH97: 1->0, CH324: 1->0, CH347: 1->0.

Pelodiscus sinensis:
Unambiguous:
CH124: 0->1.

Lissemys punctata:
Unambiguous:
CH30: 1->0, CH70: 1->0, CH102: 1->0, CH105: 1->2, CH166: 1->0, CH186: 1->0;
ACCTRAN:
CH183: 1->0.

total-group Carettochelyidae:
Unambiguous:
CH86: 0->1, CH118: 1->2, CH122: 0->3, CH126: 0->1, CH139: 0->1;
DELTRAN:
CH121: 0->1;
ACCTRAN:
CH33: 0->1, CH59: 0->1, CH66: 2->0, CH67: 0->1, CH77: 2->0, CH177: 1->0, CH195: 1->0, CH343: 1->2, CH344: 1->2, CH346: 0->1, CH347: 1->0.

Carettochelys insculpta:
DELTRAN:
CH33: 0->1, CH59: 0->1, CH66: 2->0, CH67: 0->1, CH77: 2->0, CH177: 1->0, CH178: 0->1, CH183: 0->1, CH189: 0->1, CH195: 1->0, CH207: 1->0, CH222: 1->0, CH253: 0->2, CH258: 0->1, CH343: 0->2, CH344: 1->2, CH346: 0->1, CH347: 1->0.

Allaeochelys libyca:
Unambiguous:
CH111: 0->1, CH128: 1->0;
DELTRAN:
CH77: 2->1;
ACCTRAN:
CH77: 0->1.

Adocus lineolatus:
Unambiguous:
CH59: 0->2, CH82: 0->1, CH111: 0->1, CH144: 0->1, CH149: 2->1, CH199: 0->1, CH201: 0->1, CH235: 1->0, CH286: 1->0;
DELTRAN:
CH60: 0->1, CH121: 0->1, CH178: 0->1, CH233: 0->1, CH264: 0->1, CH272: 0->1, CH274: 0->1, CH278: 0->1, CH282: 0->1, CH297: 0->1;
ACCTRAN:
CH35: 0->1, CH76: 1->0, CH258: 1->0, CH319: 0->1.

total-group Pleurodira:
Unambiguous:
CH63: 0->1, CH66: 2->0, CH235: 1->0, CH251: 0->1, CH257: 0->1, CH265: 0->1, CH311: 0->1;
DELTRAN:
CH330: 0->1;
ACCTRAN:
CH98: 1->0, CH100: 0->1, CH105: 0->1, CH122: 0->1, CH124: 0->1, CH134: 0->1, CH282: 1->0, CH283: 1->0, CH288: 2->0, CH334: 0->1.

Angolachelonia:

Unambiguous:
CH19: 2->0, CH84: 0->1, CH85: 0->1, CH96: 1->0, CH117: 1->0, CH183: 0->1, CH184: 0->1, CH186: 0->1;
DELTRAN:
CH24: 0->1, CH105: 0->1;
ACCTRAN:
CH1: 1->0, CH16: 1->0, CH29: 1->0, CH36: 1->0, CH57: 0->1, CH68: 0->2, CH144: 0->1, CH175: 0->1, CH236: 0->1, CH354: 0->1.

Thalassochelydia:
Unambiguous:
CH41: 1->0, CH82: 0->1, CH115: 0->1, CH187: 1->0, CH216: 0->1, CH229: 1->0, CH285: 1->0, CH294: 1->0;
DELTRAN:
CH98: 1->0, CH144: 0->1, CH236: 0->1, CH334: 0->1, CH354: 0->1;
ACCTRAN:
CH109: 2->1, CH118: 0->2, CH124: 1->0, CH129: 0->1, CH134: 1->0, CH299: 2->1.

Solnhofia parsoni:
Unambiguous:
CH18: 0->1, CH50: 1->0, CH61: 0->1, CH65: 0->1, CH97: 0->1, CH102: 1->0, CH121: 0->1, CH128: 1->0, CH176: 0->1, CH212: 0->1, CH222: 1->2, CH235: 0->1;
DELTRAN:
CH57: 0->1, CH68: 0->2, CH100: 0->1, CH109: 2->1, CH118: 0->2, CH122: 0->1, CH175: 0->1, CH180: 0->1;
ACCTRAN:
CH210: 1->0.

Plesiochelyidae:
Unambiguous:
CH28: 1->0, CH40: 0->1, CH59: 0->1, CH95: 0->1, CH141: 1->2, CH154: 1->0, CH157: 0->1, CH177: 1->0, CH182: 1->0;
DELTRAN:
CH101: 0->1, CH134: 0->2;
ACCTRAN:
CH57: 1->0, CH68: 2->0, CH100: 1->0, CH122: 1->0, CH134: 0->2, CH175: 1->0, CH225: 1->0, CH325: 0->1.

(*Plesiochelys planiceps* ++ *Jurassichelon*):
Unambiguous:
CH4: 1->0, CH111: 0->1, CH117: 0->1;
ACCTRAN:
CH76: 0->1.

(*Jurassichelon* + *Portlandemys*):
Unambiguous:
CH7: 0->1, CH91: 2->1, CH98: 0->1, CH130: 1->0, CH133: 0->1;
DELTRAN:
CH124: 0->1;
ACCTRAN:
CH109: 1->2, CH112: 1->0, CH118: 2->0, CH124: 0->1, CH183: 1->0.

Jurassichelon oleronensis:
Unambiguous:
CH19: 0->1, CH82: 1->0, CH96: 0->1, CH149: 2->1;

DELTRAN:
CH76: 0->1, CH112: 1->0, CH299: 2->1.

Portlandemys mcdowelli:
Unambiguous:
CH63: 1->0, CH95: 1->0, CH101: 1->0, CH157: 1->0;
DELTRAN:
CH183: 1->0.

Plesiochelys planiceps:
Unambiguous:
CH35: 1->2, CH142: 0->1;
DELTRAN:
CH109: 2->1, CH118: 0->2, CH180: 0->1.

(*Plesiochelys etalloni* + *Plesiochelys bigleri*):
Unambiguous:
CH63: 1->0, CH219: 1->0;
DELTRAN:
CH210: 0->1, CH225: 1->0;
ACCTRAN:
CH45: 0->1, CH180: 1->0.

Plesiochelys etalloni:
Unambiguous:
CH106: 1->0, CH142: 0->1, CH145: 1->0;
DELTRAN:
CH45: 0->1;
ACCTRAN:
CH109: 1->2.

Plesiochelys bigleri:
Unambiguous:
CH144: 1->0, CH149: 2->1;
DELTRAN:
CH109: 2->1, CH325: 0->1.

Sandownidae:
Unambiguous:
CH17: 0->1, CH35: 1->0, CH58: 0->1, CH69: 0->1, CH110: 0->1, CH111: 0->1, CH141: 1->0;
DELTRAN:
CH57: 0->1, CH100: 0->1, CH134: 0->1, CH175: 0->1;
ACCTRAN:
CH22: 1->2, CH78: 1->2, CH91: 2->1, CH98: 0->1, CH107: 0->1, CH282: 0->1.

(*Brachyopsemys* ++ *Sandownia*):
Unambiguous:
CH2: 0->1, CH50: 1->0, CH63: 1->0;
DELTRAN:
CH68: 0->2;
ACCTRAN:
CH49: 0->1, CH144: 1->0.

(*Sandownia* + *Angolachelys*):
Unambiguous:

CH56: 0->1, CH112: 1->0, CH118: 0->1;
DELTRAN:
CH22: 1->2, CH78: 1->2;
ACCTRAN:
CH39: 0->1, CH55: 0->1, CH97: 0->1, CH107: 1->0.

Sandownia harrisi:
Unambiguous:
CH7: 0->1, CH17: 1->0, CH149: 2->1;
DELTRAN:
CH55: 0->1, CH91: 2->1, CH97: 0->1, CH124: 0->1, CH129: 1->0;
ACCTRAN:
CH49: 1->0.

Angolachelys mbaxi:
Unambiguous:
CH29: 0->1, CH48: 1->0, CH95: 0->2, CH106: 1->0, CH111: 1->0, CH134: 1->2;
DELTRAN:
CH39: 0->1, CH49: 0->1.

Brachyopsemys tingitana:
Unambiguous:
CH65: 0->1, CH72: 0->1, CH117: 0->1, CH141: 0->2, CH163: 0->1, CH165: 1->0;
DELTRAN:
CH49: 0->1, CH107: 0->1;
ACCTRAN:
CH22: 2->1, CH78: 2->1.

Leyvachelys cipadi:
Unambiguous:
CH3: 1->0, CH7: 0->1, CH28: 1->0, CH143: 0->1;
DELTRAN:
CH22: 1->2, CH78: 1->2, CH107: 0->1, CH122: 0->1, CH144: 0->1, CH282: 0->1.

crown-group Pleurodira:
Unambiguous:
CH5: 0->1, CH6: 0->1, CH42: 0->1, CH71: 0->1, CH80: 2->0, CH88: 0->1, CH92: 1->0, CH94: 0->1, CH106: 1->0, CH108: 1->2, CH109: 2->0, CH160: 0->1, CH193: 0->3, CH199: 0->1, CH222: 1->0, CH253: 0->1, CH259: 0->1, CH278: 0->1, CH281: 1->0, CH284: 0->1, CH318: 0->1, CH328: 1->0;
DELTRAN:
CH29: 0->1, CH36: 0->1, CH98: 1->0, CH124: 0->1, CH134: 0->1, CH210: 0->1, CH288: 2->0, CH319: 1->0;
ACCTRAN:
CH2: 0->1, CH24: 1->0, CH78: 1->3, CH112: 1->0, CH129: 0->2, CH180: 1->0, CH208: 0->1, CH224: 0->1, CH240: 1->0.

total-group Chelidae:
Unambiguous:
CH7: 0->1, CH73: 1->0, CH125: 0->2, CH153: 0->1, CH156: 1->0, CH174: 0->1, CH182: 1->0, CH192: 0->1, CH296: 0->1;
DELTRAN:
CH129: 1->2;
ACCTRAN:
CH105: 1->0, CH155: 1->0, CH258: 0->1, CH334: 1->0.

crown-group Chelidae:
Unambiguous:
CH4: 1->0, CH32: 0->1, CH35: 1->2, CH63: 1->0, CH187: 1->0, CH288: 0->3, CH289: 0->1;
DELTRAN:
CH2: 0->1, CH78: 1->3, CH208: 0->1;
ACCTRAN:
CH1: 1->0, CH16: 1->0, CH116: 0->1, CH118: 0->2, CH119: 0->2, CH184: 0->1, CH224: 1->0, CH240: 0->1, CH345: 0->1.

Chelinae:
Unambiguous:
CH165: 1->0;
DELTRAN:
CH112: 1->0, CH116: 0->1, CH118: 0->3, CH119: 0->2, CH122: 0->1;
ACCTRAN:
CH118: 2->3.

Chelus fimbriatus:
Unambiguous:
CH6: 1->0, CH23: 1->0, CH49: 0->1, CH130: 1->0, CH150: 1->0, CH161: 1->0, CH190: 0->1, CH191: 0->1, CH202: 1->0, CH210: 1->0, CH226: 1->0, CH257: 1->3, CH267: 0->2, CH269: 0->1, CH272: 0->1, CH282: 0->1, CH283: 0->1;
DELTRAN:
CH1: 0->1, CH258: 0->1, CH345: 0->1;
ACCTRAN:
CH1: 0->1, CH155: 0->1.

Phrynops geoffroanus:
Unambiguous:
CH25: 0->1, CH143: 0->1, CH192: 1->0, CH205: 0->1, CH296: 1->0;
DELTRAN:
CH105: 0->1, CH155: 1->0, CH184: 0->1;
ACCTRAN:
CH105: 0->1, CH258: 1->0, CH345: 1->0.

Chelodininae:
Unambiguous:
CH209: 0->1, CH211: 0->1, CH347: 1->0;
DELTRAN:
CH118: 0->2, CH345: 0->1;
ACCTRAN:
CH122: 1->0, CH331: 0->1.

Elseya dentata:
Unambiguous:
CH17: 0->1, CH19: 2->0, CH24: 0->1, CH25: 0->1, CH59: 0->2, CH61: 0->1, CH174: 1->0, CH176: 0->1, CH177: 1->0, CH185: 0->1, CH253: 1->0, CH346: 0->1;
DELTRAN:
CH184: 0->1, CH224: 0->1, CH334: 0->1;
ACCTRAN:
CH112: 0->1, CH116: 1->0, CH119: 2->0, CH155: 0->1, CH224: 0->1, CH258: 1->0, CH334: 0->1.

Chelodina:
Unambiguous:
CH13: 0->1, CH83: 0->1, CH130: 1->0, CH181: 1->0, CH257: 1->0, CH267: 0->1;

DELTRAN:
CH16: 0->1, CH105: 0->1, CH112: 1->0, CH116: 0->1, CH119: 0->2, CH155: 1->0, CH258: 0->1, CH331: 0->1;
ACCTRAN:
CH16: 0->1, CH105: 0->1, CH184: 1->0.

Araripemys barretoi:
Unambiguous:
CH39: 0->1, CH74: 0->1, CH96: 1->2, CH97: 0->1, CH117: 1->0, CH181: 1->0, CH190: 0->1, CH191: 0->1, CH193: 3->4, CH212: 0->1, CH232: 0->1, CH235: 0->1, CH236: 0->1, CH237: 0->1, CH248: 0->1, CH249: 0->1, CH257: 1->0, CH266: 0->1, CH276: 0->1, CH346: 0->1;
DELTRAN:
CH1: 0->1, CH16: 0->1, CH122: 0->1, CH155: 1->0, CH224: 0->1, CH240: 1->0, CH258: 0->1;
ACCTRAN:
CH78: 3->1, CH208: 1->0.

total-group Pelomedusoides:
Unambiguous:
CH22: 1->2, CH55: 0->1, CH83: 0->1;
DELTRAN:
CH1: 0->1, CH16: 0->1;
ACCTRAN:
CH25: 0->1, CH99: 0->1, CH122: 1->0, CH150: 1->0, CH177: 1->0, CH254: 1->0, CH347: 1->0.

crown-group Pelomedusoides:
Unambiguous:
CH59: 0->2, CH62: 1->2, CH74: 0->1, CH165: 1->0;
DELTRAN:
CH78: 1->3, CH129: 1->2, CH177: 1->0, CH208: 0->1, CH224: 0->1, CH240: 1->0, CH254: 1->0, CH256: 0->1, CH334: 0->1, CH347: 1->0;
ACCTRAN:
CH24: 0->1, CH112: 0->1.

Podocnemis:
Unambiguous:
CH19: 2->0, CH31: 0->1, CH43: 0->1, CH56: 0->1, CH97: 0->1, CH117: 1->0, CH118: 0->2, CH155: 1->0, CH160: 1->0, CH272: 0->1, CH331: 0->1, CH345: 0->1, CH346: 0->1;
DELTRAN:
CH24: 0->1, CH25: 0->1, CH99: 0->1, CH150: 1->0;
ACCTRAN:
CH105: 1->0.

Pelomedusa subrufa:
Unambiguous:
CH23: 1->0, CH39: 0->1, CH125: 0->2, CH126: 0->1, CH143: 0->1, CH145: 1->0, CH153: 0->1, CH156: 1->0, CH311: 1->0;
DELTRAN:
CH105: 0->1;
ACCTRAN:
CH25: 1->0, CH99: 1->0, CH150: 0->1.

Galianemys whitei:
Unambiguous:
CH33: 0->1, CH35: 1->0, CH44: 0->1, CH85: 0->1, CH115: 0->1, CH133: 0->1, CH140: 0->2, CH154: 1->0;

DELTRAN:
CH25: 0->1, CH78: 1->2, CH99: 0->1, CH105: 0->1, CH112: 1->0, CH150: 1->0;
ACCTRAN:
CH78: 3->2, CH129: 2->1.

(*Judithemys* ++ *Sinemys*):
Unambiguous:
CH19: 2->1, CH136: 0->1, CH246: 1->2, CH248: 0->1, CH266: 0->1;
DELTRAN:
CH39: 0->1, CH282: 0->1, CH283: 0->1;
ACCTRAN:
CH41: 1->0, CH115: 0->1, CH133: 0->2, CH181: 1->0, CH249: 0->1.

(*Sinemys* ++ *Kirgizemys*):
Unambiguous:
CH278: 0->1;
DELTRAN:
CH115: 0->1;
ACCTRAN:
CH35: 1->2, CH91: 2->1, CH98: 1->0.

Sinemydidae:
Unambiguous:
CH111: 0->1, CH212: 0->1, CH224: 0->1, CH232: 0->1, CH299: 2->0;
ACCTRAN:
CH4: 1->0, CH117: 1->0, CH192: 0->1, CH226: 1->0, CH236: 0->1, CH249: 1->0, CH261: 0->1, CH287: 1->0.

(*Ordosemys* + *Dracochelys*):
Unambiguous:
CH82: 0->1, CH118: 0->2;
ACCTRAN:
CH35: 2->1, CH98: 0->1, CH154: 1->0, CH194: 0->1, CH220: 0->1.

Ordosemys sp. IVPP V12092:
Unambiguous:
CH81: 0->1, CH84: 0->1, CH101: 0->2;
DELTRAN:
CH4: 1->0, CH117: 1->0, CH154: 1->0, CH261: 0->1.

Dracochelys bicuspis:
Unambiguous:
CH54: 0->1, CH59: 0->1, CH66: 2->0, CH136: 1->0, CH148: 1->0;
DELTRAN:
CH194: 0->1, CH220: 0->1, CH226: 1->0, CH236: 0->1, CH287: 1->0;
ACCTRAN:
CH4: 0->1, CH261: 1->0.

(*Sinemys gamera* + *Sinemys lens*):
Unambiguous:
CH7: 0->1, CH19: 1->2, CH78: 1->2, CH92: 1->0;
DELTRAN:
CH4: 1->0, CH16: 0->1, CH35: 1->2, CH98: 1->0, CH133: 0->2, CH208: 0->1;
ACCTRAN:
CH25: 0->1, CH39: 1->0, CH74: 0->1, CH283: 1->0, CH332: 0->1.

Sinemys gamera:
Unambiguous:
CH2: 0->1;
DELTRAN:
CH25: 0->1, CH36: 0->1, CH74: 0->1, CH192: 0->1, CH332: 0->1.

Sinemys lens:
Unambiguous:
CH109: 2->1;
DELTRAN:
CH39: 1->0, CH261: 0->1, CH283: 1->0.

(*Kirgizemys hoburensis* + *Kirgizemys dmitrievi*):
Unambiguous:
CH151: 0->1;
DELTRAN:
CH35: 1->2, CH249: 0->1;
ACCTRAN:
CH16: 1->0, CH36: 1->0, CH109: 2->1, CH156: 1->0, CH205: 0->1, CH295: 0->1.

Kirgizemys hoburensis:
Unambiguous:
CH41: 0->1, CH165: 1->0;
DELTRAN:
CH24: 0->1, CH109: 2->1, CH156: 1->0, CH205: 0->1, CH295: 0->1.

Kirgizemys dmitrievi:
Unambiguous:
CH14: 1->0, CH59: 0->1, CH62: 1->0, CH66: 2->1, CH80: 1->2, CH142: 0->1, CH163: 0->1;
DELTRAN:
CH98: 1->0.

Judithemys sukhanovi:
Unambiguous:
CH76: 0->1, CH297: 0->1;
DELTRAN:
CH91: 1->2, CH249: 0->1.

Xinjiangchelyidae:
Unambiguous:
CH92: 1->0, CH127: 0->1, CH205: 0->1, CH264: 0->1, CH277: 0->1;
DELTRAN:
CH39: 0->1, CH111: 0->1, CH181: 0->1, CH208: 0->1;
ACCTRAN:
CH35: 1->2, CH109: 2->1, CH154: 1->0, CH231: 0->1, CH301: 1->0, CH344: 1->0, CH345: 0->1.

Annemys levensis:
Unambiguous:
CH138: 0->1, CH210: 0->1, CH230: 1->0;
DELTRAN:
CH4: 0->1, CH35: 1->2, CH109: 2->1, CH231: 0->1;
ACCTRAN:
CH177: 1->0.

(*Annemys latiens* ++ *Xinjiangchelys*):
Unambiguous:
CH7: 0->1, CH147: 1->0;
ACCTRAN:
CH4: 1->0, CH118: 0->2.

Annemys latiens:
Unambiguous:
CH89: 1->0, CH123: 0->1;
DELTRAN:
CH231: 0->1.

(*Annemys* sp. IVPP V18106 ++ *Xinjiangchelys*):
Unambiguous:
CH2: 0->1;
DELTRAN:
CH118: 0->2;
ACCTRAN:
CH231: 1->0.

Annemys sp. IVPP V18106:
Unambiguous:
CH82: 0->1, CH98: 1->0, CH133: 0->1, CH145: 1->0, CH157: 0->1;
DELTRAN:
CH35: 1->2, CH41: 0->1, CH109: 2->1, CH154: 1->0.

(*Xinjiangchelys wusu* + *Xinjiangchelys radiplicatoides*):
Unambiguous:
CH76: 0->1, CH77: 2->1;
ACCTRAN:
CH35: 2->1, CH63: 0->1, CH109: 1->2, CH125: 1->0, CH141: 1->2.

Xinjiangchelys wusu:
Unambiguous:
CH264: 1->0;
DELTRAN:
CH345: 0->1;
ACCTRAN:
CH154: 0->1.

*Xinjiangchelys radiplicatoide*s:
Unambiguous:
CH96: 1->2, CH111: 1->0, CH187: 0->1, CHs 92: 0->1.

Xinjiangchelys radiplicatoides:
DELTRAN:
CH63: 0->1, CH125: 1->0, CH141: 1->2, CH154: 1->0, CH177: 0->1.

Paracryptodira:
Unambiguous:
CH7: 0->1, CH44: 0->1, CH147: 1->2;
DELTRAN:
CH25: 0->1, CH111: 0->1;
ACCTRAN:

CH2: 0->1, CH25: 0->1, CH29: 0->1, CH59: 0->1, CH66: 2->0, CH73: 1->0, CH149: 1->0, CH192: 0->1, CH235: 1->0, CH251: 0->1, CH254: 1->0, CH256: 1->0, CH257: 0->1, CH284: 0->1, CH311: 0->1.

(*Eubaena* + *Pleurosternidae*):
Unambiguous:
CH118: 0->2, CH133: 0->1, CH139: 0->1;
DELTRAN:
CH29: 0->1;
ACCTRAN:
CH70: 0->1, CH124: 0->1, CH130: 0->1, CH135: 0->1.

Eubaena cephalica:
Unambiguous:
CH7: 1->2, CH12: 1->0, CH16: 0->1, CH76: 0->1, CH88: 0->1, CH91: 1->2, CH131: 0->1, CH164: 0->1;
DELTRAN:
CH70: 0->1;
ACCTRAN:
CH2: 1->0, CH66: 0->2.

Pleurosternidae:
Unambiguous:
CH3: 0->1, CH19: 2->0, CH77: 2->1, CH96: 1->2, CH109: 2->1, CH134: 0->1;
DELTRAN:
CH2: 0->1, CH66: 2->0, CH124: 0->1, CH192: 0->1, CH235: 1->0, CH253: 0->1, CH257: 0->1;
ACCTRAN:
CH18: 0->1, CH81: 0->1, CH122: 2->0.

Pleurosternon bullockii:
Unambiguous:
CH36: 0->1, CH59: 1->0, CH133: 1->2, CH193: 0->3, CH222: 1->0, CH224: 0->1, CH265: 0->1;
DELTRAN:
CH18: 0->1, CH23: 0->1, CH81: 0->1, CH130: 0->1, CH135: 0->1.

Glyptops plicatulus:
DELTRAN:
CH122: 2->0, CH251: 0->1, CH284: 0->1, CH301: 0->1, CH311: 0->1.

Arundelemys dardeni:
Unambiguous:
CH107: 0->1, CH127: 0->1;
DELTRAN:
CH2: 0->1, CH66: 2->0;
ACCTRAN:
CH23: 1->0.

Meiolania planiceps:
Unambiguous:
CH26: 1->0, CH34: 0->1, CH37: 0->1, CH38: 0->1, CH59: 0->2, CH67: 0->1, CH76: 0->1, CH77: 2->0, CH78: 1->3, CH79: 0->1, CH91: 1->2, CH106: 1->0, CH113: 0->1, CH141: 1->0, CH144: 1->0, CH161: 1->0, CH163: 0->1, CH184: 0->1, CH226: 1->0, CH236: 0->1, CH285: 0->1, CH307: 0->1;
DELTRAN:
CH73: 0->1, CH123: 0->1, CH124: 0->1, CH149: 0->1, CH151: 0->1, CH157: 0->1, CH168: 0->1, CH172: 0->1, CH253: 0->1, CH254: 0->1, CH281: 0->1, CH304: 0->2, CH344: 0->1;

ACCTRAN:
CH12: 1->0, CH151: 0->1, CH157: 0->1, CH168: 0->1, CH172: 0->1, CH246: 1->0, CH281: 0->1, CH303: 1->0, CH304: 1->2.

Kallokibotion bajazidi:
Unambiguous:
CH61: 0->1, CH80: 1->2, CH119: 0->1, CH131: 0->1, CH146: 0->1, CH148: 0->1, CH177: 0->1, CH223: 0->1, CH258: 0->2, CH312: 0->1;
DELTRAN:
CH12: 0->1, CH123: 0->1, CH124: 0->1, CH246: 0->1, CH256: 0->1.

Chubutemys copelloi:
Unambiguous:
CH4: 0->1, CH28: 1->0, CH30: 1->0, CH63: 0->1, CH97: 0->1, CH284: 0->1;
DELTRAN:
CH59: 1->0;
ACCTRAN:
CH12: 1->0.

(*Kayentachelys* + *Eileanchelys*):
Unambiguous:
CH7: 0->1, CH29: 0->1, CH48: 0->1, CH111: 0->1, CH174: 0->1;
DELTRAN:
CH12: 0->1, CH134: 0->1;
ACCTRAN:
CH90: 1->0, CH136: 0->1, CH157: 0->1, CH208: 0->1.

Eileanchelys waldmanni:
Unambiguous:
CH205: 0->1;
DELTRAN:
CH21: 0->1, CH157: 0->1, CH167: 0->1, CH204: 0->1, CH244: 0->1.

Kayentachelys aprix:
Unambiguous:
CH85: 0->1, CH132: 2->1, CH219: 1->0, CH222: 1->0;
DELTRAN:
CH25: 0->1, CH77: 0->1, CH136: 0->1, CH142: 0->1, CH208: 0->1, CH303: 0->1;
ACCTRAN:
CH21: 1->0, CH167: 1->0, CH204: 1->0, CH244: 1->0.

Australochelys africanus:
Unambiguous:
CH131: 0->1;
DELTRAN:
CH25: 0->1, CH75: 0->1, CH90: 0->1, CH134: 0->1, CH142: 0->1.

Proganochelys quenstedti:
DELTRAN:
CH43: 0->1, CH70: 0->1, CH163: 0->1, CH210: 0->1, CH269: 0->1, CH281: 0->1;
ACCTRAN:
CH43: 0->1, CH70: 0->1, CH163: 0->1, CH210: 0->1, CH269: 0->1, CH281: 0->1,

PCA DATA

TABLE S1.3. Measurements from different specimens of *Rhinochelys* specimens used in our Principal Component Analyses. PCA 1 is the analysis including only measurements that were used by Collins (1970) for her taxonomic assessment of *Rhinochelys*; PCA 2 includes all measurements taken in this study.

Specimen	Right nasal length–width ratio	Left nasal length–width ratio	Jaw angle (in °)	Width (mm)	Orbital width (mm)	Pre-parietal length (mm)	Height (mm)	PCA 1	PCA 2
CAMSM B55773	?	?	?	40	22	22	20	-	-
CAMSM B55774	1.38	1.10	41	32	15	24	17	included	included
CAMSM B55775	1.33	1.69	49	39	18	26	21	included	included
CAMSM B55776	1.18	1.08	44	30	14	21	18	included	included
CAMSM B55781	1.46	1.81	47	NA	22	NA	13	-	-
CAMSM B55782	1.22	1.13	43	19	9	14	12	included	included
CAMSM B55783	0.89	0.91	47	23	12	17	13	included	included
CAMSM B55784	1.27	1.34	48	30	16	22	18	included	included
CAMSM B55785	1.07	0.94	42	27	14	18	14	included	included
CAMSM B55786	0.73	0.83	43	27	12	17	16	included	included
CAMSM B55787	1.00	1.07	43	24	11	16	16	included	included
CAMSM B55788	1.10	1.10	47	NA	NA	NA	NA	-	-
CAMSM B55791	1.07	0.96	36	20	11	15	10	included	included
CAMSM B55792	1.31	1.31	43	NA	14	25	23	-	-
CAMSM B55793	?	?	40	19	11	14	11	included	-
CAMSM B55794	1.20	1.15	45	21	13	19	16	included	included
CAMSM B55795	0.88	0.85	40	NA	NA	NA	NA	-	-
CAMSM B55796	0.96	NA	47	NA	NA	NA	NA	-	-
CAMSM B55799	?	?	?	15	11	14	11	-	-
CAMSM B56274	NA	NA	39	31	NA	NA	24	-	-
CAMSM B56397	?	?	37	22	11	17	16	included	-
CAMSM B56570	1.18	1.29	33	40	17	25	19	included	included
CAMSM B56571	1.38	1.50	41	30	13	17	17	included	included
CAMSM B56572	NA	1.36	43	26	11	16	14	included	-

Specimen	Right nasal length–width ratio	Left nasal length–width ratio	Jaw angle (in °)	Width (mm)	Orbital width (mm)	Pre-parietal length (mm)	Height (mm)	PCA 1	PCA 2
CAMSM B56573	?	?	43	30	14	20	16	included	-
CAMSM B56574	0.80	0.92	47	22	12	16	13	included	included
CAMSM B56575	1.26	1.40	45	39	18	27	20	included	included
CAMSM B56576	?	?	?	22	11	16	14	-	-
CAMSM B56578	1.17	1.13	41	22	11	NA	15	-	-
CAMSM B56583	0.79	0.87	46	20	10	17	12	included	included
NHMUK PV R11521	1.14	1.04	47	20	11	14	17	included	included
NHMUK PV R1558	1.13	1.13	?	NA	15	NA	20	-	-
NHMUK PV R1806	1.67	1.55	37	27	14	20	NA	-	-
NHMUK PV R2224	0.69	1.09	47	28	19	30	22	included	included
NHMUK PV R2225	0.74	0.74	37	NA	NA	NA	NA	-	-
NHMUK PV R2226	1.11	1.20	48	40	19	30	26	included	included
NHMUK PV R2227	1.50	1.44	47	24	11	18	16	included	included
NHMUK PV R2228	1.72	1.72	45	NA	16	NA	NA	-	-
NHMUK PV R2229	1.48	1.40	NA	NA	NA	NA	NA	-	-
NHMUK PV R2230	1.79	1.67	?	NA	NA	NA	NA	-	-
NHMUK PV R2231	1.62	1.71	38	NA	NA	NA	NA	-	-
NHMUK PV R2233	0.92	1.04	38	13	10	20	16	included	included
NHMUK PV R2234	0.91	1.04	40	23	11	19	18	included	included
NHMUK PV R2235	1.19	1.28	62	NA	NA	NA	NA	-	-
NHMUK PV R2236	1.34	1.28	?	NA	NA	NA	NA	-	-
NHMUK PV OR35193	1.85	1.70	47	NA	22	36	NA	-	-
NHMUK PV OR35194	1.77	1.56	48	NA	24	32	NA	-	-
NHMUK PV OR35195	NA	NA	38	NA	NA	NA	NA	-	-
NHMUK PV OR35196	1.28	1.39	47	NA	15	NA	18	-	-
NHMUK PV	1.04	1.15	43	20	11	15	14	included	included

Specimen	Right nasal length–width ratio	Left nasal length–width ratio	Jaw angle (in °)	Width (mm)	Orbital width (mm)	Pre-parietal length (mm)	Height (mm)	PCA 1	PCA 2
OR35197									
NHMUK PV OR41796	1.59	1.70	54	NA	21	31	20	-	-
NHMUK PV OR43980	1.00	1.00	56	30	17	22	21	included	included
NHMUK PV OR46371a	0.71	0.73	43	21	11	19	16	included	included
NHMUK PV R8339	1.30	1.30	45	24	12	21	18	included	included
UJF-ID.11167	0.92	0.96	57	43	20	28	29	included	included

PCA USING MEASUREMENTS OF COLLINS (1970)

Methods

As described in the main text section on PCA in Evers et al. 2018*a,* we carried out a PCA analysis using the four measurements reported by Collins (1970) to be of taxonomic importance. These were the skull width, the skull height, the pre-parietal skull length and the 'jaw angle'. 31 specimens were well enough preserved the retrieve all four measurements. The PCA analysis was conducted in R (R Core Team 2016), using the *prcomp* command. The data was transformed to have unit variance with the 'Scale = TRUE' argument, which was necessary as one of our measurements (the 'jaw angle') is in different units that the other measurements. As in the second analysis, the first Principal Component (PC1) described size and allometric shape changes (see Results), we excluded it for constructing bivarite morphospace plots and re-scaled the absolute variance explained by the remaining PCs to represent the proportion of non-size (i.e. shape) variation.

Results

Our PCA results are shown in Table S1.4. PC1 explains 72.1% of the variance in the data. As the eigenvector coefficients have the same sign, indicating relative increases/decreases in all measurements, PC1 represents size and allometric changes. We re-scaled the remaining PC axes to exclude size and allometric changes. PC2 explains 68.4% of shape variance in the data, and describes relative increases in skull size with relative decreases in the 'jaw angle'. PC3 explains 21.1% of the shape variance, and largely accounts for relative increases in skull length and height with relative decreases in skull width. PC3 explains 10.5% of the shape variance, and is associated with relative increases in skull length and decreases in skull height.

As is the case for the PCA including all measurements (see main text, Evers *et al.* 2018*a*), the data only using the measurements of Collins (1970) does not show clusters of specimens that have been reported to belong to the same species, and also no previously unrecognized clusters (Fig. S1.34). Instead, the data is seemingly randomly distributed, and the holotype specimens are relatively closely clustered.

Table S1.4. PCA results from an analysis using four cranial measurements (n=31).

	PC1	PC2	PC3	PC4
Eigenvalues	2.884	0.736	0.236	0.117
Proportion of total variance explained	0.721	0.191	0.059	0.029
Proportion of shape variance explained	-	0.684	0.211	0.105
Eigenvector coefficients				
Jaw angle	0.357	-0.904	0.123	-0.199
Width	0.519	0.313	0.796	0.008
Pre-parietal length	0.539	0.292	-0.459	-0.643
Heigth	0.539	0.007	-0.375	0.739

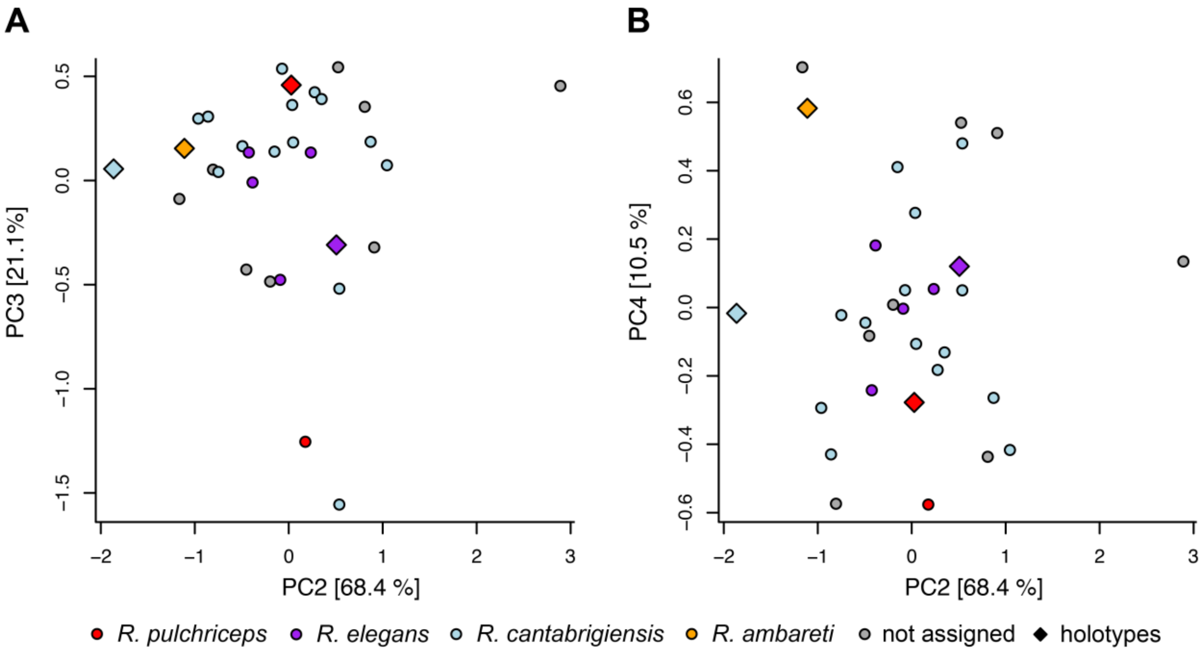

FIG. S1.34. <Collins_PCA>. Distribution of specimens of *Rhinochelys* (n = 31) in cranial geometry morphospaces recovered by Principal Component Analysis of four cranial measurements. **A**, PC1 vs. PC2; **B**, PC1 vs. PC3.

INSTITUTIONAL ABBREVIATIONS

ALMNH, Alabama Museum of Natural History, Tuscaloosa, AL, USA; **AMNH**, American Museum of Natural History, New York City, NY, USA; **AUMP**, Auburn University Museum of Paleontology, Auburn, AL, USA; **BP**, Bernhard Price Institute, Johannesburg, South Africa; **BSPG**, Bayerische Staatsammlung für Paläontologies und Geologie, Munich, Germany; **CAMSM**, Sedgwick Museum of Earth Sciences, Cambridge, UK; **CCNHM**, College of Charleston Natural History Museum, Charleston, SC, USA; **CM**, Carnegie Museum of Natural History, Pittsburgh, PA, USA; **CMM**, Carter County Museum, Ekalaka, MT, USA; **CMNH**, Cleveland Museum of Natural History, Cleveland, OH, USA; **DMNH**, Denver Museum of Nature and Science, Denver, CO, USA; **FCG–CBP**, Fundación Colombiana de Geobiología, Dentro de Investigaciones Paleontológicas, Villa de Leyva, Colombia; **FMNH**, Field Museum of Natural History, Chicago, IL, USA; **FUM**, Fur Museum (Museum Salling), Fur, Denmark; **FWMSH**, Fort Worth Museum of Science and History, Forth Worth, TX, USA; **IVPP**, Institute of Vertebrate Paleontology and Paleoanthropology, Beijing, China; **IRSNB**, Royal Belgian Institute of Natural Sciences, Brussels, Belgium; **IW**, Ingmar Werneburg private collection; **JM**, Jura-Museum, Eichstätt, Germany; **KUVP**, University of Kansas, Lawrence, KS, USA; **MAJ**, Musée d'archéologie du Jura, Lons-le-Saunier, France; **MCZ**, Museum of Comparative Zoology, Harvard University, Cambridge, MA, USA; **MGUAN-PA**, Museo Geológico da Universidade Agistino Neto, Luanda, Angola (Paleo-Angola Project Collection); **MIWG**, Museum of Isle of Wight Geology, Sandown, Isle of Wight; **MJSN**, JURASSICA Museum, Porrentruy, Switzerland; **MNA**, Museum of Northern Arizona, Flagstaff, AZ, USA; **MNB**, Naturhistorisches Museum Basel, Basel, Switzerland; **MPEF**, Museo Paleontológico Egidio Feruglio, Trelew, Argentina; **MRAC**, Musée Royal de l'Afrique Centrale, Tervuren, Belgium; **MSC**, McWane Science Center, Birmingham, AL, USA; **NHMM**, Natuurhistorisch Museum Maastricht, Maastricht, Netherlands; **NHMUK**, Natural History Museum, London, UK; **NMS**, National Museum of Scotland, Edinburgh, UK; **OCPDEK**, Office Chérifien des Phosphates, Khouribga, Morocco; **OUMNH**, Oxford University Museum of Natural History, Oxford, UK; **PIMUZ**, Paläontologisches Institut und Museum der Universität Zürich, Zurich, Switzerland; **PIN**, Paleontological Insitute, Russian Academy of Sciences, Moscow, Russia; **PMOL**, Paleontological Museum of Liaoning, Shenyang Normal University, China; **QM**, Queensland Museum, Brisbane, Australia; **SMF**, Senckenberg Museum Frankfurt, Frankfurt, Germany; **SMNS**, Staatliches Museum für Naturkunde, Stuttgart, Germany; **SMU**, Shuler Museum of Paleontology, Southern Methodist University, Dallas, TX, USA; **TM**, Teylers Museum, Haarlem, Netherlands; **TMM**, Texas Memorial Museum, Austin, TX, USA; **TMP**, Royal Tyrell Museum, Drumheller, AB, Canada; **UCMP**, University of California Museum of Paleontology, Berkeley, CA, USA; **UJF**, Universite Joseph Fourier, Grenoble, France; **UMZC**, University Museum of Zoology, Cambridge, UK; **USNM**, United States National Museum, Washington, DC, USA; **WGJ**, Walter Joyce private collection; **YPM**, Yale Peabody Museum, New Haven, CT, USA; **ZIN PH**, Zoological Insitute of Russian Academy of Sciences palaeoherpotological collection, St. Petersburg, Russia.

REFERENCES

Anquetin J. 2010. The anatomy of the basal turtle *Eileanchelys waldmani* from the Middle Jurassic of the Isle of Skye, Scotland. *Earth and Environmental Science Transactions of the Royal Society of Edinburgh* **101**:67–96. DOI: 10.1017/S1755691010009217

Anquetin J, Deschamps S, Claude J. 2014. The rediscovery and redescription of the holotype of the Late Jurassic turtle *Plesiochelys etalloni*. *PeerJ* **2**:e258. DOI: 10.7717/peerj.258

Bardet N, Jalil NE, De Lapparent De Broin F, Germain D, Lambert O, Amaghzaz M. 2013. A giant chelonioid turtle from the Late Cretaceous of Morocco with a suction feeding apparatus unique among tetrapods. *PLoS ONE* **8**:1–10. DOI: 10.1371/journal.pone.0063586

Bever GS. 2009. Postnatal ontogeny of the skull in the extant North American turtle *Sternotherus odoratus* (Cryptodira, Kinosternidae). *Bulletin of the American Museum of Natural History* **330**:1–97. DOI: 10.1206/330.1

Brinkman DB. 2001. New material of *Dracochelys* (Eucryptodira: Sinemydidae) from the Junggar Basin, Xinjiang, People's Republic of China. *Canadian Journal of Earth Sciences* **38**:1645–1651. DOI: 10.1139/e01-047

Brinkman DB, Wu X-C. 1999. The skull of *Ordosemys*, an early Cretaceous turtle from Inner Mongolia, People's Republic of China, and the interrelationships of Eucryptodira (Chelonia, Cryptodira). *Paludicola* **2**:134–147.

Brinkman DB, Hart M, Jamniczky H, Colbert M. 2006. *Nichollsemys baieri* gen. et sp. nov., a primitive chelonioid turtle from the Late Campanian of North America. *Paludicola* **5**:111–124.

Brinkman DB, Eberth DA, Xu X, Clark JM, Wu X-C. 2013. Turtles from the Jurassic Shishugou Formation of the Junggar Basin, People's Republic of China, with comments on the basicranial region of basal eucryptodires. In: Brinkman DB, Holroyd PA, Gardner JD, eds. *Morphology and Evolution of Turtles*. Dordrecht: Springer, 147–172.

Cadena E. 2015. The first South American sandownid turtle from the Lower Cretaceous of Colombia. *PeerJ* **3**:e1431. DOI: 10.7717/peerj.1431

Cadena E, Parham JF. 2015. Oldest known marine turtle? A new protostegid from the Lower Cretaceous of Colombia. *PaleoBios* **32**:1–421. Available at: https://escholarship.org/uc/item/147611bv

Case EC. 1897. On the osteology and relationships of *Protostega*. *Journal of Morphology* **14**:21–60. DOI: 10.1002/jmor.1050140103

Casier E. 1968. Le squelette cephalique de *Eochelone brabantica* L. Dollo, du Bruxellien

(Lutetien inferieur) de Belgique, et sa comparaison avec celui de *Chelone mydas* Linne. *Bulletin de l'Institut Royal des Sciences Naturelles de Belgique, Sciences de la Terre* **44**:1–22.

Collins JI. 1970. The chelonian *Rhinochelys* Seeley from the Upper Cretaceous of England and France. *Palaeontology* **13**:355–378.

Danilov IG, Averianov AO, Skutchas PP, Rezvyi AS. 2006. *Kirgizemys* (Testudines, 'Macrobaenidae'): New material from the Lower Cretaceous of Buryatia (Russia) and taxonomic revision. In: Danilov IG, Parham JF, eds. *Fossil Turtle Research. Proceedings of the Symposium on Turtle Origins, Evolution and Systematics*, St. Petersburg.

Evans J, Kemp TS. 1975. The cranial morphology of a new Lower Cretaceous turtle from southern England. *Palaeontology* **18**:1–25.

Evers SW, Benson RBJ. 2018*a*. A new phylogenetic hypothesis of turtles with implications for the number of evolutionary transitions to marine lifestyles supports an Early Cretaceous origin and rapid diversification of Chelonioidea. *Palaeontology*: 1–42. DOI: 10.1111/pala.12384

Gaffney ES. 1975*a*. A taxonomic revision of the Jurassic turtles *Portlandemys* and *Plesiochelys*. *American Museum Novitates* **2574**:1–19. Available at: http://hdl.handle.net/2246/2761

Gaffney ES. 1972. An illustrated glossary of turtle skull nomenclature. *American Museum Novitates* **2486**:1–33. Available at: http://hdl.handle.net/2246/2694

Gaffney ES. 1975*b*. *Solnhofia parsonsi*, a new cryptodiran turtle from the late Jurassic of Europe. *American Museum Novitates* **2576**:1–25. Available at: http://hdl.handle.net/2246/2763

Gaffney ES. 1976. Cranial morphology of the European Jurassic turtles *Portlandemys* and *Plesiochelys*. *Bulletin of the American Museum of Natural History* **157**:487–544. Available at: http://hdl.handle.net/2246/1204

Gaffney ES. 1979. Comparative cranial morphology of recent and fossil turtles. *Bulletin of the American Museum of Natural History* **164**:65–376. Available at: http://hdl.handle.net/2246/565

Gaffney ES. 1982*a*. Cranial morphology of the baenid turtles. *American Museum Novitates* **2737**:1–22. Available at: http://hdl.handle.net/2246/5347

Gaffney ES. 1982*b*. The lower jaws of baenid turtles. *American Museum Novitates* **2749**:1–10. Available at: http://hdl.handle.net/2246/5297

Gaffney ES. 1990. The comparative osteology of the Triassic turtle *Proganochelys*. *Bulletin*

of the American Museum of Natural History **194**:1–176. Available at: http://hdl.handle.net/2246/884

Gaffney ES, Jenkins JR FA. 2010. The cranial morphology of *Kayentachelys*, an Early Jurassic cryptodire, and the early history of turtles. *Acta Zoologica* **91**:335–368. DOI: 10.1111/j.1463-6395.2009.00439.x

Gaffney ES, Kitching JW. 1995. The morphology and relationships of *Australochelys*, an early Jurassic turtle from South Africa. *American Museum Novitates* **3130**:1–29. Available at: http://hdl.handle.net/2246/3666

Gaffney ES, Meylan PA. 1992. The Transylvanian turtle, *Kallokibotion*, a primitive cryptodire of Cretaceous Age. *American Museum Novitates* **3040**:1–37. Available at: http://hdl.handle.net/2246/5005

Gaffney ES, Ye X. 1992. *Dracochelys*: a new cryptodiran turtle from the early Cretaceous of China. *American Museum Novitates* **3048**:1–13. Available at: http://hdl.handle.net/2246/5015

Gaffney ES, Zangerl R. 1968. A revision of the chelonian genus *Bothremys* (Pleurodira: Pelomedusidae). *Fieldiana. Geology Memoirs* **16** (7):193–239. DOI: 10.5962/bhl.title.5195

Gaffney ES, Tong H, Meylan PA. 2002. *Galianemys*, a new side-necked turtle (Pelomedusoides: Bothremydidae) from the Late Cretaceous of Morocco. *American Museum Novitates* **3379**:1–20. DOI: 10.1206/0003-0082(2002)379%3C0001:GANSNT%3E2.0.CO;2

Gaffney ES, Rich TH, Vickers-Rich P, Constantine A, Vacca R, Kool L. 2007. *Chubutemys*, a new eucryptodiran turtle from the Early Cretaceous of Argentina, and the relationships of the Meiolaniidae. *American Museum Novitates* **3599**:1–35. DOI: 10.1206/0003-0082(2007)3599[1:CANETF]2.0.CO;2

Gentry AD. 2016. New material of the Late Cretaceous marine turtle *Ctenochelys acris* Zangerl, 1953 and a phylogenetic reassessment of the 'toxochelyid'-grade taxa. *Journal of Systematic Palaeontology* **15** (8):675–696. DOI: 10.1080/14772019.2016.1217087

Gentry AD, Parham JF, Ehret DJ, Eversole JA. 2018. A new species of *Peritresius* Leidy, 1856 (Testudines: Pan-Cheloniidae) from the Late Cretaceous (Campanian) of Alabama, USA, and the occurrence of the genus within the Mississippi Embayment of North America. *PLoS ONE* **13** (4):e0195651. DOI: 10.1371/journal.pone.0195651

Havlik PE, Joyce WG, Böhme M. 2014. *Allaeochelys libyca* , a new carettochelyine turtle from the Middle Miocene (Langhian) of Libya. *Bulletin of the Peabody Museum of*

Natural History **55**:201–214. DOI: 10.3374/014.055.0207

Hawkins J, Hughes C, Scotland R. 1997. Primary homology assessment, characters and character states. *Cladistics* **283**:275–283. DOI: 10.1111/j.1096-0031.1997.tb00320.x

Hay OP. 1896. On the skeleton of *Toxochelys latiremis*. *Field Columbian Museum Publications, Zoological Series*, **1**. DOI: 10.5962/bhl.title.2548

Hay OP. 1908. *The fossil turtles of North America*. Carnegie Institution of Washington. DOI: 10.5962/bhl.title.12500

Hirayama R. 1992. Humeral morphology of chelonioid sea-turtles; its functional analysis and phylogenetic implications. *Bulletin of the Hobetsu Museum* **8**:17–57.

Hirayama R. 1994. Phylogenetic systematics of chelonioid sea turtles. *Island Arc* **3**:270–284. DOI: 10.1111/j.1440-1738.1994.tb00116.x

Hirayama R. 1998. Oldest known sea turtle. *Nature* **392**:705–708. DOI: 10.1038/33669

Hooks GE. 1998. Systematic revision of the Protostegidae, with a redescription of *Calcarichelys gemma* Zangerl, 1953. *Journal of Vertebrate Paleontology* **18**:85–98. DOI: 10.1080/02724634.1998.10011036

Jones MEH, Werneburg I, Curtis N, Penrose R, O'Higgins P, Fagan MJ, Evans SE. 2012. The head and neck anatomy of sea turtles (Cryptodira: Chelonioidea) and skull shape in testudines. *PLoS ONE* **7**:e47852. DOI: 10.1371/journal.pone.0047852

Joyce WG. 2000. The first complete skeleton of *Solnhofia parsoni* (Cryptodira, Eurysternidae) from the Upper Jurassic of Germany and its taxonomic implcations. *Journal of Paleontology* **74** (4):684–700.

Joyce WG. 2007. Phylogenetic relationships of Mesozoic turtles. *Bulletin of the Peabody Museum of Natural History* **48**:3–102. DOI: 10.3374/0079-032X(2007)48[3:PROMT]2.0.CO;2

Joyce WG. 2016. A review of the fossil record of turtles of the clade Pan-Chelydridae. *Bulletin of the Peabody Museum of Natural History* **57**:21–56. DOI: 10.3374/014.057.0103

Kear BP, Lee MSY. 2006. A primitive protostegid from Australia and early sea turtle evolution. *Biology letters* **2**:116–119. DOI: 10.1098/rsbl.2005.0406

Knauss GE. 2014. A morphological description of *Baptemys wyomingensis* and an analysis of its phylogenetic relationship within Kinosternoidea. Unpublished M.Sc. thesis, University of Iowa, Iowa City, 181 pp. Available at: https://ir.uiowa.edu/etd/4665/

Lipka TR, Therrien F, Weishampel DB, Jamniczky HA, Joyce WG, Colbert MW, Brinkman DB. 2006. A new turtle from the Arundel Clay facies (Potomac Formation, Early

Cretaceous) of Maryland, U.S.A. *Journal of Vertebrate Paleontology* **26**:300–307. DOI: 10.1671/0272-4634(2006)26[300:ANTFTA]2.0.CO;2

Mateus O, Jacobs L, Polcyn M, Schulp AS, Vineyard D, Buta Neto A, Telles Antunes M. 2009. The oldest African eucryptodiran turtle from the Cretaceous of Angola. *Acta Palaeontologica Polonica* **54**:581–588. DOI: 10.4202/app.2008.0063

Matzke AT. 2007. An almost complete juvenile specimen of the cheloniid turtle *Ctenochelys stenoporus* (Hay, 1905) from the Upper Cretaceous Niobrara Formation of Kansas, USA. *Palaeontology* **50** (3):669–691. DOI: 10.1111/j.1475-4983.2007.00650.x

Meylan PA. 1987. The phylogenetic relationships of soft-shelled turtles (family Trionychidae). *Bulletin of the American Museum of Natural History* **186**:1–101. Available at: http://hdl.handle.net/2246/972

Meylan PA. 1996. Skeletal morphology and relationships of the early Cretaceous side-necked turtle, *Araripemys barretoi* (Testudines: Pelomedusoides: Araripemydidae), from the Santana Formation of Brazil. *Journal of Vertebrate Paleontology* **16**:20–33. DOI: 10.1080/02724634.1996.10011280

Meylan PA, Gaffney ES. 1989. The skeletal morphology of the Cretaceous cryptodiran turtle, *Adocus*, and the relationships of the Trionychoidea. *American Museum Novitates* **2941**:1–60. Available at: http://hdl.handle.net/2246/5096

Moody RTJ. 1974. The taxonomy and morphology of *Puppigerus camperi* (Gray), an Eocene sea-turtle from Northern Europe. *Bulletin of the British Museum (Natural History), Geology* **25**:153–186. Available at: http://biostor.org/reference/118672

Mulder EWA. 2003. Comparative osteology, palaeoecology and systematics of the Late Cretaceous turtle *Allopleuron hofmanni* (Gray 1831) from the Maastrichtian type area. *Pubicaties van het Natuurhistorisch Genootschap in Limburg*, 23–92.

Myers TS, Polcyn MJ, Mateus O, Vineyard DP, Gonçalves AO, Jacobs LL. 2018. A new durophagous stem cheloniid turtle from the Lower Paleocene of Cabinda, Angola. *Papers in Palaeontology* **4** (2):161–176. DOI: 10.1002/spp2.1100

Nicholls EL. 1988. New material of *Toxochelys latiremis* Cope, and a revision of the genus *Toxochelys* (Testudines, Chelonioidea). *Journal of Vertebrate Paleontology* **8**:181–187. DOI: 10.1080/02724634.1988.10011696

Nielsen E. 1959. Eocene turtles from Denmark. *Dansk Geologisk Forening* **14**:96–114.

Nielsen E. 1963. On the post-cranial skeleton of Eosphargis breineri Nielsen. *Bulletin of the Geological Society of Denmark* **15**:281–328.

Parham JF, Hutchison JH. 2003. A new eucryptodiran turtle from the Late Cretaceous of

North America (Dinosaur Provincial Park, Alberta, Canada). *Journal of Vertebrate Paleontology* **23**:783–798. DOI: 10.1671/5

Parham JF, Pyenson ND. 2010. New sea turtle from the Miocene of Peru and the iterative evolution of feeding ecomorphologies since the Cretaceous. *Journal of Paleontology* **84**:231–247. DOI: 10.1666/09-077R.1

Pünterer C, Anquetin J, Billot-Bruyat J-P. 2017. The comparative osteology of *Plesiochelys bigleri* n. sp., a new coastal marine turtle from the Late Jurassic of Porrentruy (Switzerland). *PeerJ* **5**:e3482. DOI: 10.7717/peerj.3482

R Core Team. 2016. *R: a language and environment for statistical computing*. Version 3.3.1. R Foundation for Statistical Computing. http://www.R-project.org

Rabi M, Zhou C-F, Wings O, GE S, Joyce WG. 2013. A new xinjiangchelyid turtle from the Middle Jurassic of Xinjiang, China and the evolution of the basipterygoid process in Mesozoic turtles. *BMC Evolutionary Biology* **13**:203. DOI: 10.1186/1471-2148-13-203

Rabi M, Sukhanov VB, Egorova VN, Danilov IG, Joyce WG. 2014. Osteology, relationships, and ecology of *Annemys* (Testudines, Eucryptodira) from the Late Jurassic of Shar Teg, Mongolia, and phylogenetic definitions for Xinjiangchelyidae, Sinemydidae, and Macrobaenidae. *Journal of Vertebrate Paleontology* **34**:327–352. DOI: 10.1080/02724634.2013.807274

Rieppel O. 1980. The skull of the Upper Jurassic cryptodire turtle *Thalassemys*, with a reconsideration of the chelonian braincase. *Palaeontographica Abteilung A*:105–140.

Rollot Y, Lyson TR, Joyce WG. 2018. A description of the skull of *Eubaena cephalica* (Hay, 1904) and new insights into the cranial circulation and innervation of baenid turtles. *Journal of Vertebrate Paleontology* **38** (3):e1474886. DOI: 10.1080/02724634.2018.1474886

Sterli J, Joyce WG. 2007. The cranial anatomy of the Early Jurassic turtle *Kayentachelys aprix*. *Acta Palaeontologica Polonica* **52**:675–694. DOI: 10.1111/j.1463-6395.2009.00439.x

Sterli J, De La Fuente MS, Umazano AM. 2015. New remains and new insights on the Gondwanan meiolaniform turtle *Chubutemys copelloi* from the Lower Cretaceous of Patagonia, Argentina. *Gondwana Research* **27**:978–994. DOI: 10.1016/j.gr.2013.08.016

Sukhanov VB. 2000. Mesozoic turtles of middle and central Asia. *The age of dinosaurs in Russia and Mongolia* **17**:309–367.

Swofford DL. 2002. PAUP*. Phylogenetic Analysis Using Parsimony (*and other methods). Version. 4.0. Sinauer Associates, Sunderland, MA.

Tong H, Hirayama R, Makhoul E, Escuilliè F. 2006. *Rhinochelys* (Chelonioidea: Protoszegidae) from the Late Cretaceous (Cenomanian) of Nammoura, Lebanon. *Atti della Società italiana di scienze naturali e del museo civico di storia naturale di Milano* **147** (1):113–138.

Vitek NS, Danilov IG, Nakajima Y, Hirayama R. 2018. Redescription of the skull of '*Trionyx' kyrgyzensis* and improved phylogenetic taxon sampling of Cretaceous and Palaeogene soft-shelled turtles (Trionychidae) of Asia, including the oldest crown trionychids. *Journal of Systematic Palaeontology* **16**:199–211. DOI: 10.1080/14772019.2017.1283365

Völker H. 1913. Über das Stamm-, Gliedmaßen- und Hauptskelet von *Dermochelys coriacea* L. *Zoologische Jahrbücher, Abteilung für Anatomie und Ontogenie der Tiere* **33** (3):431–552.

Weems RE, Brown KM. 2017. More-complete remains of *Procolpochelys charlestonensis* (Oligocene, South Carolina), an occurrence of *Euclastes* (upper Eocene, South Carolina), and their bearing on Cenozoic pancheloniid sea turtle distribution and phylogeny. *Journal of Paleontology* **91**:1228–1243. DOI: 10.1017/jpa.2017.64

Weems RE, Sanders AE. 2014. Oligochene pancheloniid sea turtle from the vicinity of Charleston, South Carolina, U.S.A. *Journal of Vertebrate Paleontology* **34** (1):80–99. DOI: 10.1080/02724634.2013.792826

Whetstone KN. 1978. A new genus of cryptodiran turtles (Testudinoidea, Chelydridae) from the Upper Cretaceous Hell Creek Formation of Montana. *The University of Kansas Sciene Bulletin*, v. 51, 539–563. DOI: 10.5962/bhl.part.17248

Wieland GR. 1896. *Archelon ischyros*: A new gigantic Cryptodire Testudinate from the Fort Pierre Cretaceous of South Dakota. *American Journal of Science* **2**:399. DOI: 10.2475/ajs.s4-2.12.399

Wieland GR. 1902. Notes on the Cretaceous turtles, *Toxochelys* and *Archelon*, with a classification of the marine Testudinata. *American Journal of Science* **14**:95–108. DOI: 10.2475/ajs.s4-14.80.95

Wieland GR. 1906. The osteology of *Protostega*. *Memoirs of the Carnegie Museum* **II**:279–305. DOI: 10.5962/bhl.title.43187

Williston SW. 1902. On the hindlimb of *Protostega*. *American Journal of Science* **13** (76):276–276. DOI: 10.2475/ajs.s4-13.76.276

Wyneken J. 2001. The anatomy of sea turtles. *U.S. Department of Commerce NOAA Technical Memorandum NMFS-SEFC-470*:1–172.

Zangerl R. 1953*a*. The vertebrate fauna of the Selma Formation of Alabama. Part III. The turtles of the family Protostegidae. *Fieldiana. Geology Memoirs* **3** (3):63–133. DOI: 10.5962/bhl.title.5334

Zangerl R, Sloan RE. 1960. A new specimen of *Desmatochelys lowi* Williston, A primitive cheloniid sea turtle from the Cretaceous of South Dakota. *Fieldiana. Geology Memoirs* **14** (2):7–39. DOI: 10.5962/bhl.title.5178

Zangerl R. 1971. Two toxochelyid sea turtle from the Landenian sands of Erquelinnes (Hainaut), of Belgium. *Institute Royal des Sciences Naturelles de Belgique Mémoires* **169**:3–32. Available at: https://lib.ugent.be/catalog/rug01:001995819
