# Supplementary material for: Anatomy of Rhinochelys pulchriceps (Protostegidae) and marine adaptation during the early evolution of chelonioids
Source: PeerJ. 2019 May 1;7:e6811. doi: 10.7717/peerj.6811 (PMC6500378; doi:10.7717/peerj.6811)
Supplement: Supplemental Information 3 — This document is the full character list and also includes by-character optimisations. [file peerj-07-6811-s003.docx]

**APPENDIX S3**

**Content**

1. General notes on our character list

2. Character list with documentation of modified characters

3. Supplementary references

**1. General notes on our character list**

Our character list aims to capture all variation documented so far for non-pleurodiran turtles. This includes 32 characters that, with respect to our taxon sampling, are parsimony uninformative because they are only scored for one taxon differently from all other taxa or invariant. We keep these characters in our list, because other people might chose to test the phylogenetic positions of additional taxa using our matrix in future.

The sources for characters listed as ‘Evers & Benson (2018)’ in most instances, because we used the character list of that paper as a baseline for our modifications. However, most characters are ultimately from older analyses. The original sources are summarized in Evers & Benson (2018), and also in Cadena & Parham (2015).

Each character is followed by a section called “character optimization”, which lists the characters state changes of that character for each node in our phylogeny. “Unambiguous” synapomorphies are those found under both ACCTRAN and DELTRAN optimization. Because some nodes of the phylogeny represent unnamed clades, we use a code to denote the content of these clades. Hereby, the notation “Taxon+++” indicates the clade comprising the named taxon and all more crownwardly positioned taxa. The notation “(Taxon A ++ Taxon B)” indicates the most inclusive clade including Taxon A and Taxon B.

**2. Character list with documentation of modified characters**

**CRANIUM**

**Character 1 (Evers & Benson [2018]: character 1).** Nasals: 0 = present; 1 = absent.

*Character optimisation*: Unambiguous: *Calcarichelys gemma* (0->1), (*Protostega* + *Archelon*) (0->1); DELTRAN: Cryptodira (0->1), Protostegidae (1->0), *Toxochelys* sp. (1->0), *Chelus fimbriatus* (0->1), *Araripemys barretoi* (0->1), total-group Pelomedusoides (0->1); ACCTRAN: Testudines (0->1), total-group Chelonioidea (1->0), *Corsochelys*+++ (0->1), Angolachelonia (1->0), crown-group Chelidae (1->0), *Chelus fimbriatus* (0->1).

**Character 2 (Evers & Benson [2018]: character 2).** Nasal, medial contact of nasals: 0 = nasals contact one another medially along their entire length; 1 = medial contact of nasals partially or fully hindered by long an- terior frontal process.

*Character optimisation*: Unambiguous: (*Brachyopsemys* ++ *Sandownia*) (0->1), *Sinemys gamera* (0->1), (*Annemys* sp. IVPP V18106 ++ *Xinjiangchelys*) (0->1); DELTRAN: crown-group Chelidae (0->1), Pleurosternidae (0->1), *Arundelemys dardeni* (0->1); ACCTRAN: crown-group Pleurodira (0->1), Paracryptodira (0->1), *Eubaena cephalica* (1->0).

**Character 3 (Evers & Benson [2018]: character 3**).

*Character optimisation*: Unambiguous: Xinjiangchelyidae+++ (0->1), *Leyvachelys cipadi* (1->0), Pleurosternidae (0->1); DELTRAN: *Ocepechelon bouyai* (1->0); ACCTRAN: (*Ocepechelon* ++ *Protostega*) (1->0).

**Character 4 (Evers & Benson [2018]: character 4).** Prefrontals, medial contact of prefrontals on the dorsal skull surface: 0 = absent; 1 = present, absence of contact between the nasal or apertura narium externa and the frontal.

*Character optimisation*: Unambiguous: (*Chelosphargis* + *Rhinochelys*) (1->0), (*Desmatochelys* ++ *Protostega*) (1->0), (*Plesiochelys planiceps* ++ *Jurassichelon*) (1->0), crown-group Chelidae (1->0), *Chubutemys copelloi* (0->1); DELTRAN: Sinemydidae/Macroabenidae+++ (0->1), *Ordosemys* sp. IVPP V12092 (1->0), (*Sinemys gamera* + *Sinemys lens*) (1->0), *Annemys* *levensis* (0->1); ACCTRAN: Xinjiangchelyidae+++ (0->1), Sinemydidae (1->0), *Dracochelys bicuspis* (0->1), (*Annemys* *latiens* ++ *Xinjiangchelys*) (1->0).

**Character 5 (Evers & Benson [2018]: character 5).** Prefrontal, prefrontal-vomer contact: 0 = present; 1 = absent.

*Character optimisation*: Unambiguous: (*Kayentachelys*+*Eileanchelys*)+++ (1->0), crown-group Pleurodira (0->1).

**Character 6 (Evers & Benson [2018]: character 6**). Prefrontal, prefrontal-palatine contact: 0 = present; 1 = absent.

*Character optimisation*: Unambiguous: crown-group Cheloniidae (0->1), *Ctenochelys* (0->1), crown-group Trionychia (0->1), crown-group Pleurodira (0->1), *Chelus fimbriatus* (1->0); DELTRAN: *Dermochelys coriacea* (0->1); ACCTRAN: Dermochelyidae (0->1).

**Character 7 (Evers & Benson [2018]: character 7).** Prefrontal, dorsal prefrontal exposure: 0 = present, large; 1 = reduced; 2 = absent or near absent.

*Character optimisation*: Unambiguous: (*Jurassichelon* + *Portlandemys*) (0->1), *Sandownia* *harrisi* (0->1), *Leyvachelys cipadi* (0->1), total-group Chelidae (0->1), (*Sinemys gamera* + *Sinemys lens*) (0->1), (*Annemys* *latiens* ++ *Xinjiangchelys*) (0->1), Paracryptodira (0->1), *Eubaena cephalica* (1->2), (*Kayentachelys* + *Eileanchelys*) (0->1); DELTRAN: *Ocepechelon bouyai* (0->1), (*Desmatochelys* *lowii* + *Desmatochelys* *padillai*) (0->1); ACCTRAN: (*Desmatochelys* ++ *Protostega*) (0->1), (*Protostega* + *Archelon*) (1->0).

**Character 8 (Evers & Benson [2018]: character 8).** Prefrontal, cranial scutes on the prefrontal: 0 = one pair; 1= two pairs or more.

*Character optimisation*: Unambiguous: (*Eretmochelys* ++ *Lepidochelys*) (0->1).

**Character 9 (Evers & Benson [2018]: character 9).** Prefrontal, sculpturing: 0 = heavily sculptured; 1 = sculpturing absent.

*Character optimisation*: Unambiguous: (*Kayentachelys*+*Eileanchelys*)+++ (0->1), *Bouliachelys suteri* (1->0), *Chelydra serpentina* (1->0).

**Character 10 (new character)**: Prefrontal, preorbital bulge formed between the prefrontal and premaxilla: 0 = absent; 1 = present.

*Character optimisation*: Unambiguous: (*Rhinochelys* *pulchriceps* + *Rhinochelys* *nammourensis*) (0->1).

**Character 11 (Evers & Benson [2018]: character 10).** Lacrimal: 0 = present; 1 = absent. JY1 & Sterli & de la Fuente (2013: ch 9, Lacrimal A).

*Character optimisation*: Unambiguous: (*Kayentachelys*+*Eileanchelys*)+++ (0->1).

**Character 12 (Evers & Benson [2018]: character 11).** Frontal, frontal contribution to orbit: 0 = absent, contact between prefrontal and postorbital; 1 = present.

*Character optimisation*: Unambiguous: *Bouliachelys suteri* (1->0), (*Protostega* + *Archelon*) (1->0), Dermochelyidae (1->0), crown-group Chelydridae (1->0), Kinosternidae (1->0), *Eubaena cephalica* (1->0); DELTRAN: Paracryptodira+++ (0->1), *Lepidochelys* *olivacea* (1->0), *Caretta Caretta* (1->0), *Emys orbicularis* (1->0), *Platysternon megacephalum* (1->0), *Geoclemys hamiltonii* (1->0), *Kallokibotion bajazidi* (0->1), (*Kayentachelys* + *Eileanchelys*) (0->1); ACCTRAN: *Australochelys*+++ (0->1), (*Caretta* ++ *Lepidochelys*) (1->0), *Lepidochelys* *kempii* (0->1), Testudinoidea (1->0), *Chrysemys picta* (0->1), Testudinidae (0->1), *Meiolania planiceps* (1->0), *Chubutemys copelloi* (1->0).

**Character 13 (Evers & Benson [2018]: character 12)**. Frontals, both frontals medially fused: 0 = absent; 1 = present.

*Character optimisation*: Unambiguous: *Chelodina* (0->1).

**Character 14 (Evers & Benson [2018]: character 13).** Frontal, direction of the orbits in dorsal view of the skull: 0 = laterally facing, with a very narrow to almost complete absent dorsal exposure of the maxilla and jugal; 1 = dorsolateral facing, with portions of the maxilla and jugal dorsally exposed.

*Character optimisation*: Unambiguous: Xinjiangchelyidae+++ (0->1), Cryptodira (1->0), *Ocepechelon bouyai* (0->1), *Corsochelys* *halinches* (0->1), *Toxochelys* sp. (0->1), *Chelonoidis* sp. (0->1), Trionychidae (0->1), *Kirgizemys dmitrievi* (1->0); DELTRAN: (*Ctenochelys* + *Cabindachelys*) (0->1), *Erquelinnesia gosseleti* (0->1); ACCTRAN: (*Ctenochelys* ++ *Peritresius*) (0->1), (*Oligochelone* + *Erquelinnesia*) (0->1).

**Character 15 (Evers & Benson [2018]: character 14).** Frontals, development of crista cranii: 0 = crista cranii on ventral surface of frontals very shallow, sulcus olfactorius developed is a low trough; 1 = crista cranii developed as moderately deep parasagittal ridges on the ventral surface of each frontal, forming a ventrally open, median trough (the sulcus olfactorius) that extends from the anterior margin of the cavum cranii posteriorly to the fissura ethmoidalis anteriorly; 2 = crista cranii very deep anteriorly, forming extended processes that meet along the midline of the cranium and are sutured to one another, forming an ossified olfactory canal.

*Character optimisation*: Unambiguous: (*Santanachelys* ++ *Rhinochelys*) (1->2); DELTRAN: *Dermochelys coriacea* (1->0); ACCTRAN: Dermochelyidae (1->0).

**Character 16 (Evers & Benson [2018]: character 15).** Parietal, parietal-squamosal contact: 0 = present, upper temporal emargination absent or poorly developed; 1 = absent, upper temporal emargination well developed.

*Character optimisation*: Unambiguous: (*Ocepechelon* ++ *Protostega*) (1->0), *Corsochelys*+++ (1->0), *Eubaena cephalica* (0->1); DELTRAN: Cryptodira (0->1), *Ctenochelys* (0->1), *Chelodina* (0->1), *Araripemys barretoi* (0->1), total-group Pelomedusoides (0->1), (*Sinemys gamera* + *Sinemys lens*) (0->1); ACCTRAN: Sinemydidae/Macroabenidae+++ (0->1), (*Ctenochelys* ++ *Peritresius*) (0->1), Angolachelonia (1->0), crown-group Chelidae (1->0), *Chelodina* (0->1), (*Kirgizemys* *hoburensis* + *Kirgizemys dmitrievi*) (1->0).

**Character 17 (Evers & Benson [2018]: character 16).** Parietal, posterodorsal margin of the temporal fossa roofed by an overhanging process of the skull roof: 0 = absent; 1 = present.

*Character optimisation*: Unambiguous: Paracryptodira+++ (1->0), *Rhinochelys* *nammourensis* (1->0), Dermochelyidae (0->1), (*Erquelinnesia* ++ *Lepidochelys*) (0->1), *Ctenochelys* (1->0), *Platysternon megacephalum* (0->1), Sandownidae (0->1), *Sandownia* *harrisi* (1->0), *Elseya dentata* (0->1); DELTRAN: (*Chelosphargis* + *Rhinochelys*) (0->1); ACCTRAN: (*Bouliachelys* + *Rhinochelys*) (0->1).

**Character 18 (Evers & Benson [2018]: character 17).** Parietal, contribution to the processus trochlearis oticum: 0 = absent; 1 = present.

*Character optimisation*: Unambiguous: Kinosternidae (0->1), total-group Trionychia (0->1), *Solnhofia parsoni* (0->1); DELTRAN: *Geoclemys hamiltonii* (0->1), (*Testudo* + *Chelonoidis*) (0->1), *Pleurosternon bullockii* (0->1); ACCTRAN: Testuguria (0->1), *Gopherus polyphemus* (1->0), Pleurosternidae (0->1).

**Character 19 (Evers & Benson [2018]: character 18)**. Parietals, foramen stapedio-temporalis: 0 = absent or weak, foramen stapedio- temporale concealed in dorsal view; 1 = moderate foramen stapedio-temporale, partial exposition of the processes trochlearis in dorsal view; 2 = strong, entire exposition of the processus trochlearis in dorsal view.

*Character optimisation*: Unambiguous: Paracryptodira+++ (0->2), *Desmatochelys* *padillai* (0->1), *Nichollsemys baieri* (0->1), *Platysternon megacephalum* (2->0), Angolachelonia (2->0), *Jurassichelon* *oleronensis* (0->1), *Elseya dentata* (2->0), *Podocnemis* (2->0), (*Judithemys* ++ *Sinemys*) (2->1), (*Sinemys gamera* + *Sinemys lens*) (1->2), Pleurosternidae (2->0); DELTRAN: Protostegidae+++ (2->0), *Toxochelys* sp. (2->1); ACCTRAN: total-group Chelonioidea (2->0), *Toxochelys* sp. (0->1).

**Character 20 (Evers & Benson [2018]: character 19).** Parietal, pineal foramen located medially between parietals: 0 = absent; 1 = present.

*Character optimisation*: Unambiguous: (*Desmatochelys* *lowii* + *Desmatochelys* *padillai*) (0->1).

**Character 21 (Evers & Benson [2018]: character 20).** Parietal, processus inferior parietalis: 0 = weak or absent, parietal does not contact the pterygoid, epipterygoid, and/or palatine; 1 = present and well developed, the parietal contacts the pterygoid, epipterygoid, and/or palatine.

*Character optimisation*: DELTRAN: *Chubutemys*+++ (0->1), *Eileanchelys* *waldmanni* (0->1); ACCTRAN: (*Kayentachelys*+*Eileanchelys*)+++ (0->1), *Kayentachelys* *aprix* (1->0).

**Character 22 (Evers & Benson [2018]: character 21).** Parietal, closure of foramen nervi trigemini and the length of the anterior extension of the lateral braincase wall: 0 = foramen nervi trigemini anteriorly open, anterior extension of lateral braincase wall absent; 1 = foramen nervi trigemini anteriorly closed, processus inferior parietalis only produces a narrow strut anterior to the foramen nervi trigemini, usually absence of contact with palatine; 2 = foramen nerivi trigemini anteriorly closed, processus inferior parietalis produces an ex- tended process anterior to the foramen nervi trigemini, contact with palatine commonly present.

*Character optimisation*: Unambiguous: *Chubutemys*+++ (0->1), *Cabindachelys* *landanensis* (1->2), Kinosternoidea (1->2), *Gopherus polyphemus* (1->2), total-group Trionychia (1->2), total-group Pelomedusoides (1->2); DELTRAN: *Dermochelys coriacea* (1->0), (*Sandownia* + *Angolachelys*) (1->2), *Leyvachelys cipadi* (1->2); ACCTRAN: Dermochelyidae (1->0), Sandownidae (1->2), *Brachyopsemys* *tingitana* (2->1).

**Character 23 (Evers & Benson [2018]: character 22).** Parietal, posterior ramus of processus inferior parietalis forming the posterior margin of the trigeminal foramen: 0 = absent; 1 = present.

*Character optimisation*: Unambiguous: (*Bouliachelys* + *Rhinochelys*) (1->0), crown-group Chelonioidea (1->0), Kinosternoidea (1->0), *Chrysemys picta* (1->0), *Gopherus polyphemus* (1->0), total-group Trionychia (1->0), *Chelus fimbriatus* (1->0), *Pelomedusa subrufa* (1->0); DELTRAN: Xinjiangchelyidae+++ (0->1), *Pleurosternon bullockii* (0->1); ACCTRAN: Paracryptodira+++ (0->1), *Arundelemys dardeni* (1->0).

**Character 24 (Evers & Benson [2018]: character 23).** Posterior ramus of processus inferior parietalis of the parietal: 0 = short; 1 = long, excludes the prootic from the trigeminal foramen. This character is scored inapplicable for taxa that lack the process altogether.

*Character optimisation*: Unambiguous: *Elseya dentata* (0->1); DELTRAN: *Emys orbicularis* (0->1), Testuguria (0->1), Angolachelonia (0->1), *Podocnemis* (0->1), *Kirgizemys* *hoburensis* (0->1); ACCTRAN: Sinemydidae/Macroabenidae+++ (0->1), Americhelydia (1->0), *Platysternon megacephalum* (1->0), crown-group Pleurodira (1->0), crown-group Pelomedusoides (0->1).

**Character 25 (Evers & Benson [2018]: character 24).** Parietal, ridge on lateral surface of processus inferior parietalis: 0 = absent; 1 = present, a ridge between the ventral surface of the parietal and the lateral surface of the descending process marks the border between the temporal and orbital fossae.

*Character optimisation*: Unambiguous: (*Puppigerus* ++ *Ctenochelys*) (0->1), *Chelydra serpentina* (0->1), Kinosterninae (0->1), *Phrynops geoffroanus* (0->1), *Elseya dentata* (0->1); DELTRAN: crown-group Trionychia (0->1), *Podocnemis* (0->1), *Galianemys whitei* (0->1), *Sinemys gamera* (0->1), Paracryptodira (0->1), *Kayentachelys* *aprix* (0->1), *Australochelys* *africanus* (0->1); ACCTRAN: *Australochelys*+++ (0->1), *Chubutemys*+++ (1->0), total-group Trionychia (0->1), total-group Pelomedusoides (0->1), *Pelomedusa subrufa* (1->0), (*Sinemys gamera* + *Sinemys lens*) (0->1), Paracryptodira (0->1).

**Character 26 (Evers & Benson [2018]: character 25).** Jugal, jugal-squamosal contact: 0 = present; 1 = absent.

*Character optimisation*: Unambiguous: *Dermochelys coriacea* (1->0), *Allopleuron hoffmanni* (1->0), *Meiolania planiceps* (1->0); DELTRAN: (*Kayentachelys*+*Eileanchelys*)+++ (0->1); ACCTRAN: *Australochelys*+++ (0->1).

**Character 27 (Evers & Benson [2018]: character 26).** Jugal, jugal participation in the margin of the upper temporal emargination: 0 = absent; 1 = present, upper temporal emargination extensive.

*Character optimisation*: Unambiguous: Kinosternidae (0->1), *Geoclemys hamiltonii* (0->1), Trionychidae (0->1).

**Character 28 (Evers & Benson [2018]: character 27).** Jugal, medial process of jugal ventral to orbit: 0 = weakly developed or absent, jugal contacts only the maxilla; 1 = present and well developed, jugal contacts the maxilla as well as the palatine and/or pterygoid.

*Character optimisation*: Unambiguous: Testudinidae (1->0), Plesiochelyidae (1->0), *Leyvachelys cipadi* (1->0), *Chubutemys copelloi* (1->0); DELTRAN: Protostegidae (1->0), Dermochelyidae (1->0); ACCTRAN: Protostegidae+++ (1->0), total-group Cheloniidae (0->1).

**Character 29 (Evers & Benson [2018]: character 28).** Jugal, contact with the palatine: 0 = absent; 1 = present. This character is scored as inapplicable when the jugal lacks a medial process (ch 27.0).

*Character optimisation*: Unambiguous: *Chelydra serpentina* (1->0), *Emys orbicularis* (1->0), *Angolachelys* *mbaxi* (0->1), (*Kayentachelys* + *Eileanchelys*) (0->1); DELTRAN: Cryptodira (0->1), *Nichollsemys baieri* (1->0), *Toxochelys* sp. (1->0), crown-group Pleurodira (0->1), (*Eubaena* + Pleurosternidae) (0->1); ACCTRAN: Testudines (0->1), total-group Chelonioidea (1->0), *Allopleuron*+++ (0->1), Angolachelonia (1->0), Paracryptodira (0->1).

**Character 30 (Evers & Benson [2018]: character 29)**. Jugal, contact with the pterygoid: 0 = absent; 1 = present. This character is scored as inapplicable when the jugal lacks a medial process (ch 27.0).

*Character optimisation*: Unambiguous: (*Eretmochelys* ++ *Lepidochelys*) (1->0), *Lepidochelys* *kempii* (0->1), *Emarginachelys cretacea* (1->0), *Lissemys punctata* (1->0), *Chubutemys copelloi* (1->0); DELTRAN: *Procolpochelys charlestonensis* (1->0); ACCTRAN: (*Procolpochelys* ++ *Erquelinnesia*) (1->0).

**Character 31 (Evers & Benson [2018]: character 30).** Jugal, jugal-parietal contact: 0 = absent; 1 = present.

*Character optimisation*: Unambiguous: Trionychidae (0->1), *Podocnemis* (0->1).

**Character 32 (Evers & Benson [2018]: character 31).** Quadratojugal: 0 = present; 1 = absent.

*Character optimisation*: Unambiguous: crown-group Chelidae (0->1).

**Character 33 (Evers & Benson [2018]: character 32**). Quadratojugal, quadratojugal-maxilla contact: 0 = absent; 1 = present, jugal does not contribute to lower temporal emargination.

*Character optimisation*: Unambiguous: Kinosternidae (0->1), *Platysternon megacephalum* (0->1), *Galianemys whitei* (0->1); DELTRAN: *Carettochelys insculpta* (0->1); ACCTRAN: total-group Carettochelyidae (0->1).

**Character 34 (Evers & Benson [2018]: character 33).** Quadratojugal, quadratojugal-squamosal contact below the cavum tympani: 0 = absent; 1 = present.

*Character optimisation*: Unambiguous: *Meiolania planiceps* (0->1).

**Character 35 (Evers & Benson [2018]: character 34).** Quadratojugal, lower temporal emargination: 0 = weak to no emargination, the margin of the lower temporal emargination is formed by the quadratojugal or quadratojugal and jugal; 1 = moderate emargination, the margin of the lower temporal emargination is principally formed by the quadratojugal and jugal, but the maxilla is included in the anterior section of the margin and/or the quadrate is included in the posterior section of the margin; 2 = large emargination, the postorbital and/or squamosal and parietal are included in the margin of the lower temporal emargination.

*Character optimisation*: Unambiguous: Paracryptodira+++ (0->1), *Nichollsemys baieri* (0->1), total-group Dermatemydidae (0->1), *Plesiochelys planiceps* (1->2), Sandownidae (1->0), crown-group Chelidae (1->2), *Galianemys whitei* (1->0); DELTRAN: Americhelydia (1->0), *Platysternon megacephalum* (1->0), *Geoclemys hamiltonii* (1->0), crown-group Trionychia (1->0), (*Sinemys gamera* + *Sinemys lens*) (1->2), (*Kirgizemys* *hoburensis* + *Kirgizemys dmitrievi*) (1->2), *Annemys* *levensis* (1->2), *Annemys* sp. IVPP V18106 (1->2); ACCTRAN: Cryptodira (1->0), Emydidae (0->1), Testudinidae (0->1), *Adocus beatus* (0->1), (*Sinemys* ++ *Kirgizemys*) (1->2), (*Ordosemys* + *Dracochelys*) (2->1), Xinjiangchelyidae (1->2), (*Xinjiangchelys* *wusu* + *Xinjiangchelys* *radiplicatoides*) (2->1).

**Character 36 (Evers & Benson [2018]: character 35).** Squamosal, squamosal-postorbital contact: 0 = present; 1 = absent.

*Character optimisation*: Unambiguous: total-group Chelonioidea (1->0), crown-group Chelydridae (1->0), *Platysternon megacephalum* (1->0), *Pleurosternon bullockii* (0->1); DELTRAN: Cryptodira (0->1), crown-group Pleurodira (0->1), *Sinemys gamera* (0->1); ACCTRAN: Sinemydidae/Macroabenidae+++ (0->1), Angolachelonia (1->0), (*Kirgizemys* *hoburensis* + *Kirgizemys dmitrievi*) (1->0).

**Character 37 (Evers & Benson [2018]: character 36)**. Squamosal, squamosal-supraoccipital contact: 0 = absent; 1 = present.

*Character optimisation*: Unambiguous: *Meiolania planiceps* (0->1).

**Character 38 (Evers & Benson [2018]: character 37).** Squamosal, posterolateral protuberances developing horns: 0 = absent; 1 = present.

*Character optimisation*: Unambiguous: *Meiolania planiceps* (0->1).

**Character 39 (Evers & Benson [2018]: character 38).** Squamosal, very long posterior process, formed exclusively by the squamosal and protruding beyond condyles occipitalis: 0 = absent; 1 = present.

*Character optimisation*: Unambiguous: *Ocepechelon bouyai* (0->1), *Toxochelys* sp. (0->1), *Staurotypus* (0->1), *Chrysemys picta* (0->1), *Araripemys barretoi* (0->1), *Pelomedusa subrufa* (0->1); DELTRAN: total-group Trionychia (0->1), *Angolachelys* *mbaxi* (0->1), (*Judithemys* ++ *Sinemys*) (0->1), *Sinemys lens* (1->0), Xinjiangchelyidae (0->1); ACCTRAN: Xinjiangchelyidae+++ (0->1), Testudines (1->0), total-group Trionychia (0->1), (*Sandownia* + *Angolachelys*) (0->1), (*Sinemys gamera* + *Sinemys lens*) (1->0).

**Character 40 (Evers & Benson [2018]: character 39).** Squamosal, squamosal-quadrate contact: 0 = tightly sutured; 1 = wide open.

*Character optimisation*: Unambiguous: total-group Chelonioidea (0->1), Plesiochelyidae (0->1).

**Character 41 (Evers & Benson [2018]: character 40).** Squamosal, posterodorsal margin of cavum tympanum: 0 = the squamosal forms the posterodorsal margin of the cavum tympanum; 1 = the squamosal is excluded from the posterodorsal margin of the cavum tympanum.

*Character optimisation*: Unambiguous: *Desmatochelys* *lowii* (1->0), *Corsochelys*+++ (1->0), *Baptemys wyomingensis* (1->0), *Platysternon megacephalum* (1->0), *Chelonoidis* sp. (1->0), Trionychidae (1->0), Thalassochelydia (1->0), *Kirgizemys* *hoburensis* (0->1); DELTRAN: Testudines (0->1), *Archelon* *ischyros* (1->0), *Macrochelys temminckii* (1->0), *Emarginachelys cretacea* (1->0), *Annemys* sp. IVPP V18106 (0->1); ACCTRAN: Xinjiangchelyidae+++ (0->1), (*Protostega* + *Archelon*) (1->0), total-group Chelydridae (1->0), *Chelydra serpentina* (0->1), (*Judithemys* ++ *Sinemys*) (1->0).

**Character 42 (Evers & Benson [2018]: character 41).** Postorbital, postorbital-palatine contact: 0 = absent; 1 = present, foramen palatinum posterius situated posterior to the orbital wall.

*Character optimisation*: Unambiguous: crown-group Pleurodira (0->1).

**Character 43 (Evers & Benson [2018]: character 42).** Postorbital, contact with the quadratojugal: 0 = present; 1 = absent.

*Character optimisation*: Unambiguous: *Dermochelys coriacea* (0->1), *Allopleuron hoffmanni* (0->1), *Geoclemys hamiltonii* (0->1), *Testudo* (0->1), Trionychidae (0->1), *Podocnemis* (0->1); DELTRAN: *Kinosternon suburum hippocrepisis* (0->1), *Staurotypus* (0->1), *Proganochelys quenstedti* (0->1); ACCTRAN: Kinosternidae (0->1), *Sternotherus* (1->0), *Proganochelys quenstedti* (0->1).

**Character 44 (Evers & Benson [2018]: character 43).** Postorbital, postorbital-maxilla contact preventing the jugal from entering the orbital margin: 0 = absent; 1 = present.

*Character optimisation*: Unambiguous: *Platysternon megacephalum* (0->1), *Galianemys whitei* (0->1), Paracryptodira (0->1).

**Character 45 (Evers & Benson [2018]: character 44).** Postorbital, dorsal margin of orbit: 0 = continuously and concavely curved margin between frontals and jugal; 1 = frontal margin relatively narrow, with lateral bulge of postorbital.

*Character optimisation*: Unambiguous: *Archelon* *ischyros* (0->1), (*Erquelinnesia* ++ *Lepidochelys*) (0->1); DELTRAN: (*Ctenochelys* + *Cabindachelys*) (1->0), *Plesiochelys etalloni* (0->1); ACCTRAN: (*Ctenochelys* ++ *Peritresius*) (1->0), (*Plesiochelys etalloni* + *Plesiochelys* *bigleri*) (0->1).

**Character 46 (Evers & Benson [2018]: character 45**). Supratemporal: 0 = present; 1 = absent.

*Character optimisation*: DELTRAN: *Chubutemys*+++ (0->1); ACCTRAN: *Australochelys*+++ (0->1).

**Character 47 (Evers & Benson [2018]: character 46).** Teeth in premaxilla, maxilla, and dentary: 0 = present; 1 = absent.

*Character optimisation*: -.

**Character 48 (Evers & Benson [2018]: character 47).** Premaxilla, subdivision of the apertura narium externa by an internarial process of the premaxilla: 0 = present; 1 = absent.

*Character optimisation*: Unambiguous: Paracryptodira+++ (0->1), *Angolachelys* *mbaxi* (1->0), (*Kayentachelys* + *Eileanchelys*) (0->1).

**Character 49 (Evers & Benson [2018]: character 48).** Premaxilla, fusion of premaxillae: 0 = absent; 1 = present.

*Character optimisation*: Unambiguous: *Ocepechelon bouyai* (0->1), crown-group Trionychia (0->1), *Chelus fimbriatus* (0->1); DELTRAN: *Erquelinnesia gosseleti* (0->1), *Angolachelys* *mbaxi* (0->1), *Brachyopsemys* *tingitana* (0->1); ACCTRAN: (*Oligochelone* + *Erquelinnesia*) (0->1), (*Brachyopsemys* ++ *Sandownia*) (0->1), *Sandownia* *harrisi* (1->0).

**Character 50 (Evers & Benson [2018]: character 49).** Premaxilla, foramen praepalatinum: 0 = absent; 1 = present.

*Character optimisation*: Unambiguous: (*Erquelinnesia* ++ *Lepidochelys*) (1->0), *Staurotypus* (1->0), crown-group Trionychia (1->0), *Solnhofia parsoni* (1->0), (*Brachyopsemys* ++ *Sandownia*) (1->0); DELTRAN: (*Kayentachelys*+*Eileanchelys*)+++ (0->1), (*Bouliachelys* + *Rhinochelys*) (1->0), *Ocepechelon bouyai* (1->0); ACCTRAN: *Australochelys*+++ (0->1), Protostegidae (1->0), (*Desmatochelys* *lowii* + *Desmatochelys* *padillai*) (0->1).

**Character 51 (Evers & Benson [2018]: character 50).** Premaxilla, foramen intermaxillaris: 0 = absent; 1 = present.

*Character optimisation*: Unambiguous: Trionychidae (0->1).

**Character 52 (Evers & Benson [2018]: character 51).** Premaxilla, exclusion of the premaxillae from the apertura narium externa: 0 = absent; 1 = present.

*Character optimisation*: Unambiguous: Trionychidae (0->1).

**Character 53 (Evers & Benson [2018]: character 52).** Premaxilla, distinct, median premaxillary hook along the labial margin of the premaxillae: 0 = absent; 1 = present.

*Character optimisation*: Unambiguous: *Kinosternon suburum hippocrepis* (0->1), (*Protostega* + *Archelon*) (0->1), *Lepidochelys* *kempii* (0->1), crown-group Chelydridae (0->1), *Platysternon megacephalum* (0->1).

**Character 54 (Evers & Benson [2018]: character 53)** Premaxilla, cusps developed on the labial ridge in conjunction with maxilla: 0 = absent; 1 = present.

*Character optimisation*: Unambiguous: Dermochelyidae (0->1), *Dracochelys bicuspis* (0->1).

**Character 55 (Evers & Benson [2018]: character 54).** Palatine, contribution to the anterior extension of the lateral braincase wall: 0 = absent; 1 = present.

*Character optimisation*: Unambiguous: Kinosternoidea (0->1), *Gopherus polyphemus* (0->1), total-group Trionychia (0->1), total-group Pelomedusoides (0->1); DELTRAN: *Sandownia* *harrisi* (0->1); ACCTRAN: (*Sandownia* + *Angolachelys*) (0->1).

**Character 56 (Evers & Benson [2018]: character 55).** Palatine, contribution to the upper triturating surface: 0 = absent or less than 30% of the total width of the triturating surface; 1 = present, at least 30% or more of the total width of the triturating surface.

*Character optimisation*: Unambiguous: *Allopleuron*+++ (0->1), *Eochelone brabantica* (1->0), (*Sandownia* + *Angolachelys*) (0->1), *Podocnemis* (0->1).

**Character 57 (Evers & Benson [2018]: character 56**). Palatine, secondary palate: 0 = absent; 1 = present, complete separation of the narial cavity from the oral cavity.

*Character optimisation*: Unambiguous: *Allopleuron*+++ (0->1), *Eochelone brabantica* (1->0); DELTRAN: *Solnhofia parsoni* (0->1), Sandownidae (0->1); ACCTRAN: Angolachelonia (0->1), Plesiochelyidae (1->0).

**Character 58 (Evers & Benson [2018]: character 57).** Palatine, vomer-palatine contact anterior to internal naris (apertura narium interna): 0 = absent; 1 = present.

*Character optimisation*: Unambiguous: *Eochelone brabantica* (1->0), Sandownidae (0->1); DELTRAN: crown-group Chelonioidea (0->1), *Toxochelys* sp. (0->1); ACCTRAN: total-group Chelonioidea (0->1), Protostegidae (1->0).

**Character 59 (Evers & Benson [2018]: character 58).** Maxilla, triturating surface definition: 0 = triturating surface with labial ridge only; 1 = triturating surface with labial and lingual ridge; 2 = triturating surface with labial, lingual, and accessory ridge(s).

*Character optimisation*: Unambiguous: *Ocepechelon bouyai* (1->0), *Ctenochelys* (0->1), *Argillochelys cuneiceps* (0->1), *Chelydra serpentina* (0->1), *Emarginachelys cretacea* (0->2), total-group Dermatemydidae (0->2), *Chrysemys picta* (0->2), *Platysternon megacephalum* (0->1), Testudinidae (0->2), *Adocus beatus* (0->2), Plesiochelyidae (0->1), *Elseya dentata* (0->2), crown-group Pelomedusoides (0->2), *Dracochelys bicuspis* (0->1), *Kirgizemys dmitrievi* (0->1), *Pleurosternon bullockii* (1->0), *Meiolania planiceps* (0->2); DELTRAN: Xinjiangchelyidae+++ (1->0), Protostegidae (0->1), *Eosphargis breineri* (0->1), *Lepidochelys* *kempii* (0->2), *Eretmochelys* *imbricata* (0->2), (*Chelonia* + *Natator*) (0->2), *Toxochelys* sp. (0->1), *Apalone spinifera* (0->1), *Carettochelys insculpta* (0->1), *Chubutemys copelloi* (1->0); ACCTRAN: *Chubutemys*+++ (1->0), total-group Chelonioidea (0->1), *Corsochelys*+++ (1->0), *Eosphargis breineri* (0->1), crown-group Cheloniidae (0->2), (*Caretta* ++ *Lepidochelys*) (2->0), *Lepidochelys* *kempii* (0->2), (*Apalone* + *Petrochelys*) (0->1), total-group Carettochelyidae (0->1), Paracryptodira (0->1).

**Character 60 (Evers & Benson [2018]: character 59).** Maxilla, accessory ridge(s): 0 = accessory ridge(s) on maxilla present along the triturating surface; 1 = accessory ridge(s) only in some sectors of the triturating surface. Thic character is scored as inapplicable when no accessory ridges are present (ch 58.0 or ch 58.1).

*Character optimisation*: DELTRAN: *Emarginachelys cretacea* (0->1), *Baptemys wyomingensis* (0->1), *Adocus beatus* (0->1); ACCTRAN: Chelydroidea (0->1), *Dermatemys mawii* (1->0), total-group Trionychia (0->1).

**Character 61 (Evers & Benson [2018]: character 60).** Maxilla, median contact between right and left maxilla on the palate: 0 = absent; 1 = present.

*Character optimisation*: Unambiguous: *Caretta Caretta* (0->1), Trionychidae (0->1), *Solnhofia parsoni* (0->1), *Elseya dentata* (0->1), *Kallokibotion bajazidi* (0->1).

**Character 62 (Evers & Benson [2018]: character 61).** Vomer, number of vomer(s): 0 = paired; 1 = single, but large; 2 = single and greatly reduced or absent.

*Character optimisation*: Unambiguous: crown-group Pelomedusoides (1->2), *Kirgizemys dmitrievi* (1->0); DELTRAN: (*Kayentachelys*+*Eileanchelys*)+++ (0->1); ACCTRAN: *Australochelys*+++ (0->1).

**Character 63 (Evers & Benson [2018]: character 62).** Vomer, vomer-pterygoid contact in palatal view: 0 = present; 1 = absent, medial contact of palatines present.

*Character optimisation*: Unambiguous: Protostegidae (0->1), *Nichollsemys baieri* (0->1), crown-group Trionychia (0->1), total-group Pleurodira (0->1), *Portlandemys mcdowelli* (1->0), (*Plesiochelys etalloni* + *Plesiochelys* *bigleri*) (1->0), (*Brachyopsemys* ++ *Sandownia*) (1->0), crown-group Chelidae (1->0), *Chubutemys copelloi* (0->1); DELTRAN: *Xinjiangchelys* *radiplicatoides* (0->1); ACCTRAN: (*Xinjiangchelys* *wusu* + *Xinjiangchelys* *radiplicatoides*) (0->1).

**Character 64 (Evers & Benson [2018]: character 63).** Vomer, vomerine and palatine teeth: 0 = present; 1 = absent.

*Character optimisation*: DELTRAN: *Australochelys*+++ (0->1); ACCTRAN: *Australochelys*+++ (0->1).

**Character 65 (Evers & Benson [2018]: character 64).** Vomer, vomer-premaxilla contact in ventral view: 0 = present; 1 = absent.

*Character optimisation*: Unambiguous: *Caretta Caretta* (0->1), *Dermatemys mawii* (0->1), crown-group Trionychia (0->1), *Solnhofia parsoni* (0->1), *Brachyopsemys* *tingitana* (0->1).

**Character 66 (Evers & Benson [2018]: character 65).** Vomer, ventral median crest: 0 = absent, ventral surface of vomer is smooth; 1 = present, shallow ridge extends along the ventral surface posterior to ventral process of the vomer, ridge becomes shallower posteriorly; 2 = narrow and tall ventral crest present all along the vomer.

*Character optimisation*: Unambiguous: *Chubutemys*+++ (0->2), Americhelydia (2->1), (*Protostega* + *Archelon*) (1->0), total-group Cheloniidae (1->0), *Eochelone brabantica* (0->1), *Ctenochelys* (0->1), *Toxochelys* sp. (1->0), *Macrochelys temminckii* (1->0), *Emys orbicularis* (2->0), *Platysternon megacephalum* (2->1), total-group Pleurodira (2->0), *Dracochelys bicuspis* (2->0), *Kirgizemys dmitrievi* (2->1); DELTRAN: *Carettochelys insculpta* (2->0), Pleurosternidae (2->0), *Arundelemys dardeni* (2->0); ACCTRAN: total-group Carettochelyidae (2->0), Paracryptodira (2->0), *Eubaena cephalica* (0->2).

**Character 67 (Evers & Benson [2018]: character 66).** Vomer, shape of the palate roof: 0 = flat; 1 = domed.

*Character optimisation*: Unambiguous: (*Kayentachelys*+*Eileanchelys*)+++ (1->0), *Ctenochelys* (0->1), Testuguria (0->1), *Testudo* (1->0), *Meiolania planiceps* (0->1); DELTRAN: *Carettochelys insculpta* (0->1); ACCTRAN: total-group Carettochelyidae (0->1).

**Character 68 (Evers & Benson [2018]: character 67).** Vomer, shape of anterior end contacting the maxillae and praemaxillae: 0 = flat, near horizontal contact with maxillae; 1 = the anterior end of the vomer is anteroventrally directed and laterally expanded; 2 = the anterior end of the vomer is ventrally expanded to form a horizontal footplate with a flat ventral surface.

*Character optimisation*: Unambiguous: Americhelydia (0->1), *Notochelone* (1->0), (*Ocepechelon* ++ *Protostega*) (1->0), *Dermochelys coriacea* (1->0), *Allopleuron*+++ (1->2), *Eochelone brabantica* (2->1), *Ctenochelys* (2->1), crown-group Chelydridae (1->0), *Geoclemys hamiltonii* (0->1); DELTRAN: *Sternotherus* (1->2), *Staurotypus* (1->2), *Solnhofia parsoni* (0->2), (*Brachyopsemys* ++ *Sandownia*) (0->2); ACCTRAN: Kinosternidae (1->2), *Kinosternon suburum hippocrepis* (2->1), Angolachelonia (0->2), Plesiochelyidae (2->0).

**Character 69 (Evers & Benson [2018]: character 68).** Vomer, contribution to the upper triturating surface: 0 = absent, triturating surface narrow to absent; 1 = present.

*Character optimisation*: Unambiguous: *Allopleuron*+++ (0->1), *Chrysemys picta* (0->1), *Geoclemys hamiltonii* (0->1), Sandownidae (0->1); DELTRAN: *Sternotherus* (0->1), *Staurotypus* (0->1); ACCTRAN: Kinosternidae (0->1), *Kinosternon suburum hippocrepis* (1->0).

**Character 70 (Evers & Benson [2018]: character 69).** Vomer, median trough on dorsal surface posterior to sulcus vomeri: 0 = absent, dorsal surface of vomer flat or transversely convex; 1 = present, dorsal surface bears a median trough that extends posteriorly from the sulcus vomeri.

*Character optimisation*: Unambiguous: Cryptodira (0->1), total-group Chelonioidea (1->0), *Lissemys punctata* (1->0); DELTRAN: *Notochelone* (0->1), (*Lepidochelys* *olivacea* + *Lepidochelys* *kempii*) (0->1), *Eretmochelys* *imbricata* (0->1), *Natator* *depressus* (0->1), *Eubaena cephalica* (0->1), *Proganochelys quenstedti* (0->1); ACCTRAN: (*Santanachelys* + *Notochelone*) (0->1), crown-group Cheloniidae (0->1), *Caretta Caretta* (1->0), *Chelonia* *mydas* (1->0), (*Eubaena* + Pleurosternidae) (0->1), *Proganochelys quenstedti* (0->1).

**Character 71 (Evers & Benson [2018]: character 70)***.* Foramen orbito-nasale: 0 = formed as true foramen that is surrounded by bone from all sides; 1 = foramen orbito-nasale is not completely surrounded by bone and coalescent with the passage between the fossa orbitalis and the fossa nasalis.

*Character optimisation*: Unambiguous: crown-group Pleurodira (0->1).

**Character 72 (Evers & Benson [2018]: character 71).** Foramen orbito-nasale, contribution of vomer: 0 = absent; 1 = present.

*Character optimisation*: Unambiguous: *Gopherus polyphemus* (0->1), crown-group Trionychia (0->1), *Brachyopsemys* *tingitana* (0->1); DELTRAN: *Dermochelys coriacea* (0->1), (*Eretmochelys* ++ *Lepidochelys*) (0->1), *Natator* *depressus* (0->1); ACCTRAN: Dermochelyidae (0->1), crown-group Cheloniidae (0->1), *Chelonia* *mydas* (1->0).

**Character 73 (Evers & Benson [2018]: character 72)***.* Foramen orbito-nasale, contribution of the maxilla: 0 = absent; 1 = present.

*Character optimisation*: Unambiguous: *Emys orbicularis* (1->0), Testuguria (1->0), total-group Chelidae (1->0); DELTRAN: Xinjiangchelyidae+++ (0->1), *Meiolania planiceps* (0->1); ACCTRAN: *Australochelys*+++ (0->1), Paracryptodira (1->0).

**Character 74 (Evers & Benson [2018]: character 73).** Quadrate, precolumellar fossa: 0 = absent; 1 = present.

*Character optimisation*: Unambiguous: *Araripemys barretoi* (0->1), crown-group Pelomedusoides (0->1); DELTRAN: *Sinemys gamera* (0->1); ACCTRAN: (*Sinemys gamera* + *Sinemys lens*) (0->1).

**Character 75 (Evers & Benson [2018]: character 74).** Quadrate, development of the cavum tympani: 0 = shallow, but not developed anteroposteriorly; 1 = shallow, but anteroposteriorly developed; 2 = deep and anteroposteriorly developed.

*Character optimisation*: DELTRAN: (*Kayentachelys*+*Eileanchelys*)+++ (0->2), *Australochelys* *africanus* (0->1); ACCTRAN: *Australochelys*+++ (0->1), (*Kayentachelys*+*Eileanchelys*)+++ (1->2).

**Character 76 (Evers & Benson [2018]: character 75)***.* Quadrate, anterior margin of the cavum tympanum: 0 = formed entirely by the quadrate; 1 = formed by the quadratojugal, which overlaps the lateral surface of the quadrate, reaching the anterior margin of the cavum tympanum.

*Character optimisation*: Unambiguous: Protostegidae (1->0), *Natator* *depressus* (1->0), *Platysternon megacephalum* (1->0), (*Testudo* + *Chelonoidis*) (1->0), *Judithemys* *sukhanovi* (0->1), (*Xinjiangchelys* *wusu* + *Xinjiangchelys* *radiplicatoides*) (0->1), *Eubaena cephalica* (0->1), *Meiolania planiceps* (0->1); DELTRAN: Durocryptodira (0->1), *Santanachelys* *gaffneyi* (0->1), *Archelon* *ischyros* (0->1), crown-group Trionychia (0->1), *Jurassichelon* *oleronensis* (0->1); ACCTRAN: Cryptodira (0->1), (*Santanachelys* + *Notochelone*) (0->1), (*Protostega* + *Archelon*) (0->1), *Adocus beatus* (1->0), (*Plesiochelys planiceps* ++ *Jurassichelon*) (0->1).

**Character 77 (Evers & Benson [2018]: character 76).** Quadrate, antrum postoticum: 0 = absent; 1 = incipient, the antrum postoticum is completely formed within quadrate (irrespective of the elements involved in forming the margin of the cavum tympanum); 2 = antrum postoticum fully developed, and extending posterodorsally into the squamosal (i.e. there is a large posterodorsal fenestra in the quadrate that leads to a pocket within the squamosal). This character is scored as inapplicable for turtles without a cavum tympanum.

*Character optimisation*: Unambiguous: *Bouliachelys suteri* (2->0), (*Desmatochelys* *lowii* + *Desmatochelys* *padillai*) (2->1), *Allopleuron hoffmanni* (2->1), Testudinidae (2->1), (*Xinjiangchelys* *wusu* + *Xinjiangchelys* *radiplicatoides*) (2->1), Pleurosternidae (2->1), *Meiolania planiceps* (2->0); DELTRAN: *Chubutemys*+++ (0->2), *Carettochelys insculpta* (2->0), *Allaeochelys libyca* (2->1), *Kayentachelys* *aprix* (0->1); ACCTRAN: (*Kayentachelys*+*Eileanchelys*)+++ (0->1), *Chubutemys*+++ (1->2), total-group Carettochelyidae (2->0), *Allaeochelys libyca* (0->1).

**Character 78 (Evers & Benson [2018]: character 77).** Quadrate, incisura columellae auris: 0 = absent, stapes extends posteroventrall to quadrate body; 1 = present, but open posteroventrally; 2 = present and closed, but only enclosing the stapes; 3 = present and closed, enclosing stapes and the Eustachian tube.

*Character optimisation*: Unambiguous: (*Kayentachelys*+*Eileanchelys*)+++ (0->1), crown-group Chelydridae (1->2), *Platysternon megacephalum* (1->2), Testudinidae (1->2), crown-group Trionychia (1->2), (*Sinemys gamera* + *Sinemys lens*) (1->2), *Meiolania planiceps* (1->3); DELTRAN: *Sternotherus* (1->2), *Staurotypus* (1->2), (*Sandownia* + *Angolachelys*) (1->2), *Leyvachelys cipadi* (1->2), crown-group Chelidae (1->3), crown-group Pelomedusoides (1->3), *Galianemys whitei* (1->2); ACCTRAN: Kinosternidae (1->2), *Kinosternon suburum hippocrepis* (2->1), Sandownidae (1->2), *Brachyopsemys* *tingitana* (2->1), crown-group Pleurodira (1->3), *Araripemys barretoi* (3->1), *Galianemys whitei* (3->2).

**Character 79 (Evers & Benson [2018]: character 78)***.* Quadrate, formation of incisura columella auris: 0 = formed exclusively by quadrate; 1 = formed by quadrate and squamosal and/or quadratojugal.

*Character optimisation*: Unambiguous: *Meiolania planiceps* (0->1)

**Character 80 (Evers & Benson [2018]: character 79).** Quadrate, processus trochlearis oticum: 0 = absent; 1 = present, very reduced; 2 = present, large forming a well defined musculatory facet.

*Character optimisation*: Unambiguous: *Chubutemys*+++ (0->1), Testudines (1->2), (*Chelonia* + *Natator*) (2->1), *Allopleuron hoffmanni* (2->1), total-group Dermatemydidae (2->1), crown-group Pleurodira (2->0), *Kirgizemys dmitrievi* (1->2), *Kallokibotion bajazidi* (1->2); DELTRAN: *Dermochelys coriacea* (2->1), *Emys orbicularis* (2->1), *Platysternon megacephalum* (2->1), Testudinidae (2->1); ACCTRAN: Dermochelyidae (2->1), Testudinoidea (2->1), *Chrysemys picta* (1->2), *Geoclemys hamiltonii* (1->2).

**Character 81 (Evers & Benson [2018]: character 80).** Quadrate, contribution to the musculatory facet of the processus trochlearis oticum: 0 = extensive contribution; 1 = small contribution, facet formed principally by the protic and/or parietal. This character is scored as inapplicable when a processus trochlearis oticum is absent (ch 79.0).

*Character optimisation*: Unambiguous: *Allopleuron hoffmanni* (0->1), Trionychidae (0->1), *Ordosemys* sp. IVPP V12092 (0->1); DELTRAN: *Protostega* *gigas* (0->1), *Pleurosternon bullockii* (0->1); ACCTRAN: (*Protostega* + *Archelon*) (0->1), Pleurosternidae (0->1).

**Character 82 (Evers & Benson [2018]: character 81)***.* Quadrate, width of processus trochlearis oticum: 0 = the otic process spans all the mediolateral space between the braincase wall and the lateral surface of the skull; 1 = the otic process is limited to the medial part of the otic chamber, and there is a deep recess laterally. This character is scored as inapplicable when a processus trochlearis oticum is absent (ch 79.0).

*Character optimisation*: Unambiguous: total-group Chelydridae (0->1), *Staurotypus* (0->1), *Adocus beatus* (0->1), Thalassochelydia (0->1), *Jurassichelon* *oleronensis* (1->0), (*Ordosemys* + *Dracochelys*) (0->1), *Annemys* sp. IVPP V18106 (0->1); DELTRAN: Protostegidae (0->1), *Notochelone* (1->0), total-group Cheloniidae (0->1); ACCTRAN: Protostegidae+++ (0->1), (*Santanachelys* + *Notochelone*) (1->0), Dermochelyidae (1->0).

**Character 83 (Evers & Benson [2018]: character 82).** Quadrate, quadrate-basisphenoid contact: 0 = absent; 1 = present.

*Character optimisation*: Unambiguous: *Chelodina* (0->1), total-group Pelomedusoides (0->1).

**Character 84 (Evers & Benson [2018]: character 83)***.* Quadrate, infolding ridge on the posterior surface of the quadrate ventral to the incisura columella auris: 0 = absent; 1 = present.

*Character optimisation*: Unambiguous: *Bouliachelys suteri* (0->1), Angolachelonia (0->1), *Ordosemys* sp. IVPP V12092 (0->1).

**Character 85 (Evers & Benson [2018]: character 84).** Quadrate, direction of cranial articular process: 0 = ventrolaterally directed; 1 = with strong posterior inclination.

*Character optimisation*: Unambiguous: *Allopleuron hoffmanni* (0->1), *Macrochelys temminckii* (0->1), Angolachelonia (0->1), *Galianemys whitei* (0->1), *Kayentachelys* *aprix* (0->1).

**Character 86 (Evers & Benson [2018]: character 85)***.* Posterior quadrate fossa: 0 = absent; 1 = present.

*Character optimisation*: Unambiguous: total-group Carettochelyidae (0->1).

**Character 87 (Evers & Benson [2018]: character 86).** Stapes, lateral articulation: 0 = stapes articulates with medial surface of the quadrate, quadrate has stapedial pit; 1 = stapes articulates with tympanic membrane, pit on medial surface of quadrate is absent.

*Character optimisation*: DELTRAN: *Australochelys*+++ (0->1); ACCTRAN: *Australochelys*+++ (0->1).

**Character 88 (Evers & Benson [2018]: character 87).** Epipterygoid: 0 = present; 1 = absent.

*Character optimisation*: Unambiguous: crown-group Pleurodira (0->1), *Eubaena cephalica* (0->1); DELTRAN: (*Bouliachelys* + *Rhinochelys*) (0->1), *Desmatochelys* *lowii* (0->1), *Dermochelys coriacea* (0->1), crown-group Cheloniidae (0->1), *Puppigerus camperi* (0->1), *Cabindachelys* *landanensis* (0->1), *Nichollsemys baieri* (0->1); ACCTRAN: Protostegidae+++ (0->1), (*Ocepechelon* ++ *Protostega*) (1->0), *Allopleuron*+++ (1->0), crown-group Cheloniidae (0->1), *Puppigerus camperi* (0->1), *Cabindachelys* *landanensis* (0->1), *Corsochelys* *halinches* (1->0).

**Character 89 (Evers & Benson [2018]: character 88).** Epipterygoid, shape: 0 = rod-like element; 1 = laminar element. This character is scored as inapplicable if an epipterygoid is absent (ch 87.1).

*Character optimisation*: Unambiguous: *Annemys* *latiens* (1->0); DELTRAN: *Kallokibotion*+++ (0->1); ACCTRAN: *Chubutemys*+++ (0->1).

**Character 90 (Evers & Benson [2018]: character 89).** Pterygoid, pterygoid teeth: 0 = present; 1 = absent.

*Character optimisation*: Unambiguous: *Nichollsemys baieri* (1->0); DELTRAN: *Chubutemys*+++ (0->1), *Australochelys* *africanus* (0->1); ACCTRAN: *Australochelys*+++ (0->1), (*Kayentachelys* + *Eileanchelys*) (1->0).

**Character 91 (Evers & Benson [2018]: character 90).** Pterygoid, basipterygoid process and basipterygoid articulation: 0 = basipterygoid process present with a movable basiptergoid articulation; 1 = basipterygoid process present with a sutured basipterygoid articulation; 2 = basipterygoid process absent and sutured basipterygoid articulation.

*Character optimisation*: Unambiguous: (*Jurassichelon* + *Portlandemys*) (2->1), *Eubaena cephalica* (1->2), *Meiolania planiceps* (1->2); DELTRAN: *Australochelys*+++ (0->1), Testudines (1->2), *Sandownia* *harrisi* (2->1), *Judithemys* *sukhanovi* (1->2); ACCTRAN: *Australochelys*+++ (0->1), Sinemydidae/Macroabenidae+++ (1->2), Sandownidae (2->1), (*Sinemys* ++ *Kirgizemys*) (2->1).

**Character 92 (Evers & Benson [2018]: character 91).** Pterygoid, pterygoid-basioccipital contact: 0 = absent; 1 = present.

*Character optimisation*: Unambiguous: *Kallokibotion*+++ (0->1), *Eosphargis breineri* (1->0), *Puppigerus camperi* (1->0), *Emys orbicularis* (1->0), crown-group Pleurodira (1->0), (*Sinemys gamera* + *Sinemys lens*) (1->0), Xinjiangchelyidae (1->0), *Xinjiangchelys radiplicatoides* (0->1).

**Character 93 (newly added from Hooks [1998]: character 10)**: Basioccipital, anterolateral edge of basioccipital with knob-like processes fitting into sockets on the posterior processes of the pterygoids: 0 = absent; 1 = present.

*Character optimisation*: Unambiguous: Protostegidae (0->1), *Ocepechelon bouyai* (1->0).

**Character 94 (Evers & Benson [2018]: character 92).** Pterygoid, processus trochelaris pterygoideus: 0 = absent; 1 = present.

*Character optimisation*: Unambiguous: crown-group Pleurodira (0->1).

**Character 95 (Evers & Benson [2018]: character 93).** Pterygoid, foramen palatinum posterius: 0 = present; 1 = present, but open laterally; 2 = absent.

*Character optimisation*: Unambiguous: (*Ocepechelon* ++ *Protostega*) (1->2), Dermochelyidae (0->2), (*Erquelinnesia* ++ *Lepidochelys*) (0->2), Plesiochelyidae (0->1), *Portlandemys mcdowelli* (1->0), *Angolachelys* *mbaxi* (0->2); DELTRAN: (*Bouliachelys* + *Rhinochelys*) (0->1), *Desmatochelys* *padillai* (0->1), (*Ctenochelys* + *Cabindachelys*) (2->0); ACCTRAN: Protostegidae (0->1), *Desmatochelys* *lowii* (1->0), (*Ctenochelys* ++ *Peritresius*) (2->0).

**Character 96 (Evers & Benson [2018]: character 94).** Pterygoid, medial contact of pterygoid: 0 = present, pterygoids in a very long medial contact with one another, longer than the basisphenoid total length in midline; 1 = present, pterygoids in medial contact with one another, contact length equal or shorter than the basisphenoid total length in midline; 2 = absent, contact of the basisphenoid with the vomer and/or palatines present.

*Character optimisation*: Unambiguous: total-group Chelonioidea (1->0), *Dermochelys coriacea* (0->1), crown-group Chelydridae (1->0), *Platysternon megacephalum* (1->0), crown-group Trionychia (1->2), Angolachelonia (1->0), *Jurassichelon* *oleronensis* (0->1), *Araripemys barretoi* (1->2), *Xinjiangchelys* *radiplicatoides* (1->2), Pleurosternidae (1->2); DELTRAN: *Rhinochelys* *pulchriceps* (0->1); ACCTRAN: (*Rhinochelys* *pulchriceps* + *Rhinochelys* *nammourensis*) (0->1).

**Character 97 (Evers & Benson [2018]: character 95).** Pterygoid, pterygoid contribution to foramen palatinum posterius: 0 = present; 1 = absent. This character is scored inapplicable when the foramen palatinum posterius is absent (ch. 66.0).

*Character optimisation*: Unambiguous: Cryptodira (0->1), *Solnhofia parsoni* (0->1), *Araripemys barretoi* (0->1), *Podocnemis* (0->1), *Chubutemys copelloi* (0->1); DELTRAN: (*Santanachelys* ++ *Rhinochelys*) (1->0), *Desmatochelys* *padillai* (1->0), *Cabindachelys* *landanensis* (1->0), *Nichollsemys baieri* (1->0), *Toxochelys* sp. (1->0), *Chelonoidis* sp. (1->0), *Gopherus polyphemus* (1->0), *Apalone spinifera* (1->0), *Sandownia* *harrisi* (0->1); ACCTRAN: total-group Chelonioidea (1->0), *Bouliachelys suteri* (0->1), *Desmatochelys* *lowii* (0->1), *Allopleuron*+++ (0->1), *Cabindachelys* *landanensis* (1->0), Testudinidae (1->0), *Testudo* (0->1), (*Apalone* + *Petrochelys*) (1->0), (*Sandownia* + *Angolachelys*) (0->1).

**Character 98 (Evers & Benson [2018]: character 96).** Pterygoid, contact with the exoccipital: 0 = absent; 1 = present.

*Character optimisation*: Unambiguous: *Meiolania*+++ (0->1), Dermochelyidae (1->0), *Sternotherus* (1->0), total-group Dermatemydidae (1->0), Testudinoidea (1->0), (*Jurassichelon* + *Portlandemys*) (0->1), *Annemys* sp. IVPP V18106 (1->0); DELTRAN: (*Santanachelys* + *Notochelone*) (1->0), *Calcarichelys gemma* (1->0), Thalassochelydia (1->0), crown-group Pleurodira (1->0), (*Sinemys gamera* + *Sinemys lens*) (1->0), *Kirgizemys dmitrievi* (1->0); ACCTRAN: (*Calcarichelys* + *Rhinochelys*) (1->0), (*Rhinochelys* *pulchriceps* + *Rhinochelys* *nammourensis*) (0->1), total-group Pleurodira (1->0), Sandownidae (0->1), (*Sinemys* ++ *Kirgizemys*) (1->0), (*Ordosemys* + *Dracochelys*) (0->1).

**Character 99 (Evers & Benson [2018]: character 97**). Pterygoid, fossa podocnemidoidea or cavum pterygoidei: 0 = absent; 1 = present.

*Character optimisation*: DELTRAN: *Podocnemis* (0->1), *Galianemys whitei* (0->1); ACCTRAN: total-group Pelomedusoides (0->1), *Pelomedusa subrufa* (1->0).

**Character 100 (Evers & Benson [2018]: character 98).** Pterygoid, lateral margin: 0 = a processus pterygoideus externus is developed as a process that projects into the subtemporal fenestra; 1 = the lateral margin of the pterygoid is gently expanded laterally and/or expanded dorsoventrally; 2 = absent, i.e. the lateral margin of the pterygoid forms a straight or concave outline that forms the medial margin of the subtemporal fenestra. Scored inapplicable for pleurodires.

*Character optimisation*: Unambiguous: (*Protostega* + *Archelon*) (0->2), Dermochelyidae (0->2), *Caretta Caretta* (0->1), *Chelonia* *mydas* (0->2), *Sternotherus* (0->1), crown-group Trionychia (0->1); DELTRAN: *Procolpochelys charlestonensis* (0->1), *Solnhofia parsoni* (0->1), Sandownidae (0->1); ACCTRAN: (*Procolpochelys* ++ *Erquelinnesia*) (0->1), total-group Pleurodira (0->1), Plesiochelyidae (1->0).

**Character 101 (Evers & Benson [2018]: character 99).** Pterygoid, processus pterygoideus externus: 0 = forming an extensive process that contacts the maxilla anterolaterally at the posteromedial end of the triturating surface, is anteriorly sutured to the anterior palate, and has a posterior projection into the subtemporal fenestra; 1 = forming a large lateral wing that projects as a free process into the subtemporal fenestra; 2 = forming a pointed triangular process that projects laterally into the subtemporal fenestra. Scored inapplicable for taxa that lack a processus pterygoideus externus (i.e. ch 98.1 or 98.2)

*Character optimisation*: Unambiguous: Protostegidae (0->1), *Desmatochelys* *padillai* (1->2), crown-group Chelydridae (2->0), *Portlandemys mcdowelli* (1->0), *Ordosemys* sp. IVPP V12092 (0->2); DELTRAN: Cryptodira (0->2), (*Ctenochelys* + *Cabindachelys*) (2->0), *Nichollsemys baieri* (2->0), *Toxochelys* sp. (2->0), Plesiochelyidae (0->1); ACCTRAN: Testudines (0->1), Cryptodira (1->2), total-group Chelonioidea (2->0), *Allopleuron*+++ (0->2), (*Ctenochelys* ++ *Peritresius*) (2->0).

**Character 102 (Evers & Benson [2018]: character 100).** Pterygoid, vertical flange on anterolateral margin of the pterygoid: 0 = absent; 1 = present. Zhou *et al.* (2014) & Joyce (2007: ch 67) (Pterygoid I).

*Character optimisation*: Unambiguous: Paracryptodira+++ (0->1), *Lepidochelys* *olivacea* (1->0), *Chelonia* *mydas* (1->0), *Puppigerus camperi* (1->0), *Platysternon megacephalum* (1->0), *Testudo* (1->0), *Lissemys punctata* (1->0), *Solnhofia parsoni* (1->0); DELTRAN: *Procolpochelys charlestonensis* (1->0), *Kinosternon suburum hippocrepisis* (1->0), *Staurotypus* (1->0); ACCTRAN: (*Procolpochelys* ++ *Erquelinnesia*) (1->0), Kinosternidae (1->0), *Sternotherus* (0->1).

**Character 103 (Evers & Benson [2018]: character 101).** Pterygoid, level of the position of the pterygoid respect to basisphenoid: 0 = both bones are at the same level on ventral surface; 1 = two different levels, creating a step between the two bones.

*Character optimisation*: Unambiguous: *Eosphargis breineri* (0->1), *Allopleuron*+++ (0->1), *Chelonia* *mydas* (1->0), *Platysternon megacephalum* (0->1); DELTRAN: (*Ctenochelys* + *Cabindachelys*) (1->0); ACCTRAN: (*Ctenochelys* ++ *Peritresius*) (1->0).

**Character 104 (Evers & Benson [2018]: character 102).** Pterygoid, ventral median ridge: 0 = incipient to absent; 1 = present, ridge spans nearly the full length of the pterygoids, sometimes reaching the most posterior portion of the vomer. This character is scored as inapplicable for taxa in which the pterygoids lack a midline contact.

*Character optimisation*: Unambiguous: (*Protostega* + *Archelon*) (0->1), total-group Cheloniidae (0->1), *Lepidochelys* *kempii* (1->0), *Chelonia* *mydas* (1->0), *Gopherus polyphemus* (0->1); DELTRAN: *Eochelone brabantica* (1->0), (*Ctenochelys* + *Cabindachelys*) (1->0); ACCTRAN: (*Puppigerus* ++ *Ctenochelys*) (1->0), *Puppigerus camperi* (0->1).

**Character 105 (Evers & Benson [2018]: character 103).** Pterygoid, extending laterally almost reaching the mandibular condyle facet: 0 = absent; 1 = present, the pterygoid contacts the medial edge of the mandibular condyle when is seem in ventral view; 2 = present, the pterygoids extends not only laterally to reach the outline of the mandibular condyle facet, but also posteriorly far from the level of the condyles.

*Character optimisation*: Unambiguous: Protostegidae (0->1), *Nichollsemys baieri* (0->1), crown-group Trionychia (0->1), *Lissemys punctata* (1->2); DELTRAN: Angolachelonia (0->1), *Phrynops geoffroanus* (0->1), *Chelodina* (0->1), *Pelomedusa subrufa* (0->1), *Galianemys whitei* (0->1); ACCTRAN: total-group Pleurodira (0->1), total-group Chelidae (1->0), *Phrynops geoffroanus* (0->1), *Chelodina* (0->1), *Podocnemis* (1->0).

**Character 106 (Evers & Benson [2018]: character 104).** Pterygoid, ventral ridge on the palatal surface lateral to skull midline. 0 = absent; 1 = present, each pterygoid has a parasagittal ridge on its ventral surface.

*Character optimisation*: Unambiguous: *Chubutemys*+++ (0->1), *Calcarichelys gemma* (1->0), *Corsochelys*+++ (1->0), *Ctenochelys* (0->1), *Dermatemys mawii* (1->0), Emydidae (1->0), Trionychidae (1->0), *Plesiochelys etalloni* (1->0), *Angolachelys* *mbaxi* (1->0), crown-group Pleurodira (1->0), *Meiolania planiceps* (1->0); DELTRAN: *Ocepechelon bouyai* (1->0), *Testudo* (1->0), *Gopherus polyphemus* (1->0); ACCTRAN: (*Ocepechelon* ++ *Protostega*) (1->0), Testudinidae (1->0), *Chelonoidis* sp. (0->1).

**Character 107 (Evers & Benson [2018]: character 105)***.* Pterygoid, extent of ventral ridge on the palatal surface lateral to skull midline: 0 = each ridge extends along most of the ventral surface of the pterygoid, from the anteromedial margin of the pterygoid fossa to the processus pterygoideus externus; 1 = each ridge extends only along the posterior part of the pterygoid, along the level of the parabasisphenoid. This character is scored inapplicable in turtles in which ventral pterygoid ridges are absent (ch 104.0).

*Character optimisation*: Unambiguous: total-group Chelonioidea (0->1), *Santanachelys* *gaffneyi* (1->0), *Staurotypus* (0->1), *Arundelemys dardeni* (0->1); DELTRAN: *Chelonoidis* sp. (0->1), *Brachyopsemys* *tingitana* (0->1), *Leyvachelys cipadi* (0->1); ACCTRAN: Testudinidae (0->1), Sandownidae (0->1), (*Sandownia* + *Angolachelys*) (1->0).

**Character 108 (Evers & Benson [2018]: character 106).** Pterygoid/Quadrate, flooring of cavum acustico-jugulare and recessus scalae typmani: 0 = absent; 1 = present, formed primarily by the posterior part of the pterygoid; 2 = present, produced by the ventral process of the quadrate or prootic or a posterolateral expansion of the parabasisphenoid.

*Character optimisation*: Unambiguous: *Kallokibotion*+++ (0->1), crown-group Pleurodira (1->2).

**Character 109 (Evers & Benson [2018]: character 107).** Pterygoid, posterior process: 0 = posterior process of pterygoid is absent, the cranioquadrate space or posterior foramen for the canalis cavernosus is not covered by the pterygoid; 1 = posterior process of pterygoid present but very short, process extends posteriorly to cover the posterior foramen for the canalis cavernosus (i.e. the modified cranioquadrate space), but the cavum-acustico jugulare remains largely exposed ventrally; 2 = posterior process of the pterygoid present and developed as an extensive sheet that projects posteriorly and covers large parts of the cavum acustico-jugulare.

*Character optimisation*: Unambiguous: (*Kayentachelys*+*Eileanchelys*)+++ (0->1), *Kallokibotion*+++ (1->2), *Emarginachelys cretacea* (2->1), crown-group Pleurodira (2->0), *Sinemys lens* (2->1), Pleurosternidae (2->1); DELTRAN: *Solnhofia parsoni* (2->1), *Plesiochelys planiceps* (2->1), *Plesiochelys* *bigleri* (2->1), *Kirgizemys* *hoburensis* (2->1), *Annemys* *levensis* (2->1), *Annemys* sp. IVPP V18106 (2->1); ACCTRAN: Thalassochelydia (2->1), (*Jurassichelon* + *Portlandemys*) (1->2), *Plesiochelys etalloni* (1->2), (*Kirgizemys* *hoburensis* + *Kirgizemys dmitrievi*) (2->1), Xinjiangchelyidae (2->1), (*Xinjiangchelys* *wusu* + *Xinjiangchelys* *radiplicatoides*) (1->2).

**Character 110 (Evers & Benson [2018]: character 108).** Pterygoid, development of a posteromedial wing covering partially to completely the basisphenoid and sometime the basioccipital too, seen in ventral view of the skull: 0 = absent; 1 = present.

*Character optimisation*: Unambiguous: Sandownidae (0->1).

**Character 111 (Evers & Benson [2018]: character 109).** Pterygoid, pterygoid fossa: 0 = weakly developed; 1 = developed as a deep concavity between the articular process of the quadrate and the basicranium.

*Character optimisation*: Unambiguous: total-group Cheloniidae (0->1), *Emarginachelys cretacea* (0->1), *Platysternon megacephalum* (0->1), *Allaeochelys libyca* (0->1), *Adocus beatus* (0->1), (*Plesiochelys planiceps* ++ *Jurassichelon*) (0->1), Sandownidae (0->1), *Angolachelys* *mbaxi* (1->0), Sinemydidae (0->1), *Xinjiangchelys* *radiplicatoides* (1->0), (*Kayentachelys* + *Eileanchelys*) (0->1); DELTRAN: Xinjiangchelyidae (0->1), Paracryptodira (0->1); ACCTRAN: Paracryptodira+++ (0->1), Sinemydidae/Macroabenidae+++ (1->0).

**Character 112 (Evers & Benson [2018]: character 110).** Supraoccipital, crista supraoccipitalis: 0 = poorly developed; 1 = protruding significantly posterior to the foramen magnum.

*Character optimisation*: Unambiguous: *Chubutemys*+++ (0->1), *Ocepechelon bouyai* (1->0), Dermochelyidae (1->0), *Kinosternon suburum hippocrepis* (1->0), (*Sandownia* + *Angolachelys*) (1->0); DELTRAN: *Jurassichelon* *oleronensis* (1->0), Chelinae (1->0), *Chelodina* (1->0), *Galianemys whitei* (1->0); ACCTRAN: (*Jurassichelon* + *Portlandemys*) (1->0), crown-group Pleurodira (1->0), *Elseya dentata* (0->1), crown-group Pelomedusoides (0->1).

**Character 113 (Evers & Benson [2018]: character 111).** Supraoccipital, large supraoccipital exposure on dorsal skull roof: 0 = absent; 1 = present.

*Character optimisation*: Unambiguous: *Meiolania planiceps* (0->1).

**Character 114 (Evers & Benson [2018]: character 112).** Supraoccipital, horizontal crest in the crista supraoccipitalis: 0 = absent or poorly developed anteriorly; 1 = present, along the entire crista supraoccipitalis.

*Character optimisation*: Unambiguous: crown-group Trionychia (0->1).

**Character 115 (Evers & Benson [2018]: character 113).** Supraoccipital, fossa on the posterodorsal surface of the floor of the supratemporal fossa: 0 = absent; 1 = present, fossa is formed on the lateral surface of the suparoccipital, dorsal to the contact area between exoccipital, opisthotic, and supraoccipital.

*Character optimisation*: Unambiguous: (*Kayentachelys*+*Eileanchelys*)+++ (1->0), *Corsochelys*+++ (0->1), (*Eretmochelys* ++ *Lepidochelys*) (1->0), (*Puppigerus* + *Eochelone*) (1->0), crown-group Chelydridae (0->1), *Chrysemys picta* (0->1), *Testudo* (0->1), crown-group Trionychia (0->1), Thalassochelydia (0->1), *Galianemys whitei* (0->1); DELTRAN: *Notochelone* (0->1), (*Sinemys* ++ *Kirgizemys*) (0->1); ACCTRAN: (*Santanachelys* + *Notochelone*) (0->1), (*Judithemys* ++ *Sinemys*) (0->1).

**Character 116 (Evers & Benson [2018]: character 114).** Exoccipital, medial contact of exoccipitals dorsal to foramen magnum: 0 = absent; 1 = present.

*Character optimisation*: Unambiguous: *Kinosternon suburum hippocrepis* (0->1); DELTRAN: Chelinae (0->1), *Chelodina* (0->1); ACCTRAN: crown-group Chelidae (0->1), *Elseya dentata* (1->0).

**Character 117 (Evers & Benson [2018]: character 115).** Exoccipital, median contact of exoccipitals in the floor of the foramen magnum, excluding the basioccipital from the latter: 0 = absent; 1 = present.

*Character optimisation*: Unambiguous: Xinjiangchelyidae+++ (0->1), (*Desmatochelys* ++ *Protostega*) (0->1), *Chelonia* *mydas* (0->1), (*Argillochelys* ++ *Puppigerus*) (0->1), *Sternotherus* (1->0), *Dermatemys mawii* (1->0), Angolachelonia (1->0), (*Plesiochelys planiceps* ++ *Jurassichelon*) (0->1), *Brachyopsemys* *tingitana* (0->1), *Araripemys barretoi* (1->0), *Podocnemis* (1->0); DELTRAN: total-group Chelonioidea (1->0), *Chrysemys picta* (1->0), *Platysternon megacephalum* (1->0), *Geoclemys hamiltonii* (1->0), *Ordosemys* sp. IVPP V12092 (1->0); ACCTRAN: Durocryptodira (1->0), Chelydroidea (0->1), *Emys orbicularis* (0->1), Testudinidae (0->1), Sinemydidae (1->0).

**Character 118 (Evers & Benson [2018]: character 116).** Foramen nervi hypoglossi (XII), ventral covering: 0 = exposed in ventral view; 1 = covered in ventral view by an extension of the pterygoid and the basioccipital; 2 = covered in ventral view an extension of the basioccipital; 3 = covered in ventral view by an expansion of the exoccipital and basioccipital.

*Character optimisation*: Unambiguous: Protostegidae (0->2), (*Protostega* + *Archelon*) (2->0), *Eretmochelys* *imbricata* (0->1), *Macrochelys temminckii* (0->1), Kinosternoidea (0->2), *Sternotherus* (2->0), total-group Trionychia (0->1), total-group Carettochelyidae (1->2), (*Sandownia* + *Angolachelys*) (0->1), *Podocnemis* (0->2), (*Ordosemys* + *Dracochelys*) (0->2), (*Eubaena* + Pleurosternidae) (0->2); DELTRAN: Emysternia (0->2), *Geoclemys hamiltonii* (0->2), *Solnhofia parsoni* (0->2), *Plesiochelys planiceps* (0->2), Chelinae (0->3), Chelodininae (0->2), (*Annemys* sp. IVPP V18106 ++ *Xinjiangchelys*) (0->2); ACCTRAN: Testudinoidea (0->2), Testudinidae (2->0), Thalassochelydia (0->2), (*Jurassichelon* + *Portlandemys*) (2->0), crown-group Chelidae (0->2), Chelinae (2->3), (*Annemys* *latiens* ++ *Xinjiangchelys*) (0->2).

**Character 119 (Evers & Benson [2018]: character 117).** Exoccipital, foramina nervi hypoglossi: 0 = the foramina nervi hypoglossi exit the exoccipital on the occipital surface posteriorly to the margin of the fenestra postotica; 1 = at least one foramen nervi hypoglossi opens within the recessus scalae tympani anterior to margin of the fenestra postotica, and the others exit the exoccipital on the occipital surface; 2 = all foramina nervi hypoglossi open within the recessus scalae tympani anterior to the margin of the fenestra postotica.

*Character optimisation*: Unambiguous: *Corsochelys* *halinches* (0->1), *Kallokibotion bajazidi* (0->1); DELTRAN: Chelinae (0->2), *Chelodina* (0->2); ACCTRAN: crown-group Chelidae (0->2), *Elseya dentata* (2->0).

**Character 120 (Evers & Benson [2018]: character 118)**. Basioccipital, morphology of the anteriormost part of the basioccipital: 0 = with two or one ventral tubercle; 1 = tubercle absent.

*Character optimisation*: Unambiguous: (*Kayentachelys*+*Eileanchelys*)+++ (0->1).

**Character 121 (Evers & Benson [2018]: character 119)**. Basioccipital, deep C-shaped concavity between basioccipital tubera: 0 = absent; 1 = present.

*Character optimisation*: Unambiguous: *Chubutemys*+++ (0->1), Paracryptodira+++ (1->0), *Baptemys wyomingensis* (0->1), *Solnhofia parsoni* (0->1); DELTRAN: *Eosphargis breineri* (0->1), total-group Cheloniidae (0->1), total-group Carettochelyidae (0->1), *Adocus beatus* (0->1); ACCTRAN: *Corsochelys*+++ (0->1), *Dermochelys coriacea* (1->0), total-group Trionychia (0->1), Trionychidae (1->0).

**Character 122 (Evers & Benson [2018]: character 120).** Basioccipital, basal tubera: 0 = the basal tubera are completely formed by the basioccipital; 1 = the exoccipitals and the basioccipital form the basal tubera; 2 = the pterygoids and the basioccipital form the basal tubera; 3 = the basal tubera are formed by the exoccipitals, basioccipital and pterygoids.

*Character optimisation*: Unambiguous: *Kallokibotion*+++ (0->2), Xinjiangchelyidae+++ (2->0), *Eretmochelys* *imbricata* (0->1), *Chelonia* *mydas* (0->1), *Chrysemys picta* (0->1), *Geoclemys hamiltonii* (0->3), total-group Carettochelyidae (0->3); DELTRAN: *Dermochelys coriacea* (0->3), *Eosphargis breineri* (0->1), *Puppigerus camperi* (0->1), *Eochelone brabantica* (0->2), *Sternotherus* (0->2), *Staurotypus* (0->2), *Baptemys wyomingensis* (0->2), *Solnhofia parsoni* (0->1), *Leyvachelys cipadi* (0->1), Chelinae (0->1), *Araripemys barretoi* (0->1), *Glyptops plicatulus* (2->0); ACCTRAN: Dermochelyidae (0->1), *Dermochelys coriacea* (1->3), (*Puppigerus* + *Eochelone*) (0->1), *Eochelone brabantica* (1->2), Kinosternoidea (0->2), *Kinosternon suburum hippocrepis* (2->0), *Dermatemys mawii* (2->0), total-group Pleurodira (0->1), Plesiochelyidae (1->0), Chelodininae (1->0), total-group Pelomedusoides (1->0), Pleurosternidae (2->0).

**Character 123 (Evers & Benson [2018]: character 121**). Prootic, dorsal exposure: 0 = large; 1 = very reduced or absent.

*Character optimisation*: Unambiguous: Emydidae (0->1), *Geoclemys hamiltonii* (0->1), *Testudo* (0->1), *Annemys* *latiens* (0->1); DELTRAN: *Meiolania planiceps* (0->1), *Kallokibotion bajazidi* (0->1); ACCTRAN: *Chubutemys*+++ (0->1), Paracryptodira+++ (1->0).

**Character 124 (Evers & Benson [2018]: character 122).** Prootic, lateral semicircular canal enclosure by bone: 0 = canal only formed by bone of the opisthotic, the prootic portion of the canal is not ossified and is instead medially confluent with the recessus labyrinthicus prooticus; 1 = prootic and opisthotic both contribute to the formation of the lateral semicircular canal.

*Character optimisation*: Unambiguous: *Bouliachelys suteri* (0->1), *Chrysemys picta* (0->1), *Pelodiscus sinensis* (0->1); DELTRAN: *Staurotypus* (0->1), *Dermatemys mawii* (0->1), (*Jurassichelon* + *Portlandemys*) (0->1), *Sandownia* *harrisi* (0->1), crown-group Pleurodira (0->1), Pleurosternidae (0->1), *Meiolania planiceps* (0->1), *Kallokibotion bajazidi* (0->1); ACCTRAN: *Chubutemys*+++ (0->1), Paracryptodira+++ (1->0), Kinosternoidea (0->1), Kinosterninae (1->0), total-group Pleurodira (0->1), Thalassochelydia (1->0), (*Jurassichelon* + *Portlandemys*) (0->1), (*Eubaena* + Pleurosternidae) (0->1).

**Character 125 (Evers & Benson [2018]: character 123).** Prootic, ventral process: 0 = ventral process is short and without extensive posterior contact with the pterygoid; 1 = ventral process is large, with a broad contact with the pterygoid along a posteriorly expanded footplate, forming parts of the floor of the inner ear cavity; 2 = ventral process is extensive, and forms parts of the floor of the basicranium so that it is visible on the ventral surface of the skull. This character is scored as inapplicable for taxa that lack an anatomically modern middle ear chamber.

*Character optimisation*: Unambiguous: Testudines (1->0), *Nichollsemys baieri* (0->1), *Chelydra serpentina* (0->1), Kinosternoidea (0->1), Testudinoidea (0->1), total-group Chelidae (0->2), *Pelomedusa subrufa* (0->2); DELTRAN: *Xinjiangchelys* *radiplicatoides* (1->0); ACCTRAN: (*Xinjiangchelys* *wusu* + *Xinjiangchelys* *radiplicatoides*) (1->0).

**Character 126 (Evers & Benson [2018]: character 124).** Prootic, unnamed foramen exiting into the subtemporal fossa from the canalis cavernosus: 0 = absent; 1 = present.

*Character optimisation*: Unambiguous: *Chelonia* *mydas* (0->1), *Platysternon megacephalum* (0->1), *Gopherus polyphemus* (0->1), total-group Carettochelyidae (0->1), *Pelomedusa subrufa* (0->1); DELTRAN: *Dermochelys coriacea* (0->1); ACCTRAN: Dermochelyidae (0->1).

**Character 127 (Evers & Benson [2018]: character 125).** Prootic/pterygoid, posteroventral elongation of the trigeminal foramen: 0 = absent, the trigeminal foramen is approximately circular or oval, but the ventral margin of the foramen is positioned above the level of the canalis/sulcus cavernosus; 1 = the trigeminal foramen is oval, anterodorsally-posteroventrally elongate and anteroventrally-posterodorsaly narrow, and the ventral margin is level with the canalis/sulcus cavernosus. This character is scored inapplicable for turtles without a trigeminal foramen (i.e. without a neomorphic secondary lateral wall of the braincase formed by the parietal and pterygoid).

*Character optimisation*: Unambiguous: *Notochelone* (0->1), *Kinosternon suburum hippocrepis* (0->1), *Platysternon megacephalum* (0->1), Xinjiangchelyidae (0->1), *Arundelemys dardeni* (0->1); DELTRAN: *Desmatochelys* *lowii* (0->1), total-group Cheloniidae (0->1), (*Ctenochelys* + *Cabindachelys*) (1->0), crown-group Chelydridae (0->1), crown-group Trionychia (0->1); ACCTRAN: (*Desmatochelys* ++ *Protostega*) (0->1), crown-group Chelonioidea (0->1), (*Ctenochelys* ++ *Peritresius*) (1->0), total-group Chelydridae (0->1), total-group Trionychia (0->1).

**Character 128 (Evers & Benson [2018]: character 126).** Prootic, recess on posterior surface of the element anterodorsolaterally to the fenestra ovalis: 0 = absent; 1 = present.

*Character optimisation*: Unambiguous: *Meiolania*+++ (0->1), *Chelonia* *mydas* (0->1), *Nichollsemys baieri* (0->1), *Chrysemys picta* (1->0), *Gopherus polyphemus* (1->0), *Allaeochelys libyca* (1->0), *Solnhofia parsoni* (1->0); DELTRAN: Protostegidae+++ (1->0), *Lepidochelys* *kempii* (0->1), *Caretta Caretta* (0->1), *Dermatemys mawii* (1->0); ACCTRAN: total-group Chelonioidea (1->0), (*Caretta* ++ *Lepidochelys*) (0->1), *Lepidochelys* *olivacea* (1->0), total-group Dermatemydidae (1->0).

**Character 129 (Evers & Benson [2018]: character 127).** Prootic, position of the geniculate ganglion and the split of the facial nerve into the hyomandibular and palatine branches: 0 = the geniculate ganglion is positioned with the canalis cavernosus; 1 = the geniculate ganglion is positioned within the facial nerve canal; 2 = the geniculate ganglion is positioned in the canalis carotici interni.

*Character optimisation*: Unambiguous: *Corsochelys* *halinches* (0->1); DELTRAN: Cryptodira (1->0), *Sandownia* *harrisi* (1->0), total-group Chelidae (1->2), crown-group Pelomedusoides (1->2); ACCTRAN: Testudines (1->0), Thalassochelydia (0->1), crown-group Pleurodira (0->2), *Galianemys whitei* (2->1).

**Character 130 (Evers & Benson [2018]: character 128).** Prootic/opisthotic, enclosure of fenestra ovalis: 0 = the fenestra ovalis is ventrally enclosed by the prootic and opisthotic; 1 = the prootic and opisthotic do not have a contact ventrally to the fenestra ovalis.

*Character optimisation*: Unambiguous: Testudines (0->1), (*Desmatochelys* ++ *Protostega*) (1->0), *Sternotherus* (1->0), (*Jurassichelon* + *Portlandemys*) (1->0), *Chelus fimbriatus* (1->0), *Chelodina* (1->0); DELTRAN: *Allopleuron hoffmanni* (1->0), *Nichollsemys baieri* (1->0), *Pleurosternon bullockii* (0->1); ACCTRAN: total-group Cheloniidae (1->0), (*Erquelinnesia* ++ *Lepidochelys*) (0->1), (*Eubaena* + Pleurosternidae) (0->1).

**Character 131 (Evers & Benson [2018]: character 129).** Opisthotic, wide transverse occipital plane with depression for the nuchal musculature: 0 = absent; 1 = present.

*Character optimisation*: Unambiguous: *Eubaena cephalica* (0->1), *Kallokibotion bajazidi* (0->1), *Australochelys* *africanus* (0->1).

**Character 132 (Evers & Benson [2018]: character 130).** Opisthotic, processus interfenestralis: 0 = developed as a robust ridge that does not form a ventrally projecting process and does not reach closely to the floor of the basicranium; 1 = incipient, developed as a ventrally low and mediolaterally broad, robust structure that separates the cavum labyrinthicum anteriorly from an incipient recessus scalae tympani posteriorly; 2 = present, developed as a ventrally directed process that separates the cavum labyrinthicum anteriorly from the recessus scalae tympani posteriorly.

*Character optimisation*: Unambiguous: (*Kayentachelys*+*Eileanchelys*)+++ (0->2), *Kayentachelys* *aprix* (2->1).

**Character 133 (Evers & Benson [2018]: character 131).** Opisthotic, development of the processus interfenestralis: 0 = developed as a ventrally directed process that separates the cavum labyrinthicum anteriorly from the recessus scalae tympani posteriorly, the process is not expanded at its ventral end, and almost or just about reaches the floor of the basicranium, but a small gap (hiatus postlagenum) usually remains; 1 = developed as a ventrally directed process that separates the cavum labyrinthicum anteriorly from the recessus scalae tympani posteriorly, but the process has a horizontally expanded footplate at its ventral end that is sutured to elements of the basicranium; 2 = developed as a ventrally directed process that separates the cavum labyrinthicum anteriorly from the recessus scalae tympani posteriorly, and the ventral surface of the process is integrated into the basicranium to form parts of the ventral surface of the cranium. This character is scored as inapplicable if an anatomically modern processus interfenestralis is absent (i.e. ch 131.0 or 131.1).

*Character optimisation*: Unambiguous: *Allopleuron hoffmanni* (0->1), Kinosternidae (0->1), (*Jurassichelon* + *Portlandemys*) (0->1), *Galianemys whitei* (0->1), *Annemys* sp. IVPP V18106 (0->1), (*Eubaena* + Pleurosternidae) (0->1), *Pleurosternon bullockii* (1->2); DELTRAN: *Desmatochelys* *lowii* (0->1), *Chelonoidis* sp. (0->2), *Gopherus polyphemus* (0->2), (*Sinemys gamera* + *Sinemys lens*) (0->2); ACCTRAN: (*Desmatochelys* ++ *Protostega*) (0->1), Testudinidae (0->2), *Testudo* (2->0), (*Judithemys* ++ *Sinemys*) (0->2).

**Character 134 (Evers & Benson [2018]: character 132).** Opisthotic, posterior surface of paroccipital process: 0 = the occipital side of the paroccipital process is developed as a posteriorly exposed, broad, planar or gently concave surface; 1 = the occipital side of the paroccipital process is dorsoventrally flattened and forms a posterior ridge that traverses the paroccipital process mediolaterally; 2 = the paroccipital process has a dorsoventrally convex surface.

*Character optimisation*: Unambiguous: total-group Chelonioidea (0->2), *Lepidochelys* *olivacea* (2->1), *Eretmochelys* *imbricata* (2->0), *Chelonia* *mydas* (2->0), *Eochelone brabantica* (2->0), crown-group Trionychia (0->1), *Angolachelys* *mbaxi* (1->2), Pleurosternidae (0->1); DELTRAN: *Notochelone* (2->0), Plesiochelyidae (0->2), Sandownidae (0->1), crown-group Pleurodira (0->1), (*Kayentachelys* + *Eileanchelys*) (0->1), *Australochelys* *africanus* (0->1); ACCTRAN: *Australochelys*+++ (0->1), *Chubutemys*+++ (1->0), (*Santanachelys* + *Notochelone*) (2->0), total-group Pleurodira (0->1), Thalassochelydia (1->0), Plesiochelyidae (0->2).

**Character 135 (Evers & Benson [2018]: character 133**). Fenestra perilymphatica: 0 = large; 1 = reduced in size to that of a small foramen.

*Character optimisation*: Unambiguous: *Bouliachelys suteri* (0->1); DELTRAN: *Pleurosternon bullockii* (0->1); ACCTRAN: (*Eubaena* + Pleurosternidae) (0->1).

**Character 136 (Evers & Benson [2018]: character 134).** Parabasisphenoid, paired pits on ventral surface of basisphenoid: 0 = absent; 1 = present.

*Character optimisation*: Unambiguous: (*Judithemys* ++ *Sinemys*) (0->1), *Dracochelys bicuspis* (1->0); DELTRAN: *Kayentachelys* *aprix* (0->1); ACCTRAN: (*Kayentachelys* + *Eileanchelys*) (0->1).

**Character 137 (Evers & Benson [2018]: character 135).** Parabasiphenoid, ventral surface: 0= flat to slightly convex, with posterior margin straight or slightly concave; 1= V-shaped crest, with posterior margin forming the basipterygoid process projected posterolaterally.

*Character optimisation*: Unambiguous: *Toxochelys* sp. (0->1); DELTRAN: *Eosphargis breineri* (0->1), total-group Cheloniidae (0->1); ACCTRAN: crown-group Chelonioidea (0->1), *Dermochelys coriacea* (1->0).

**Character 138 (Evers & Benson [2018]: character 136).** Parabasiphenoid, rough surface between basisphenoid and basioccipital: 0 = absent; 1 = present.

*Character optimisation*: Unambiguous: *Eosphargis breineri* (0->1), *Annemys* *levensis* (0->1).

**Character 139 (Evers & Benson [2018]: character 137)***.* Parabasisphenoid, posterolateral processes lapping onto the ventral surface of the basioccipital: 0 = absent; 1 = present.

*Character optimisation*: Unambiguous: *Sternotherus* (0->1), total-group Carettochelyidae (0->1), (*Eubaena* + Pleurosternidae) (0->1).

**Character 140 (Evers & Benson [2018]: character 138).** Parabasisphenoid, rostrum basisphenoidale: 0 = flat; 1 = flat base, but with trabeculae contact one another medially forming a short rod at the anterior end of the parabasisphenoid; 2 = singular median, rod-like, thick and rounded process.

*Character optimisation*: Unambiguous: *Eochelone brabantica* (2->1), *Ctenochelys* (2->1), *Corsochelys* *halinches* (2->1), *Platysternon megacephalum* (0->2), *Galianemys whitei* (0->2); DELTRAN: Protostegidae (0->2), total-group Cheloniidae (0->2); ACCTRAN: Protostegidae+++ (0->2), Dermochelyidae (2->0).

**Character 141 (Evers & Benson [2018]: character 139).** Parabasisphenoid, dorsum sellae: 0 = deep, i.e. the dorsal surface of parabasisphenoid between the clinoid processes is a transversely concave floor forming a trough between the posterior part of the dorsal surface of the parabasisphenoid, which is usually cup-like, and the anterior portion of the parabasisphenoid forming the rostrum basisphenoidale and sella turcica; 1 = low, i.e. the dorsum sellae is formed as a transverse ridge between the clinoid processes that projects anteriorly at a low angle from posterodorsal surface of the parabasisphenoid; 2 = high, i.e. a transverse ridge or wall of bone between the clinoid processes is present that projects dorsally at a high angle from the posteriorly positioned cup, separating the cup very clearly from of the anteriorly positioned rostrum basisphenoidale and sella turcica.

*Character optimisation*: Unambiguous: (*Kayentachelys*+*Eileanchelys*)+++ (2->1), total-group Chelonioidea (1->2), *Chelonia* *mydas* (2->0), *Macrochelys temminckii* (1->0), *Staurotypus* (1->0), *Dermatemys mawii* (1->0), *Platysternon megacephalum* (1->0), *Gopherus polyphemus* (1->0), Plesiochelyidae (1->2), Sandownidae (1->0), *Brachyopsemys* *tingitana* (0->2), *Meiolania planiceps* (1->0); DELTRAN: *Xinjiangchelys* *radiplicatoides* (1->2); ACCTRAN: (*Xinjiangchelys* *wusu* + *Xinjiangchelys* *radiplicatoides*) (1->2).

**Character 142 (Evers & Benson [2018]: character 140).** Parabasisphenoid, anterior surface of dorsum sellae: 0 = anterior surface of the dorsum sellae is flat and smooth; 1 = vertical median ridge on anterior surface of dorsum sellae between the clinoid processes is present, ridge may have a small anterodorsal projection.

*Character optimisation*: Unambiguous: Protostegidae+++ (0->1), *Plesiochelys planiceps* (0->1), *Plesiochelys etalloni* (0->1), *Kirgizemys dmitrievi* (0->1); DELTRAN: *Rhinochelys* *pulchriceps* (1->0), (*Puppigerus* + *Eochelone*) (1->0), *Cabindachelys* *landanensis* (1->0), *Kayentachelys* *aprix* (0->1), *Australochelys* *africanus* (0->1); ACCTRAN: *Australochelys*+++ (0->1), *Chubutemys*+++ (1->0), (*Rhinochelys* *pulchriceps* + *Rhinochelys* *nammourensis*) (1->0), (*Puppigerus* ++ *Ctenochelys*) (1->0), *Ctenochelys* (0->1).

**Character 143 (Evers & Benson [2018]: character 141).** Parabasisphenoid, retractor bulbi pits on anterolateral surface: 0 = absent; 1 = present.

*Character optimisation*: Unambiguous: Testudines (1->0), *Chelydra serpentina* (0->1), *Kinosternon suburum hippocrepis* (0->1), *Emys orbicularis* (0->1), Testudinidae (0->1), *Leyvachelys cipadi* (0->1), *Phrynops geoffroanus* (0->1), *Pelomedusa subrufa* (0->1); DELTRAN: *Australochelys*+++ (0->1); ACCTRAN: *Australochelys*+++ (0->1).

**Character 144 (Evers & Benson [2018]: character 142).** Parabasisphenoid, foramina anterius canalis carotici cerebralis: 0 = widely separated; 1 = close together; 2 = right and left cerebral arteries converge within the parabasisphenoid and run within a short joint canal, and exit anteriorly into the cavum cranii via a single foramen within the sella turcica.

*Character optimisation*: Unambiguous: Sinemydidae/Macroabenidae+++ (1->0), *Chelosphargis* advena (2->1), *Ocepechelon bouyai* (2->1), *Natator* *depressus* (1->2), *Macrochelys temminckii* (0->1), *Baptemys wyomingensis* (0->1), *Platysternon megacephalum* (0->1), *Adocus beatus* (0->1), *Plesiochelys* *bigleri* (1->0), *Meiolania planiceps* (1->0); DELTRAN: Protostegidae+++ (0->2), crown-group Chelonioidea (2->1), *Lepidochelys* *olivacea* (1->2), *Caretta Caretta* (1->2), *Toxochelys* sp. (0->1), Thalassochelydia (0->1), *Leyvachelys cipadi* (0->1); ACCTRAN: total-group Chelonioidea (0->1), Protostegidae+++ (1->2), crown-group Chelonioidea (2->1), (*Caretta* ++ *Lepidochelys*) (1->2), *Lepidochelys* *kempii* (2->1), Angolachelonia (0->1), (*Brachyopsemys* ++ *Sandownia*) (1->0).

**Character 145 (Evers & Benson [2018]: character 143).** Parabasisphenoid, prootic foramen in primary lateral wall of the braincase: 0 = present, the clinoid process of the parabasisphenoid and parts of the prootic, an ossified pila antotica, the laterosphenoid, and/or parts of the parietal form a foramen medially to the cavum epiptericum; 1 = the prootic foramen is reduced, and the clinoid processes are free structures.

*Character optimisation*: Unambiguous: *Plesiochelys etalloni* (1->0), *Pelomedusa subrufa* (1->0), *Annemys* sp. IVPP V18106 (1->0); DELTRAN: *Australochelys*+++ (0->1); ACCTRAN: *Australochelys*+++ (0->1).

**Character 146 (Evers & Benson [2018]: character 144).** Parabasisphenoid, crista tuberculi basalis developed on posterior part of dorsal surface: 0 = absent, parabasisphenoid dorsal surface is relatively flat or gently concavely excavated; 1 = present as median, dorsally high projecting tubercle or ridge.

*Character optimisation*: Unambiguous: crown-group Chelonioidea (0->1), (*Lepidochelys* *olivacea* + *Lepidochelys* *kempii*) (1->0), *Puppigerus camperi* (1->0), *Toxochelys* sp. (0->1), *Emys orbicularis* (0->1), *Kallokibotion bajazidi* (0->1).

**Character 147 (Evers & Benson [2018]: character 145).** Carotid artery/Pterygoid, interpterygoid vacuity: 0 = large opening, triangular in shape; 1 = reduced to an interpterygoid slit; 2 = entirely closed, no foramina posterius canalis carotici palatinum present. Scored inapplicable for taxa with a bony canal for the palatine artery (ch 146.1)

*Character optimisation*: Unambiguous: *Chubutemys*+++ (0->1), (*Annemys* *latiens* ++ *Xinjiangchelys*) (1->0), Paracryptodira (1->2).

**Character 148 (Evers & Benson [2018]: character 146).** Embedding of the palatine division of carotid artery: 0 = the palatine artery is not encased in a bony canal (artery enters the skull through the interpterygoid cavuity, interpterygoid slit, or possibly other skull openings); 1 = the palatine artery is encased in a bony canal (irrespective of the exposure or embedding of the internal carotid artery split).

*Character optimisation*: Unambiguous: Xinjiangchelyidae+++ (0->1), *Dracochelys bicuspis* (1->0), *Kallokibotion bajazidi* (0->1).

**Character 149 (Evers & Benson [2018]: character 147).** Embedding of internal carotid artery and its bifurcation: 0 = Internal carotid arterial system is not embedded by bone, a foramen posterius canalis carotici is absent, and the split into palatine and cerebral artery occurs extracranially; 1 = internal carotid arterial system is partially embedded, a foramen posterius canalis carotici interni is present, and the split into palatine and cerebral artery happens at the fenestra caroticus and is thus ventrally exposed; 2 = internal carotid arterial system is ventrally fully embedded by bone, a foramen posterius canalis carotici interni is present and the split into palatine and cerebral artery is ventrally covered by bone.

*Character optimisation*: Unambiguous: Testudines (1->2), *Adocus beatus* (2->1), *Jurassichelon* *oleronensis* (2->1), *Plesiochelys* *bigleri* (2->1), *Sandownia* *harrisi* (2->1); DELTRAN: Xinjiangchelyidae+++ (0->1), *Meiolania planiceps* (0->1); ACCTRAN: *Meiolania*+++ (0->1), Paracryptodira (1->0).

**Character 150 (Evers & Benson [2018]: character 148).** Position of the foramen posterius canalis carotici interni (fpcci): 0 = the fpcci is located at the ventral surface of the skull in a position far anterior to the margin of the fenestra postotica; 1 = the fpcci is located at the posterior end of the skull, either on the ventral surface of the skull close to the margin of the fenestra postotica, or on the posterior surface of the skull at the ventral margin of the fenestra postotica. This character is scored inapplicable for taxa that lack a foramen posterius canalis carotici interni.

*Character optimisation*: Unambiguous: *Santanachelys* *gaffneyi* (1->0), Trionychidae (1->0), *Chelus fimbriatus* (1->0); DELTRAN: *Protostega* *gigas* (1->0), *Podocnemis* (1->0), *Galianemys whitei* (1->0); ACCTRAN: (*Protostega* + *Archelon*) (1->0), total-group Pelomedusoides (1->0), *Pelomedusa subrufa* (0->1).

**Character 151 (Evers & Benson [2018]: character 149).** Entry of the internal carotid artery into skull relative to cavum acustico-jugulare: 0 = internal carotid enters the skull ventrally to the level of the cavum acustico-jugulare; 1 = internal carotid artery enters the skull within the cavum acustico-jugulare, artery lies in dorsally open trough on dorsal surface of pterygoid and only becomes dorsally covered within the cavum acustico-jugulare.

*Character optimisation*: Unambiguous: Dermochelyidae (0->1), *Puppigerus camperi* (0->1), *Argillochelys cuneiceps* (0->1), *Macrochelys temminckii* (1->0), *Baptemys wyomingensis* (1->0), (*Kirgizemys* *hoburensis* + *Kirgizemys dmitrievi*) (0->1); DELTRAN: Chelydroidea (0->1), Testudinoidea (0->1), *Meiolania planiceps* (0->1); ACCTRAN: Durocryptodira (0->1), total-group Chelonioidea (1->0), *Meiolania planiceps* (0->1).

**Character 152 (Evers & Benson [2018]: character 150).** Dorsal exposure of the palatine artery and/or anterior parts of the internal carotid artery: 0 = absent, the split of the internal carotid artery is dorsally covered by bone, and the palatine artery exits the basicranium into the cavum cranii via the foramen anterius canalis carotici palatinum; 1 = the internal carotid artery exits the braincase anterodorsally into the sulcus cavernosus where it continues on the floor of the sulcus cavernosus (= sulcus caroticus of Gaffney 1979), and the split into cerebral and palatine arteries occurs within the sulcus cavernosus. The cerebral artery enters the parabasisphenoid medially, whereas the palatine artery continues in the sulcus cavernosus anteriorly without ever being embedded in a canal. This character is scored as inapplicable when the carotid split is ventrally exposed (i.e. ch 148.0 or 148.1).

*Character optimisation*: Unambiguous: crown-group Cheloniidae (0->1), *Nichollsemys baieri* (0->1).

**Character 153 (Evers & Benson [2018]: character 151).** Formation of the foramen posterius canalis carotici interni, pterygoid involvement: 0 = present; 1 = absent.

*Character optimisation*: Unambiguous: total-group Chelidae (0->1), *Pelomedusa subrufa* (0->1).

**Character 154 (Evers & Benson [2018]: character 152).** Formation of the foramen posterius canalis carotici interni, parabasisphenoid involvement: 0 = present; 1 = absent.

*Character optimisation*: Unambiguous: (*Calcarichelys* + *Rhinochelys*) (1->0), *Desmatochelys* *padillai* (1->0), Plesiochelyidae (1->0), *Galianemys whitei* (1->0); DELTRAN: *Protostega* *gigas* (1->0), *Ordosemys* sp. IVPP V12092 (1->0), *Annemys* sp. IVPP V18106 (1->0), *Xinjiangchelys* *radiplicatoides* (1->0); ACCTRAN: (*Protostega* + *Archelon*) (1->0), (*Ordosemys* + *Dracochelys*) (1->0), Xinjiangchelyidae (1->0), *Xinjiangchelys* *wusu* (0->1).

**Character 155 (Evers & Benson [2018]: character 153)**. Formation of the foramen posterius canalis carotici interni, quadrate involvement: 0 = present; 1 = absent.

*Remarks*: See remarks for character 151.

*Character optimisation*: Unambiguous: *Podocnemis* (1->0); DELTRAN: *Phrynops geoffroanus* (1->0), *Chelodina* (1->0), *Araripemys barretoi* (1->0); ACCTRAN: total-group Chelidae (1->0), *Chelus fimbriatus* (0->1), *Elseya dentata* (0->1).

**Character 156 (Evers & Benson [2018]: character 154).** Formation of the foramen posterius canalis carotici interni, prootic involvement: 0 = present; 1 = absent.

*Character optimisation*: Unambiguous: *Argillochelys cuneiceps* (1->0), Chelydroidea (1->0), *Macrochelys temminckii* (0->1), *Baptemys wyomingensis* (0->1), Emydidae (1->0), Testudinidae (1->0), total-group Chelidae (1->0), *Pelomedusa subrufa* (1->0); DELTRAN: *Kirgizemys* *hoburensis* (1->0); ACCTRAN: (*Kirgizemys* *hoburensis* + *Kirgizemys dmitrievi*) (1->0).

**Character 157 (Evers & Benson [2018]: character 155).** Hyomandibular branch of the facial nerve: 0 = contained within the canalis cavernosus; 1 = contained in a sulcus or separate canal paralleling the canalis cavernosus.

*Character optimisation*: Unambiguous: *Corsochelys* *halinches* (0->1), Testuguria (0->1), Plesiochelyidae (0->1), *Portlandemys mcdowelli* (1->0), *Annemys* sp. IVPP V18106 (0->1); DELTRAN: *Meiolania planiceps* (0->1), *Eileanchelys* *waldmanni* (0->1); ACCTRAN: *Meiolania planiceps* (0->1), (*Kayentachelys* + *Eileanchelys*) (0->1).

**Character 158 (Evers & Benson [2018]: character 156).** Stapedial artery, foramen stapedio-temporale: 0 = present; 1 = absent.

*Character optimisation*: Unambiguous: total-group Dermatemydidae (0->1).

**Character 159 (Evers & Benson [2018]: character 157).** Stapedial artery, size of foramen stapedio-temporale: 0 = relatively large (the size of a large blood foramina, ≥5 mm diameter); 1 = significantly reduced in size (the size of a nerve foramina, ≤3 mm diameter. This character is scored as inapplicable for taxa without a foramen stepedio-temporale (i.e. ch 156.1).

*Character optimisation*: DELTRAN: Kinosternidae (0->1); ACCTRAN: Kinosternoidea (0->1).

**Character 160 (Evers & Benson [2018]: character 158).** Stapedial artery, foramen stapedio-temporale location in the otic chamber: 0 = on dorsal part and pointing dorsally; 1 = on the anterior wall of the otic region, pointing anteriorly. This character is scored as inapplicable for taxa without a foramen stepedio-temporale (i.e. ch 156.1).

*Character optimisation*: Unambiguous: crown-group Pleurodira (0->1), *Podocnemis* (1->0).

**Character 161 (Evers & Benson [2018]: character 159).** Stapedial artery, formation of the foramen stapedio-temporale, contribution of the quadrate: 0 = absent; 1 = present. Scored inapplicable for taxa without a foramen stapedio-temporale. This character is scored as inapplicable for taxa without a foramen stepedio-temporale (i.e. ch 156.1).

*Character optimisation*: Unambiguous: *Kinosternon suburum hippocrepis* (1->0), *Platysternon megacephalum* (1->0), *Chelus fimbriatus* (1->0), *Meiolania planiceps* (1->0).

**Character 162 (Evers & Benson [2018]: character 160).** Stapedial artery, formation of the foramen stapedio-temporale, contribution of the prootic: 0 = absent; 1 = present. Scored inapplicable for taxa without a foramen stapedio-temporale. This character is scored as inapplicable for taxa without a foramen stepedio-temporale (i.e. ch 156.1).

*Character optimisation*: DELTRAN: (*Kayentachelys*+*Eileanchelys*)+++ (0->1); ACCTRAN: *Australochelys*+++ (0->1).

**Character 163 (Evers & Benson [2018]: character 161).** Stapedial artery, formation of the foramen stapedio-temporale, contribution of the opisthotic: 0 = absent; 1 = present. Scored inapplicable for taxa without a foramen stapedio-temporale. This character is scored as inapplicable for taxa without a foramen stepedio-temporale (i.e. ch 156.1).

*Character optimisation*: Unambiguous: Protostegidae+++ (0->1), (*Calcarichelys* + *Rhinochelys*) (1->0), total-group Cheloniidae (1->0), *Caretta Caretta* (0->1), *Ctenochelys* (0->1), *Chelonoidis* sp. (0->1), *Brachyopsemys* *tingitana* (0->1), *Kirgizemys dmitrievi* (0->1), *Meiolania planiceps* (0->1); DELTRAN: *Proganochelys quenstedti* (0->1); ACCTRAN: *Proganochelys quenstedti* (0->1).

**Character 164 (Evers & Benson [2018]: character 162).** Stapedial artery, formation of the foramen stapedio-temporale, contribution of the supraoccipital: 0 = absent; 1 = present. Scored inapplicable for taxa without a foramen stapedio-temporale. This character is scored as inapplicable for taxa without a foramen stepedio-temporale (i.e. ch 156.1).

*Character optimisation*: Unambiguous: *Ctenochelys* (0->1), *Eubaena cephalica* (0->1).

**Character 165 (Evers & Benson [2018]: character 163).** Foramen jugulare posterius, relationship with the fenestra postotica: 0 = separate from fenestra postotica; 1 = coalescent with fenestra postotica.

*Character optimisation*: Unambiguous: Cryptodira (1->0), total-group Chelonioidea (0->1), *Allopleuron hoffmanni* (1->0), *Platysternon megacephalum* (0->1), *Brachyopsemys* *tingitana* (1->0), Chelinae (1->0), crown-group Pelomedusoides (1->0), *Kirgizemys* *hoburensis* (1->0); DELTRAN: (*Apalone* + *Petrochelys*) (0->1); ACCTRAN: Trionychinae (0->1).

**Character 166 (Evers & Benson [2018]: character 164).** Foramen jugulare posterius, formation of lateral bar separating foramen from fenestra postotica: 0 = formed by pterygoid; 1 = formed by opisthotic and/or exoccipital. The character is scored inapplicable for taxa in which the foramen jugulare posterius is coalescent with the fenestra postotica (ch. 163.1).

*Character optimisation*: Unambiguous: *Lissemys punctata* (1->0).

**Character 167 (Evers & Benson [2018]: character 165).** Recessus scalae tympani: 0 = almost nonexistent, not surrounded by bone; 1 = well developed.

*Character optimisation*: DELTRAN: *Chubutemys*+++ (0->1), *Eileanchelys* *waldmanni* (0->1); ACCTRAN: (*Kayentachelys*+*Eileanchelys*)+++ (0->1), *Kayentachelys* *aprix* (1->0

**Character 168 (Evers & Benson [2018]: character 166).** Cranial scutes, scute D meeting in midline: 0 = absent; 1 = present.

*Character optimisation*: DELTRAN: *Meiolania planiceps* (0->1); ACCTRAN: *Meiolania planiceps* (0->1).

**Character 169 (Evers & Benson [2018]: character 167).** Cranial scutes, scute X much smaller than scute D: 0 = absent; 1 = present.

*Character optimisation*: -.

**Character 170 (Evers & Benson [2018]: character 168).** Cranial scutes, scute X partially separates scutes G: 0 = absent; 1 = present.

*Character optimisation*: -.

**Character 171 (Evers & Benson [2018]: character 169).** Cranial scutes, scutes A, B, and C forming a continuous posterolateral shelf: 0 = absent; 1 = present.

*Character optimisation*: -.

**Character 172 (Evers & Benson [2018]: character 170).** Cranial scutes, scute F: 0 = formed by several scutes; 1 = formed by a single scute.

*Character optimisation*: DELTRAN: *Meiolania planiceps* (0->1); ACCTRAN: *Meiolania planiceps* (0->1).

**Character 173 (Evers & Benson [2018]: character 171).** Cranial scutes, scute J: 0 = formed by several scutes; 1 = formed by a single scute.

*Character optimisation*: -.

**Character 174 (Evers & Benson [2018]: character 172).** Dentary, medial contact of dentaries: 0 = fused; 1 = open suture.

*Character optimisation*: Unambiguous: *Toxochelys* sp. (0->1), *Baptemys wyomingensis* (0->1), total-group Chelidae (0->1), *Elseya dentata* (1->0), (*Kayentachelys* + *Eileanchelys*) (0->1).

**Character 175 (Evers & Benson [2018]: character 173).** Dentary, width triturating surface vs. jaw length: 0 = narrow triturating surface, symphysis less than 1/3 of jaw length; 1 = broad triturating surface, symphysis ≥1/3 jaw length.

*Character optimisation*: Unambiguous: *Lepidochelys* *olivacea* (0->1), *Puppigerus camperi* (0->1), (*Procolpochelys* ++ *Erquelinnesia*) (0->1); DELTRAN: *Solnhofia parsoni* (0->1), Sandownidae (0->1); ACCTRAN: Angolachelonia (0->1), Plesiochelyidae (1->0).

**Character 176 (Evers & Benson [2018]: character 174).** Dentary, symphyseal ridge: 0 = absent; 1 = present.

*Character optimisation*: Unambiguous: (*Bouliachelys* + *Rhinochelys*) (0->1), *Eochelone brabantica* (1->0), *Solnhofia parsoni* (0->1), *Elseya dentata* (0->1); DELTRAN: (*Lepidochelys* *olivacea* + *Lepidochelys* *kempii*) (0->1), (*Chelonia* + *Natator*) (0->1), (*Argillochelys* ++ *Puppigerus*) (0->1), crown-group Chelydridae (0->1); ACCTRAN: (*Puppigerus* ++ *Lepidochelys*) (0->1), (*Eretmochelys* ++ *Lepidochelys*) (1->0), (*Lepidochelys* *olivacea* + *Lepidochelys* *kempii*) (0->1), total-group Chelydridae (0->1).

**Character 177 (Evers & Benson [2018]: character 175).** Dentary, lingual (tomial) ridge: 0 = prominent; 1 = weak or absent.

*Character optimisation*: Unambiguous: *Eretmochelys* *imbricata* (1->0), *Natator* *depressus* (1->0), *Allopleuron hoffmanni* (1->0), *Sternotherus* (1->0), total-group Dermatemydidae (1->0), Testudinoidea (1->0), Plesiochelyidae (1->0), *Elseya dentata* (1->0), *Kallokibotion bajazidi* (0->1); DELTRAN: Sinemydidae/Macroabenidae+++ (0->1), *Carettochelys insculpta* (1->0), crown-group Pelomedusoides (1->0), *Xinjiangchelys* *radiplicatoides* (0->1); ACCTRAN: Xinjiangchelyidae+++ (0->1), total-group Carettochelyidae (1->0), total-group Pelomedusoides (1->0), *Annemys* *levensis* (1->0).

**Character 178 (Evers & Benson [2018]: character 176).** Dentary, size of foramen dentofaciale majus: 0 = small, size of a small vessel; 1 = enlarged, foramen is several mm in diameter.

*Character optimisation*: Unambiguous: total-group Dermatemydidae (0->1); DELTRAN: (*Erquelinnesia* ++ *Lepidochelys*) (0->1), *Chrysemys picta* (0->1), *Geoclemys hamiltonii* (0->1), *Gopherus polyphemus* (0->1), *Carettochelys insculpta* (0->1), *Adocus beatus* (0->1); ACCTRAN: Cryptodira (0->1), Americhelydia (1->0), total-group Cheloniidae (0->1), *Platysternon megacephalum* (1->0), (*Testudo* + *Chelonoidis*) (1->0), Trionychidae (1->0).

**Character 179 (Evers & Benson [2018]: character 177).** Dentary-Surangular arrangement: 0 = lack of a posterior expansion of dentary and anterior projection of surangular; 1 = posterior expansion of dentary present almost reaching the articular surface, covering the dorsal half of the surangular in lateral view, surangular with anterior projection.

*Character optimisation*: Unambiguous: *Lepidochelys* *kempii* (1->0); DELTRAN: *Bouliachelys suteri* (0->1), *Dermochelys coriacea* (0->1), crown-group Cheloniidae (0->1), *Ctenochelys* (0->1), *Procolpochelys charlestonensis* (0->1), *Toxochelys* sp. (0->1), crown-group Chelydridae (0->1); ACCTRAN: Americhelydia (0->1), Protostegidae (1->0), *Bouliachelys suteri* (0->1), (*Argillochelys* ++ *Puppigerus*) (1->0), (*Ctenochelys* ++ *Peritresius*) (0->1), (*Oligochelone* + *Erquelinnesia*) (1->0), Kinosternoidea (1->0).

**Character 180 (Evers & Benson [2018]: character 178).** Surangular, with anteromedial process forming a vertical lamina that projects anteriorly into the fossa meckelii: 0 = absent; 1 = present.

*Character optimisation*: Unambiguous: (*Chelonia* + *Natator*) (1->0); DELTRAN: Cryptodira (0->1), Testudinidae (1->0), Trionychinae (1->0), *Solnhofia parsoni* (0->1), *Plesiochelys planiceps* (0->1); ACCTRAN: Xinjiangchelyidae+++ (0->1), Testuguria (1->0), Trionychidae (1->0), (*Plesiochelys etalloni* + *Plesiochelys* *bigleri*) (1->0), crown-group Pleurodira (1->0).

**Character 181 (Evers & Benson [2018]: character 179).** Coronoid, anteromedial process: 0 = absent; 1 = present.

*Character optimisation*: Unambiguous: Chelydroidea (1->0), Testuguria (1->0), *Chelodina* (1->0), *Araripemys barretoi* (1->0); DELTRAN: Testudines (0->1), Xinjiangchelyidae (0->1); ACCTRAN: Paracryptodira+++ (0->1), (*Judithemys* ++ *Sinemys*) (1->0).

**Character 182 (Evers & Benson [2018]: character 180).** Coronoid, contribution to triturating surface: 0 = absent; 1 = present.

*Character optimisation*: Unambiguous: Testudines (0->1), *Platysternon megacephalum* (1->0), (*Testudo* + *Chelonoidis*) (1->0), Plesiochelyidae (1->0), total-group Chelidae (1->0); DELTRAN: total-group Chelonioidea (1->0), crown-group Chelydridae (1->0), *Kinosternon suburum hippocrepisis* (1->0), *Baptemys wyomingensis* (1->0); ACCTRAN: Americhelydia (1->0), Kinosternoidea (0->1), *Kinosternon suburum hippocrepis* (1->0), *Baptemys wyomingensis* (1->0)

**Character 183 (Evers & Benson [2018]: character 181).** Coronoid, notch on posterior margin of coronoid: 0 = absent; 1 = present.

*Character optimisation*: Unambiguous: *Kinosternon suburum hippocrepis* (0->1), *Platysternon megacephalum* (0->1), Angolachelonia (0->1); DELTRAN: Trionychinae (0->1), *Carettochelys insculpta* (0->1), *Portlandemys mcdowelli* (1->0); ACCTRAN: crown-group Trionychia (0->1), *Lissemys punctata* (1->0), (*Jurassichelon* + *Portlandemys*) (1->0).

**Character 184 (Evers & Benson [2018]: character 182).** Coronoid, foramen at anterior end, leading from fossa meckelii into space between mandibular rami: 0 = absent; 1 = present.

*Character optimisation*: Unambiguous: *Natator* *depressus* (0->1), *Toxochelys* sp. (0->1), Angolachelonia (0->1), *Meiolania planiceps* (0->1); DELTRAN: *Lepidochelys* *olivacea* (0->1), *Caretta Caretta* (0->1), *Phrynops geoffroanus* (0->1), *Elseya dentata* (0->1); ACCTRAN: (*Caretta* ++ *Lepidochelys*) (0->1), *Lepidochelys* *kempii* (1->0), crown-group Chelidae (0->1), *Chelodina* (1->0).

**Character 185 (Evers & Benson [2018]: character 183).** Coronoid process, principally formed by: 0 = coronoid; 1 = dentary; 2 = surangular.

*Character optimisation*: Unambiguous: *Testudo* (0->1), *Elseya dentata* (0->1); DELTRAN: *Dermochelys coriacea* (0->2), Kinosternidae (0->1), *Dermatemys mawii* (0->1); ACCTRAN: Dermochelyidae (0->2), Kinosternoidea (0->1), *Baptemys wyomingensis* (1->0).

**Character 186 (Evers & Benson [2018]: character 184).** Coronoid process: 0 = relatively low, dorsally well rounded; 1 = relatively high, process is dorsally or posterodorsally pointed.

*Character optimisation*: Unambiguous: Kinosterninae (0->1), *Baptemys wyomingensis* (0->1), *Platysternon megacephalum* (0->1), total-group Trionychia (0->1), *Lissemys punctata* (1->0), Angolachelonia (0->1).

**Character 187 (Evers & Benson [2018]: character 185).** Splenial: 0 = present; 1 = absent.

*Character optimisation*: Unambiguous: Sinemydidae/Macroabenidae+++ (0->1), *Protostega* *gigas* (1->0), *Allopleuron hoffmanni* (1->0), Thalassochelydia (1->0), crown-group Chelidae (1->0), *Xinjiangchelys* *radiplicatoides* (0->1); DELTRAN: *Rhinochelys* *pulchriceps* (1->0); ACCTRAN: (*Santanachelys* ++ *Rhinochelys*) (1->0).

**POSTCRANIUM**

**Character 188 (Evers & Benson [2018]: character 186).** Carapace, carapacial scutes: 0 = present; 1 = absent.

*Character optimisation*: Unambiguous: Trionychidae (0->1); DELTRAN: (*Protostega* + *Archelon*) (0->1), Dermochelyidae (0->1), *Allopleuron hoffmanni* (0->1); ACCTRAN: (*Ocepechelon* ++ *Protostega*) (0->1), *Corsochelys*+++ (0->1), (*Erquelinnesia* ++ *Lepidochelys*) (1->0).

**Character 189 (Evers & Benson [2018]: character 187).** Carapace, carapacial scutes: 0 = present, ful9y covering the carapace; 1 = reduced not fully covering the carapace.

*Character optimisation*: DELTRAN: *Carettochelys insculpta* (0->1); ACCTRAN: crown-group Trionychia (0->1).

**Character 190 (Evers & Benson [2018]: character 188).** Carapace, continuous keel on costals: 0 = absent; 1 = present.

*Character optimisation*: Unambiguous: (*Ctenochelys* ++ *Peritresius*) (0->1), *Geoclemys hamiltonii* (0->1), *Chelus fimbriatus* (0->1), *Araripemys barretoi* (0->1); DELTRAN: *Emarginachelys cretacea* (0->1), Kinosternidae (0->1), *Baptemys wyomingensis* (0->1); ACCTRAN: Chelydroidea (0->1), crown-group Chelydridae (1->0), *Dermatemys mawii* (1->0).

**Character 191 (Evers & Benson [2018]: character 189).** Carapace, continuous keel on neurals: 0 = absent; 1 = present.

*Character optimisation*: Unambiguous: *Eosphargis breineri* (0->1), *Chelonia* *mydas* (0->1), (*Ctenochelys* ++ *Peritresius*) (0->1), *Allopleuron hoffmanni* (0->1), *Geoclemys hamiltonii* (0->1), *Chelus fimbriatus* (0->1), *Araripemys barretoi* (0->1); DELTRAN: (*Calcarichelys* + *Rhinochelys*) (0->1), *Santanachelys* *gaffneyi* (1->0), (*Protostega* + *Archelon*) (0->1), *Lepidochelys* *olivacea* (0->1), *Caretta Caretta* (0->1), *Emarginachelys cretacea* (0->1), Kinosternidae (0->1), *Baptemys wyomingensis* (0->1); ACCTRAN: Protostegidae (0->1), (*Santanachelys* + *Notochelone*) (1->0), (*Desmatochelys* *lowii* + *Desmatochelys* *padillai*) (1->0), (*Caretta* ++ *Lepidochelys*) (0->1), *Lepidochelys* *kempii* (1->0), Chelydroidea (0->1), crown-group Chelydridae (1->0), *Dermatemys mawii* (1->0).

**Character 192 (Evers & Benson [2018]: character 190)**. Shell, sculpturing of dorsal surface (carapace) and ventral surface (plastron): 0 = absent, smooth to slightly rugose; 1 = present, development of striations, vermiculations, striations, or pitting.

*Character optimisation*: Unambiguous: crown-group Trionychia (0->1), total-group Chelidae (0->1), *Phrynops geoffroanus* (1->0); DELTRAN: *Sinemys gamera* (0->1), Pleurosternidae (0->1); ACCTRAN: Sinemydidae (0->1), Paracryptodira (0->1).

**Character 193 (Evers & Benson [2018]: character 191).** Shell, pattern of sculpturing of the dorsal surface (carapace) and ventral surface (plastron): 0 = parallel to radial striations; 1 = vermiculation; 2 = highly dense pattern of pitting combined with striations; 3 = dichotomic striations; 4 = spread pitting without marked striation pattern; 5 = granules (positive relief).

*Character optimisation*: Unambiguous: crown-group Pleurodira (0->3), *Araripemys barretoi* (3->4), *Pleurosternon bullockii* (0->3); DELTRAN: Kinosternidae (0->1), crown-group Trionychia (0->2); ACCTRAN: Cryptodira (0->1), total-group Trionychia (1->2).

**Character 194 (Evers & Benson [2018]: character 192).** Carapacial Sutures: 0 = carapacial elements finely sutured or the contact is smooth; 1 = carapacial sutures strongly serrated in adult stage.

*Character optimisation*: DELTRAN: *Dracochelys bicuspis* (0->1); ACCTRAN: (*Ordosemys* + *Dracochelys*) (0->1).

**Character 195 (Evers & Benson [2018]: character 193).** Nuchal, articulation of nuchal with neural spine of eighth cervical vertebra: 0 = articulation via a blunt facet is present; 1 = articulation along a blunt facet absent.

*Character optimisation*: Unambiguous: *Meiolania*+++ (0->1), (*Ctenochelys* ++ *Peritresius*) (1->0); DELTRAN: *Carettochelys insculpta* (1->0); ACCTRAN: total-group Carettochelyidae (1->0).

**Character 196 (Evers & Benson [2018]: character 194).** Raised pedestal on the visceral surface of the nuchal for the articulation with the neural spine of the eighth cervical vertebra: 0 = absent; 1 = present.

*Character optimisation*: Unambiguous: total-group Chelonioidea (0->1), (*Desmatochelys* ++ *Protostega*) (1->0), *Allopleuron hoffmanni* (1->0).

**Character 197 (Evers & Benson [2018]: character 195).** Nuchal, elongate costiform process: 0 = absent; 1 = present.

*Character optimisation*: Unambiguous: Chelydroidea (0->1).

**Character 198 (Evers & Benson [2018]: character 196).** Nuchal, length of costiform process: 0 = crosses peripheral 1; 1 = reaches peripherals 2 or 3. This character is scored inapplicable for taxa without a costiform process on the nuchal (ch. 195.0).

*Character optimisation*: Unambiguous: *Chelydra serpentina* (1->2).

**Character 199 (Evers & Benson [2018]: character 197).** Nuchal, length versus width: 0 = wider than long; 1 = longer than wide or as long as wide.

*Character optimisation*: Unambiguous: *Chelonoidis* sp. (1->0), *Adocus beatus* (0->1), crown-group Pleurodira (0->1), *Procolpochelys charlestonensi*s (0->1); DELTRAN: Emydidae (0->1), Testuguria (0->1); ACCTRAN: Testudinoidea (0->1), *Platysternon megacephalum* (1->0).

**Character 200 (Evers & Benson [2018]: character 198).** Nuchal, posteriomedial fontanelles: 0 = absent; 1 = present.

*Character optimisation*: Unambiguous: *Desmatochelys* *lowii* (0->1), (*Ctenochelys* ++ *Peritresius*) (0->1), *Toxochelys* sp. (0->1).

**Character 201 (Evers & Benson [2018]: character 199).** Neurals, neural formula 6>4<6<6<6<6: 0 = absent; 1 = present.

*Character optimisation*: Unambiguous: *Adocus beatus* (0->1).

**Character 202 (Evers & Benson [2018]: character 200).** Neurals, shape of neurals: 0 = very irregular in shape, wider than long or squared; 1 = regular, often perfectly hexagonal or pentagonal, longer than wide.

*Character optimisation*: Unambiguous: *Platysternon megacephalum* (1->0), Testudinidae (1->0), *Chelus fimbriatus* (1->0); DELTRAN: *Kallokibotion*+++ (0->1), *Eosphargis breineri* (1->0), crown-group Chelydridae (1->0); ACCTRAN: *Chubutemys*+++ (0->1), Dermochelyidae (1->0), total-group Chelydridae (1->0).

**Character 203 (Evers & Benson [2018]: character 201).** Neurals: 0 = present; 1 = absent.

*Character optimisation*: Unambiguous: *Dermochelys coriacea* (0->1).

**Character 204 (Evers & Benson [2018]: character 202).** Neurals, number of neurals: 0 = ten or more; 1 = nine or less.

*Character optimisation*: Unambiguous: (*Lepidochelys* *olivacea* + *Lepidochelys* *kempii*) (1->0), *Procolpochelys* *charlestonensis* (1->0); DELTRAN: *Kallokibotion*+++ (0->1), *Eileanchelys* *waldmanni* (0->1); ACCTRAN: *Australochelys*+++ (0->1), *Kayentachelys* *aprix* (1->0).

**Character 205 (Evers & Benson [2018]: character 203).** Peripheral Gutter: 0 = peripheral gutter absent of only anteriorly developed; 1 = peripheral gutter extensively developed along anterior and bridge peripherals.

*Character optimisation*: Unambiguous: *Phrynops geoffroanus* (0->1), Xinjiangchelyidae (0->1), *Eileanchelys* *waldmanni* (0->1); DELTRAN: *Kirgizemys* *hoburensis* (0->1); ACCTRAN: (*Kirgizemys* *hoburensis* + *Kirgizemys dmitrievi*) (0->1).

**Character 206 (Evers & Benson [2018]: character 204).** Peripherals,: 0 = present; 1 = absent.

*Character optimisation*: Unambiguous: *Dermochelys coriacea* (0->1), Trionychidae (0->1).

**Character 207 (Evers & Benson [2018]: character 205).** Peripherals, number of peripherals: 0 = more than 11 pairs of peripherals present; 1 = 11 pairs of peripherals present; 2 = 10 pairs of peripherals present. This character is scored as inapplicable when peripherals are absent (ch. 204.1).

*Character optimisation*: Unambiguous: Kinosternidae (1->2); DELTRAN: (*Kayentachelys*+*Eileanchelys*)+++ (0->1), *Caretta Caretta* (1->0), *Carettochelys insculpta* (1->0); ACCTRAN: *Australochelys*+++ (0->1), (*Caretta* ++ *Lepidochelys*) (1->0), crown-group Trionychia (1->0).

**Character 208 (Evers & Benson [2018]: character 206).** Peripherals, anterior peripherals incised by musk ducts: 0 = absent; 1 = present.

*Character optimisation*: Unambiguous: Kinosternoidea (0->1), *Kinosternon suburum hippocrepis* (1->0), *Platysternon megacephalum* (0->1), *Geoclemys hamiltonii* (0->1), *Chelonoidis* sp. (0->1); DELTRAN: crown-group Chelidae (0->1), crown-group Pelomedusoides (0->1), (*Sinemys gamera* + *Sinemys lens*) (0->1), Xinjiangchelyidae (0->1), *Kayentachelys* *aprix* (0->1); ACCTRAN: Paracryptodira+++ (0->1), Testudines (1->0), crown-group Pleurodira (0->1), *Araripemys barretoi* (1->0), (*Kayentachelys* + *Eileanchelys*) (0->1).

**Character 209 (Evers & Benson [2018]: character 207).** Costals, medial contact of the first pair of costals: 0 = absent; 1 = present.

*Character optimisation*: Unambiguous: Kinosterninae (0->1), Chelodininae (0->1).

**Character 210 (Evers & Benson [2018]: character 208).** Costals, medial contact of posterior costals: 0 = absent; 1 = present.

*Character optimisation*: Unambiguous: *Chelus fimbriatus* (1->0), *Annemys* *levensis* (0->1); DELTRAN: *Desmatochelys* *lowii* (0->1), Kinosterninae (0->1), *Dermatemys mawii* (0->1), crown-group Trionychia (0->1), (*Plesiochelys etalloni* + *Plesiochelys* *bigleri*) (0->1), crown-group Pleurodira (0->1), *Proganochelys quenstedti* (0->1); ACCTRAN: Testudines (0->1), Durocryptodira (1->0), (*Desmatochelys* *lowii* + *Desmatochelys* *padillai*) (0->1), Kinosternoidea (0->1), *Baptemys wyomingensis* (1->0), *Solnhofia parsoni* (1->0), *Proganochelys quenstedti* (0->1).

**Character 211 (Evers & Benson [2018]: character 209).** Costals, number of costals involved in medial contact: 0 = medial contact of up to three posterior costals; 1 = medial contact of all costals. This character is scored as inapplicable for turtles without a medial contact of posterior costals.

*Character optimisation*: Unambiguous: *Sternotherus* (0->1), Chelodininae (0->1); DELTRAN: *Desmatochelys* *lowii* (0->1); ACCTRAN: total-group Chelonioidea (0->1).

**Character 212 (modified from Evers & Benson [2018]: character 210)**: Costals, lateral ossification: 0 = all costals fully ossified laterally with strong sutural contact with peripherals, lack of dorsal exposure of distal end of costal ribs and absence of costo-peripheral fontanelles; 1 = lateral sutural contact contact between costals and peripherals absent in at least parts of the costo-peripherial series, resulting in the presence of costo-peripheral fontanelles and/or the exposure of the distal rib ends.

*Character optimisation*: Unambiguous: total-group Chelonioidea (0->1), *Puppigerus camperi* (1->0), crown-group Chelydridae (0->1), Trionychinae (0->1), *Solnhofia parsoni* (0->1), *Araripemys barretoi* (0->1), Sinemydidae (0->1).

**Character 213 (modified from Evers & Benson [2018]: character 210)**: Costals, position of costo-peripheral fontanelles and exposure of dorsal rib ends: 0 = limited to parts of the carapace; 1 = fontanelles and exposed rib ends present and retained in adults between all costals and along the anterior margin of the first costal. Scored as inapplicable for taxa that lack costo-peripheral fontanelles (character 212.0).

*Character optimisation*: Unambiguous: *Procolpochelys* *charlestonensis* (1->0); DELTRAN: total-group Chelonioidea (0->1), *Rhinochelys* *nammourensis* (1->0), Trionychinae (0->1); ACCTRAN: Cryptodira (0->1), (*Rhinochelys* *pulchriceps* + *Rhinochelys* *nammourensis*) (1->0), Chelydroidea (1->0).

**Character 214 (new character)**: Costal, fontanelle along anterior margin of costal 1: 0 = anterior margin of first costal positioned very close to nuchal and/or anteriormost peripherals, reducing the fontanelle to an anteroposteriorly narrow, slot-like opening; 1 = extensive fontanelle between first costal and anterior margin of carapace. Scored inapplicable for taxa without costo-peripheral fontanelles (character 212.0) or when costo-peripheral fontanelles are absent along the first costal.

*Character optimisation*: Unambiguous: *Erquelinnesia gosseleti* (1->0); DELTRAN: Protostegidae (0->1), crown-group Chelonioidea (0->1); ACCTRAN: Protostegidae+++ (0->1), *Corsochelys* *halinches* (1->0).

**Character 215 (new character)**: Posterior costals, shape: 0 = rectangular, much wider mediolaterally than long anteroposteriorly; 1 = square or hexagonal, as wide as long.

*Character optimisation*: DELTRAN: (*Protostega* + *Archelon*) (0->1), *Eosphargis breineri* (0->1), *Allopleuron hoffmanni* (0->1); ACCTRAN: (*Ocepechelon* ++ *Protostega*) (0->1), crown-group Chelonioidea (0->1), (*Erquelinnesia* ++ *Lepidochelys*) (1->0).

**Character 216 (Evers & Benson [2018]: character 211).** Rib free peripherals: 0 = absent; 1 = present.

*Character optimisation*: Unambiguous: *Corsochelys*+++ (0->1), Thalassochelydia (0->1).

**Character 217 (Evers & Benson [2018]: character 212).** Position of rib free peripherals: 0 = only present anterior and posterior to ribs; 1 = present between sixth and seventh ribs. This character is scored as inapplicable for taxa that lack rib free peripherals (ch. 211.0).

*Character optimisation*: Unambiguous: crown-group Cheloniidae (2->1), *Chelonia* *mydas* (1->0); DELTRAN: (*Erquelinnesia* ++ *Lepidochelys*) (0->2), *Ctenochelys* (2->0); ACCTRAN: crown-group Chelonioidea (0->2), (*Ctenochelys* + *Cabindachelys*) (2->0).

**Character 218 (Evers & Benson [2018]: character 213).** Costals, alternative short and long ends in the lateral part of costals: 0 = absent; 1 = present.

*Character optimisation*: DELTRAN: *Testudo* (0->1), *Gopherus polyphemus* (0->1); ACCTRAN: Testudinidae (0->1), *Chelonoidis* sp. (1->0).

**Character 219 (Evers & Benson [2018]: character 214).** Costals, costal 9: 0 = present; 1 = absent.

*Character optimisation*: Unambiguous: (*Plesiochelys etalloni* + *Plesiochelys* *bigleri*) (1->0), *Kayentachelys* *aprix* (1->0); DELTRAN: *Rhinochelys* *nammourensis* (1->0), (*Protostega* + *Archelon*) (1->0); ACCTRAN: (*Rhinochelys* *pulchriceps* + *Rhinochelys* *nammourensis*) (1->0), (*Ocepechelon* ++ *Protostega*) (1->0).

**Character 220 (Evers & Benson [2018]: character 215).** Costals, shape of Costal 3: 0 = tapering towards the lateral side of the shell or with parallel anterior and posterior borders; 1 = broadens towards the lateral side of the shell.

*Character optimisation*: DELTRAN: *Dracochelys bicuspis* (0->1); ACCTRAN: (*Ordosemys* + *Dracochelys*) (0->1).

**Character 221 (Evers & Benson [2018]: character 216).** Suprapygals: 0 = present; 1 = absent.

*Character optimisation*: Unambiguous: Trionychidae (0->1).

**Character 222 (Evers & Benson [2018]: character 217).** Suprapygals, number of suprapygals: 0 = one; 1 = two; 2 = more than two.

*Character optimisation*: Unambiguous: Protostegidae (1->0), *Procolpochelys* *charlestonensis* (1->0), Chelydroidea (1->0), *Chelydra serpentina* (0->2), Emydidae (1->0), *Solnhofia parsoni* (1->2), crown-group Pleurodira (1->0), *Pleurosternon bullockii* (1->0), *Kayentachelys* *aprix* (1->0); DELTRAN: *Carettochelys insculpta* (1->0); ACCTRAN: total-group Trionychia (1->0).

**Character 223 (Evers & Benson [2018]: character 218).** Suprapygals, size between suprapygal 1 and 2: 0 = suprapygal 1 smaller than suprapygal 2; 1 = suprapygal 1 larger. Reworded from KL (ch 88). Turtles with only one suprapygal or suprapygals absent are coding as inapplicable.

*Character optimisation*: Unambiguous: *Caretta Caretta* (1->0), *Puppigerus camperi* (1->0), *Kallokibotion bajazidi* (0->1); DELTRAN: total-group Chelonioidea (0->1); ACCTRAN: Americhelydia (0->1).

**Character 224 (Evers & Benson [2018]: character 219).** Cervical scutes: 0 = present; 1 = cervical scutes absent.

*Character optimisation*: Unambiguous: Protostegidae+++ (0->1), (*Desmatochelys* *lowii* + *Desmatochelys* *padillai*) (1->0), (*Erquelinnesia* ++ *Lepidochelys*) (1->0), crown-group Trionychia (0->1), Sinemydidae (0->1), *Pleurosternon bullockii* (0->1); DELTRAN: *Elseya dentata* (0->1), *Araripemys barretoi* (0->1), crown-group Pelomedusoides (0->1); ACCTRAN: crown-group Pleurodira (0->1), crown-group Chelidae (1->0), *Elseya dentata* (0->1).

**Character 225 (Evers & Benson [2018]: character 220).** Number of cervical scutes: 0 = more than one cervical scute present; 1 = one cervical scute present. This character is scored as inapplicable when cervical scutes are absent (ch. 219.1)

*Character optimisation*: DELTRAN: (*Plesiochelys etalloni* + *Plesiochelys* *bigleri*) (1->0); ACCTRAN: Plesiochelyidae (1->0).

**Character 226 (Evers & Benson [2018]: character 221).** Pygal, posterior notch: 0 = present; 1 = absent.

*Character optimisation*: Unambiguous: *Eretmochelys* *imbricata* (1->0), *Natator* *depressus* (1->0), (*Ctenochelys* ++ *Peritresius*) (1->0), *Allopleuron hoffmanni* (1->0), Emysternia (1->0), *Chelonoidis* sp. (1->0), *Chelus fimbriatus* (1->0), *Meiolania planiceps* (1->0); DELTRAN: (*Kayentachelys*+*Eileanchelys*)+++ (0->1), (*Chelosphargis* + *Rhinochelys*) (1->0), crown-group Chelydridae (1->0), *Dracochelys bicuspis* (1->0); ACCTRAN: *Australochelys*+++ (0->1), (*Bouliachelys* + *Rhinochelys*) (1->0), total-group Chelydridae (1->0), Sinemydidae (1->0).

**Character 227 (Evers & Benson [2018]: character 222).** Supramarginals: 0 = present; 1 = absent.

*Character optimisation*: Unambiguous: *Macrochelys temminckii* (1->0); DELTRAN: (*Kayentachelys*+*Eileanchelys*)+++ (0->1); ACCTRAN: *Australochelys*+++ (0->1).

**Character 228 (Evers & Benson [2018]: character 223).** Supramarginals, separating marginal and pleurals: 0 = complete row present, fully separating marginals from pleurals; 1 = partial row present, incompletely separating marginals from pleurals. This character is scored as inapplicable for turtles in which supramarginals are absent.

*Character optimisation*: -.

**Character 229 (Evers & Benson [2018]: character 224).** Vertebrals, shape of the verterbrals: 0 = vertebrals 2 to 4 signicantly broader than pleurals; 1 = vertebrals 2 to 4 as narrow as, or narrower than, pleurals.

*Character optimisation*: Unambiguous: Xinjiangchelyidae+++ (0->1), Thalassochelydia (1->0); DELTRAN: *Santanachelys* *gaffneyi* (1->0); ACCTRAN: (*Santanachelys* + *Notochelone*) (1->0).

**Character 230 (Evers & Benson [2018]: character 225).** Vertebrals, position of vertebral 3-4 sulcus in taxa with five vertebrals: 0 = sulcus positioned on neural 6; 1 = sulcus positioned on neural 5.

*Character optimisation*: Unambiguous: *Peritresius* *martini* (1->0), *Erquelinnesia gosseleti* (1->0), *Annemys* *levensis* (1->0); DELTRAN: Paracryptodira+++ (0->1), *Santanachelys* *gaffneyi* (1->0); ACCTRAN: *Meiolania*+++ (0->1), (*Santanachelys* + *Notochelone*) (1->0).

**Character 231 (Evers & Benson [2018]: character 226).** Vertebrals, vertebral 3-4 sulcus with a wide posteriorly oriented medial embayment: 0 = absent; 1 = present.

*Character optimisation*: DELTRAN: *Annemys* *levensis* (0->1), *Annemys* *latiens* (0->1); ACCTRAN: Xinjiangchelyidae (0->1), (*Annemys* sp. IVPP V18106 ++ *Xinjiangchelys*) (1->0).

**Character 232 (Evers & Benson [2018]: character 227).** Vertebrals, verebral 1: 0 = vertebral 1 does not enter anterior margin of carapace; 1 = enters anterior margin.

*Character optimisation*: Unambiguous: *Araripemys barretoi* (0->1), Sinemydidae (0->1).

**Character 233 (Evers & Benson [2018]: character 228).** Marginals, marginal scutes overlap onto costals: 0 = absent, marginals restricted to peripherals; 1 = present.

*Character optimisation*: Unambiguous: *Meiolania*+++ (0->1), Sinemydidae/Macroabenidae+++ (1->0); DELTRAN: *Adocus beatus* (0->1); ACCTRAN: total-group Trionychia (0->1).

**Character 234 (Evers & Benson [2018]: character 229).** Pleurals, at least one pair of additional pleural scutes located laterally of vertebral scute 1, with anterior contact with cervical scute: 0 = absent; 1 = present.

*Character optimisation*: Unambiguous: (*Caretta* ++ *Lepidochelys*) (0->1).

**Character 235 (Evers & Benson [2018]: character 230).** Plastron, connection between carapace and plastron: 0 = osseous; 1 = ligamentous.

*Character optimisation*: Unambiguous: Kinosternoidea (1->0), *Chrysemys picta* (1->0), Testuguria (1->0), *Chelonoidis* sp. (0->1), *Adocus beatus* (1->0), total-group Pleurodira (1->0), *Solnhofia parsoni* (0->1), *Araripemys barretoi* (0->1); DELTRAN: (*Kayentachelys*+*Eileanchelys*)+++ (0->1), Pleurosternidae (1->0); ACCTRAN: *Australochelys*+++ (0->1), Paracryptodira (1->0).

**Character 236 (Evers & Benson [2018]: character 231).** Plastron, central plastral fontanelle: 0 = absent; 1 = present.

*Character optimisation*: Unambiguous: total-group Chelonioidea (0->1), crown-group Chelydridae (0->1), Trionychidae (0->1), *Araripemys barretoi* (0->1), *Meiolania planiceps* (0->1); DELTRAN: Thalassochelydia (0->1), *Dracochelys bicuspis* (0->1); ACCTRAN: Angolachelonia (0->1), Sinemydidae (0->1).

**Character 237 (Evers & Benson [2018]: character 232).** Plastron, posterior plastral fontanelle, posterior plastral fontanelle between the xiphiplastra and/or the hypoplastra: 0 = absent in adult stage; 1 = retained in adult stage.

*Character optimisation*: Unambiguous: (*Puppigerus* + *Eochelone*) (1->0), Trionychidae (0->1), *Araripemys barretoi* (0->1); DELTRAN: Protostegidae+++ (0->1), *Santanachelys* *gaffneyi* (1->0); ACCTRAN: total-group Chelonioidea (0->1), (*Santanachelys* + *Notochelone*) (1->0).

**Character 238 (Evers & Benson [2018]: character 233).** Plastron, plastral kinesis: 0 = absent, scutes sulci and bony sutures do not overlap; 1 = present, scutes sulci coincide with epiplastral-hyoplastral contact.

*Character optimisation*: Unambiguous: Kinosternidae (0->1), *Emys orbicularis* (0->1).

**Character 239 (Evers & Benson [2018]: character 234).** Plastron, plastral kinesis: 0 = between hyoplastron and hypoplastron; 1 = between hyoplastron and epiplastron- entoplastron.

*Character optimisation*: DELTRAN: Kinosternidae (0->1); ACCTRAN: Chelydroidea (0->1).

**Character 240 (Evers & Benson [2018]: character 235).** Plastron, hyo-hypoplastra contact: 0 = contact between hyo-hyoplastra absent or reduced; 1 = extensive contact between hyo-hyoplastra (even for those taxa with plastral kinesis).

*Character optimisation*: Unambiguous: Xinjiangchelyidae+++ (0->1), total-group Chelonioidea (1->0); DELTRAN: *Araripemys barretoi* (1->0), crown-group Pelomedusoides (1->0); ACCTRAN: crown-group Pleurodira (1->0), crown-group Chelidae (0->1).

**Character 241 (modified from Evers & Benson [2018]: character 236)**: Plastron, hyo-hypoplastra serrations: 0 = serrations on the lateral and medial margins absent or weakly developed; 1 = strong serrations present along the surfaces that face other bones, but serrations are absent along the margin of the central fontanelle and the lateral contact area of hyo- and hypoplastro; 2 = strong serrations along all margins but the anterolateral margin of the hyoplastra and the posterlateral margin of the hypoplastra present, giving these elements a ‘star-shaped’ appearance.

*Character optimisation*: Unambiguous: total-group Chelonioidea (0->1), (*Desmatochelys* ++ *Protostega*) (1->2), *Dermochelys coriacea* (1->0), (*Puppigerus* + *Eochelone*) (1->0).

**Character 242 (Evers & Benson [2018]: character 237).** Axillar and inguinal notches: 0 = deep U or V-shaped axillar and inguinal notches; 1 = very shallow axillar and inguinal notches, and long lateral edges.

*Character optimisation*: DELTRAN: Protostegidae (0->1), Dermochelyidae (0->1); ACCTRAN: Protostegidae+++ (0->1), total-group Cheloniidae (1->0).

**Character 243 (Evers & Benson [2018]: character 238).** Entoplastron: 0 = present; 1 = absent.

*Character optimisation*: Unambiguous: Kinosterninae (0->1); DELTRAN: *Dermochelys coriacea* (0->1); ACCTRAN: Dermochelyidae (0->1).

**Character 244 (Evers & Benson [2018]: character 239).** Entoplastron, anterior entoplastral process: 0= present, medial contact of epiplastra absent; 1 = absent, medial contact of epiplastra present.

*Character optimisation*: DELTRAN: *Kallokibotion*+++ (0->1), *Eileanchelys* *waldmanni* (0->1); ACCTRAN: *Australochelys*+++ (0->1), *Kayentachelys* *aprix* (1->0).

**Character 245 (Evers & Benson [2018]: character 241).** Entoplastron, distinct posterolateral process: 0 = present; 1 = absent.

*Character optimisation*: DELTRAN: (*Kayentachelys*+*Eileanchelys*)+++ (0->1); ACCTRAN: *Australochelys*+++ (0->1).

**Character 246 (modified from Evers & Benson [2018]: characters 240 & 242):** Entoplastron, shape of the entoplastron: 0 = ‘dagger-shaped’, with dorsoventrally thick anterior end and long posterior process that extends along the dorsal surface of the plastron and sometimes reaches the mesoplastra; 1 = plate like and diamond-shaped or hexagonal in ventral view, with all margins of subequal length; 2 = T-shaped or triangular, i.e. entoplastron has a mediolaterally expanded anterior end and a progressively narrowing posterior process; 3 = strap like and V-shaped, with posterolateral processes diverging from the midline of the plastron.

*Character optimisation*: Unambiguous: total-group Chelonioidea (1->2), crown-group Chelydridae (1->2), Trionychidae (1->3), (*Judithemys* ++ *Sinemys*) (1->2); DELTRAN: Paracryptodira+++ (0->1), *Kallokibotion bajazidi* (0->1); ACCTRAN: *Chubutemys*+++ (0->1), *Meiolania planiceps* (1->0).

**Character 247 (new character):** Entepiplastron: 0 = absent, entoplastron and epiplastra are separate elements; 1 = present, entoplastron is fused with epiplastra, resulting in a laterally extremely expanded entepiplastron wings.

*Character optimisation*: Unambiguous: *Calcarichelys gemma* (0->1); DELTRAN: (*Protostega* + *Archelon*) (0->1); ACCTRAN: (*Ocepechelon* ++ *Protostega*) (0->1).

**Character 248 (Evers & Benson [2018]: character 243).** Entoplastron, suture with hyoplastra: 0 = tightly sutured; 1 = lightly sutured to almost absent contact between both.

*Character optimisation*: Unambiguous: Americhelydia (0->1), total-group Dermatemydidae (1->0), Trionychidae (0->1), *Araripemys barretoi* (0->1), (*Judithemys* ++ *Sinemys*) (0->1).

**Character 249 (modified from Evers & Benson [2018]: character 244)**: Epiplastra, shape: 0 = epiplastra squarish in shape and forming parts of the anterior plastral lobe; 1 = epiplastra elongate, become narrower posteriorly along the anterolateral margin of the hyoplastron, and with gently convex lateral margin; 2 = epiplastra are laterally strongy expanded to a wing-like shape; 3 = epiplastra rod-like and anteriorly as narrow as posteriorly, with concave lateral margin.

*Character optimisation*: Unambiguous: total-group Chelonioidea (0->1), Protostegidae (1->2), total-group Chelydridae (0->2), Trionychidae (0->3), *Araripemys barretoi* (0->1); DELTRAN: (*Kirgizemys* *hoburensis* + *Kirgizemys dmitrievi*) (0->1), *Judithemys* *sukhanovi* (0->1); ACCTRAN: (*Judithemys* ++ *Sinemys*) (0->1), Sinemydidae (1->0).

**Character 250 (Evers & Benson [2018]: character 245).** Epiplastra, very thick anterior lip in dorsal view: 0 = present; 1 = absent.

*Character optimisation*: Unambiguous: Testuguria (1->0), *Chelonoidis* sp. (0->1).

**Character 251 (Evers & Benson [2018]: character 246).** Hyoplastra, contacts of axillary buttresses: 0 = absent to slightly contacting peripherals only; 1 = peripherals and costal 1.

*Character optimisation*: Unambiguous: total-group Dermatemydidae (0->1), *Chrysemys picta* (0->1), Testuguria (0->1), *Chelonoidis* sp. (1->0), total-group Pleurodira (0->1); DELTRAN: *Glyptops plicatulus* (0->1); ACCTRAN: Paracryptodira (0->1).

**Character 252 (Evers & Benson [2018]: character 247).** Hyoplastra, axillary buttresses: 0 = present; 1 = ossified axillary buttresses absent.

*Character optimisation*: Unambiguous: *Platysternon megacephalum* (0->1), *Chelonoidis* sp. (0->1), Trionychidae (0->1); DELTRAN: total-group Chelonioidea (0->1), crown-group Chelydridae (0->1); ACCTRAN: Americhelydia (0->1), Kinosternoidea (1->0).

**Character 253 (Evers & Benson [2018]: character 248).** Hyoplastra, termination of axillary buttresses: 0 = terminates on peripheral 1 or 2; 1 = terminates on peripheral 3; 2 = terminates on peripheral 4 or 5 level. This character is scored as inapplicable for turtles without axillary buttresses (ch. 247.1).

*Character optimisation*: Unambiguous: Durocryptodira (0->1), Kinosternidae (1->2), Testudinidae (1->0), crown-group Pleurodira (0->1), *Elseya dentata* (1->0); DELTRAN: *Carettochelys insculpta* (0->2), Pleurosternidae (0->1), *Meiolania planiceps* (0->1); ACCTRAN: *Meiolania*+++ (0->1), Xinjiangchelyidae+++ (1->0), crown-group Trionychia (0->2).

**Character 254 (Evers & Benson [2018]: character 249).** Mesoplastron: 0 = present; 1 = absent.

*Character optimisation*: DELTRAN: Xinjiangchelyidae+++ (0->1), crown-group Pelomedusoides (1->0), *Meiolania planiceps* (0->1); ACCTRAN: *Meiolania*+++ (0->1), total-group Pelomedusoides (1->0), Paracryptodira (1->0).

**Character 255 (Evers & Benson [2018]: character 250).** Number of mesoplastra: 0 = two; 1 = one. This character is scored as inapplicable when mesoplastra are absent (ch. 249.1).

*Character optimisation*: -.

**Character 256 (Evers & Benson [2018]: character 251).** Mesoplastron, medial contact of mesoplastra: 0=present, or virtually present when a central plastral fonta- nelle is present, absence of contact between hyoplastron and hypoplastron; 1 = absent, partial contact between hyoplastron and hypoplastron present.

*Character optimisation*: DELTRAN: crown-group Pelomedusoides (0->1), *Kallokibotion bajazidi* (0->1); ACCTRAN: *Chubutemys*+++ (0->1), Paracryptodira (1->0).

**Character 257 (Evers & Benson [2018]: character 252).** Hypoplastra, contacts of inguinal buttresses: 0 = absent to slightly contacting peripherals; 1 = peripheral and costal 5; 2 = peripheral, costals 5 and 6; 3 = peripherals and costal 4.

*Character optimisation*: Unambiguous: *Baptemys wyomingensis* (0->1), *Chrysemys picta* (0->1), total-group Pleurodira (0->1), *Chelus fimbriatus* (1->3), *Chelodina* (1->0), *Araripemys barretoi* (1->0); DELTRAN: *Geoclemys hamiltonii* (0->1), *Gopherus polyphemus* (0->1), Pleurosternidae (0->1); ACCTRAN: Testuguria (0->1), (*Testudo* + *Chelonoidis*) (1->0), Paracryptodira (0->1).

**Character 258 (Evers & Benson [2018]: character 253).** Hypoplastra, termination of inguinal buttresses: 0 = peripheral 8; 1 = peripheral 7; 2 = peripheral 6.

*Character optimisation*: Unambiguous: *Platysternon megacephalum* (1->2), *Chelonoidis* sp. (0->2), *Kallokibotion bajazidi* (0->2); DELTRAN: *Macrochelys temminckii* (0->1), Kinosternidae (0->1), *Emys orbicularis* (0->1), *Geoclemys hamiltonii* (0->1), *Carettochelys insculpta* (0->1), *Chelus fimbriatus* (0->1), *Chelodina* (0->1), *Araripemys barretoi* (0->1); ACCTRAN: Cryptodira (0->1), total-group Dermatemydidae (1->0), *Chrysemys picta* (1->0), Testudinidae (1->0), *Adocus beatus* (1->0), total-group Chelidae (0->1), *Phrynops geoffroanus* (1->0), *Elseya dentata* (1->0).

**Character 259 (Evers & Benson [2018]: character 254).** Xiphiplastra, distinct anal notch: 0 = absent; 1 = present.

*Character optimisation*: Unambiguous: *Dermatemys mawii* (0->1), Testudinoidea (0->1), *Emys orbicularis* (1->0), crown-group Pleurodira (0->1).

**Character 260 (modified from Evers & Benson [2018]: character 255):** Xiphiplastra, shape of xiphiplastra: 0 = triangular, trapezoidal, or rectangular plate-like element; 1 = anteroposterior elongate rods.

*Character optimisation*: Unambiguous: Protostegidae+++ (0->1), (*Santanachelys* ++ *Rhinochelys*) (1->0), *Puppigerus camperi* (1->0), crown-group Chelydridae (0->1).

**Character 261 (modified from Evers & Benson [2018]: character 255)**: Xiphiplastra, articulation with hypoplastron: 0 = the xiphiplastra articulate with the hypoplastra along an anteriorly facing margin, forming a mediolaterally broad suture; 1 = the xiphiplastra have an elongate anterolateral process articulating along the posterolateral margin of the hypoplastron, resulting in an oblique suture, and the hypoplastra extend posteriorly along the anteromedial margin of the xiphiplastra.

*Character optimisation*: Unambiguous: total-group Chelonioidea (0->1), crown-group Chelydridae (0->1); DELTRAN: *Ordosemys* sp. IVPP V12092 (0->1), *Sinemys lens* (0->1); ACCTRAN: Sinemydidae (0->1), *Dracochelys bicuspis* (1->0).

**Character 262 (modified from Evers & Benson [2018]: character 255)**: Xiphiplastra, posteriorly in contact with one another, often sutured along the midline and forming a plastral lobe: 0 = present; 1 = absent.

*Character optimisation*: DELTRAN: crown-group Chelonioidea (0->1), (*Puppigerus* ++ *Ctenochelys*) (1->0); ACCTRAN: *Corsochelys*+++ (0->1), (*Argillochelys* ++ *Puppigerus*) (1->0).

**Character 263 (Evers & Benson [2018]: character 256).** Plastral scutes: 0 = present; 1 = absent.

*Character optimisation*: Unambiguous: crown-group Trionychia (0->1); DELTRAN: (*Protostega* + *Archelon*) (0->1), Dermochelyidae (0->1), *Allopleuron hoffmanni* (0->1); ACCTRAN: (*Ocepechelon* ++ *Protostega*) (0->1), *Corsochelys*+++ (0->1), (*Erquelinnesia* ++ *Lepidochelys*) (1->0).

**Character 264 (Evers & Benson [2018]: character 257).** Plastral scutes, midline sulcus: 0 = straight; 1 = distinctly sinuous, at least for part of its length.

*Character optimisation*: Unambiguous: Xinjiangchelyidae (0->1), *Xinjiangchelys* *wusu* (1->0); DELTRAN: *Adocus beatus* (0->1); ACCTRAN: total-group Trionychia (0->1).

**Character 265 (Evers & Benson [2018]: character 258).** Gular, number of gulars: 0 = one pair of scutes; 1 = only one scute.

*Character optimisation*: Unambiguous: total-group Pleurodira (0->1), *Pleurosternon bullockii* (0->1).

**Character 266 (Evers & Benson [2018]: character 259).** Extragulars: 0 = present; 1 = absent.

*Character optimisation*: Unambiguous: Durocryptodira (0->1), *Araripemys barretoi* (0->1), (*Judithemys* ++ *Sinemys*) (0->1).

**Character 267 (Evers & Benson [2018]: character 260).** Extagulars, medial contact: 0 = absent; 1 = present, contacting one another anterior to gular(s); 2 = present, contacting one another posterior to gular(s).

*Character optimisation*: Unambiguous: *Chelus fimbriatus* (0->2), *Chelodina* (0->1).

**Character 268 (Evers & Benson [2018]: character 261).** Extragulars, anterior plastral tuberosities: 0 = present; 1 = absent.

*Character optimisation*: DELTRAN: (*Kayentachelys*+*Eileanchelys*)+++ (0->1); ACCTRAN: *Australochelys*+++ (0->1).

**Character 269 (Evers & Benson [2018]: character 262).** Extragulars, restricted to epiplastra: 0 = present; 1 = absent, extragulars reach the entoplastron.

*Character optimisation*: Unambiguous: *Chelus fimbriatus* (0->1); DELTRAN: *Proganochelys quenstedti* (0->1); ACCTRAN: *Proganochelys quenstedti* (0->1).

**Character 270 (Evers & Benson [2018]: character 263).** Intergulars: 0 = absent; 1 = present.

*Character optimisation*: Unambiguous: Kinosterninae (0->1), *Dermatemys mawii* (0->1); DELTRAN: *Eretmochelys* *imbricata* (0->1), (*Chelonia* + *Natator*) (0->1); ACCTRAN: total-group Chelonioidea (0->1), (*Caretta* ++ *Lepidochelys*) (1->0).

**Character 271 (Evers & Benson [2018]: character 264).** Humerals, number of pairs: 0 = one pair present; 1 = two pairs present, subdivided by a plastral hinge.

*Character optimisation*: Unambiguous: Kinosterninae (0->1).

**Character 272 (Evers & Benson [2018]: character 265).** Humerals, humero-pectoral sulcus: 0 = restricted to hyoplastra; 1 = crossing the posterior portion of ento- plastron.

*Character optimisation*: Unambiguous: *Emys orbicularis* (0->1), *Geoclemys hamiltonii* (0->1), *Chelus fimbriatus* (0->1), *Podocnemis* (0->1); DELTRAN: *Chelydra serpentina* (0->1), *Baptemys wyomingensis* (0->1), *Adocus beatus* (0->1); ACCTRAN: Chelydroidea (0->1), *Dermatemys mawii* (1->0), total-group Trionychia (0->1).

**Character 273 (Evers & Benson [2018]: character 266).** Pectorals: 0 = present; 1 = absent.

*Character optimisation*: Unambiguous: Kinosternoidea (0->1).

**Character 274 (Evers & Benson [2018]: character 267).** Pectorals, antero-posteriorly developed: 0 = present; 1 = absent, very short antero-posterior development.

*Character optimisation*: Unambiguous: *Chrysemys picta* (0->1), Testuguria (0->1), *Chelonoidis* sp. (1->0); DELTRAN: *Adocus beatus* (0->1); ACCTRAN: total-group Trionychia (0->1).

**Character 275 (Evers & Benson [2018]: character 268).** Abdominals: 0 = present; 1 = absent.

*Character optimisation*: Unambiguous: *Toxochelys* sp. (0->1), Kinosternidae (0->1).

**Character 276 (Evers & Benson [2018]: character 269).** Abdominals, medial contact to one another: 0 = present; 1 = absent. This character is scored as inapplicable for turtles that lack abdominals.

*Character optimisation*: Unambiguous: total-group Chelydridae (0->1), *Araripemys barretoi* (0->1).

**Character 277 (Evers & Benson [2018]: character 270).** Anals: 0 = only cover parts of the xiphiplastra; 1 = overlap anteromedially onto the hypoplastra.

*Character optimisation*: Unambiguous: *Toxochelys* sp. (0->1), crown-group Chelydridae (0->1), Xinjiangchelyidae (0->1).

**Character 278 (Evers & Benson [2018]: character 271).** Inframarginals: 0 = present; 1 = absent.

*Character optimisation*: Unambiguous: *Toxochelys* sp. (0->1), crown-group Pleurodira (0->1), (*Sinemys* ++ *Kirgizemys*) (0->1); DELTRAN: *Adocus beatus* (0->1); ACCTRAN: total-group Trionychia (0->1).

**Character 279 (Evers & Benson [2018]: character 272).** Number of inframarginals: 0 = more than two pair present, plastral scales do not contact marginals; 1 = two pair present (axillaries and inguinals), limited contact between plastral scales and marginals present. This character is scored as inapplicable when inframarginals are absent (ch. 271.1).

*Character optimisation*: Unambiguous: *Peritresius* *martini* (0->1), Kinosternidae (0->1), *Chelonoidis* sp. (1->0); DELTRAN: *Chrysemys picta* (0->1), Testuguria (0->1); ACCTRAN: Testudinoidea (0->1), *Platysternon megacephalum* (1->0).

**Character 280 (Evers & Benson [2018]: character 273).** Cervical ribs: 0 = large cervical ribs present; 1 = cervical ribs reduced or absent.

*Character optimisation*: Unambiguous: Testudines (0->1).

**Character 281 (Evers & Benson [2018]: character 274).** Cervicals, position of the transverse processes: 0 = middle of the centrum; 1 = anterior end of the centrum.

*Character optimisation*: Unambiguous: crown-group Pleurodira (1->0); DELTRAN: Sinemydidae/Macroabenidae+++ (0->1), *Archelon* *ischyros* (1->0), (*Desmatochelys* *lowii* + *Desmatochelys* *padillai*) (1->0), *Meiolania planiceps* (0->1), *Proganochelys quenstedti* (0->1); ACCTRAN: Sinemydidae/Macroabenidae+++ (0->1), (*Desmatochelys* ++ *Protostega*) (1->0), *Protostega* *gigas* (0->1), *Meiolania planiceps* (0->1), *Proganochelys quenstedti* (0->1).

**Character 282 (Evers & Benson [2018]: character 275).** Cervicals, posterior cervicals with strongly developed ventral keels: 0 = absent or slightly developed in all vertebrae; 1 = present, more developed on posterior vertebrae.

*Character optimisation*: Unambiguous: *Platysternon megacephalum* (1->0), *Chelonoidis* sp. (1->0), *Chelus fimbriatus* (0->1); DELTRAN: Durocryptodira (0->1), *Adocus beatus* (0->1), *Leyvachelys cipadi* (0->1), (*Judithemys* ++ *Sinemys*) (0->1); ACCTRAN: Xinjiangchelyidae+++ (0->1), crown-group Trionychia (1->0), total-group Pleurodira (1->0), Sandownidae (0->1).

**Character 283 (Evers & Benson [2018]: character 276).** Cervicals, cervical 8 centrum significantly shorter than cervical 7: 0 = absent; 1 = present.

*Character optimisation*: Unambiguous: *Chelus fimbriatus* (0->1); DELTRAN: Cryptodira (0->1), (*Judithemys* ++ *Sinemys*) (0->1), *Sinemys lens* (1->0); ACCTRAN: Xinjiangchelyidae+++ (0->1), total-group Pleurodira (1->0), (*Sinemys gamera* + *Sinemys lens*) (1->0).

**Character 284 (Evers & Benson [2018]: character 277).** Cervicals, triangular diapophyses: 0 = absent; 1 = present.

*Character optimisation*: Unambiguous: crown-group Pleurodira (0->1), *Chubutemys copelloi* (0->1); DELTRAN: *Glyptops plicatulus* (0->1); ACCTRAN: Paracryptodira (0->1).

**Character 285 (Evers & Benson [2018]: character 278).** Cervicals, central articulations of cervical vertebrae: 0 = articulations not formed, cervical vertebrae am- phicoelous or platycoelous; 1 = articulations formed, cervical vertebrae procoelous or opisthocoelous.

*Character optimisation*: Unambiguous: Sinemydidae/Macroabenidae+++ (0->1), *Corsochelys* *halinches* (1->0), Thalassochelydia (1->0), *Meiolania planiceps* (0->1).

**Character 286 (Evers & Benson [2018]: character 279).** Cervicals, articulation between cervical 8 and dorsal vertebrae 1: 0 = 8 (dorsal 1; 1 = 8) dorsal 1; 2 = vertebrae articulate along zygapophyses only.

*Character optimisation*: Unambiguous: Trionychidae (1->2), *Adocus beatus* (1->0); DELTRAN: *Ctenochelys* (1->0); ACCTRAN: (*Ctenochelys* ++ *Peritresius*) (1->0).

**Character 287 (Evers & Benson [2018]: character 280).** Cervicals, biconvex cervical vertebrae in the middle of the neck: 0 = absent; 1 = present.

*Character optimisation*: Unambiguous: *Dermatemys mawii* (1->0), total-group Trionychia (1->0); DELTRAN: *Dracochelys bicuspis* (1->0); ACCTRAN: Sinemydidae (1->0).

**Character 288 (Evers & Benson [2018]: character 281).** Cervicals, biconvex cervical vertebra in the middle of the neck: 0 = cervical 2; 1 = cervical 3; 2 = cervical 4; 3 = cervical 5.

*Character optimisation*: Unambiguous: crown-group Chelidae (0->3); DELTRAN: Kinosternidae (2->1), crown-group Pleurodira (2->0); ACCTRAN: Kinosternoidea (2->1), total-group Pleurodira (2->0).

**Character 289 (Evers & Benson [2018]: character 282).** Cervicals, biconcave cervical vertebrae: 0 = absent; 1 = present.

*Character optimisation*: Unambiguous: Testudinoidea (0->1), crown-group Chelidae (0->1).

**Character 290 (Evers & Benson [2018]: character 283).** Cervicals, double articulation between cervical 5 and 6: 0 = absent; 1 = present.

*Character optimisation*: Unambiguous: *Chrysemys picta* (0->1).

**Character 291 (Evers & Benson [2018]: character 284).** Cervicals, double articulation between cervical 6 and 7: 0 = absent; 1 = present.

*Character optimisation*: Unambiguous: Cryptodira (0->1); DELTRAN: Protostegidae+++ (1->0); ACCTRAN: total-group Chelonioidea (1->0).

**Character 292 (Evers & Benson [2018]: character 285).** Cervicals, central articulation between cervical 6 and 7: 0 = cervical 6 concave ( cervical 7 convex; 1 = platycoelous, cervical 6 II cervical 7.

*Character optimisation*: DELTRAN: crown-group Chelonioidea (0->1); ACCTRAN: *Corsochelys*+++ (0->1).

**Character 293 (Evers & Benson [2018]: character 286).** Cervicals, double articulation between cervical 7 and 8: 0 = absent; 1 = present.

*Character optimisation*: Unambiguous: Cryptodira (0->1); DELTRAN: Protostegidae (1->0), *Toxochelys* sp. (1->0); ACCTRAN: total-group Chelonioidea (1->0), *Corsochelys*+++ (0->1).

**Character 294 (Evers & Benson [2018]: character 287).** Cervicals, height versus length of centra and neural arch: 0 = total height of centra and neural arch longer than the anteroposterior length of the cervical centra; 1 = total height of centra and neural arch much shorter than the anteroposterior length of the cervical centra.

*Character optimisation*: Unambiguous: Thalassochelydia (1->0); DELTRAN: Xinjiangchelyidae+++ (0->1), Dermochelyidae (1->0), *Puppigerus camperi* (1->0), *Corsochelys* *halinches* (1->0); ACCTRAN: Paracryptodira+++ (0->1), *Corsochelys*+++ (1->0), total-group Cheloniidae (0->1), (*Puppigerus* + *Eochelone*) (1->0).

**Character 295 (Evers & Benson [2018]: character 288).** Cervicals, modification of neural arch on cervical 8: 0 = neural arch without modificiation of postzygapophyses; 1 = neural arch with postzygapophyses pointing anteroventrally.

*Character optimisation*: Unambiguous: Cryptodira (0->1); DELTRAN: Protostegidae+++ (1->0), *Kirgizemys* *hoburensis* (0->1); ACCTRAN: total-group Chelonioidea (1->0), (*Kirgizemys* *hoburensis* + *Kirgizemys dmitrievi*) (0->1).

**Character 296 (Evers & Benson [2018]: character 289).** Cervicals, postzygapophyses united in midline: 0 = absent; 1 = present.

*Character optimisation*: Unambiguous: total-group Chelidae (0->1), *Phrynops geoffroanus* (1->0).

**Character 297 (Evers & Benson [2018]: character 290).** Cervicals, ventral process on cervical 8: 0 = absent; 1 = present, well developed (as tall or taller than the height of the centrum).

*Character optimisation*: Unambiguous: *Peritresius* *martini* (0->1), *Chelonoidis* sp. (1->0), *Judithemys* *sukhanovi* (0->1); DELTRAN: Chelydroidea (0->1), Emydidae (0->1), Testuguria (0->1), *Adocus beatus* (0->1); ACCTRAN: Cryptodira (0->1), total-group Chelonioidea (1->0), *Platysternon megacephalum* (1->0), crown-group Trionychia (1->0).

**Character 298 (Evers & Benson [2018]: character 291).** Cervicals, shape of central articulation of cervicals 7 and 8: 0 = as high as wide; 1 = much wider than high.

*Character optimisation*: DELTRAN: crown-group Chelonioidea (0->1), *Toxochelys* sp. (0->1); ACCTRAN: total-group Chelonioidea (0->1), Protostegidae (1->0).

**Character 299 (Evers & Benson [2018]: character 292).** Ribs, length of first dorsal rib: 0 = long, extends full length of first costal and may even contact peripherals distally; 1 = intermediate, in contact with well-developed anterior bridge buttresses; 2 = intermediate to short, extends less than halfway across first costal.

*Character optimisation*: Unambiguous: Sinemydidae/Macroabenidae+++ (0->2), Sinemydidae (2->0); DELTRAN: *Jurassichelon* *oleronensis* (2->1); ACCTRAN: Thalassochelydia (2->1).

**Character 300 (Evers & Benson [2018]: character 293).** Ribs, contact of dorsal ribs 9 and 10 with costals: 0 = present; 1 = absent.

*Character optimisation*: Unambiguous: Kinosternidae (0->1).

**Character 301 (Evers & Benson [2018]: character 294).** Dorsal rib 10: 0 = long, spanning full length of costals and contacting peripherals distally; 1 = short, not span- ning father distally than pelvis.

*Character optimisation*: DELTRAN: Sinemydidae/Macroabenidae+++ (0->1), *Glyptops plicatulus* (0->1); ACCTRAN: *Chubutemys*+++ (0->1), Xinjiangchelyidae (1->0).

**Character 302 (Evers & Benson [2018]: character 295).** Dorsals, anterior articulation of the first dorsal centrum: 0 = faces at most slightly anteroventrally; 1 = faces strongly anteroventrally.

*Character optimisation*: Unambiguous: Cryptodira (0->1), total-group Chelonioidea (1->0), crown-group Cheloniidae (0->1).

**Character 303 (Evers & Benson [2018]: character 296).** Caudals, tail club: 0 = present; 1 = absent.

*Character optimisation*: DELTRAN: Paracryptodira+++ (0->1), *Kayentachelys* *aprix* (0->1); ACCTRAN: *Australochelys*+++ (0->1), *Meiolania planiceps* (1->0).

**Character 304 (Evers & Benson [2018]: character 297).** Caudals, anterior caudal centra: 0 = amphicoelous; 1 = procoelous or platycoelous; 2 = opisthocoelous.

*Character optimisation*: Unambiguous: *Macrochelys temminckii* (1->2); DELTRAN: Sinemydidae/Macroabenidae+++ (0->1), *Meiolania planiceps* (0->2); ACCTRAN: *Chubutemys*+++ (0->1), *Meiolania planiceps* (1->2).

**Character 305 (Evers & Benson [2018]: character 298).** Caudals, posterior caudal centra: 0 = amphicoelous; 1 = procoelous or platycoelous: 2 = opisthocoelous.

*Character optimisation*: Unambiguous: Testudines (2->1), *Platysternon megacephalum* (1->2), *Chelonoidis* sp. (1->2); DELTRAN: *Meiolania*+++ (0->2), crown-group Chelydridae (1->2); ACCTRAN: *Chubutemys*+++ (0->2), total-group Chelydridae (1->2).

**Character 306 (Evers & Benson [2018]: character 299).** Caudals, chevrons: 0 = present on nearly all caudal vertebrae: 1 = absent, or only poorly developed, along the posterior caudal vertebrae.

*Character optimisation*: Unambiguous: *Toxochelys* sp. (1->0), *Platysternon megacephalum* (1->0), *Chelonoidis* sp. (1->0); DELTRAN: Sinemydidae/Macroabenidae+++ (0->1), crown-group Chelydridae (1->0); ACCTRAN: Paracryptodira+++ (0->1), total-group Chelydridae (1->0).

**Character 307 (Evers & Benson [2018]: character 300).** Caudals, tail ring: 0 = absent; 1 = present.

*Character optimisation*: Unambiguous: *Meiolania planiceps* (0->1).

**Character 308 (Evers & Benson [2018]: character 301).** Scapula, anterodorsal ridge of acromion: 0 = present; 1 = absent.

*Character optimisation*: DELTRAN: (*Kayentachelys*+*Eileanchelys*)+++ (0->1); ACCTRAN: *Australochelys*+++ (0->1).

**Character 309 (Evers & Benson [2018]: character 302).** Scapula, ventral ridge of acromion: 0 = present; 1 = absent developed proximally near glenoid.

*Character optimisation*: Unambiguous: *Chubutemys*+++ (0->1).

**Character 310 (Evers & Benson [2018]: character 303).** Scapula, horizontal ridge of acromion: 0 = well-developed, coracoid foramen present; 1 = reduced, only developed along distal portion of acromion.

*Character optimisation*: DELTRAN: (*Kayentachelys*+*Eileanchelys*)+++ (0->1); ACCTRAN: *Australochelys*+++ (0->1).

**Character 311 (Evers & Benson [2018]: character 304).** Scapula, glenoid neck on scapula: 0 = absent; 1 = present.

*Character optimisation*: Unambiguous: total-group Pleurodira (0->1), *Pelomedusa subrufa* (1->0); DELTRAN: *Glyptops plicatulus* (0->1); ACCTRAN: Paracryptodira (0->1).

**Character 312 (Evers & Benson [2018]: character 305).** Scapula, lamina between the dorsal process of the scapula and the acromion: 0 = well developed; 1 = reduced; 2 = absent.

*Character optimisation*: Unambiguous: Paracryptodira+++ (0->2), *Kallokibotion bajazidi* (0->1).

**Character 313 (Evers & Benson [2018]: character 306).** Scapula, internal angle between acromion process and scapular process ≥110°: 0 = absent; 1 = present.

*Character optimisation*: Unambiguous: Protostegidae+++ (0->1), (*Caretta* ++ *Lepidochelys*) (1->0).

**Character 314 (Evers & Benson [2018]: character 307).** Coracoid, coracoid vs humerus length: 0 = shorter than humerus; 1 = at least as long as humerus.

*Character optimisation*: Unambiguous: Protostegidae+++ (0->1).

**Character 315 (Evers & Benson [2018]: character 308).** Coracoid, foramen: 0 = present; 1 = absent.

*Character optimisation*: DELTRAN: (*Kayentachelys*+*Eileanchelys*)+++ (0->1); ACCTRAN: *Australochelys*+++ (0->1).

**Character 316 (Evers & Benson [2018]: character 309).** Cleithrum: 0 = present; 1 = absent.

*Character optimisation*: Unambiguous: Sinemydidae/Macroabenidae+++ (0->1); DELTRAN: *Ctenochelys* (1->0); ACCTRAN: (*Ctenochelys* ++ *Peritresius*) (1->0).

**Character 317 (Evers & Benson [2018]: character 310).** Cleithrum, contact with carapace: 0 = present; 1 = osseous contact with carapace absent.

*Character optimisation*: DELTRAN: (*Kayentachelys*+*Eileanchelys*)+++ (0->1); ACCTRAN: *Australochelys*+++ (0->1).

**Character 318 (Evers & Benson [2018]: character 311).** Pelvis, pelvis-shell attachment: 0 = pelvis-shell attachment by ligaments; 1 = pelvis attached by strong sutural contact of the ischium and pubis with the plastron, and illium with the carapace.

*Character optimisation*: Unambiguous: crown-group Pleurodira (0->1).

**Character 319 (Evers & Benson [2018]: character 312).** Pelvis, thyroid fenestra: 0 = coalescent; 1 = two separated fenestra completely or partially separated.

*Character optimisation*: Unambiguous: *Peritresius* *martini* (0->1), *Chelonoidis* sp. (1->0); DELTRAN: *Protostega* *gigas* (1->0), Dermochelyidae (1->0), (*Puppigerus* ++ *Lepidochelys*) (1->0), *Toxochelys* sp. (1->0), crown-group Chelydridae (1->0), *Staurotypus* (1->0), *Platysternon megacephalum* (1->0), crown-group Trionychia (1->0), crown-group Pleurodira (1->0); ACCTRAN: Paracryptodira+++ (1->0), *Archelon* *ischyros* (0->1), total-group Cheloniidae (0->1), (*Puppigerus* ++ *Lepidochelys*) (1->0), Kinosternoidea (0->1), *Staurotypus* (1->0), Testudinoidea (0->1), *Platysternon megacephalum* (1->0), *Adocus beatus* (0->1).

**Character 320 (Evers & Benson [2018]: character 313).** Ilium, elongated iliac neck: 0 = absent; 1 = present.

*Character optimisation*: DELTRAN: (*Kayentachelys*+*Eileanchelys*)+++ (0->1); ACCTRAN: *Australochelys*+++ (0->1).

**Character 321 (Evers & Benson [2018]: character 314).** Ilium, iliac scar: 0 = extends from costals onto the peripherals and pygal; 1 = positioned on costals only.

*Character optimisation*: -.

**Character 322 (Evers & Benson [2018]: character 315).** Ilium, shape of the ilium articular site on the visceral surface of the carapace: 0 = narrow and pointed poste- riorly; 1 = oval.

*Character optimisation*: -.

**Character 323 (Evers & Benson [2018]: character 316).** Ilium, posterior notch in acetabulum: 0 = absent; 1 = present.

*Character optimisation*: DELTRAN: Kinosternidae (0->1), *Baptemys wyomingensis* (0->1); ACCTRAN: Kinosternoidea (0->1), *Dermatemys mawii* (1->0).

**Character 324 (Evers & Benson [2018]: character 317).** Ilium, thelial process: 0 = absent; 1 = present.

*Character optimisation*: Unambiguous: *Emarginachelys cretacea* (0->1), Kinosternidae (0->1), total-group Trionychia (0->1); DELTRAN: *Apalone spinifera* (1->0); ACCTRAN: Trionychinae (1->0).

**Character 325 (Evers & Benson [2018]: character 318).** Pubis, lateral process: 0 = small, poorly developed, columnar; 1 = well developed and flat.

*Character optimisation*: Unambiguous: crown-group Trionychia (0->1); DELTRAN: Protostegidae+++ (0->1), Kinosternoidea (0->1), *Plesiochelys* *bigleri* (0->1); ACCTRAN: Americhelydia (0->1), total-group Chelydridae (1->0), Plesiochelyidae (0->1).

**Character 326 (Evers & Benson [2018]: character 319).** Pubis, epipubis process: 0 = osseus or calcified; 1 = cartilaginous or absent.

*Character optimisation*: Unambiguous: *Kinosternon suburum hippocrepis* (0->1); DELTRAN: Testudines (0->1), *Puppigerus camperi* (1->0), (*Oligochelone* + *Erquelinnesia*) (1->0), *Macrochelys temminckii* (1->0), Kinosternoidea (1->0); ACCTRAN: Paracryptodira+++ (0->1), (*Erquelinnesia* ++ *Lepidochelys*) (1->0), crown-group Cheloniidae (0->1), Chelydroidea (1->0), *Chelydra serpentina* (0->1).

**Character 327 (Evers & Benson [2018]: character 320).** Ischium, ischial contacts with plastron: 0 = contact via a large central tubercle; 1 = contact via two separate ischial processes.

*Character optimisation*: DELTRAN: (*Kayentachelys*+*Eileanchelys*)+++ (0->1); ACCTRAN: *Australochelys*+++ (0->1).

**Character 328 (Evers & Benson [2018]: character 321).** Ischium, lateral process of ischium or metischial process: 0 = absent; 1 = present.

*Character optimisation*: Unambiguous: Protostegidae+++ (1->0), crown-group Trionychia (1->0), crown-group Pleurodira (1->0); DELTRAN: (*Puppigerus* ++ *Ctenochelys*) (0->1), Kinosternidae (1->0); ACCTRAN: (*Argillochelys* ++ *Puppigerus*) (0->1), Kinosternoidea (1->0).

**Character 329 (Evers & Benson [2018]: character 322).** Hypoischium: 0 = present; 1 = absent.

*Character optimisation*: DELTRAN: (*Kayentachelys*+*Eileanchelys*)+++ (0->1); ACCTRAN: *Australochelys*+++ (0->1).

**Character 330 (Evers & Benson [2018]: character 323).** Humerus, ectepicondylar foramen: 0 = in a channel; 1 = only a groove.

*Character optimisation*: Unambiguous: (*Puppigerus* ++ *Lepidochelys*) (0->1), *Eochelone brabantica* (1->0); DELTRAN: Americhelydia (0->1), crown-group Chelonioidea (1->0), Emydidae (0->1), *Geoclemys hamiltonii* (0->1), *Testudo* (0->1), Trionychidae (0->1), total-group Pleurodira (0->1); ACCTRAN: Testudines (0->1), *Corsochelys*+++ (1->0), *Platysternon megacephalum* (1->0), Testudinidae (1->0), *Testudo* (0->1), total-group Trionychia (1->0), Trionychidae (0->1).

**Character 331 (Evers & Benson [2018]: character 324).** Humerus, proximal articular surface of humerus: 0 = with shoulder on preaxial side, upturned; 1 = without shoulder, not upturned.

*Character optimisation*: Unambiguous: Trionychidae (0->1), *Podocnemis* (0->1); DELTRAN: (*Desmatochelys* ++ *Protostega*) (0->1), crown-group Chelonioidea (0->1), *Chelodina* (0->1); ACCTRAN: Protostegidae+++ (0->1), *Corsochelys* *halinches* (1->0), Chelodininae (0->1).

**Character 332 (Evers & Benson [2018]: character 325).** Humerus, lateral process of humerus: 0 = abuts caput humeri; 1 = slightly separated from caput humeri; 2 = located distal to caput humeri but along proximal end of shaft; 3 = located at middle of humeral shaft.

*Character optimisation*: Unambiguous: total-group Chelonioidea (0->2), Dermochelyidae (2->3), *Corsochelys* *halinches* (2->1); DELTRAN: (*Protostega* + *Archelon*) (2->3), *Ctenochelys* (2->0), *Sinemys gamera* (0->1); ACCTRAN: (*Ocepechelon* ++ *Protostega*) (2->3), (*Ctenochelys* ++ *Peritresius*) (2->0), (*Sinemys gamera* + *Sinemys lens*) (0->1).

**Character 333 (Evers & Benson [2018]: character 330).** Humerus, prominent anterior projection of lateral process: 0 = absent; 1 = present.

*Character optimisation*: Unambiguous: Dermochelyidae (0->1).

**Character 334 (new character)**: Humerus, distal articulation: 0 = articular surface forms distinct trochlea; 1 = rounded epiphyseal surface without clearly defined articulation facets.

*Character optimisation*: Unambiguous: Protostegidae+++ (0->1), *Erquelinnesia gosseleti* (1->0), *Geoclemys hamiltonii* (0->1), crown-group Trionychia (0->1); DELTRAN: Thalassochelydia (0->1), *Elseya dentata* (0->1), crown-group Pelomedusoides (0->1); ACCTRAN: total-group Pleurodira (0->1), total-group Chelidae (1->0), *Elseya dentata* (0->1).

**Character 335 (Evers & Benson [2018]: character 331).** Humerus, length of the humerus versus the width of the proximal end: 0 = two times or less the width of the proximal end: 1 = more than two times the width of the proximal end.

*Character optimisation*: Unambiguous: Paracryptodira+++ (0->1).

**Character 336 (Evers & Benson [2018]: character 332).** Humerus, scar for Muscle latissimus dorsi and Muscle teres major: 0 = located anterior to humeral shaft; 1 =

located at middle of shaft.

*Character optimisation*: Unambiguous: crown-group Cheloniidae (0->1); DELTRAN: (*Protostega* + *Archelon*) (0->1); ACCTRAN: (*Ocepechelon* ++ *Protostega*) (0->1).

**Character 337 (Evers & Benson [2018]: character 333).** Humerus, humerus length vs femur length: 0 = shorter than femur; 1 = longer than femur.

*Character optimisation*: Unambiguous: total-group Chelonioidea (0->1).

**Character 338 (Evers & Benson [2018]: character 334).** Ulna, contact with radius through rugosity and ridge: 0 = absent; 1 = present.

*Character optimisation*: Unambiguous: total-group Chelonioidea (0->1); DELTRAN: *Santanachelys* *gaffneyi* (1->0), *Dermochelys coriacea* (1->0); ACCTRAN: (*Santanachelys* + *Notochelone*) (1->0), Dermochelyidae (1->0).

**Character 339 (Evers & Benson [2018]: character 335).** Radius, curves towards anterior: 0 = absent; 1 = present.

*Character optimisation*: Unambiguous: Protostegidae (0->1).

**Character 340 (Evers & Benson [2018]: character 336).** Manus, phalangeal formula of the manus: 0 = most digits with two shortenened phalanges: 1 = most digits with three elongated phalanges.

*Character optimisation*: DELTRAN: Xinjiangchelyidae+++ (0->1), *Testudo* (1->0), *Gopherus polyphemus* (1->0); ACCTRAN: Paracryptodira+++ (0->1), Testudinidae (1->0), *Chelonoidis* sp. (0->1).

**Character 341 (Evers & Benson [2018]: character 337).** Manus, rigid articulations in 1^st^ and 2^nd^ digit: 0 = absent; 1 = present.

*Character optimisation*: Unambiguous: Protostegidae+++ (0->1); DELTRAN: *Santanachelys* *gaffneyi* (1->0); ACCTRAN: (*Santanachelys* + *Notochelone*) (1->0).

**Character 342 (Evers & Benson [2018]: character 338).** Manus, rigid articulations in 3^rd^ to 5^th^ digit: 0 = absent; 1 = present.

*Character optimisation*: Unambiguous: total-group Chelonioidea (0->1).

**Character 343 (Evers & Benson [2018]: character 339).** Manus, flippers: 0 = absent; 1 = short flippers present; 2 = elongate flippers present. Joyce (2007: ch 134, Manus C).

*Character optimisation*: DELTRAN: Trionychidae (0->1), *Carettochelys insculpta* (0->2); ACCTRAN: crown-group Trionychia (0->1), total-group Carettochelyidae (1->2).

**Character 344 (Evers & Benson [2018]: character 340).** Ulnare, size of the ulnare vs the intermedium: 0 = smaller than intermedium: 1 = nearly as large as intermedium; 2 = much larger than intermedium.

*Character optimisation*: DELTRAN: Testudines (0->1), Protostegidae+++ (1->2), *Allopleuron*+++ (2->1), *Carettochelys insculpta* (1->2), *Meiolania planiceps* (0->1); ACCTRAN: *Australochelys*+++ (0->1), total-group Chelonioidea (1->2), total-group Cheloniidae (2->1), total-group Carettochelyidae (1->2), Xinjiangchelyidae (1->0).

**Character 345 (new character)**: Size of proximal carpals vs. distal carpals: 0 = proximal carpals are of similar size with respect to distal carpals; 1 = proximal carpals are much larger than distal carpals.

*Character optimisation*: Unambiguous: Protostegidae+++ (0->1), *Emarginachelys cretacea* (0->1), Emysternia (0->1), crown-group Trionychia (0->1), *Podocnemis* (0->1); DELTRAN: *Sternotherus* (0->1), *Chelus fimbriatus* (0->1), Chelodininae (0->1), *Xinjiangchelys* *wusu* (0->1); ACCTRAN: Kinosternidae (0->1), crown-group Chelidae (0->1), *Phrynops geoffroanus* (1->0), Xinjiangchelyidae (0->1).

**Character 346 (new character)**: Relative lengths of manual phalanges on the 3^rd^ and 4^th^ digit: 0 = the 1^st^ phalanx is longer than or equally long as the 2^nd^ phalanx; 1 = the 2^nd^ phalanx is longer than the 1^st^ phalanx. This character is scored as inapplicable when the manus digits only have two phalanges (i.e. the second phalanx is an ungual).

*Character optimisation*: Unambiguous: *Elseya dentata* (0->1), *Araripemys barretoi* (0->1), *Podocnemis* (0->1); DELTRAN: *Allopleuron*+++ (0->1), *Carettochelys insculpta* (0->1); ACCTRAN: total-group Cheloniidae (0->1), total-group Carettochelyidae (0->1).

**Character 347 (new character)**: 3^rd^ phalanx on 5^th^ manual digit: 0 = absent; 1 = present.

*Character optimisation*: Unambiguous: Chelodininae (1->0); DELTRAN: Xinjiangchelyidae+++ (0->1), crown-group Chelonioidea (1->0), *Emys orbicularis* (1->0), *Apalone spinifera* (1->0), *Carettochelys insculpta* (1->0), crown-group Pelomedusoides (1->0); ACCTRAN: Paracryptodira+++ (0->1), *Corsochelys*+++ (1->0), Emydidae (1->0), (*Apalone* + *Petrochelys*) (1->0), total-group Carettochelyidae (1->0), total-group Pelomedusoides (1->0).

**Character 348 (new character)**: Longest digit in the manus: 0 = 4^th^ digit; 1 = 3^rd^ digit. This character is scored as inapplicable when the 3^rd^ and 4^th^ digits are equally long.

*Character optimisation*: DELTRAN: crown-group Chelonioidea (0->1); ACCTRAN: *Corsochelys*+++ (0->1).

**Character 349 (Evers & Benson [2018]: character 341).** Pes, number of digits: 0 = five; 1 = four.

*Character optimisation*: DELTRAN: *Macrochelys temminckii* (0->1), *Testudo* (0->1), *Gopherus polyphemus* (0->1); ACCTRAN: total-group Chelydridae (0->1), Testudinidae (0->1), *Chelonoidis* sp. (1->0).

**Character 350 (Evers & Benson [2018]: character 342).** Manus and Pes, flattening of carpals and tarsal elements: 0 = absent; 1 = present.

*Character optimisation*: Unambiguous: total-group Chelonioidea (0->1).

**Character 351 (Evers & Benson [2018]: character 343).** Manus and Pes, hyperphalangy manus digits 4 and 5, pes digit 4: 0 = absent; 1 = present.

*Character optimisation*: Unambiguous: Trionychidae (0->1).

**Character 352 (modified from Evers & Bensons [2018]: character 344)**: Femur, femoral trochanters: 0 = distinct, and separated from one another; 1 = connected by a ridge.

*Character optimisation*: Unambiguous: Protostegidae+++ (0->1).

**Character 353 (modified from Evers & Bensons [2018]: character 344)**: Femur, intertrochanteric ridge: 0 = ridge is low and concave, creating a notch between the major and minor trachenter; 1 = ridge is high and obliterates intertrochanteric notch, and the proximal surface of the trochanters and their connecting ridge forms a continuous surface. This character is scored as inapplicable when an intertrochanteric ridge is absent (character 352.0)

*Character optimisation*: Unambiguous: *Dermochelys coriacea* (0->1), crown-group Cheloniidae (0->1).

**Character 354 (new character)**: Femur, connection between femoral head surface and the major trochanter: 0 = the femoral head and major trochanter have distinct proximal surfaces separated by a deep notch; 1 = the femoral head surface slopes toward the major trochanter and forms a continuous proximal surface with it.

*Character optimisation*: Unambiguous: Protostegidae+++ (0->1); DELTRAN: *Meiolania*+++ (1->0), *Geoclemys hamiltonii* (0->1), Thalassochelydia (0->1); ACCTRAN: *Chubutemys*+++ (1->0), Testuguria (0->1), Angolachelonia (0->1).

**Character 355 (Evers & Benson [2018]: character 345).** Tibia, tibial pit for pubotibialis and flexor tibialis internus muscles: 0 = absent; 1 = present.

*Character optimisation*: Unambiguous: *Natator* *depressus* (0->1); DELTRAN: (*Puppigerus* ++ *Ctenochelys*) (0->1); ACCTRAN: (*Argillochelys* ++ *Puppigerus*) (0->1).

**REFERENCES**

CADENA, E. and PARHAM, J. F. 2015. Oldest known marine turtle? A new protostegid from the Lower Cretaceous of Colombia. *PaleoBios*, **32**, 1–421.

EVERS, S. W. and BENSON, R. B. J. 2018. A new phylogenetic hypothesis of turtles with implications for the number of evolutionary transitions to marine lifestyles supports an Early Cretaceous origin and rapid diversification of Chelonioidea. *Palaeontology*, 1–42.

HOOKS, G. E. 1998. Systematic revision of the Protostegidae, with a redescription of *Calcarichelys gemma* Zangerl, 1953. *Journal of Vertebrate Paleontology*, **18**, 85–98.
